# Base-promoted highly efficient synthesis of nitrile-substituted cyclopropanes *via* Michael-initiated ring closure†

**DOI:** 10.1039/d2ra05393d

**Published:** 2022-10-06

**Authors:** Min Ye, Fan Xu, Yun Bai, Fanglian Zhang, Wenjia Wang, Yiping Qian, Zhengwang Chen

**Affiliations:** Key Laboratory of Organo-pharmaceutical Chemistry of Jiangxi Province, Gannan Normal University 341000 China yemin@gnnu.edu.cn chenzwang2021@163.com +86 797-8793670 +86 797-8793670

## Abstract

A convenient and efficient annulation reaction has been developed for the general synthesis of dinitrile-substituted cyclopropanes in moderate to excellent yields. A variety of 2-arylacetonitriles and α-bromoennitriles were compatible under the standard conditions. The reaction was achieved through tandem Michael-type addition followed by intramolecular cyclization. The preliminary application of this method was confirmed by the synthesis of the 2,4-dioxo-3-azabicyclo[3.1.0]hexane scaffold.

## Abstract 2

An efficient base-promoted synthesis of nitrile-substituted cyclopropanes from 2-arylacetonitriles and α-bromoennitriles under mild conditions *via* Michael-initiated ring closure.

## Graphical abstract

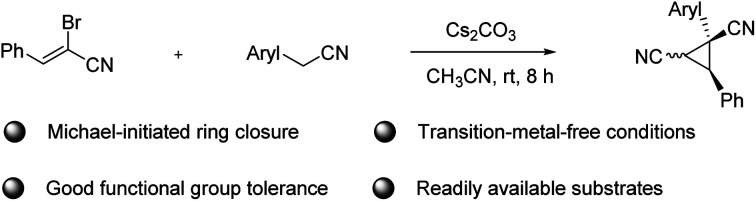

Substituted cyclopropanes, as attractive structural units, are commonly found in a variety of natural products and biologically active compounds.^1^ The strained structure, interesting bonding characteristics, and value as an internal mechanistic probe of the cyclopropane subunit have attracted the attention of the physical organic community.^2^ As a consequence, considerable efforts have been made to develop new and effective approaches toward cyclopropane derivatives.^3^ Classical approaches to cyclopropane synthesis are the Simmons–Smith cyclopropanation.^4^ Transition-metal-catalyzed cyclopropanation of alkenes with diazo compounds represents a direct protocol for their preparation.^5^ Furthermore, the new types of cyclopropanation reactions based on nucleophilic addition-ring closure sequence were well documented (Scheme 1a and b).^6^ Nitrile-substituted cyclopropanes are of great interest as they are versatile templates for the rapid formation of biologically active and synthetically useful functionalized cyclopropane derivatives.^7^ Recently, nitrile-substituted cyclopropanes were synthesized *via* transition-metal-catalyzed olefin functionalization with diazoacetonitriles.^8^ Despite the significant advancement, the development of complementary strategy toward functionalized cyclopropanes by using readily available substrates and cheap agents with high efficiency would be highly desirable.

**Scheme 1:**
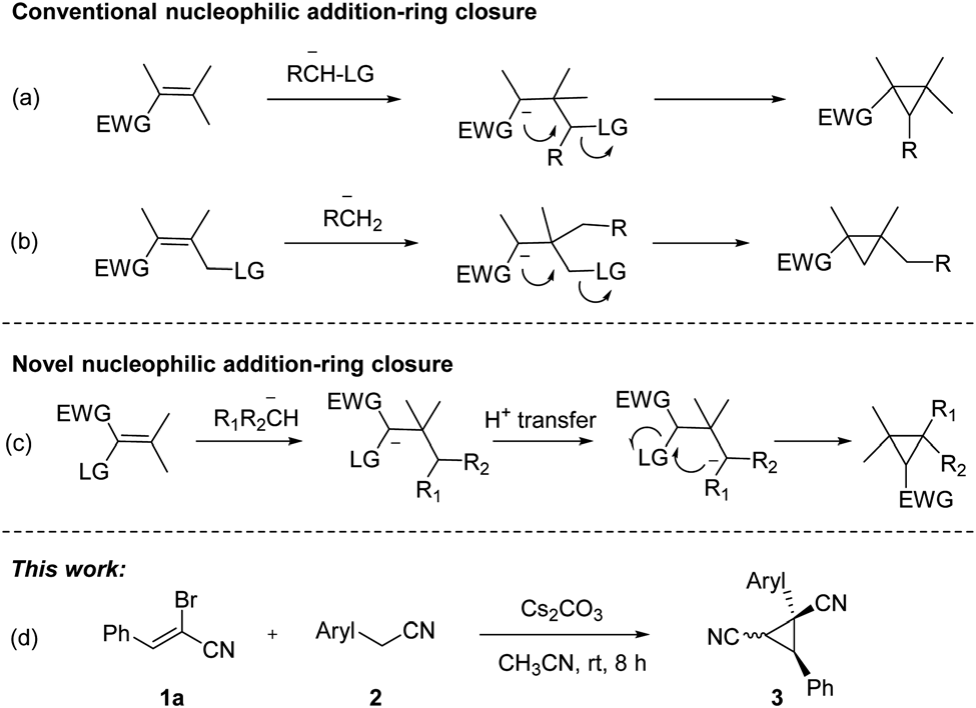
Methods for synthesis of cyclopropane derivatives.

α-Bromoennitrile is a class of readily available intermediate in organic synthesis.^9^ However, this intermediate is rarely used in organic synthesis compared to its analog α-bromoenal.^10^ Recently, our group has reported a series of functionalization of 2-arylacetonitriles and their derivatives.^11^ We hypothesized five-membered nitrogen containing heterocycles could be formed from 2-pyridylacetonitrile and α-bromoennitriles *via* [3 + 2] annulation. However, dinitrile-substituted cyclopropanes were afforded through a novel Michael-initiated ring closure procedure (Scheme 1c). Herein, we present a base-promoted synthesis of dinitrile-substituted cyclopropanes from 2-arylacetonitriles and α-bromoennitriles under mild conditions *via* Michael-initiated ring closure (Scheme 1d).

Initially, (*Z*)-2-bromo-3-phenylacrylonitrile 1a and 2-pyridylacetonitrile 2a were selected as the model substrates for the condition optimization. As illustrated in Table 1, a variety of commonly used organic and inorganic bases were screened. Among the tested organic bases, only DBU was found to afford the desired product in 38% yield (Table 1, entries 1–4). Then various inorganic bases were examined. All of the inorganic bases had some effect on the reaction. We were pleased to find Cs_2_CO_3_ was the most suitable base and furnished the corresponding product in 95% yield (Table 1, entries 5–9). The control experiment revealed that the base was indispensable for the cyclization reaction, no product was produced without the addition of the base (Table 1, entry 10). Having this promising result, we subsequently evaluated the effects of several solvents. Solvent screening indicated that besides MeCN, DMF also promoted this reaction, whereas the use of DMSO, H_2_O, DCE, THF and dioxane resulted in significantly lower yields (Table 1, entries 11–16). Finally, the reaction temperatures were investigated, and decreasing or increasing the temperatures led to diminishing yields (Table 1, entries 17–18). The *cis*/*trans* isomers ratio of the product was the same for 0° and room temperature. Therefore, base, solvent and reaction temperature are all essential for this transformation.

**Table 1:** Optimization of the reaction conditions^a^

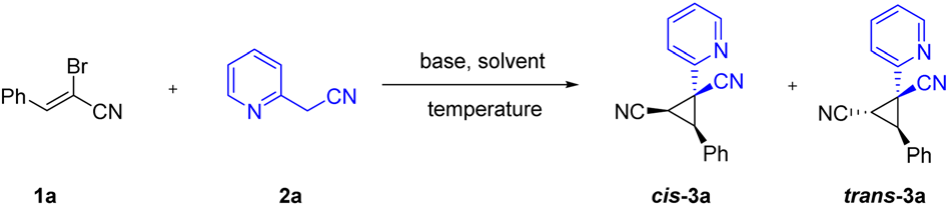				
Entry	Base	Solvent	Temp.	Yield^b^ (%)
1	DABCO	MeCN	rt	Trace
2	DBU	MeCN	rt	38
3	TEA	MeCN	rt	np
4	DMAP	MeCN	rt	np
5	Cs_2_CO_3_	MeCN	rt	95
6	K_2_CO_3_	MeCN	rt	67
7	NaOAc	MeCN	rt	42
8	K_3_PO_4_	MeCN	rt	89
9	KO^*t*^Bu	MeCN	rt	84
10	—	MeCN	rt	np
11	Cs_2_CO_3_	DMF	rt	87
12	Cs_2_CO_3_	DMSO	rt	36
13	Cs_2_CO_3_	H_2_O	rt	np
14	Cs_2_CO_3_	DCE	rt	67
15	Cs_2_CO_3_	THF	rt	np
16	Cs_2_CO_3_	Dioxane	rt	53
17	Cs_2_CO_3_	MeCN	0	62
18	Cs_2_CO_3_	MeCN	50	np

a Reaction conditions: 1a (0.2 mmol), 2a (0.2 mmol) and base (1.5 equiv.) in solvent (1.0 mL) for 12 h.

b Yields of isolated *cis*-3a and *trans*-3a are given. *Cis* refers the two nitriles positioned on the same face of the cyclopropane; *trans* refers the two nitriles positioned on the opposite face of the cyclopropane.

Having the developed optimal conditions for the Michael-initiated ring closure reaction, the substrate scope was investigated. As illustrated in Scheme 2, a wide range of 2-arylacetonitriles were tolerated with (*Z*)-2-bromo-3-phenylacrylonitrile 1a to render dinitrile-substituted cyclopropanes in moderate to excellent yields (3a–3u). Except for 2-pyridylacetonitrile, 3-pyridyl and 4-pyridyl derivatives also reacted smoothly to generate the products in good yields (3a–3c). The annulation with 2-pyridylacetonitrils bearing electron-donating groups and withdrawing groups in the pyridine ring worked well to deliver the products in satisfactory yields (3d–3h). Thienyl derivatives were reactive to afford the corresponding products, but exhibited lower reactivity compared with pyridyl (3i–3j). In addition to heteroaryl-substituted substrates, various 2-arylacetonitriles were further tested. The reaction conditions were compatible with an array of substituents, such as alkyl, methoxy, phenyl, chloro, bromo, trifluoromethyl, fluoro, and cyano groups (3k–3u). In particular, the aryl bromide could be further functionalized in metal-catalyzed cross-coupling reactions and hold the enormous potential application in pharmaceutical and materials science (3p–3q). To our delight, nitrile-containing substrate could provide the product 3u in 86% yield. Significantly, the annulation reaction could be carried out on large-scale synthesis and formed the product 3a in 87% yield. The structure of *cis*-3a was verified by X-ray crystal analysis (CCDC: 2141258†).

**Scheme 2:**
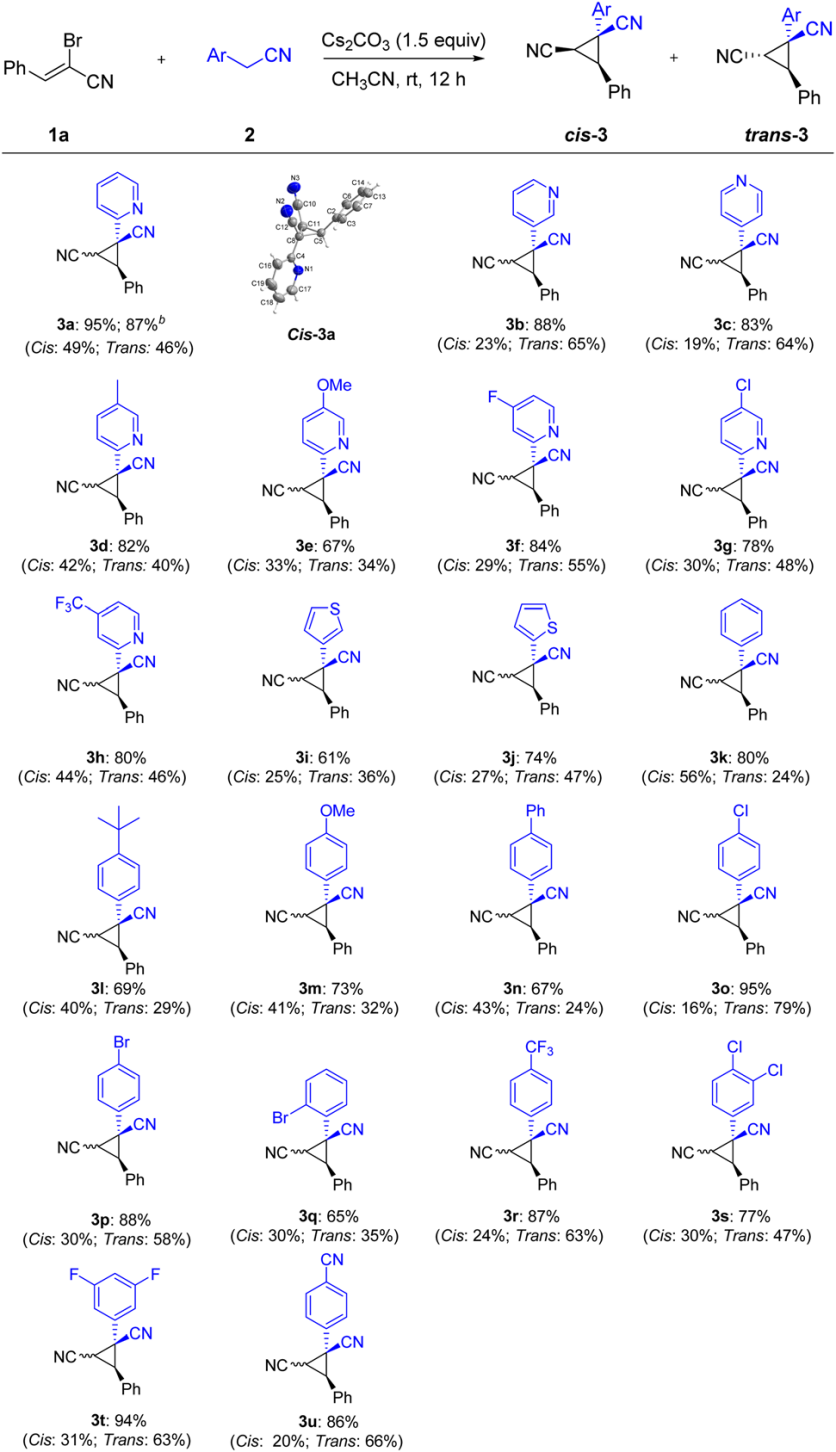
Substrate scope of 2-arylacetonitriles^*a*^. ^*a*^Reaction conditions: 1a (0.2 mmol), 2 (0.2 mmol), Cs_2_CO_3_ (1.5 equiv.) and CH_3_CN (1.0 mL) at room temperature for 12 h; isolated yields are given unless otherwise noted. *Cis* refers the two nitriles positioned on the same face of the cyclopropane; *trans* refers the two nitriles positioned on the opposite face of the cyclopropane. ^*b*^2 mmol scale.

Continuing to examine the generality and scope of the annulation reaction, we explored various α-bromoennitriles under the standard conditions (Scheme 3). α-Bromoennitriles bearing electron-rich or electron-deficient groups on the benzene ring reacted successfully with 2-pyridylacetonitrile to achieve the desired products in good yields (4a–4h). The substrates bearing a methyl at the *ortho*- and *meta*-positions of the benzene ring were suitable substrates for the transformation, thus indicating the steric hindrance is negligible (4a–4b). The bulky *tert*-butyl group was accommodated in this transformation (4c and 4j). The disubstituted α-bromoennitrile proved to be good substrate under the same reaction conditions (4d). The reaction of the fused ring system also yielded the products in satisfactory yields (4f). Finally, phenylacetonitriles also worked well with α-bromoennitriles to obtain the corresponding product in good yields (4i–4m). The structure of *tran*s-4b was further confirmed by X-ray crystal diffraction measurements (CCDC: 2142244†). It implied that this Michael-initiated ring closure reaction can be effective for the construction of dinitrile-substituted cyclopropane library.

**Scheme 3:**
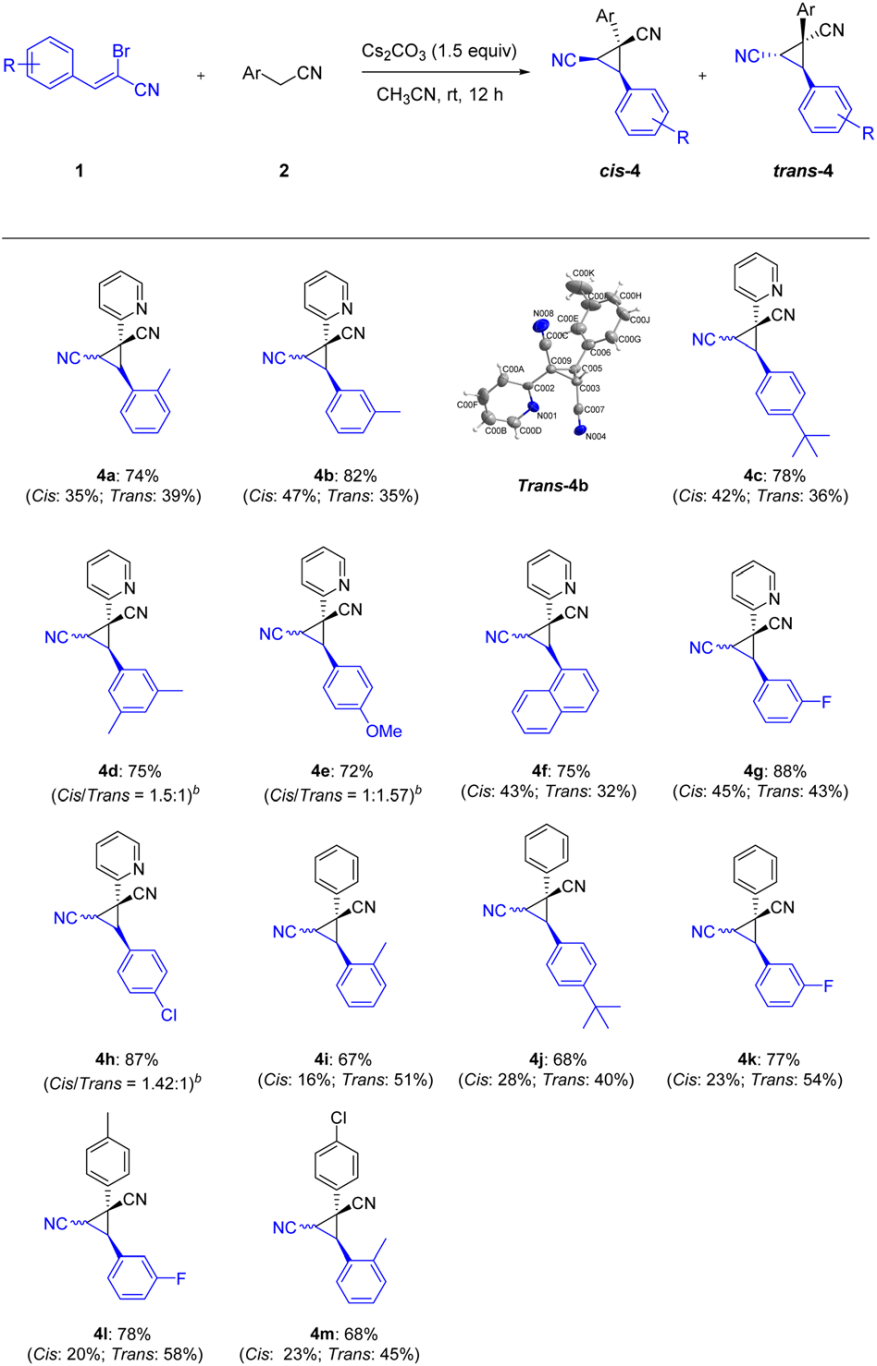
Synthesis of nitrile-substituted cyclopropanes^*a*^. ^*a*^Reaction conditions: 1 (0.2 mmol), 2 (0.2 mmol), Cs_2_CO_3_ (1.5 equiv.) and CH_3_CN (1.0 mL) at room temperature for 12 h; isolated yields are given unless otherwise noted. *Cis* refers the two nitriles positioned on the same face of the cyclopropane; *trans* refers the two nitriles positioned on the opposite face of the cyclopropane. ^*b*^The *cis*/*trans* (isomer) ratio was determined by crude ^1^H NMR.

To illustrate the applicability of this reaction, further transformation of product *cis*-3a was carried out as depicted in Scheme 4. 2,4-Dioxo-3-azabicyclo[3.1.0]hexane scaffold is known to be an important pharmacology agent and synthons for synthesis of functionally substituted cyclopropanes and various spirocompounds.^12^ The target compound 5a can be readily accessible in excellent yield *via* a simple hydrolysis reaction. It is worth noting that similar result was obtained for *trans*-3a.

**Scheme 4:**
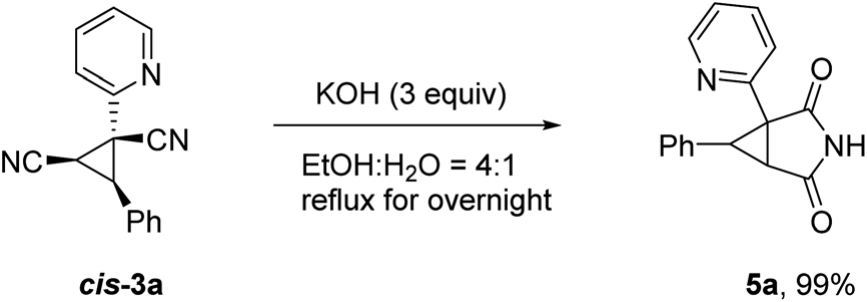
Synthetic application.

A tentative mechanism for cyclopropane formation was proposed and outlined in Scheme 5 on the basis of aforementioned results as well as our experimental observations. Initially, carbanion intermediate B was produced *via* the sequential extraction of hydrogen proton and Michael-type addition process. Then the intermediate B was converted into intermediate C through 1,3-hydride transfer. Finally, the dinitrile-substituted cyclopropane 3a was formed through intramolecular nucleophilic substitution. The diastereomer 3a is the favored product due to steric effects, in which the two aryl groups are located on the opposite face of the plane of the cyclopropane moiety.

**Scheme 5:**
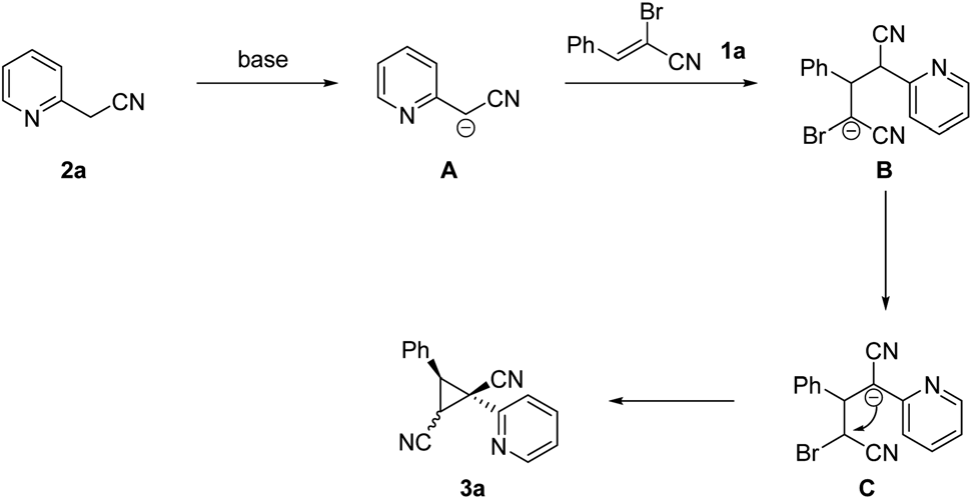
Possible reaction mechanism.

In summary, we have explored a convenient and highly efficient annulation reaction of 2-arylacetonitriles and α-bromoennitriles. A wide range of dinitrile-substituted cyclopropanes were obtained in moderate to excellent yields through a novel Michael-initiated ring closure procedure. The advantages of this transformation include readily accessible substrates, transition-metal-free conditions, good functional group tolerance, simple operation, *etc.* In addition, nitrile-substituted products have potential applications in synthetic and pharmaceutical chemistry. Further synthetic utilization and asymmetric transformations are currently ongoing in our laboratory.

## Conflicts of interest

There are no conflicts to declare.

## Supplementary Material

RA-012-D2RA05393D-s001

RA-012-D2RA05393D-s002
