# Supplementary material for: Base-promoted highly efficient synthesis of nitrile-substituted cyclopropanes via Michael-initiated ring closure
Source: RSC Adv. 2022 Oct 6;12(44):28576–9. doi: 10.1039/d2ra05393d (PMC9536251; doi:10.1039/d2ra05393d)
Supplement: RA-012-D2RA05393D-s001 [file RA-012-D2RA05393D-s001.pdf]

Electronic Supplementary Material (ESI) for RSC Advances.
This journal is © The Royal Society of Chemistry 2022

Base-promoted high efficient synthesis of nitrile-substituted cyclopropanes via Michael-initiated ring closure

Min Ye,* Fan Xu, Yun Bai, Fanglian Zhang, Wenjia Wang, Yiping Qian and Zhengwang Chen*

Key Laboratory of Organo-Pharmaceutical Chemistry of Jiangxi Province, Gannan Normal University, Ganzhou 341000, PR China.

Supporting Information

List of Contents

A. General method S2

B. Preparation of starting materials S2

C. General procedure for the synthesis of cyclopropane-1,2-dicarbonitriles S2

D. Analytical data S3

E. Reference S20

F. Copies of ^{1}H and ^{13}C NMR spectra S21

G. X-ray crystallographic data S87

A. General method

Melting points were investigated using a melting point instrument and are uncorrected. ^1H and ^{13}C NMR spectra were obtained on a 400 MHz for ^1H NMR and 100 MHz for ^{13}C NMR. The chemical shifts are referenced to signals at 7.26 and 77.0 ppm, respectively, chloroform is solvent with TMS as the internal standard unless otherwise noted. High resolution mass spectra (HRMS) (TOF) were measured using an electrospray ionization (ESI) mass spectrometry. Silica gel (300-400 mesh) was used for flash column chromatograph, eluting (unless otherwise stated) with ethyl acetate/petroleum ether (PE) (60-90 ºC) mixture.

B. Preparation of starting materials

The route toward 2-bromo-3-arylacrylonitriles:

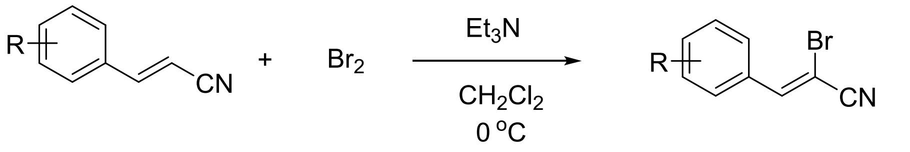

Method: Following a known procedure,[1] substituted 2-bromo-3-arylacrylonitriles were synthesized. All are known compounds and its spectral data were in good with the corresponding literature vulues.

To a solution of cinnamonitrile A’ (5 mmol) in DCM (10 mL) was added Br_2 (0.96 g, 6 mmol, 1.2 equiv) at 0 ºC. The reaction mixture was stirred for 15 min, followed by the addition of Et_3N (1.2 mL, 8.5 mmol, 1.7 equiv). The resulting mixture was stirred 2 h at 0 ºC, then the solution was diluted with DCM and washed with a 10% Na_2SO_3 solution, H_2O and brine. The organic layer was dried over Na_2SO_4, filtered, and concentrated to yield orange oil. The crude residue was purified by flash chromatography (PE/EA 30:1) to afford desired product **1**.

C. General procedure for the synthesis of cyclopropane-1,2-dicarbonitriles

A mixture of 2-arylacetonitrile (0.2 mmol), Cs_2CO_3 (98 mg, 1.5 eq), and 2-bromo-3-arylacrylonitrile (0.2 mmol) in CH_3CN (1.0 mL) was stirred in a preheated oil bath at 25 °C for 12 h in a sealed tube under air. After the reaction was finished, water (5 mL) was added and the solution was extracted with ethyl acetate (3×5 mL), and the combined extract was dried with anhydrous $MgSO_4$. Solvent was removed, and the residue was separated by column chromatography to give the pure sample.

D. Analytical data

3-phenyl-1-(pyridin-2-yl)cyclopropane-1,2-dicarbonitrile (3a)

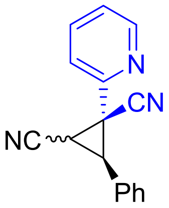

Cis isomer: Yellow solid; mp = 138-140 °C; R_f = 0.41 (petroleum ether / ethyl acetate = 4:1). ^{1}H NMR (400 MHz, $CDCl_3$) δ = 8.52 (d, J = 4.3 Hz, 1H), 7.87 (d, J = 7.8 Hz, 1H), 7.78 (t, J = 7.7 Hz, 1H), 7.58 (d, J = 7.2 Hz, 2H), 7.42 (dt, J = 19.1, 6.8 Hz, 3H), 7.29 (dd, J = 7.1, 5.0 Hz, 1H), 3.61 (d, J = 9.2 Hz, 1H), 3.30 (d, J = 9.2 Hz, 1H). ^{13}C NMR (100 MHz, $CDCl_3$) δ = 149.8, 149.7, 137.3, 130.3, 129.0, 128.8, 128.7, 123.5, 121.4, 115.3, 114.7, 37.4, 29.8, 21.0.

Trans isomer: Yellow solid; mp = 128-130 °C; R_f = 0.51 (petroleum ether / ethyl acetate = 4:1). ^{1}H NMR (400 MHz, $CDCl_3$): δ = 8.67 (d, J = 4.7 Hz, 1H), 7.85 – 7.78 (m, 2H), 7.46 – 7.40 (m, 3H), 7.38 – 7.32 (m, 3H), 4.37 (d, J = 7.7 Hz, 1H), 3.03 (d, J = 7.7 Hz, 1H). ^{13}C NMR (100 MHz, $CDCl_3$): δ = 149.3, 148.3, 137.2, 131.3, 128.8, 128.7, 127.9, 123.7, 122.6, 116.3, 114.5, 35.4, 30.0, 21.0.
HRMS (ESI): calcd. for $C_{16}H_{12}N_3$ $[M + H]^+$ 246.1026, found 246.1028.

3-phenyl-1-(pyridin-3-yl)cyclopropane-1,2-dicarbonitrile (3b)

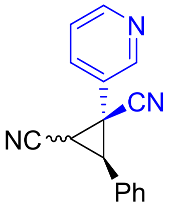

Cis isomer: Yellow liquid; R_f = 0.32 (petroleum ether / ethyl acetate = 1:1). ^{1}H NMR (400 MHz, $CDCl_3$) δ = 8.75 – 8.64 (m, 2H), 7.79 (ddd, J = 8.0, 2.5, 1.6 Hz, 1H), 7.57 (d, J = 7.1 Hz, 2H), 7.50 – 7.38 (m, 4H), 3.31 (d, J = 9.2 Hz, 1H), 2.76 (d, J = 9.2 Hz, 1H). ^{13}C NMR (100 MHz, $CDCl_3$) δ = 150.7, 147.8, 147.8, 134.7, 129.4, 129.4, 129.2, 129.2, 129.1, 129.1, 129.0, 124.0, 115.1, 114.1, 35.4, 27.2, 20.4.

Trans isomer: White solid; mp = 166-168 °C; R_f = 0.32 (petroleum ether / ethyl acetate = 2:1). ^{1}H NMR (400 MHz, $CDCl_3$): δ = 8.67 (d, J = 4.7 Hz, 1H), 7.85 – 7.78 (m, 2H), 7.46 – 7.40 (m, 3H), 7.38 – 7.32 (m, 3H), 4.37 (d, J = 7.7 Hz, 1H), 3.03 (d, J = 7.7 Hz, 1H). ^{13}C NMR (100 MHz, $CDCl_3$): δ = 151.0, 149.6, 136.1, 130.4, 129.3, 129.2, 127.9, 127.9, 127.9, 126.8, 123.9, 116.1, 114.8, 35.4, 26.8, 19.3.
HRMS (ESI): calcd. for $C_{16}H_{12}N_3$ $[M + H]^+$ 246.1026, found 246.1026.

3-phenyl-1-(pyridin-2-yl)cyclopropane-1,2-dicarbonitrile (3c)

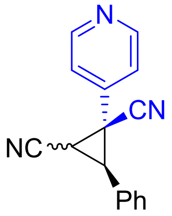

Cis isomer: Yellow solid; mp = 156-158 °C; R_f = 0.62 (petroleum ether / ethyl acetate = 1:1). ^{1}H NMR (400 MHz, $CDCl_3$) δ = 8.72 (d, J = 6.0 Hz, 2H), 7.54 (dd, J = 7.4, 1.3 Hz, 2H), 7.50 – 7.42 (m, 3H), 7.33 (dd, J = 4.6, 1.6 Hz, 2H), 3.32 (d, J = 9.3 Hz, 1H), 2.81 (d, J = 9.3 Hz, 1H). ^{13}C NMR (100 MHz, $CDCl_3$) δ = 151.0, 141.6, 129.5, 129.3, 129.2, 129.1, 120.1, 114.5, 113.8, 36.7, 28.5, 21.4.

Trans isomer: Yellow solid; mp = 145-147 °C; R_f = 0.32 (petroleum ether / ethyl acetate = 2:1). ^{1}H NMR (400 MHz, $CDCl_3$): δ = 8.76 (d, J = 5.8 Hz, 2H), 7.46 (ddd, J = 11.2, 4.7, 2.8 Hz, 5H), 7.37 – 7.33 (m, 2H), 3.60 (d, J = 7.4 Hz, 1H), 3.05 (d, J = 7.4 Hz, 1H). ^{13}C NMR (100 MHz, $CDCl_3$): δ = 150.8, 139.0, 130.2, 129.5, 129.3, 127.9, 122.0, 115.6, 114.3, 77.3, 77.0, 76.7, 35.9, 28.4, 20.4.
HRMS (ESI): calcd. for $C_{16}H_{12}N_3$ $[M + H]^+$ 246.1026, found 246.1026.

1-(5-methylpyridin-2-yl)-3-phenylcyclopropane-1,2-dicarbonitrile (3d)

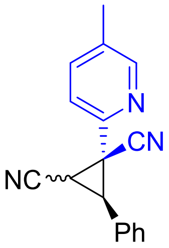

Cis isomer: White solid; mp = 152-154 °C; R_f = 0.48 (petroleum ether / ethyl acetate = 4:1). ^{1}H NMR (400 MHz, $CDCl_3$) δ = 8.35 (s, 1H), 7.76 (d, J = 8.0 Hz, 1H), 7.62 – 7.54 (m, 3H), 7.48 – 7.39 (m, 3H), 3.57 (d, J = 9.2 Hz, 1H), 3.25 (d, J = 9.2 Hz, 1H), 2.37 (s, 3H). ^{13}C NMR (100 MHz, $CDCl_3$) δ = 150.3, 147.2, 137.8, 133.5, 130.5, 129.1, 129.0, 128.9, 121.1, 115.5, 114.9, 37.4, 29.7, 21.0, 18.1.

Trans isomer: Pink solid; mp = 149-151 °C; R_f = 0.54 (petroleum ether / ethyl acetate = 4:1). ^{1}H NMR (400 MHz, $CDCl_3$): δ = 8.49 (dd, J = 1.2, 0.6 Hz, 1H), 7.72 (d, J = 8.0 Hz, 1H), 7.62 (dd, J = 8.0, 1.6 Hz, 1H), 7.46 – 7.39 (m, 3H), 7.37 – 7.34 (m, 2H), 4.35 (d, J = 7.6 Hz, 1H), 2.96 (d, J = 7.6 Hz, 1H), 2.39 (s, 3H). ^{13}C NMR (100 MHz, $CDCl_3$): δ = 149.9, 145.5, 137.8, 133.8, 131.5, 129.0, 128.9, 128.0, 122.3, 116.6, 114.7, 35.2, 29.9, 21.1, 18.1.
HRMS (ESI): calcd. for $C_{17}H_{14}N_3$ $[M + H]^+$ 260.1182, found 260.1181.

1-(5-methoxypyridin-2-yl)-3-phenylcyclopropane-1,2-dicarbonitrile (3e)

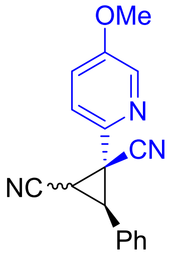

Cis isomer: White solid; mp = 112-114 °C; R_f = 0.33 (petroleum ether / ethyl acetate = 4:1). ^{1}H NMR (400 MHz, $CDCl_3$) δ = 8.20 (d, J = 2.8 Hz, 1H), 7.79 (d, J = 8.6 Hz, 1H), 7.55 (d, J = 7.2 Hz, 2H), 7.48 – 7.39 (m, 3H), 7.28 (dd, J = 8.7, 2.9 Hz, 1H), 3.89 (s, 3H), 3.54 (d, J = 9.2 Hz, 1H), 3.19 (d, J = 9.2 Hz, 1H). ^{13}C NMR (100 MHz, $CDCl_3$) δ = 155.8, 141.7, 138.1, 130.6, 129.1, 129.0, 128.9, 122.0, 121.0, 115.7, 114.9, 55.9, 37.1, 29.4, 20.9.

Trans isomer: Yellow solid; mp = 147-149 °C; R_f = 0.41 (petroleum ether / ethyl acetate = 4:1). ^{1}H NMR (400 MHz, $CDCl_3$): δ = 8.34 (d, J = 2.8 Hz, 1H), 7.74 (d, J = 8.6 Hz, 1H), 7.42 (tdd, J = 6.7, 4.5, 2.5 Hz, 3H), 7.37 – 7.33 (m, 2H), 7.30 (dd, J = 8.7, 2.9 Hz, 1H), 4.30 (d, J = 7.5 Hz, 1H), 3.90 (s, 3H), 2.92 (d, J = 7.5 Hz, 1H). ^{13}C NMR (100 MHz, $CDCl_3$): δ = 155.9, 140.0, 137.3, 131.6, 129.1, 128.9, 128.0, 123.4, 121.5, 116.8, 114.9, 55.8, 35.1, 29.7, 21.0.
HRMS (ESI): calcd. for $C_{17}H_{14}N_3O$ $[M + H]^+$ 276.1131, found 276.1132.

1-(4-fluoropyridin-2-yl)-3-phenylcyclopropane-1,2-dicarbonitrile (3f)

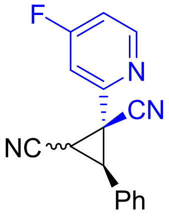

Cis isomer: Yellow solid; mp = 167-169 °C; R_f = 0.72 (petroleum ether / ethyl acetate = 1:1). ^{1}H NMR (400 MHz, $CDCl_3$) δ = 8.35 (d, J = 5.3 Hz, 1H), 7.50 (ddd, J = 19.2, 12.3, 6.4 Hz, 5H), 7.23 (d, J = 5.3 Hz, 1H), 7.00 (s, 1H), 3.35 (d, J = 9.3 Hz, 1H), 2.84 (d, J = 9.3 Hz, 1H). ^{13}C NMR (100 MHz, $CDCl_3$) δ = 164.5 (d, J = 199 Hz), 149.4 (d, J = 16 Hz), 147.3 (d, J = 8 Hz), 129.7, 129.4, 129.1, 128.9, 118.0 (d, J = 5 Hz), 114.1, 113.4, 106.8 (d, J = 40 Hz), 37.1, 28.4 (d, J = 4 Hz), 21.8.

Trans isomer: Yellow solid; mp = 139-141 °C; R_f = 0.43 (petroleum ether / ethyl acetate = 4:1). ^{1}H NMR (400 MHz, $CDCl_3$): δ = 8.34 (d, J = 5.3 Hz, 1H), 7.56 – 7.45 (m, 5H), 7.22 (d, J = 5.3 Hz, 1H), 7.00 (s, 1H), 3.35 (d, J = 9.3 Hz, 1H), 2.84 (d, J = 9.3 Hz, 1H). ^{13}C NMR (100 MHz, $CDCl_3$): δ = 164.0 (d, J = 239 Hz), 149.0 (d, J = 16 Hz), 144.5 (d, J = 9 Hz), 129.9, 129.6, 129.3, 127.9, 119.7 (d, J = 4 Hz), 115.2, 114.1, 108.7 (d, J = 39 Hz), 36.3, 28.1 (d, J = 4 Hz), 20.7.
HRMS (ESI): calcd. for $C_{16}H_{11}FN_3$ $[M + H]^+$ 264.0932, found 264.0929.

1-(5-chloropyridin-2-yl)-3-phenylcyclopropane-1,2-dicarbonitrile (3g)

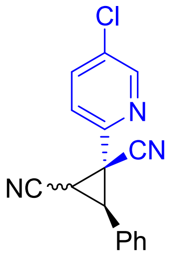

Cis isomer: Brown solid; mp = 215-217 °C; R_f = 0.33 (petroleum ether / ethyl acetate = 5:1). ^{1}H NMR (400 MHz, $CDCl_3$) δ = 8.47 (dd, J = 2.7, 0.5 Hz, 1H), 7.75 (dd, J = 8.4, 2.7 Hz, 1H), 7.57 – 7.52 (m, 2H), 7.50 – 7.40 (m, 4H), 3.28 (d, J = 9.3 Hz, 1H), 2.75 (d, J = 9.3 Hz, 1H). ^{13}C NMR (100 MHz, $CDCl_3$) δ = 152.7, 147.8, 137.5, 129.5, 129.3, 129.2, 129.1, 128.1, 125.0, 114.9, 114.0, 35.5, 26.6, 20.5.

Trans isomer: Yellow solid; mp = 126-128 °C; R_f = 0.42 (petroleum ether / ethyl acetate = 4:1). ^{1}H NMR (400 MHz, $CDCl_3$): δ = 8.62 (d, J = 2.6 Hz, 1H), 7.85 (dd, J = 8.4, 2.7 Hz, 1H), 7.50 – 7.42 (m, 4H), 7.38 – 7.33 (m, 2H), 3.54 (d, J = 7.1 Hz, 1H), 3.01 (d, J = 7.1 Hz, 1H). ^{13}C NMR (100 MHz, $CDCl_3$): δ = 153.1, 149.6, 138.7, 130.2, 129.5, 129.3, 127.9, 125.9, 125.0, 115.8, 114.6, 35.7, 26.1, 19.5.
HRMS (ESI): calcd. for $C_{16}H_{11}ClN_3$ $[M + H]^+$ 280.0636, found 280.0634.

3-phenyl-1-(4-(trifluoromethyl)pyridin-2-yl)cyclopropane-1,2-dicarbonitrile (3h)

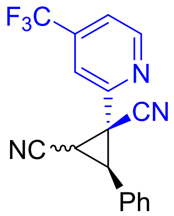

Cis isomer: Brown solid; mp = 138-140 °C; R_f = 0.49 (petroleum ether / ethyl acetate = 4:1). ^{1}H NMR (400 MHz, $CDCl_3$) δ = 8.75 (d, J = 5.0 Hz, 1H), 8.10 (s, 1H), 7.57 (d, J = 6.7 Hz, 3H), 7.50 – 7.41 (m, 3H), 3.65 (d, J = 9.3 Hz, 1H), 3.34 (d, J = 9.3 Hz, 1H). ^{13}C NMR (100 MHz, $CDCl_3$) δ = 152.1, 151.1, 140.0 (q, $J_{C,F}$ = 34 Hz), 129.9, 129.2, 129.1, 129.1, 122.2 (q, $J_{C,F}$ = 272 Hz), 119.4 (q, $J_{C,F}$ = 4 Hz), 117.5 (q, $J_{C,F}$ = 4 Hz), 114.7, 114.3, 38.3, 30.1, 21.8.

Trans isomer: Yellow solid; mp = 113-115 °C; R_f = 0.63 (petroleum ether / ethyl acetate = 4:1). ^{1}H NMR (400 MHz, $CDCl_3$): δ = 8.88 (d, J = 5.0 Hz, 1H), 8.06 (s, 1H), 7.61 (dd, J = 5.0, 0.7 Hz, 1H), 7.48 – 7.42 (m, 3H), 7.35 (dd, J = 7.4, 1.7 Hz, 2H), 4.38 (d, J = 7.8 Hz, 1H), 3.09 (d, J = 7.8 Hz, 1H). ^{13}C NMR (100 MHz, $CDCl_3$): δ = 150.6, 150.3, 139.9 (q, $J_{C,F}$ = 34 Hz), 130.8, 129.3, 129.2, 128.0, 122.2 (q, $J_{C,F}$ = 272 Hz), 119.6 (q, $J_{C,F}$ = 3 Hz), 118.6 (q, $J_{C,F}$ = 3 Hz), 115.8, 114.1, 36.4, 30.1, 21.7.
HRMS (ESI): calcd. for $C_{17}H_{11}F_3N_3$ $[M + H]^+$ 314.0900, found 314.0901.

3-phenyl-1-(thiophen-3-yl)cyclopropane-1,2-dicarbonitrile (3i)

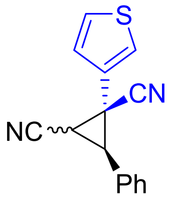

Cis isomer: Yellow solid; mp = 102-104 °C; R_f = 0.38 (petroleum ether / ethyl acetate = 4:1). ^{1}H NMR (400 MHz, $CDCl_3$) δ = 7.56 (d, J = 7.3 Hz, 2H), 7.49 – 7.41 (m, 5H), 7.05 (dd, J = 5.0, 1.5 Hz, 1H), 3.26 (d, J = 9.1 Hz, 1H), 2.66 (d, J = 9.1 Hz, 1H). ^{13}C NMR (100 MHz, $CDCl_3$) δ = 133.8, 129.9, 129.2, 129.1, 129.1, 128.5, 124.6, 123.47, 115.7, 114.4, 36.1, 25.7, 21.2.

Trans isomer: White solid; mp = 118-120 °C; R_f = 0.62 (petroleum ether / ethyl acetate = 4:1). ^{1}H NMR (400 MHz, $CDCl_3$): δ = 7.58 – 7.55 (m, 1H), 7.50 – 7.41 (m, 4H), 7.36 (d, J = 6.4 Hz, 2H), 7.25 (d, J = 1.3 Hz, 1H), 3.47 (d, J = 7.1 Hz, 1H), 2.89 (d, J = 7.2 Hz, 1H). ^{13}C NMR (100 MHz, $CDCl_3$): δ = 131.0, 129.3, 128.0, 128.0, 127.7, 126.1, 125.1, 116.6, 115.3, 37.1, 25.5, 20.2.
HRMS (ESI): calcd. For $C_{15}H_{10}N_2NaS$ $[M + Na]^+$ 273.0457, found 273.0456.

3-phenyl-1-(thiophen-2-yl)cyclopropane-1,2-dicarbonitrile (3j)

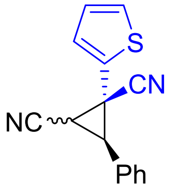

Cis isomer: Brown solid; mp = 108-110 °C; R_f = 0.38 (petroleum ether / ethyl acetate = 4:1). ^{1}H NMR (400 MHz, $CDCl_3$) δ = 7.57 (d, J = 7.2 Hz, 2H), 7.50 – 7.41 (m, 3H), 7.34 (dd, J = 5.2, 1.1 Hz, 1H), 7.26 – 7.24 (m, 1H), 7.04 (dd, J = 5.1, 3.7 Hz, 1H), 3.33 (d, J = 9.2 Hz, 1H), 2.73 (d, J = 9.2 Hz, 1H). ^{13}C NMR (100 MHz, $CDCl_3$) δ = 136.1, 129.6, 129.2, 129.1, 129.1, 127.7, 127.6, 126.6, 115.2, 114.1, 37.2, 25.2, 22.4.

Trans isomer: Yellow solid; mp = 144-146 °C; R_f = 0.56 (petroleum ether / ethyl acetate = 4:1). ^{1}H NMR (400 MHz, $CDCl_3$): δ = 7.49 – 7.42 (m, 4H), 7.39 – 7.35 (m, 3H), 7.11 (dd, J = 5.2, 3.7 Hz, 1H), 3.56 (d, J = 7.2 Hz, 1H), 2.95 (d, J = 7.2 Hz, 1H). ^{13}C NMR (100 MHz, $CDCl_3$): δ = 133.0, 130.6, 129.3, 129.2, 129.0, 127.9, 127.7, 127.7, 116.2, 115.0, 37.9, 24.7, 21.1.
HRMS (ESI): calcd. for $C_{15}H_{11}N_2S$ $[M + H]^+$ 251.0637, found 251.0635.

1,3-diphenylcyclopropane-1,2-dicarbonitrile (3k)

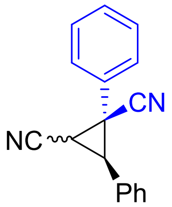

Cis isomer: Yellow solid; mp = 117-119 °C; R_f = 0.41 (petroleum ether / ethyl acetate = 4:1). ^{1}H NMR (400 MHz, $CDCl_3$) δ = 7.59 (d, J = 7.3 Hz, 2H), 7.51 – 7.43 (m, 8H), 3.28 (d, J = 9.2 Hz, 1H), 2.71 (d, J = 9.2 Hz, 1H). ^{13}C NMR (100 MHz, $CDCl_3$) δ = 132. 9, 130.1, 129.6, 129.5, 129.2, 129.2, 126.6, 115.8, 114.6, 35.6, 29.3, 20.4.

Trans isomer: White solid; mp = 118-120 °C; R_f = 0.61 (petroleum ether / ethyl acetate = 4:1). ^{1}H NMR (400 MHz, $CDCl_3$): δ = 7.59 (dd, J = 8.0, 1.5 Hz, 2H), 7.54 – 7.39 (m, 8H), 3.55 (d, J = 7.0 Hz, 1H), 2.91 (d, J = 7.0 Hz, 1H). ^{13}C NMR (100 MHz, $CDCl_3$): δ = 131.2, 130.4, 130.1, 129.6, 129.3, 129.2, 128.5, 128.0, 117.0, 115.2, 35.7, 29.2, 19.7.
HRMS (ESI): calcd. for $C_{17}H_{13}N_2$ $[M + H]^+$ 245.1073, found 245.1070.

1-(4-(*tert*-butyl)phenyl)-3-phenylcyclopropane-1,2-dicarbonitrile (3l)

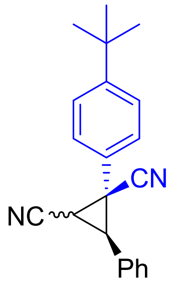

Cis isomer: Yellow solid; mp = 122-124 °C; R_f = 0.62 (petroleum ether / ethyl acetate = 4:1). ^{1}H NMR (400 MHz, $CDCl_3$) δ = 7.61 – 7.57 (m, 2H), 7.50 – 7.43 (m, 5H), 7.40 – 7.36 (m, 2H), 3.27 (d, J = 9.1 Hz, 1H), 2.68 (d, J = 9.1 Hz, 1H), 1.34 (s, 9H). ^{13}C NMR (100 MHz, $CDCl_3$) δ = 152.9, 130.2, 129.9, 129.2, 129.1, 129.1, 126.5, 126.3, 116.0, 114.7, 35.5, 34.7, 31.1, 29.0, 20.4.

Trans isomer: Yellow solid; mp = 151-153 °C; R_f = 0.70 (petroleum ether / ethyl acetate = 4:1). ^{1}H NMR (400 MHz, $CDCl_3$): δ = 7.51 (s, 4H), 7.49 – 7.42 (m, 3H), 7.41 – 7.38 (m, 2H), 3.52 (d, J = 7.0 Hz, 1H), 2.88 (d, J = 7.0 Hz, 1H), 1.35 (s, 9H). ^{13}C NMR (100 MHz, $CDCl_3$): δ = 153.2, 131.4, 129.2, 129.1, 128.1, 128.0, 127.3, 126.5, 117.1, 115.3, 35.8, 34.8, 31.2, 28.9, 19.7.
HRMS (ESI): calcd. for $C_{21}H_{20}N_2Na$ $[M + Na]^+$ 323.1519, found 323.1524.

1-(4-methoxyphenyl)-3-phenylcyclopropane-1,2-dicarbonitrile (3m)

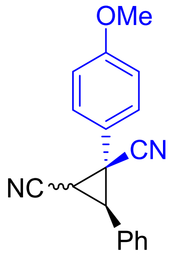

Cis isomer: Yellow liquid; R_f = 0.48 (petroleum ether / ethyl acetate = 4:1). ^{1}H NMR (400 MHz, $CDCl_3$) δ = 7.58 (d, J = 7.4 Hz, 2H), 7.49 – 7.42 (m, 3H), 7.38 – 7.35 (m, 2H), 6.97 – 6.94 (m, 2H), 3.83 (s, 3H), 3.22 (d, J = 9.1 Hz, 1H), 2.63 (d, J = 9.1 Hz,

1H). ^{13}C NMR (100 MHz, $CDCl_3$) δ = 160.4, 130.2, 129.1, 129.1, 129.0, 128.3, 124.8, 116.2, 114.9, 114.8, 55.4, 35.3, 28.7, 20.2.

Trans isomer: Brown liquid; R_f = 0.47 (petroleum ether / ethyl acetate = 2:1). ^{1}H NMR (400 MHz, $CDCl_3$): δ = 7.51 – 7.48 (m, 2H), 7.47 – 7.43 (m, 3H), 7.40 – 7.37 (m, 2H), 7.03 – 6.98 (m, 2H), 3.85 (s, 3H), 3.48 (d, J = 6.9 Hz, 1H), 2.85 (d, J = 6.9 Hz, 1H). ^{13}C NMR (100 MHz, $CDCl_3$): δ = 160.7, 131.3, 130.0, 129.6, 129.2, 129.2, 129.1, 128.0, 122.3, 117.2, 115.4, 114.9, 55.4, 35.8, 28.6, 19.6.
HRMS (ESI): calcd. for $C_{18}H_{15}N_2O$ $[M + H]^+$ 275.1179, found 275.1183.

1-([1,1'-biphenyl]-4-yl)-3-phenylcyclopropane-1,2-dicarbonitrile (3n)

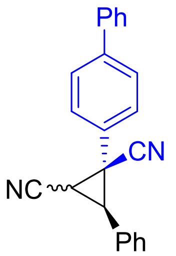

Cis isomer: Yellow solid; mp = 146-148 °C; R_f = 0.55 (petroleum ether / ethyl acetate = 4:1). ^{1}H NMR (400 MHz, $CDCl_3$) δ = 7.68 (d, J = 8.4 Hz, 2H), 7.60 (t, J = 6.9 Hz, 4H), 7.54 – 7.41 (m, 8H), 3.32 (d, J = 9.2 Hz, 1H), 2.74 (d, J = 9.2 Hz, 1H). ^{13}C NMR (100 MHz, $CDCl_3$) δ = 142.6, 139.5, 131.7, 130.1, 129.2, 129.0, 128.2, 128.0, 127.1, 127.0, 115.8, 114.6, 35.7, 29.1, 20.6.

Trans isomer: Yellow solid; mp = 170-172 °C; R_f = 0.61 (petroleum ether / ethyl acetate = 4:1). ^{1}H NMR (400 MHz, $CDCl_3$): δ = 7.75 – 7.70 (m, 2H), 7.67 – 7.60 (m, 4H), 7.51 – 7.39 (m, 8H), 3.59 (d, J = 7.0 Hz, 1H), 2.94 (d, J = 7.0 Hz, 1H). ^{13}C NMR (100 MHz, $CDCl_3$): δ = 143.0, 139.7, 131.2, 129.3, 129.3, 129.2, 128.9, 128.9, 128.2, 128.1, 128.0, 127.2, 117.0, 115.3, 35.9, 29.0, 19.8.
HRMS (ESI): calcd. for $C_{23}H_{17}N_2$ $[M + H]^+$ 321.1386, found 321.1392.

1-(4-chlorophenyl)-3-phenylcyclopropane-1,2-dicarbonitrile (3o)

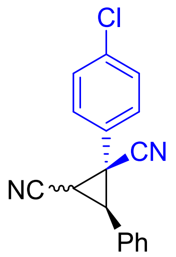

Cis isomer: Yellow solid; mp = 191-193 °C; R_f = 0.43 (petroleum ether / ethyl acetate = 4:1). ^{1}H NMR (400 MHz, $CDCl_3$) δ = 7.57 (d, J = 7.2 Hz, 2H), 7.51 – 7.38 (m, 7H), 3.24 (d, J = 9.2 Hz, 1H), 2.68 (d, J = 9.2 Hz, 1H). ^{13}C NMR (100 MHz, $CDCl_3$) δ = 135.8, 131.5, 129.8, 129.8, 129.3, 129.2, 129.1, 128.1, 115.5, 114.3, 35.7, 28.8, 20.6.

Trans isomer: Yellow solid; mp = 127-129 °C; R_f = 0.60 (petroleum ether / ethyl acetate = 4:1). ^{1}H NMR (400 MHz, $CDCl_3$): δ = 7.54 – 7.43 (m, 7H), 7.40 – 7.36 (m, 2H), 3.50 (d, J = 7.1 Hz, 1H), 2.92 (d, J = 7.1 Hz, 1H). ^{13}C NMR (100 MHz, $CDCl_3$): δ = 136.1, 130.8, 129.8, 129.7, 129.2, 129.2, 128.9, 127.9, 116.6, 115.0, 35.7, 35.5, 28.5, 19.6.
HRMS (ESI): calcd. for $C_{17}H_{12}ClN_2$ $[M + H]^+$ 279.0684, found 279.0687.

1-(4-bromophenyl)-3-phenylcyclopropane-1,2-dicarbonitrile (3p)

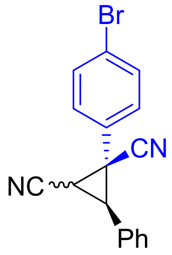

Cis isomer: Yellow solid; mp = 186-188 °C; R_f = 0.45 (petroleum ether / ethyl acetate = 4:1). ^{1}H NMR (400 MHz, $CDCl_3$) δ = 7.58 (dd, J = 14.3, 7.9 Hz, 4H), 7.51 – 7.42 (m, 3H), 7.33 (d, J = 8.6 Hz, 2H), 3.25 (d, J = 9.2 Hz, 1H), 2.68 (d, J = 9.2 Hz, 1H). ^{13}C NMR (100 MHz, $CDCl_3$) δ = 132.8, 132.0, 129.7, 129.3, 129.3, 129.1, 128.3, 123.9, 115.4, 114.3, 35.7, 28.8, 20.6.

Trans isomer: Yellow solid; mp = 129-131 °C; R_f = 0.57 (petroleum ether / ethyl acetate = 4:1). ^{1}H NMR (400 MHz, $CDCl_3$): δ = 7.66 – 7.61 (m, 2H), 7.49 – 7.43 (m, 5H), 7.40 – 7.35 (m, 2H), 3.50 (d, J = 7.1 Hz, 1H), 2.92 (d, J = 7.1 Hz, 1H). ^{13}C NMR (100 MHz, $CDCl_3$): δ = 132.7, 130.7, 130.0, 129.4, 129.2, 129.1, 127.9, 124.3, 116.5, 115.0, 35.6, 28.5, 19.6.
HRMS (ESI): calcd. for $C_{17}H_{12}BrN_2$ $[M + H]^+$ 323.0178, found 323.0173.

1-(2-bromophenyl)-3-phenylcyclopropane-1,2-dicarbonitrile (3q)

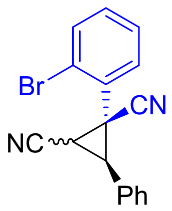

Cis isomer: Brown solid; mp = 128-130 °C; R_f = 0.38 (petroleum ether / ethyl acetate = 4:1). ^{1}H NMR (400 MHz, $CDCl_3$) δ = 7.70 – 7.65 (m, 3H), 7.51 – 7.43 (m, 4H), 7.39 (td, J = 7.5, 1.3 Hz, 1H), 7.32 (td, J = 7.7, 1.8 Hz, 1H), 3.24 (d, J = 9.2 Hz, 1H), 2.63 (d, J = 9.2 Hz, 1H). ^{13}C NMR (100 MHz, $CDCl_3$) δ = 133.8, 132.8, 131.6, 131.2, 130.1, 129.1, 129.1, 129.0, 128.3, 125.4, 114.8, 114.7, 35.6, 30.1, 20.9.

Trans isomer: Yellow solid; mp = 182-184 °C; R_f =0.47 (petroleum ether / ethyl acetate = 4:1). ^{1}H NMR (400 MHz, $CDCl_3$): δ = 7.76 (dd, J = 8.0, 1.2 Hz, 1H), 7.59 (dd, J = 7.7, 1.6 Hz, 1H), 7.50 – 7.44 (m, 6H), 7.40 – 7.35 (m, 1H), 3.43 (d, J = 7.1

Hz, 1H), 3.02 (d, J = 7.1 Hz, 1H). ^{13}C NMR (100 MHz, $CDCl_3$): δ = 134.2, 131.9, 131.8, 130.9, 130.6, 129.2, 129.1, 128.3, 128.0, 125.9, 115.6, 115.3, 37.4, 30.3, 19.6. HRMS (ESI): calcd. for $C_{17}H_{12}BrN_2$ $[M + H]^+$ 323.0178, found 323.0170.

3-phenyl-1-(4-(trifluoromethyl)phenyl)cyclopropane-1,2-dicarbonitrile (3r)

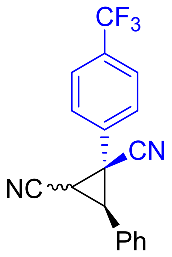

Cis isomer: Yellow solid; mp = 140-142 °C; R_f = 0.43 (petroleum ether / ethyl acetate = 4:1). ^{1}H NMR (400 MHz, $CDCl_3$) δ = 7.73 (d, J = 8.3 Hz, 2H), 7.58 (d, J = 8.5 Hz, 4H), 7.51 – 7.43 (m, 3H), 3.31 (d, J = 9.2 Hz, 1H), 2.77 (d, J = 9.2 Hz, 1H). ^{13}C NMR (100 MHz, $CDCl_3$) δ = 136.7, 131.8 (q, $J_{C,F}$ = 33 Hz), 129.6, 129.4, 129.3, 129.1, 127.0, 126.6 (q, $J_{C,F}$ = 3 Hz), 123.4 (q, $J_{C,F}$ = 271 Hz), 115.2, 114.1, 36.1, 29.0, 20.9.

Trans isomer: Yellow liquid; R_f = 0.60 (petroleum ether / ethyl acetate = 4:1). ^{1}H NMR (400 MHz, $CDCl_3$): δ = 7.79 (d, J = 8.3 Hz, 2H), 7.73 (d, J = 8.3 Hz, 2H), 7.50 – 7.43 (m, 3H), 7.39 (dd, J = 7.3, 1.7 Hz, 2H), 3.58 (d, J = 7.1 Hz, 1H), 2.99 (d, J = 7.1 Hz, 1H). ^{13}C NMR (100 MHz, $CDCl_3$): δ = 134.2, 132.0 (q, $J_{C,F}$ = 33 Hz), 130.6, 129.4, 129.3, 128.9, 128.0, 126.6 (q, $J_{C,F}$ = 4 Hz), 123.4 (q, $J_{C,F}$ = 271 Hz), 116.3, 114.8, 35.9, 28.7, 19.8.
HRMS (ESI): calcd. for $C_{18}H_{12}F_3N_2$ $[M + H]^+$ 313.0947, found 313.0951.

1-(3,4-dichlorophenyl)-3-phenylcyclopropane-1,2-dicarbonitrile (3s)

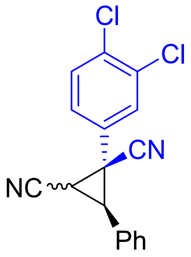

Cis isomer: Yellow solid; mp = 141-143 °C; R_f = 0.53 (petroleum ether / ethyl acetate = 4:1). ^{1}H NMR (400 MHz, $CDCl_3$) δ = 7.57 – 7.51 (m, 4H), 7.50 – 7.42 (m, 3H), 7.29 (dd, J = 8.4, 2.3 Hz, 1H), 3.25 (d, J = 9.3 Hz, 1H), 2.70 (d, J = 9.3 Hz, 1H). ^{13}C NMR (100 MHz, $CDCl_3$) δ = 134.1, 133.9, 132.8, 131.5, 129.5, 129.4, 129.2, 129.1, 128.7, 125.9, 115.1, 114.1, 35.8, 28.4, 20.7.

Trans isomer: White solid; mp = 108-110 °C; R_f = 0.62 (petroleum ether / ethyl acetate = 4:1). ^{1}H NMR (400 MHz, $CDCl_3$): δ = 7.68 (d, J = 2.3 Hz, 1H), 7.58 (d, J = 8.4 Hz, 1H), 7.49 – 7.41 (m, 4H), 7.38 – 7.34 (m, 2H), 3.50 (d, J = 7.1 Hz, 1H), 2.93

(d, J = 7.1 Hz, 1H). ^{13}C NMR (100 MHz, $CDCl_3$): δ = 134.6, 133.8, 131.5, 130.5, 130.4, 130.4, 129.3, 129.2, 127.9, 127.6, 116.1, 114.8, 35.7, 28.1, 19.8.
HRMS (ESI): calcd. for $C_{17}H_{11}Cl_2N_2$ $[M + H]^+$ 313.0294, found 313.0290.

1-(3,5-difluorophenyl)-3-phenylcyclopropane-1,2-dicarbonitrile (3t)

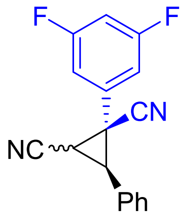

Cis isomer: Yellow solid; mp = 132-134 °C; R_f = 0.57 (petroleum ether / ethyl acetate = 4:1). ^1H NMR (400 MHz, $CDCl_3$) δ = 7.55 (dd, J = 7.4, 1.2 Hz, 2H), 7.51 – 7.44 (m, 3H), 7.03 – 6.96 (m, 2H), 6.90 (tt, J = 8.6, 2.2 Hz, 1H), 3.25 (d, J = 9.3 Hz, 1H), 2.71 (d, J = 9.3 Hz, 1H). ^{13}C NMR (100 MHz, $CDCl_3$) δ = 163.4 (dd, $J_{C,F}$ = 251, 13 Hz), 136.4 (t, $J_{C,F}$ = 10 Hz), 129.4, 129.2, 129.1, 127.9, 114.9, 113.9, 110.0 (dd, $J_{C,F}$ = 19, 8 Hz), 105.3 (t, $J_{C,F}$ = 25 Hz), 36.1, 28.7 (t, $J_{C,F}$ = 3 Hz), 21.0.

Trans isomer: Yellow solid; mp = 145-147 °C; R_f = 0.63 (petroleum ether / ethyl acetate = 4:1). ^1H NMR (400 MHz, $CDCl_3$): δ = 7.50 – 7.43 (m, 3H), 7.38 – 7.34 (m, 2H), 7.18 – 7.11 (m, 2H), 6.95 (tt, J = 8.6, 2.2 Hz, 1H), 3.51 (d, J = 7.2 Hz, 1H), 2.95 (d, J = 7.2 Hz, 1H). ^{13}C NMR (100 MHz, $CDCl_3$): δ = 163.2 (dd, $J_{C,F}$ = 250, 13 Hz), 133.8 (t, $J_{C,F}$ = 10 Hz), 130.4, 129.4, 129.2, 127.9, 116.0, 114.6, 111.9 (dd, $J_{C,F}$ = 19, 8 Hz), 105.8 (t, $J_{C,F}$ = 25 Hz), 35.8, 28.5 (t, $J_{C,F}$ = 3 Hz), 20.0.
HRMS (ESI): calcd. for $C_{17}H_{11}F_2N_2$ $[M + H]^+$ 281.0885, found 281.0889.

1-(4-cyanophenyl)-3-phenylcyclopropane-1,2-dicarbonitrile (3u)

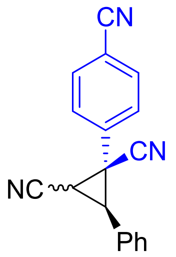

Cis isomer: Yellow solid; mp = 186-188 °C; R_f = 0.40 (petroleum ether / ethyl acetate = 2:1). ^1H NMR (400 MHz, $CDCl_3$) δ = 7.78 (d, J = 8.1 Hz, 2H), 7.60 – 7.53 (m, 4H), 7.52 – 7.45 (m, 3H), 3.31 (d, J = 9.3 Hz, 1H), 2.78 (d, J = 9.3 Hz, 1H). ^{13}C NMR (100 MHz, $CDCl_3$) δ = 137.8, 133.3, 129.6, 129.4, 129.3, 129.1, 127.2, 117.5, 114.8, 113.8, 113.7, 36.4, 29.1, 21.2.

Trans isomer: Yellow solid; mp = 128-130 °C; R_f = 0.43 (petroleum ether / ethyl acetate = 4:1). ^1H NMR (400 MHz, $CDCl_3$): δ = 7.80 – 7.76 (m, 2H), 7.72 – 7.68 (m, 2H), 7.49 – 7.43 (m, 3H), 7.39 – 7.35 (m, 2H), 3.59 (d, J = 7.2 Hz, 1H), 3.03 (d, J = 7.2 Hz, 1H). ^{13}C NMR (100 MHz, $CDCl_3$): δ = 135.2, 133.0, 130.3, 129.3, 129.1,

129.0, 127.9, 117.6, 115.9, 114.6, 113.7, 35.8, 28.7, 20.0.
HRMS (ESI): calcd. for $C_{18}H_{12}N_3$ $[M + H]^+$ 270.1026, found 270.1023.

1-(pyridin-2-yl)-3-(*o*-tolyl)cyclopropane-1,2-dicarbonitrile (4a)

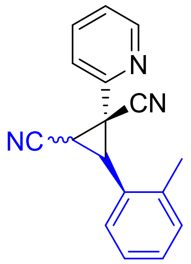

Cis isomer: White solid; mp = 136-138 °C; R_f = 0.41 (petroleum ether / ethyl acetate = 4:1). ^{1}H NMR (400 MHz, $CDCl_3$) δ = 8.54 (ddd, J = 4.8, 1.6, 0.9 Hz, 1H), 7.91 (d, J = 7.9 Hz, 1H), 7.84 (dd, J = 7.7, 1.7 Hz, 1H), 7.67 (d, J = 7.0 Hz, 1H), 7.35 – 7.28 (m, 4H), 3.51 (d, J = 9.0 Hz, 1H), 3.32 (d, J = 9.1 Hz, 1H), 2.27 (s, 3H). ^{13}C NMR (100 MHz, $CDCl_3$) δ = 150.1, 149.9, 138.3, 137.6, 130.8, 129.2, 129.06, 128.4, 126.4, 123.6, 121.5, 115.4, 115.2, 36.8, 30.2, 20.9, 19.7.

Trans isomer: White solid; mp = 129-131 °C; R_f = 0.52 (petroleum ether / ethyl acetate = 4:1). ^{1}H NMR (400 MHz, $CDCl_3$): δ = 8.69 (dt, J = 4.8, 1.4 Hz, 1H), 7.87 – 7.82 (m, 2H), 7.37 (ddd, J = 6.1, 4.8, 2.7 Hz, 1H), 7.35 – 7.26 (m, 3H), 7.22 (dd, J = 6.0, 2.7 Hz, 1H), 4.35 (d, J = 7.8 Hz, 1H), 3.03 (d, J = 7.8 Hz, 1H), 2.27 (s, 3H). ^{13}C NMR (100 MHz, $CDCl_3$): δ = 149.6, 148.4, 138.2, 137.5, 130.6, 130.4, 129.0, 127.1, 126.4, 123.8, 122.7, 116.5, 114.7, 34.8, 29.6, 21.2, 19.5.
HRMS (ESI): calcd. for $C_{17}H_{14}N_3$ $[M + H]^+$ 260.1182, found 260.1183.

1-(pyridin-3-yl)-3-(*m*-tolyl)cyclopropane-1,2-dicarbonitrile (4b)

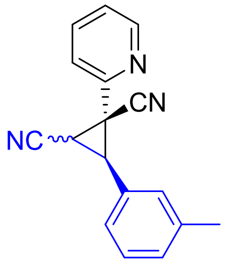

Cis isomer: Yellow solid; mp = 122-124 °C; R_f = 0.43 (petroleum ether / ethyl acetate = 4:1). ^{1}H NMR (400 MHz, $CDCl_3$) δ = 8.53 (d, J = 4.7 Hz, 1H), 7.89 (d, J = 7.9 Hz, 1H), 7.80 (td, J = 7.7, 1.7 Hz, 1H), 7.38 – 7.29 (m, 4H), 7.22 (d, J = 7.3 Hz, 1H), 3.58 (d, J = 9.2 Hz, 1H), 3.28 (d, J = 9.2 Hz, 1H), 2.40 (s, 3H). ^{13}C NMR (100 MHz, CDCl3) δ = 150.2, 149.9, 138.7, 137.4, 130.3, 129.8, 129.7, 128.9, 126.0, 123.5, 121.6, 115.4, 114.8, 37.6, 29.9, 21.3, 21.1.

Trans isomer: White solid; mp = 130-132 °C; R_f = 0.53 (petroleum ether / ethyl acetate = 4:1). ^{1}H NMR (400 MHz, $CDCl_3$): δ = 8.68 (d, J = 4.7 Hz, 1H), 7.86 – 7.80 (m, 2H), 7.37 – 7.30 (m, 2H), 7.22 – 7.13 (m, 3H), 4.34 (d, J = 7.7 Hz, 1H), 2.99 (d, J

= 7.7 Hz, 1H), 2.39 (s, 3H). ^{13}C NMR (100 MHz, CDCl$_3$): δ = 149.4, 148.5, 138.8, 137.3, 131.3, 129.7, 128.9, 128.8, 124.9, 123.8, 122.8, 116.5, 114.6, 35.6, 30.1, 21.3, 21.2.

HRMS (ESI): calcd. for $C_{17}H_{14}N_3$ [M + H]$^+$ 260.1182, found 260.1182.

3-(4-(*tert*-butyl)phenyl)-1-(pyridin-2-yl)cyclopropane-1,2-dicarbonitrile (4c)

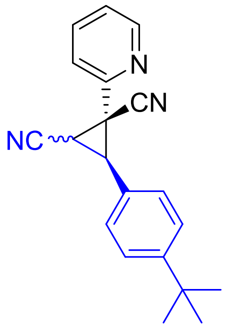

Cis isomer: White solid; mp = 141-143 °C; R_f = 0.53 (petroleum ether / ethyl acetate = 4:1).^{1}H NMR (400 MHz, CDCl$_3$) δ = 8.53 (d, J = 4.3 Hz, 1H), 7.89 (d, J = 7.9 Hz, 1H), 7.80 (td, J = 7.7, 1.7 Hz, 1H), 7.52 – 7.46 (m, 4H), 7.31 (ddd, J = 7.4, 4.8, 0.9 Hz, 1H), 3.56 (d, J = 9.2 Hz, 1H), 3.30 (d, J = 9.2 Hz, 1H), 1.34 (s, 9H). ^{13}C NMR (100 MHz, CDCl$_3$) δ = 151.9, 150.2, 149.7, 137.4, 128.7, 127.3, 125.9, 123.5, 121.5, 115.4, 114.9, 37.5, 34.6, 31.2, 29.9, 21.2.

Trans isomer: White solid; mp = 108-110 °C; R_f = 0.65 (petroleum ether / ethyl acetate = 4:1). ^{1}H NMR (400 MHz, CDCl$_3$): δ = 8.69 – 8.67 (m, 1H), 7.86 – 7.82 (m, 2H), 7.46 – 7.44 (m, 2H), 7.36 (ddd, J = 6.8, 4.8, 1.9 Hz, 1H), 7.29 (d, J = 8.3 Hz, 2H), 4.32 (d, J = 7.7 Hz, 1H), 2.98 (d, J = 7.7 Hz, 1H), 1.33 (s, 9H). ^{13}C NMR (100 MHz, CDCl$_3$): δ = 152.0, 149.4, 148.5, 137.4, 128.3, 127.6, 126.0, 123.8, 122.7, 116.6, 114.7, 35.5, 34.6, 31.2, 30.1, 21.4.

HRMS (ESI): calcd. for $C_{20}H_{20}N_3$ [M + H]$^+$ 302.1652, found 302.1650.

3-(3,5-dimethylphenyl)-1-(pyridin-2-yl)cyclopropane-1,2-dicarbonitrile (4d)

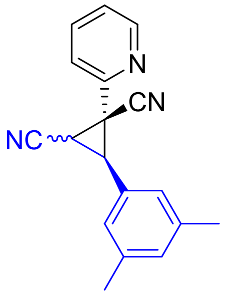

Only the *cis* isomer can be separated as pure compound, the spectroscopic data are as follows:

Cis isomer: White solid; mp = 139-141 °C; R_f = 0.50 (petroleum ether / ethyl acetate = 4:1). ^{1}H NMR (400 MHz, CDCl$_3$) δ = 8.54 – 8.51 (m, 1H), 7.89 (d, J = 7.9 Hz, 1H), 7.80 (td, J = 7.7, 1.7 Hz, 1H), 7.31 (ddd, J = 7.5, 4.8, 1.0 Hz, 1H), 7.16 (s, 2H), 7.04 (s, 1H), 3.53 (d, J = 9.2 Hz, 1H), 3.26 (d, J = 9.2 Hz, 1H), 2.36 (s, 6H). ^{13}C NMR

(100 MHz, $CDCl_3$) δ = 150.4, 149.9, 138.6, 137.4, 130.7, 130.2, 126.2, 123.5, 121.6, 115.4, 114.9, 37.7, 29.9, 21.3, 21.1.
HRMS (ESI): calcd. for $C_{18}H_{16}N_3$ $[M + H]^+$ 274.1339, found 274.1341.

3-(4-methoxyphenyl)-1-(pyridin-2-yl)cyclopropane-1,2-dicarbonitrile (4e)

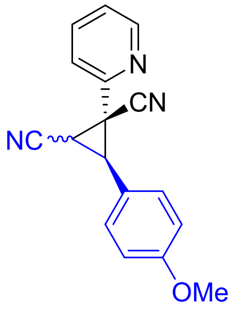

Only the *trans* isomer can be separated as pure compound, the spectroscopic data are as follows:
***Trans* isomer:** Yellow solid; mp = 149-151 °C; R_f = 0.40 (petroleum ether / ethyl acetate = 4:1). ^{1}H NMR (400 MHz, $CDCl_3$) δ = 8.67 (dt, J = 4.8, 1.4 Hz, 1H), 7.84 – 7.79 (m, 2H), 7.37 – 7.32 (m, 1H), 7.27 (d, J = 8.9 Hz, 2H), 6.97 – 6.92 (m, 2H), 4.30 (d, J = 7.6 Hz, 1H), 3.82 (s, 3H), 2.93 (d, J = 7.6 Hz, 1H). ^{13}C NMR (100 MHz, $CDCl_3$) δ =160.0, 149.5, 148.6, 137.4, 129.2, 123.8, 123.3, 122.8, 116.7, 114.8, 114.5, 55.3, 35.3, 30.2, 21.4.
HRMS (ESI): calcd. for $C_{17}H_{14}N_3O$ [M + H]+ 276.1131, found 276.11310.

3-(naphthalen-1-yl)-1-(pyridin-3-yl)cyclopropane-1,2-dicarbonitrile (4f)

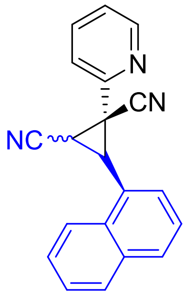

***Cis* isomer:** Yellow solid; mp = 170-172 °C; R_f = 0.38 (petroleum ether / ethyl acetate = 4:1). ^{1}H NMR (400 MHz, $CDCl_3$) δ = 8.65 – 8.55 (m, 1H), 7.92 (tdd, J = 13.7, 10.6, 7.0 Hz, 5H), 7.67 (d, J = 8.3 Hz, 1H), 7.58 – 7.46 (m, 3H), 7.40 – 7.36 (m, 1H), 3.93 (d, J = 9.0 Hz, 1H), 3.47 (d, J = 9.0 Hz, 1H). ^{13}C NMR (100 MHz, $CDCl_3$) δ = 150.1, 150.1, 137.7, 133.8, 132.2, 129.9, 129.2, 127.0, 126.9, 126.7, 126.3, 125.3, 123.7, 122.8, 121.9, 115.3, 115.2, 35.8, 30.6, 21.0.

***Trans* isomer:** White solid; mp = 200-202 °C; R_f = 0.53 (petroleum ether / ethyl acetate = 4:1). ^{1}H NMR (400 MHz, $CDCl_3$): δ = 8.78 (dt, J = 4.8, 1.3 Hz, 1H), 7.89 (ddd, J = 20.8, 16.5, 8.1 Hz, 5H), 7.57 – 7.42 (m, 5H), 4.84 (d, J = 7.7 Hz, 1H), 3.17 (d, J = 7.7 Hz, 1H). ^{13}C NMR (100 MHz, $CDCl_3$): δ = 149.8, 148.4, 137.7, 133.7, 132.2, 129.9, 129.0, 128.3, 127.3, 126.6, 125.4, 125.1, 124.1, 123.3, 123.0, 116.5,

114.8, 33.9, 30.2, 21.2.
HRMS (ESI): calcd. for $C_{20}H_{14}N_3$ $[M + H]^+$ 296.1182, found 296.1187.

3-(3-fluorophenyl)-1-(pyridin-2-yl)cyclopropane-1,2-dicarbonitrile (4g)

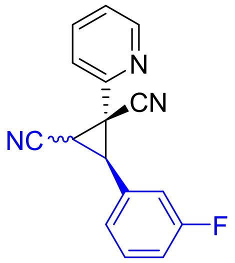

Cis isomer: Yellow solid; mp = 97-99 °C; R_f = 0.37 (petroleum ether / ethyl acetate = 4:1). ^{1}H NMR (400 MHz, $CDCl_3$) δ = 8.53 (ddd, *J* = 4.8, 1.6, 1.0 Hz, 1H), 7.88 (dt, *J* = 7.9, 0.9 Hz, 1H), 7.82 (td, *J* = 7.7, 1.7 Hz, 1H), 7.44 (td, *J* = 8.0, 5.8 Hz, 1H), 7.39 – 7.26 (m, 3H), 7.11 (td, *J* = 8.3, 2.1 Hz, 1H), 3.60 (d, *J* = 9.3 Hz, 1H), 3.29 (d, *J* = 9.3 Hz, 1H). ^{13}C NMR (100 MHz, $CDCl_3$): δ = 162.3 (d, $J_{C,F}$ = 246 Hz), 150.0, 149.7, 137.5, 132.7 (d, $J_{C,F}$ = 8 Hz), 130.7 (d, $J_{C,F}$ = 8 Hz), 124.8 (d, $J_{C,F}$ = 3 Hz), 123.7, 121.6, 116.4 (d, $J_{C,F}$ = 23 Hz), 116.1 (d, $J_{C,F}$ = 21 Hz), 115.1, 114.5, 36.8 (d, $J_{C,F}$ = 2 Hz), 29.9, 21.2.

Trans isomer: White solid; mp = 100-102 °C; R_f = 0.50 (petroleum ether / ethyl acetate = 4:1). ^{1}H NMR (400 MHz, $CDCl_3$): δ = 8.67 (dt, *J* = 4.8, 1.4 Hz, 1H), 7.87 – 7.83 (m, 2H), 7.44 – 7.35 (m, 2H), 7.16 – 7.07 (m, 3H), 4.38 (d, *J* = 7.6 Hz, 1H), 2.96 (d, *J* = 7.6 Hz, 1H). ^{13}C NMR (100 MHz, $CDCl_3$): δ = 162.9 (d, $J_{C,F}$ = 246 Hz), 149.5, 148.1, 137.5, 133.8 (d, $J_{C,F}$ = 8 Hz), 130.8 (d, $J_{C,F}$ = 8 Hz), 124.0, 123.7 (d, $J_{C,F}$ = 3 Hz), 122.9, 116.2, 116.2 (d, $J_{C,F}$ = 21 Hz), 115.4 (d, $J_{C,F}$ = 23 Hz), 114.2, 35.0 (d, $J_{C,F}$ = 2 Hz), 30.2, 21.4.
HRMS (ESI): calcd. for $C_{16}H_{11}FN_3$ [M + H]+ 264.0932, found 264.0930.

3-(4-chlorophenyl)-1-(pyridin-2-yl)cyclopropane-1,2-dicarbonitrile (4h)

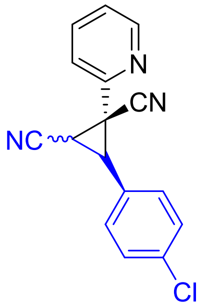

Only the *cis* isomer can be separated as pure compound, the spectroscopic data are as follows:
Cis isomer: Yellow solid; mp = 127-129 °C; R_f = 0.38 (petroleum ether / ethyl acetate = 4:1). ^{1}H NMR (400 MHz, $CDCl_3$) δ = 8.55 – 8.51 (m, 1H), 7.88 (d, *J* = 7.9 Hz, 1H), 7.82 (td, *J* = 7.7, 1.7 Hz, 1H), 7.50 (d, *J* = 8.5 Hz, 2H), 7.45 – 7.41 (m, 2H), 7.33 (ddd, *J* = 7.4, 4.8, 1.1 Hz, 1H), 3.58 (d, *J* = 9.2 Hz, 1H), 3.29 (d, *J* = 9.2 Hz, 1H). ^{13}C

NMR (100 MHz, $CDCl_3$): δ = 150.0, 149.7, 137.5, 135.1, 130.5, 129.3, 128.9, 123.7, 121.6, 115.1, 114.6, 36.8, 30.0, 21.3.
HRMS (ESI): calcd. for $C_{16}H_{11}ClN_3$ $[M + H]^+$ 280.0636, found 280.0640.

1-phenyl-3-(*o*-tolyl)cyclopropane-1,2-dicarbonitrile (4i)

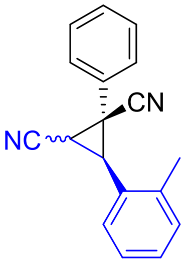

Cis isomer: Black liquid; R_f = 0.39 (petroleum ether / ethyl acetate = 4:1). ^1H NMR (400 MHz, $CDCl_3$) δ = 7.67 (d, J = 6.9 Hz, 1H), 7.49 – 7.43 (m, 5H), 7.35 – 7.29 (m, 3H), 3.20 (d, J = 9.0 Hz, 1H), 2.72 (d, J = 9.0 Hz, 1H), 2.38 (s, 3H). ^{13}C NMR (100 MHz, $CDCl_3$): δ = 138.3, 131.0, 129.7, 129.4, 129.3, 128.8, 126.5, 125.9, 115.8, 35.1, 29.5, 21.0, 20.0.

Trans isomer: Yellow solid; mp = 154-156 °C; R_f = 0.62 (petroleum ether / ethyl acetate = 4:1). ^1H NMR (400 MHz, $CDCl_3$): δ = 7.62 (d, J = 6.8 Hz, 2H), 7.52 (d, J = 7.8 Hz, 3H), 7.37 – 7.25 (m, 4H), 3.56 (d, J = 6.9 Hz, 1H), 2.93 (d, J = 6.9 Hz, 1H), 2.44 (s, 3H). ^{13}C NMR (100 MHz, $CDCl_3$): δ = 138.0, 130.9, 130.1, 130.1, 129.9, 129.6, 129.3, 127.7, 127.6, 126.6, 117.1, 115.2, 34.2, 28.9, 20.5, 20.0.
HRMS (ESI): calcd. for $C_{18}H_{15}N_2$ $[M + H]^+$ 259.1230, found 259.1235.

3-(4-(*tert*-butyl)phenyl)-1-phenylcyclopropane-1,2-dicarbonitrile (4j)

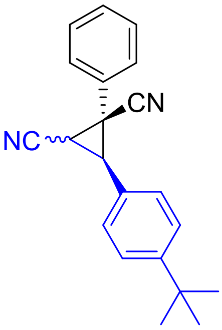

Cis isomer: Yellow liquid; R_f = 0.52 (petroleum ether / ethyl acetate = 4:1). ^1H NMR (400 MHz, $CDCl_3$) δ = 7.51 (d, J = 3.9 Hz, 3H), 7.49 – 7.40 (m, 6H), 3.23 (d, J = 9.2 Hz, 1H), 2.69 (d, J = 9.2 Hz, 1H), 1.35 (s, 9H). ^{13}C NMR (100 MHz, $CDCl_3$): δ = 152.3, 133.1, 129.6, 129.5, 128.8, 127.0, 126.7, 126.1, 115.9, 114.7, 35.5, 34.7, 31.2, 29.3, 20.5.

Trans isomer: Yellow liquid; R_f = 0.70 (petroleum ether / ethyl acetate = 4:1). ^1H NMR (400 MHz, $CDCl_3$): δ = 7.61 – 7.57 (m, 2H), 7.53 – 7.46 (m, 5H), 7.34 (dd, J = 7.6, 5.5 Hz, 3H), 3.50 (d, J = 7.0 Hz, 1H), 2.88 (d, J = 7.0 Hz, 1H), 1.35 (s, 9H). ^{13}C NMR (100 MHz, $CDCl_3$): δ = 152.3, 130.6, 129.9, 129.5, 128.5, 128.1, 127.7, 126.1,

117.1, 115.3, 35.5, 34.7, 31.2, 29.1, 19.8.
HRMS (ESI): calcd. for $C_{21}H_{21}N_2$ $[M + H]^+$ 301.1699, found 301.1694.

3-(3-fluorophenyl)-1-phenylcyclopropane-1,2-dicarbonitrile (4k)

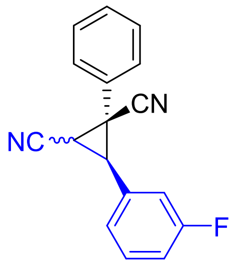

Cis isomer: Yellow liquid; R_f = 0.33 (petroleum ether / ethyl acetate = 4:1); ^{1}H NMR (400 MHz, $CDCl_3$) δ = 7.50 – 7.42 (m, 6H), 7.41 – 7.38 (m, 1H), 7.30 (d, J = 9.4 Hz, 1H), 7.14 (td, J = 8.2, 2.5 Hz, 1H), 3.25 (d, J = 9.2 Hz, 1H), 2.72 (d, J = 9.2 Hz, 1H). ^{13}C NMR (100 MHz, $CDCl_3$): δ = 162.8 (d, $J_{C,F}$ = 247 Hz), 132.6, 132.3 (d, $J_{C,F}$ = 8 Hz), 130.9 (d, $J_{C,F}$ = 8 Hz), 129.7, 129.7, 126.6, 124.9 (d, $J_{C,F}$ = 3 Hz), 116.5 (d, $J_{C,F}$ = 23 Hz), 116.4 (d, $J_{C,F}$ = 21 Hz), 115.6, 114.3, 34.9 (d, $J_{C,F}$ = 2 Hz), 29.3, 20.4.

Trans isomer: Yellow liquid; R_f = 0.56 (petroleum ether / ethyl acetate = 4:1). ^{1}H NMR (400 MHz, $CDCl_3$): δ = 7.57 (dt, J = 8.6, 2.4 Hz, 2H), 7.54 – 7.48 (m, 3H), 7.44 (td, J = 8.2, 7.0 Hz, 1H), 7.20 – 7.17 (m, 1H), 7.16 – 7.10 (m, 2H), 3.53 (d, J = 7.0 Hz, 1H), 2.90 (d, J = 7.0 Hz, 1H). ^{13}C NMR (100 MHz, $CDCl_3$): δ = 162.8 (d, $J_{C,F}$ = 246 Hz), 133.6 (d, $J_{C,F}$ = 8 Hz), 130.9 (d, $J_{C,F}$ = 8 Hz), 130.1, 130.0, 129.6, 128.4, 123.7 (d, $J_{C,F}$ = 3 Hz), 116.7, 116.3 (d, $J_{C,F}$ = 21 Hz), 115.4 (d, $J_{C,F}$ = 22 Hz), 114.9, 35.0 (d, $J_{C,F}$ = 2 Hz), 29.2, 19.7.
HRMS (ESI): calcd. for $C_{17}H_{12}FN_2$ $[M + H]^+$ 263.0979, found 263.0976.

3-(3-fluorophenyl)-1-(*p*-tolyl)cyclopropane-1,2-dicarbonitrile (4l)

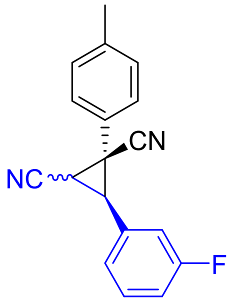

Cis isomer: Yellow solid; mp = 110-112 °C; R_f = 0.47 (petroleum ether / ethyl acetate = 4:1). ^{1}H NMR (400 MHz, $CDCl_3$) δ = 7.46 (dd, J = 8.0, 2.2 Hz, 1H), 7.40 – 7.37 (m, 1H), 7.33 – 7.26 (m, 5H), 7.14 (dd, J = 8.4, 6.0 Hz, 1H), 3.21 (d, J = 9.1 Hz, 1H), 2.67 (d, J = 9.1 Hz, 1H), 2.39 (s, 3H). ^{13}C NMR (100 MHz, $CDCl_3$): δ = 162.8 (d, $J_{C,F}$ = 246 Hz), 139.9, 132.4 (d, $J_{C,F}$ = 8 Hz), 130.9 (d, $J_{C,F}$ = 8 Hz), 130.3, 129.6, 126.5, 124.8 (d, $J_{C,F}$ = 3 Hz), 116.5 (d, $J_{C,F}$ = 23 Hz), 116.3 (d, $J_{C,F}$ = 21 Hz), 115.7, 114.4, 34.8 (d, $J_{C,F}$ = 3 Hz), 29.11, 21.1, 20.3.
Trans isomer: Yellow liquid; R_f = 0.67 (petroleum ether / ethyl acetate = 4:1). ^{1}H

NMR (400 MHz, $CDCl_3$): δ = 7.47 – 7.41 (m, 3H), 7.31 (d, J = 7.9 Hz, 2H), 7.18 (dd, J = 4.5, 3.8 Hz, 1H), 7.12 (t, J = 8.2 Hz, 2H), 3.50 (d, J = 7.0 Hz, 1H), 2.87 (d, J = 7.0 Hz, 1H), 2.41 (s, 3H). ^{13}C NMR (100 MHz, $CDCl_3$): δ = 162.8 (d, $J_{C,F}$ = 247 Hz), 140.3, 133.7 (d, $J_{C,F}$ = 8 Hz), 130.8 (d, $J_{C,F}$ = 8 Hz), 130.2, 128.3, 127.1, 123.7, (d, $J_{C,F}$ = 3 Hz), 116.9, 116.2 (d, $J_{C,F}$ = 21 Hz), 115.4 (d, $J_{C,F}$ = 23 Hz), 115.0, 35.0 (d, $J_{C,F}$ = 3 Hz), 29.0, 21.2, 19.7.
HRMS (ESI): calcd. for $C_{18}H_{14}FN_2$ $[M + H]^+$ 277.1136, found 277.1132.

1-(4-chlorophenyl)-3-(*o*-tolyl)cyclopropane-1,2-dicarbonitrile (4m)

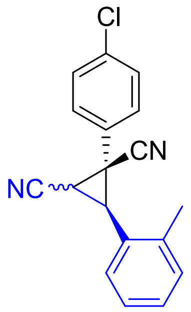

Cis isomer: Brown solid; mp = 132-134 °C; R_f = 0.50 (petroleum ether / ethyl acetate = 4:1). ^1H NMR (400 MHz, $CDCl_3$) δ = 7.64 (d, J = 6.9 Hz, 1H), 7.46 – 7.43 (m, 2H), 7.41 – 7.38 (m, 2H), 7.32 (td, J = 7.5, 3.7 Hz, 3H), 3.16 (d, J = 9.1 Hz, 1H), 2.70 (d, J = 9.1 Hz, 1H), 2.35 (s, 3H). ^{13}C NMR (100 MHz, $CDCl_3$): δ = 138.2, 135.6, 131.2, 131.0, 129.8, 129.4, 128.7, 128.5, 127.2, 126.6, 115.6, 114.6, 35.3, 29.0, 21.2, 19.9.

Trans isomer: Brown solid; mp = 110-112 °C; R_f = 0.64 (petroleum ether / ethyl acetate = 4:1). ^1H NMR (400 MHz, $CDCl_3$): δ = 7.57 – 7.53 (m, 2H), 7.52 – 7.47 (m, 2H), 7.37 – 7.27 (m, 3H), 7.23 (d, J = 7.3 Hz, 1H), 3.50 (d, J = 7.1 Hz, 1H), 2.94 (d, J = 7.1 Hz, 1H), 2.41 (s, 3H). ^{13}C NMR (100 MHz, $CDCl_3$): δ = 137.9, 136.1, 130.9, 129.8, 129.8, 129.4, 129.0, 128.7, 127.5, 126.7, 116.6, 115.0, 34.5, 28.3, 20.57, 20.0.
HRMS (ESI): calcd. for $C_{18}H_{14}ClN_2$ $[M + H]^+$ 293.0840, found 293.0846.

6-phenyl-1-(pyridin-2-yl)-3-azabicyclo[3.1.0]hexane-2,4-dione (5a)

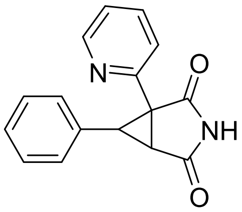

To a solution of KOH (33.6 mg, 0.6 mmol) in 80% EtOH (2 mL) was slowly added the **3a** (49 mg, 0.2 mmol). The mixture was heated to reflux for overnight. Solvent was removed under reduced pressure. The residue was dissolved in water and acidified with acetic acid followed by extraction with ethyl acetate. The crude product was purified by column chromatography on silica gel with Ethyl acetate/Hexane to afford the product as a yellow solid, 99% yield.

Yellow solid; mp = 186-188 °C; R_f = 0.33 (petroleum ether / ethyl acetate = 3:1). ^{1}H NMR (400 MHz, $CDCl_3$) δ = 8.49 (ddd, J = 4.8, 1.6, 0.9 Hz, 1H), 8.15 – 8.09 (m, 1H), 7.74 (td, J = 7.8, 1.8 Hz, 1H), 7.57 (s, 1H), 7.35 (dd, J = 7.0, 1.6 Hz, 2H), 7.32 – 7.26 (m, 3H), 7.23 (ddd, J = 7.5, 4.9, 1.1 Hz, 1H), 3.96 (d, J = 8.7 Hz, 1H), 3.37 (d, J = 8.7 Hz, 1H). ^{13}C NMR (100 MHz, $CDCl_3$): δ = 172.6, 172.1, 152.2, 149.5, 136.6, 132.5, 129.2, 128.9, 128.2, 122.8, 122.6, 44.2, 42.4, 35.9. HRMS (ESI): calcd. for $C_{16}H_{13}N_2O_2$ $[M + H]^+$ 265.0972, found 265.0968.

E. Reference

1. Goumans, T. P. M.; Alem, K.; Lodder, G. Photochemical generation and structure of vinyl radicals. *Eur. J. Org. Chem*. 2008, 435-443.

F. Copies of ^{1}H and ^{13}C NMR spectra

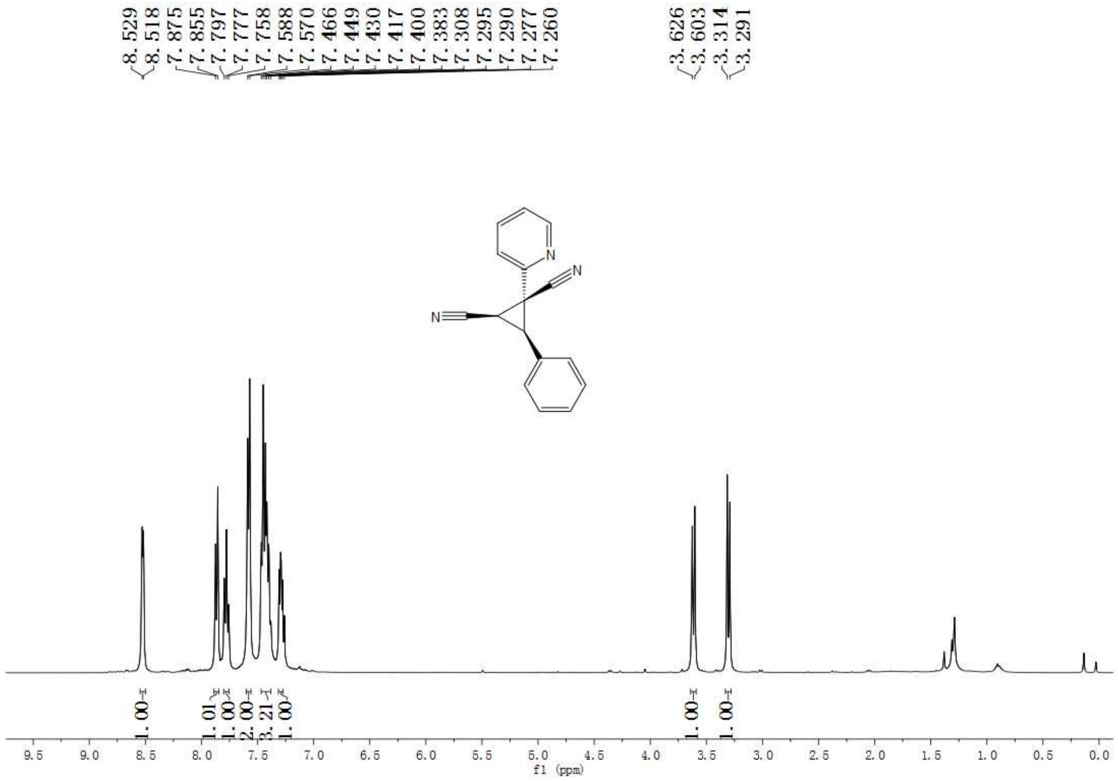

Figure S1. ^{1}H NMR Spectrum of *cis*-3a (400 MHz, $CDCl_3$)

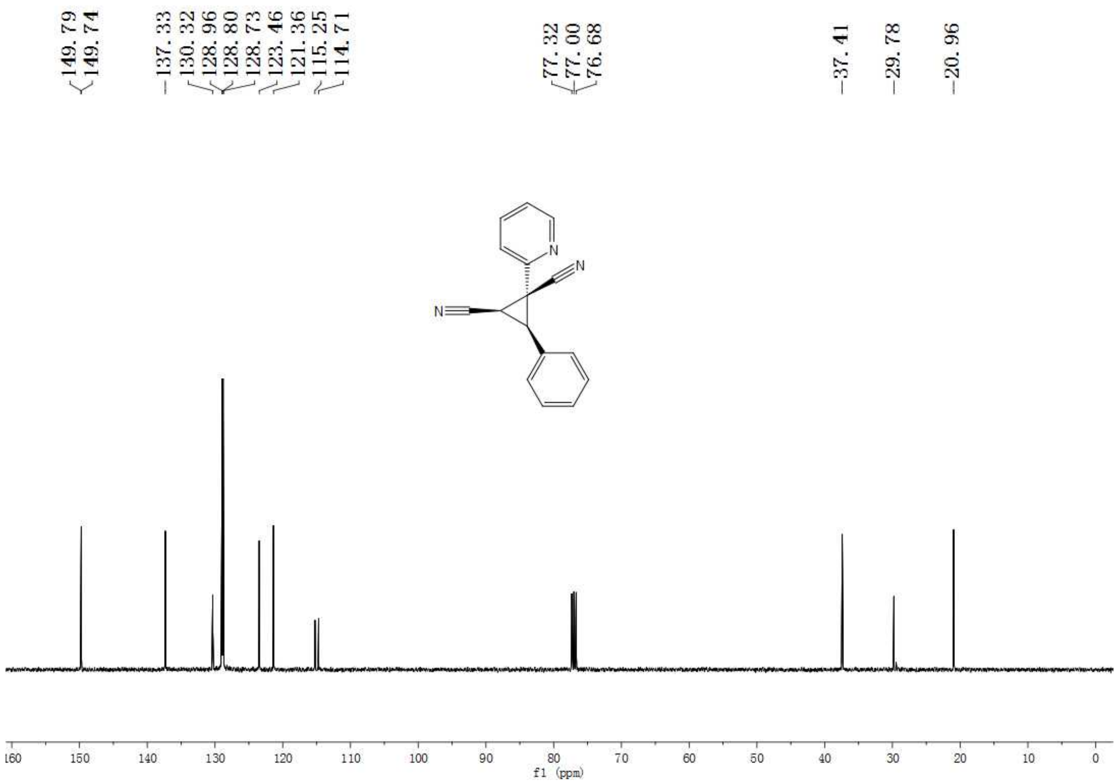

Figure S2. ^{13}C NMR Spectrum of *cis*-3a (100 MHz, $CDCl_3$)

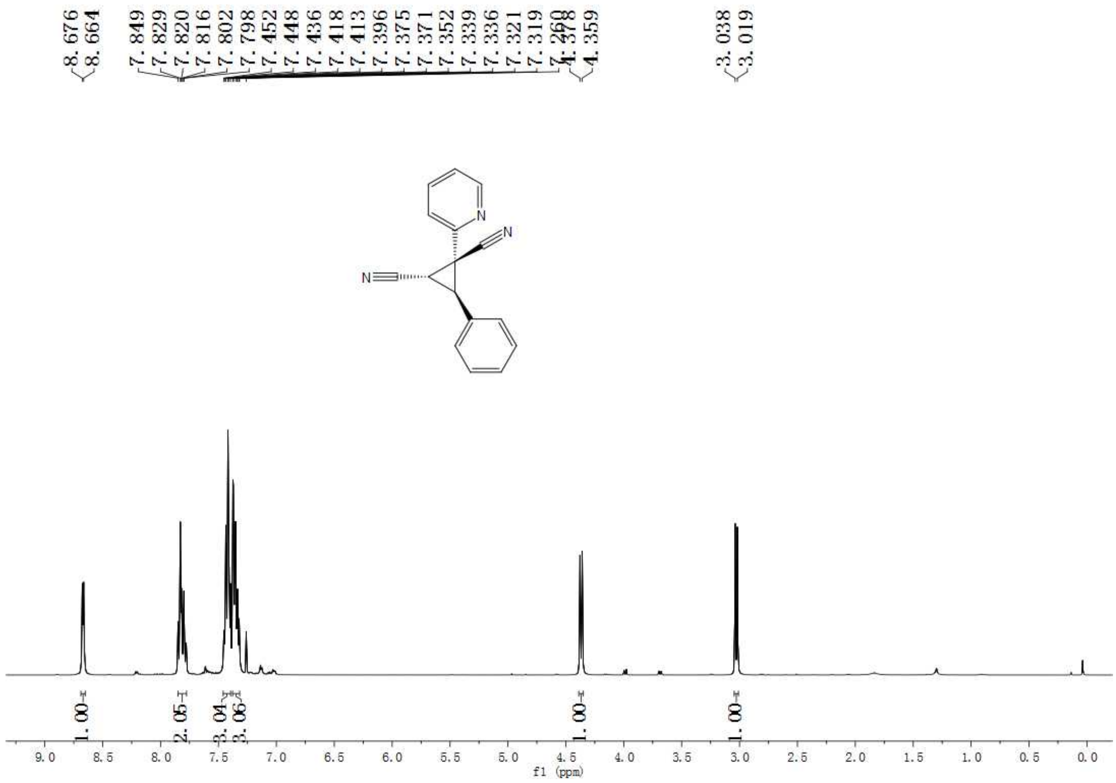

Figure S3. ^{1}H NMR Spectrum of *trans*-3a (400 MHz, $CDCl_3$)

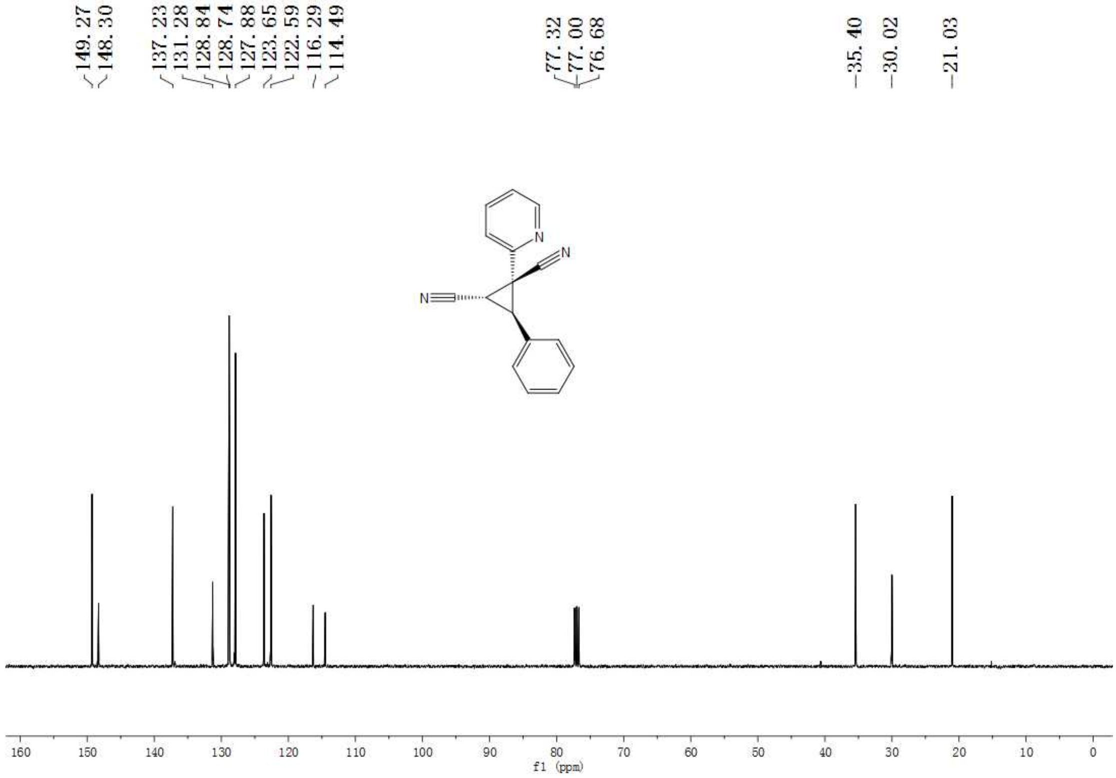

Figure S4. ^{13}C NMR Spectrum of *trans*-3a (100 MHz, $CDCl_3$)

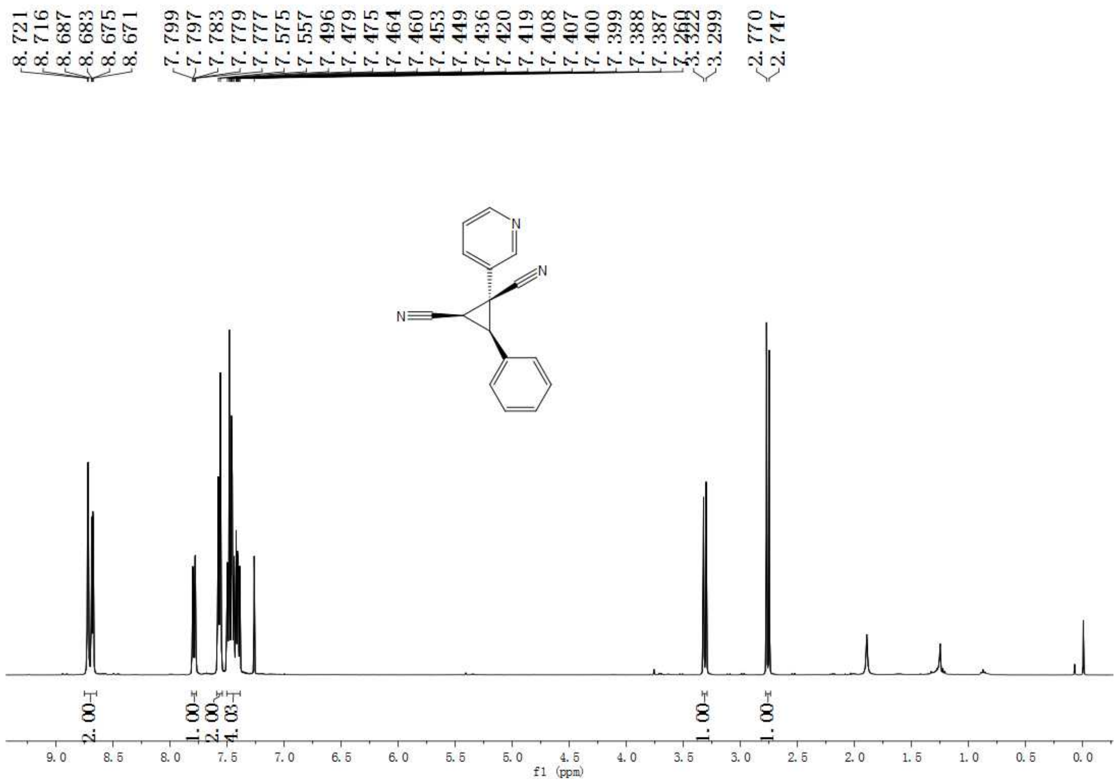

Figure S5. ^{1}H NMR Spectrum of *cis*-3b (400 MHz, $CDCl_3$)

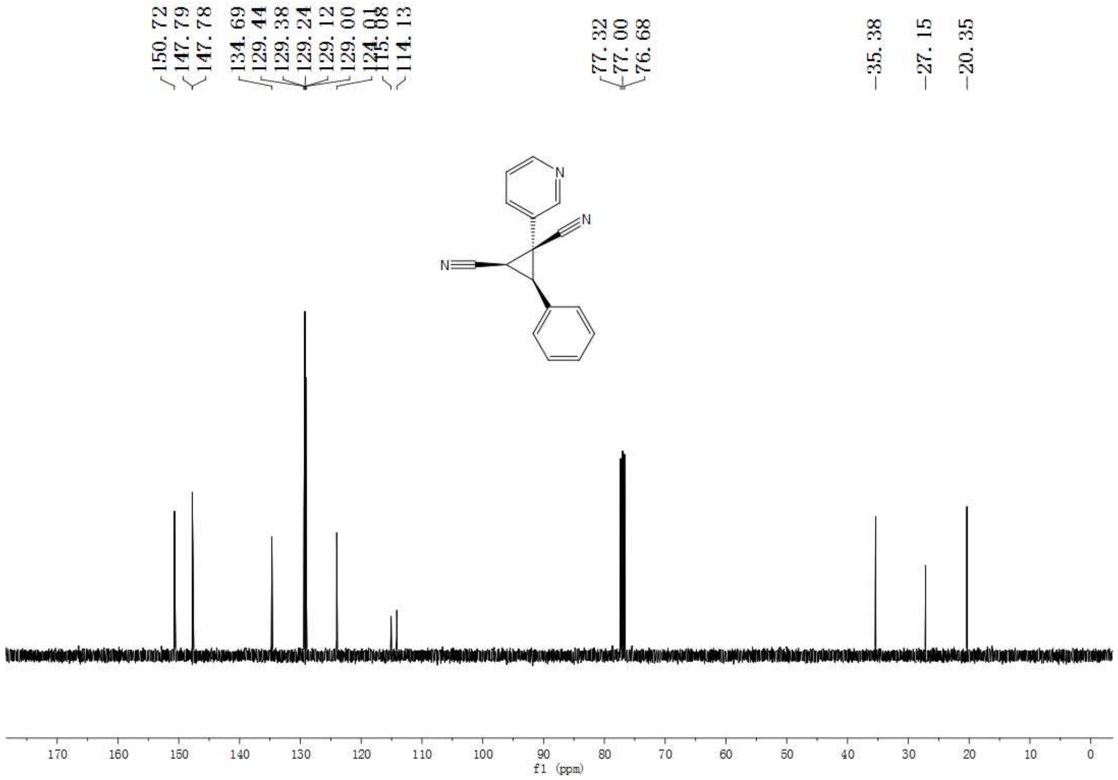

Figure S6. ^{13}C NMR Spectrum of *cis*-3b (100 MHz, $CDCl_3$)

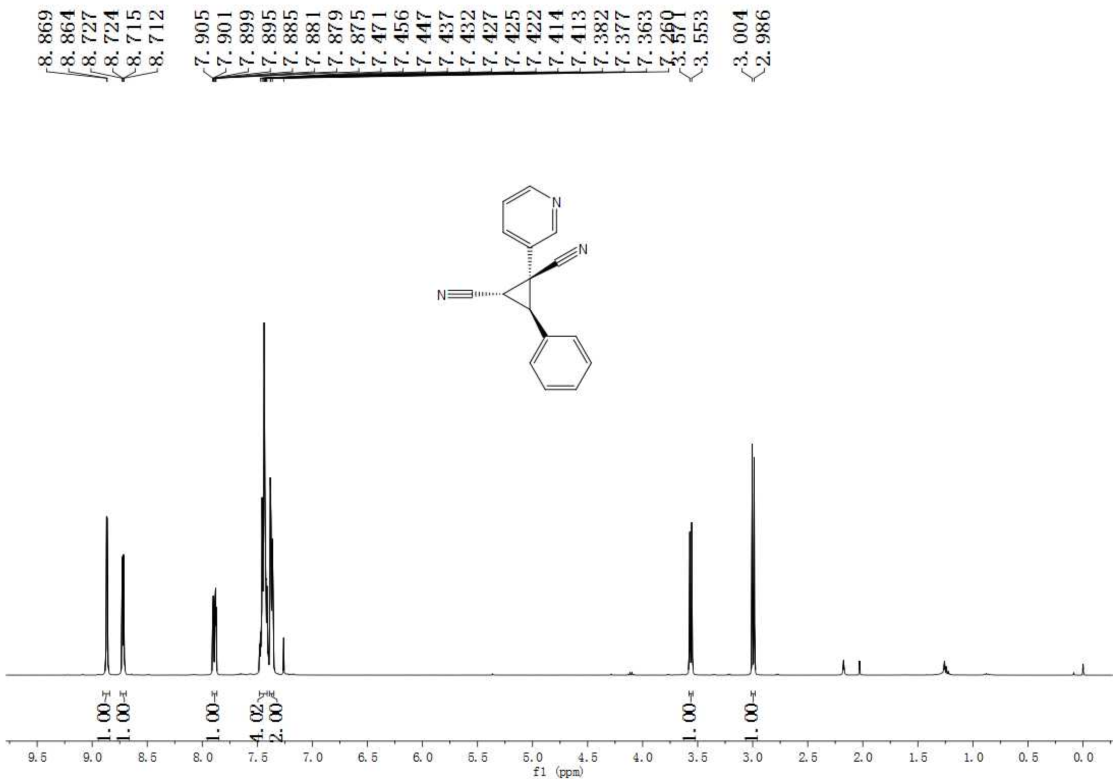

Figure S7. ^{1}H **NMR Spectrum of** ***trans*****-3b (400 MHz, CDCl$_3$)**

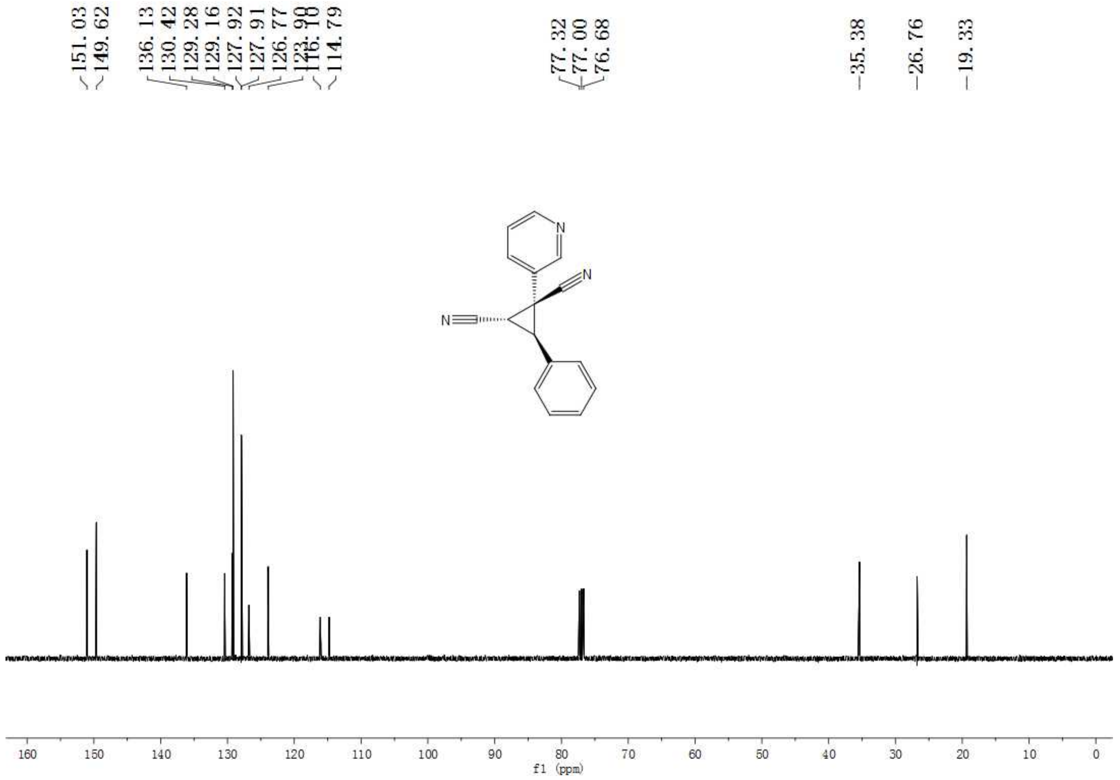

Figure S8. ^{13}C **NMR Spectrum of** ***trans*****-3b (100 MHz, CDCl$_3$)**

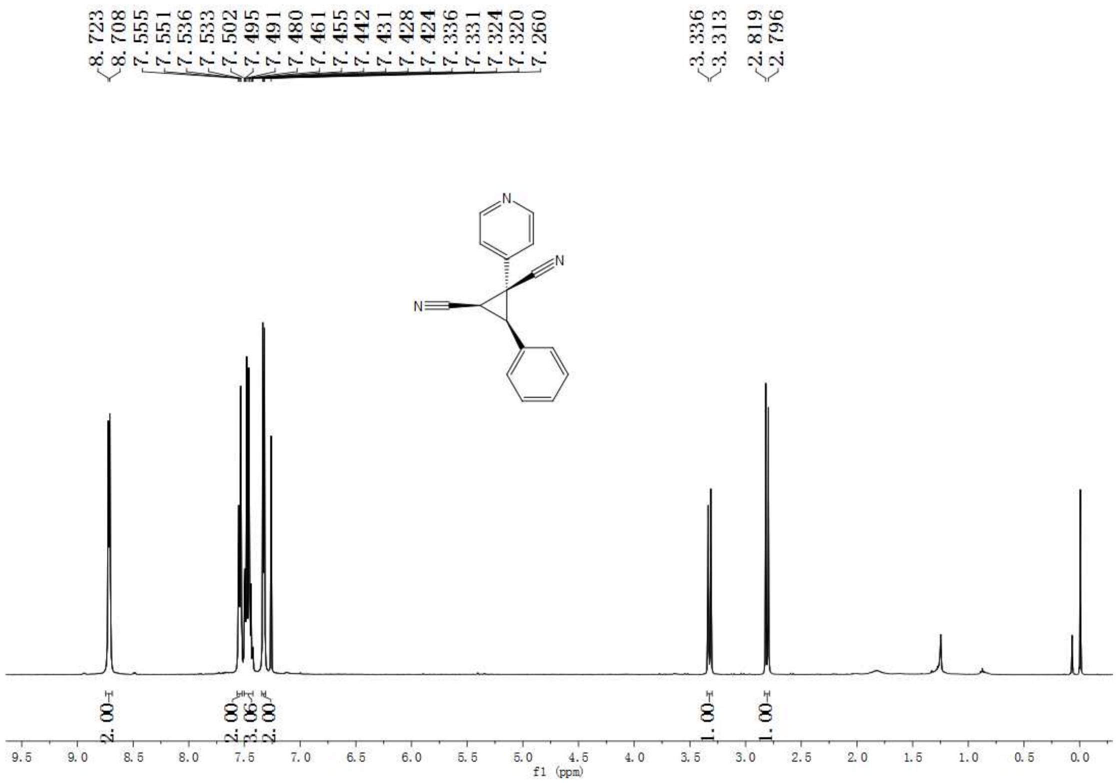

Figure S9. ¹H NMR Spectrum of *cis*-3c (400 MHz, $CDCl_3$)

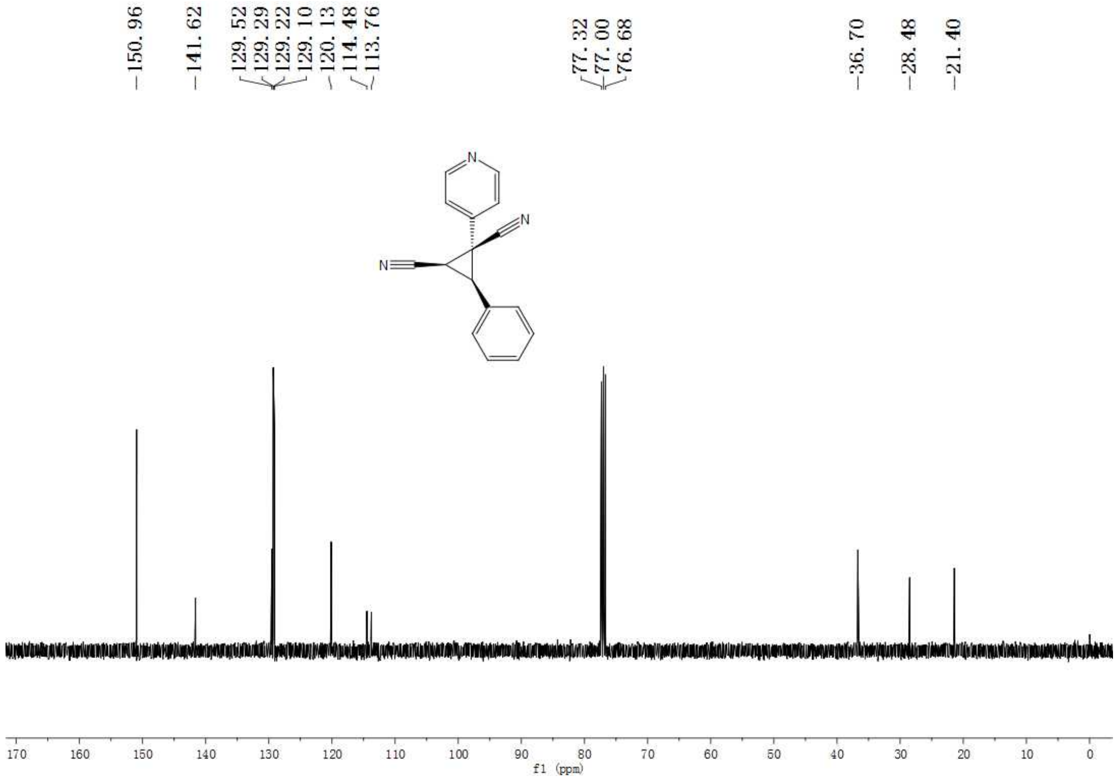

Figure S10. ¹³C NMR Spectrum of *cis*-3c (100 MHz, $CDCl_3$)

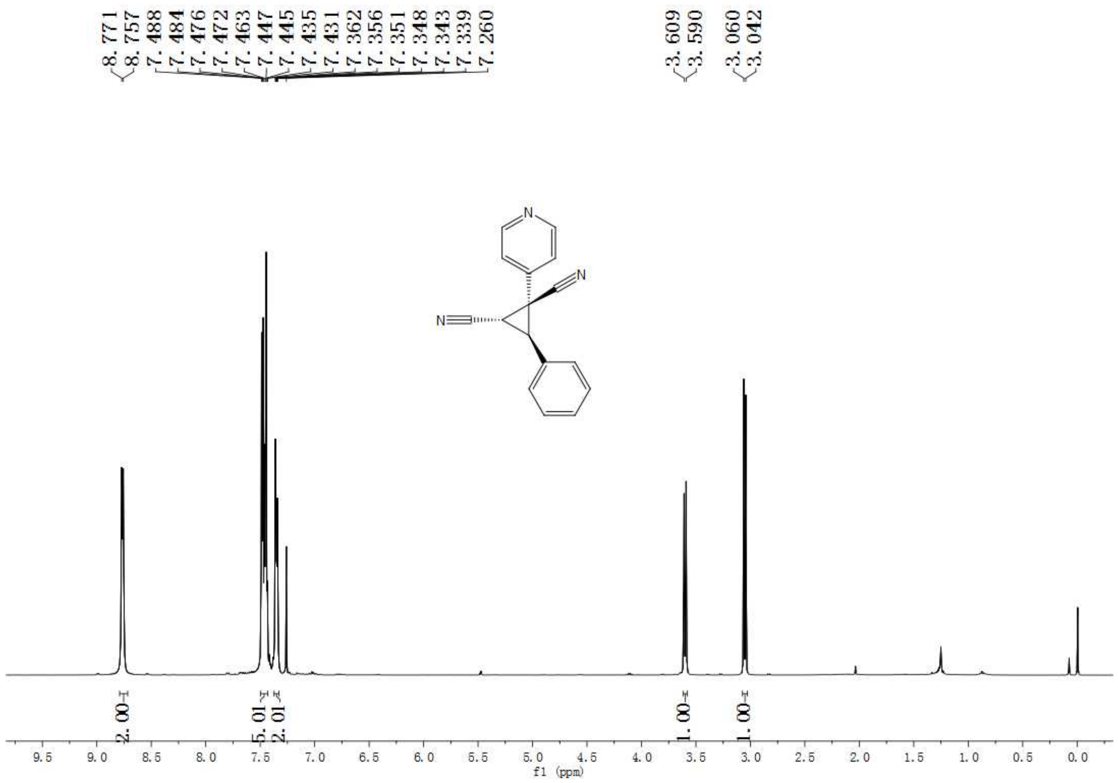

Figure S11. ^{1}H NMR Spectrum of *trans*-3c (400 MHz, $CDCl_3$)

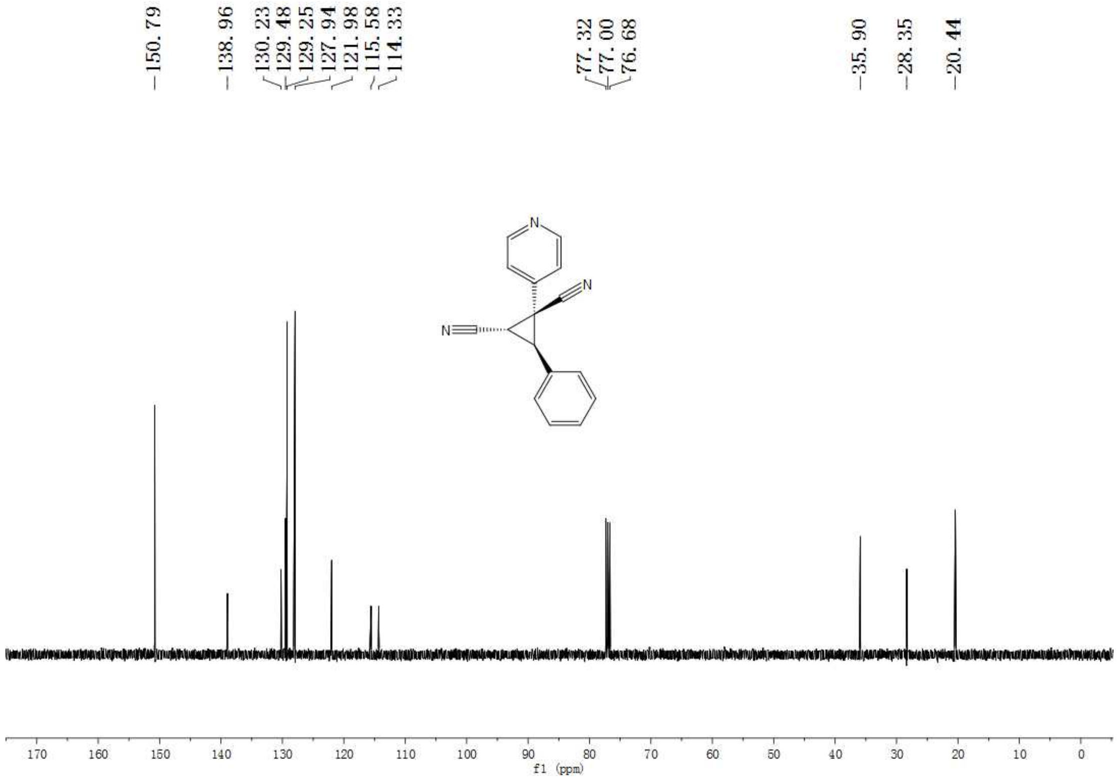

Figure S12. ^{13}C NMR Spectrum of *trans*-3c (100 MHz, $CDCl_3$)

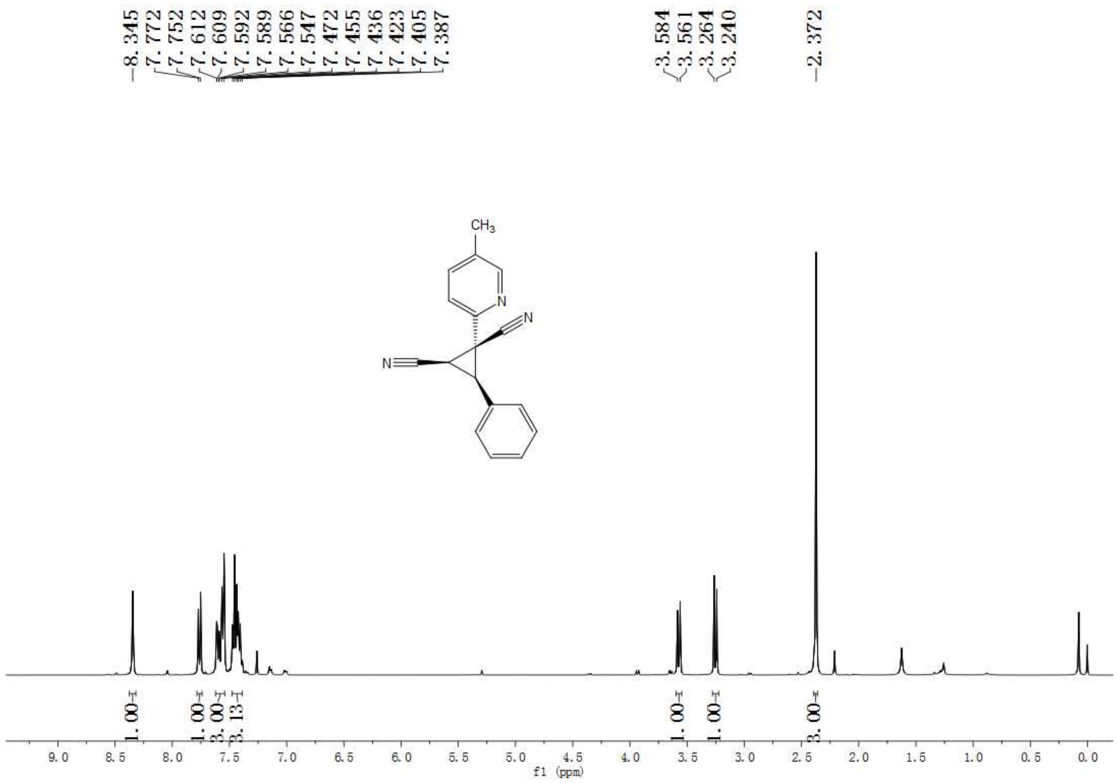

Figure S13. ^{1}H NMR Spectrum of *cis*-3d (400 MHz, $CDCl_3$)

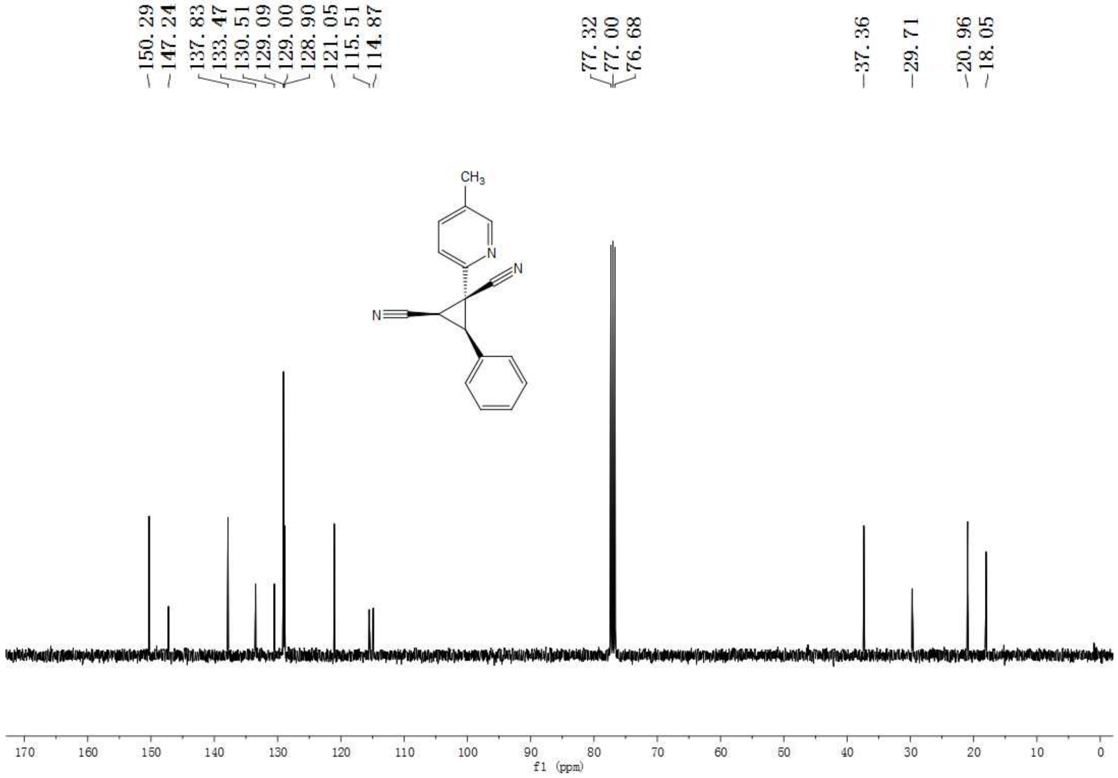

Figure S14. ^{13}C NMR Spectrum of *cis*-3d (100 MHz, $CDCl_3$)

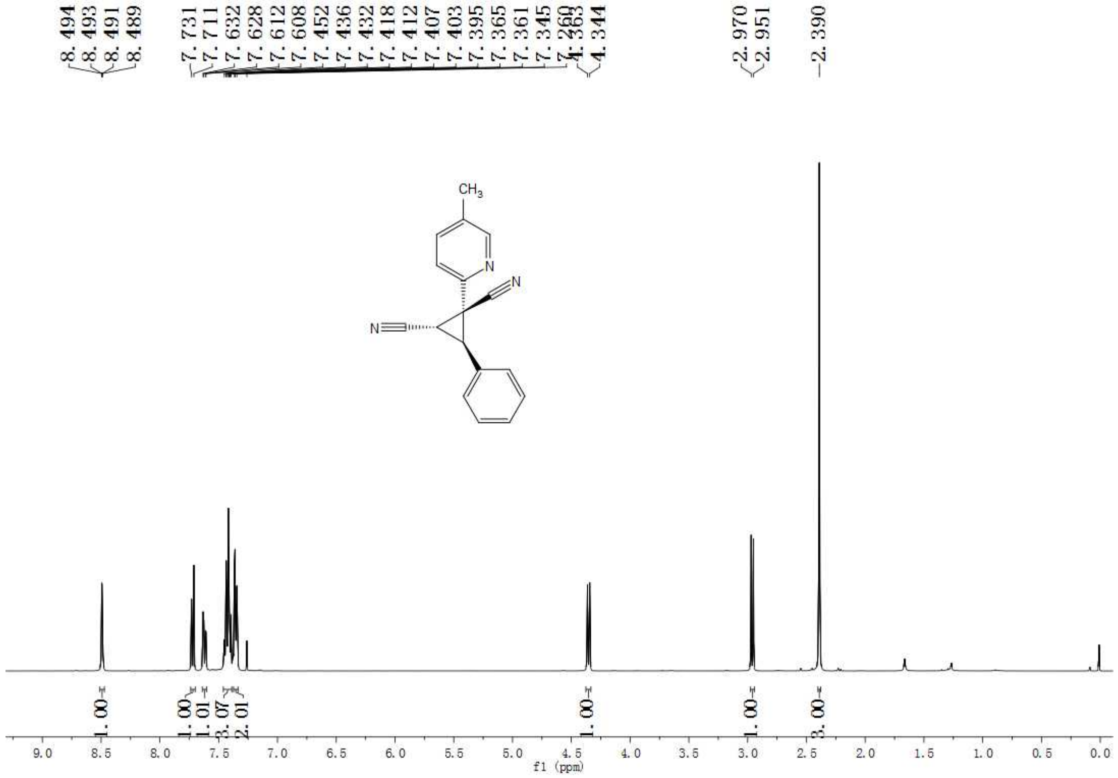

Figure S15. ^{1}H **NMR Spectrum of *trans*-3d (400 MHz,** $CDCl_3$**)**

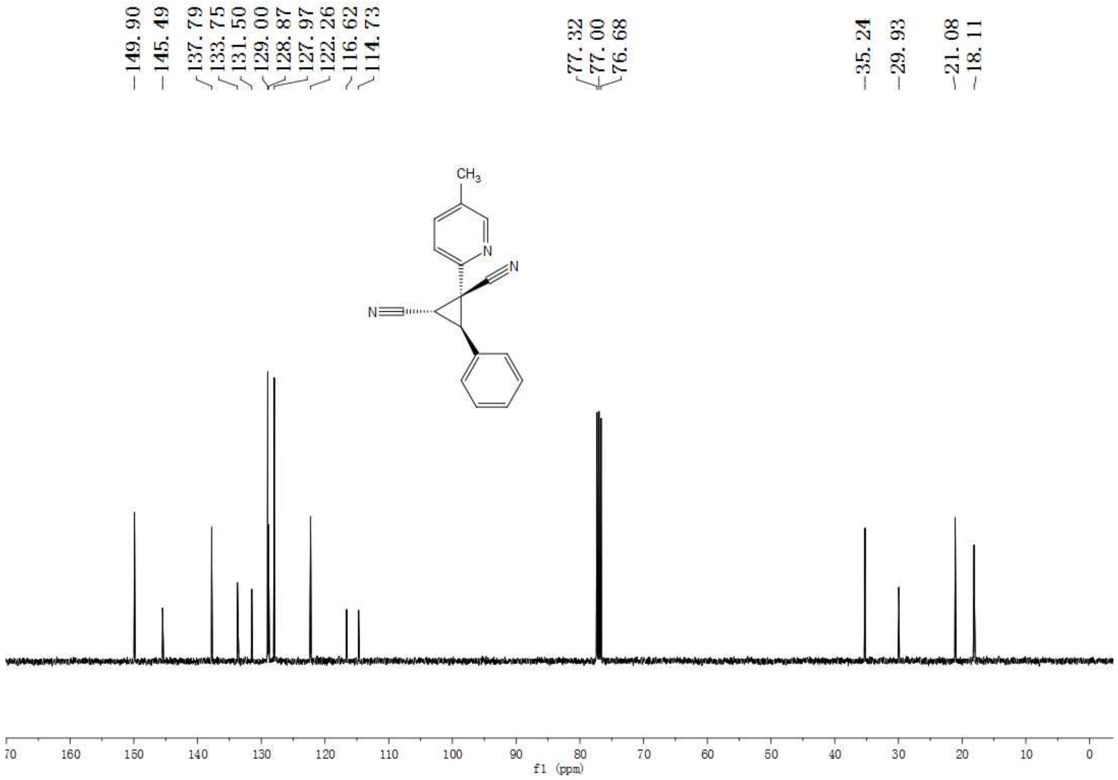

Figure S16. ^{13}C **NMR Spectrum of *trans*-3d (100 MHz,** $CDCl_3$**)**

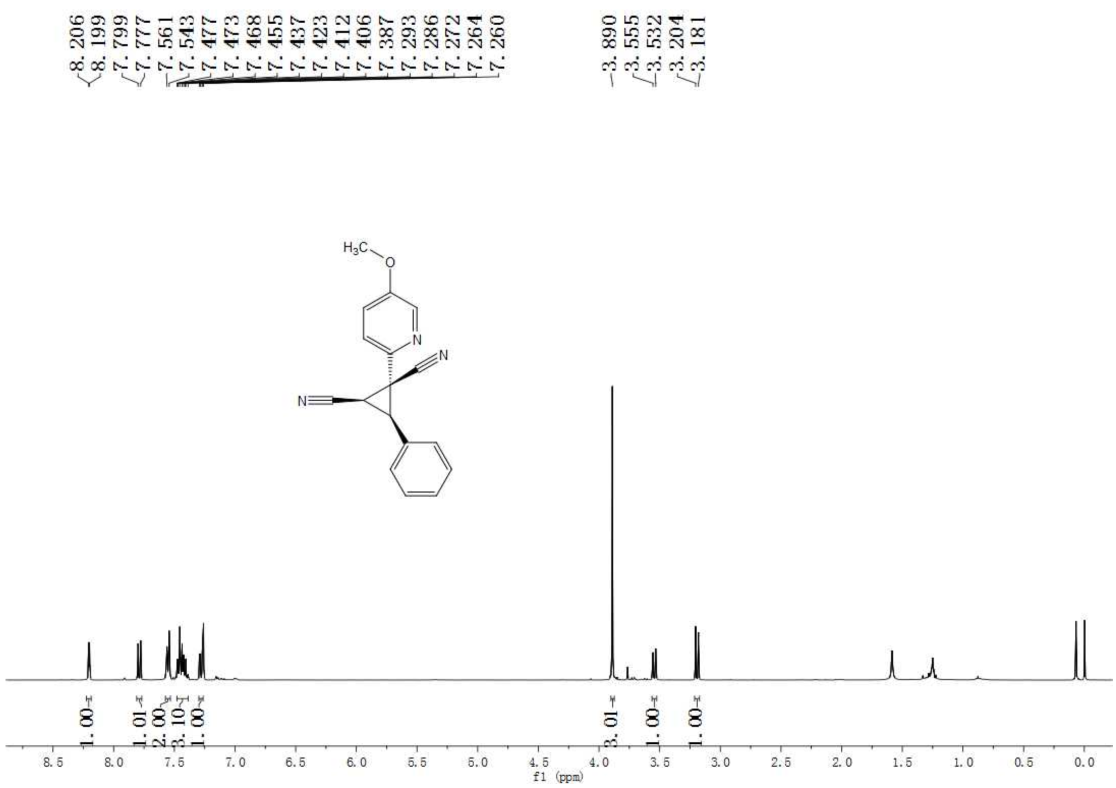

Figure S17. ^{1}H NMR Spectrum of *cis*-3e (400 MHz, $CDCl_3$)

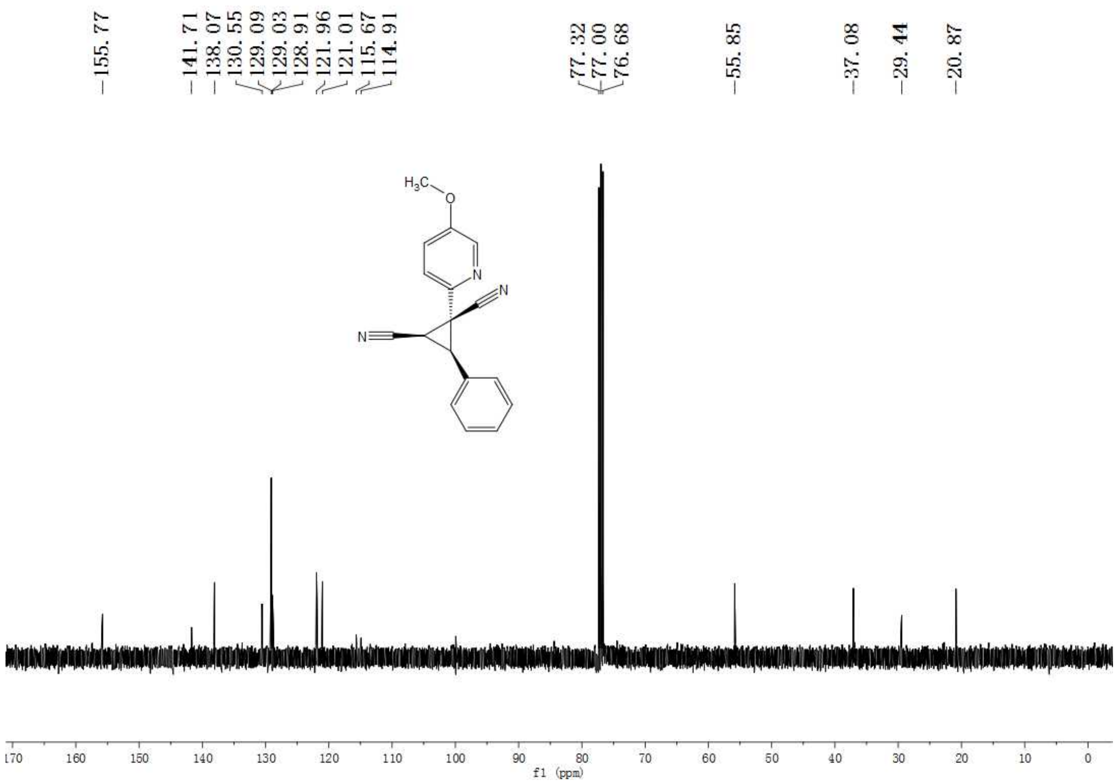

Figure S18. ^{13}C NMR Spectrum of *cis*-3e (100 MHz, $CDCl_3$)

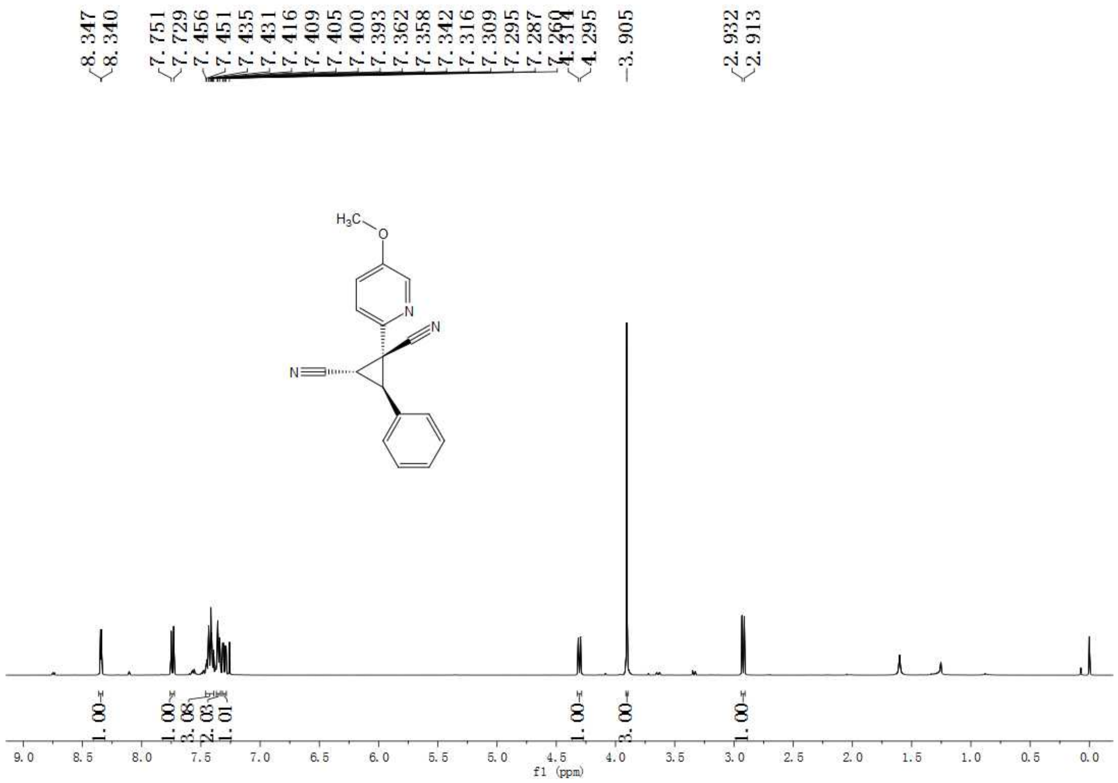

Figure S19. ^{1}H NMR Spectrum of *trans*-3e (400 MHz, CDCl$_3$)

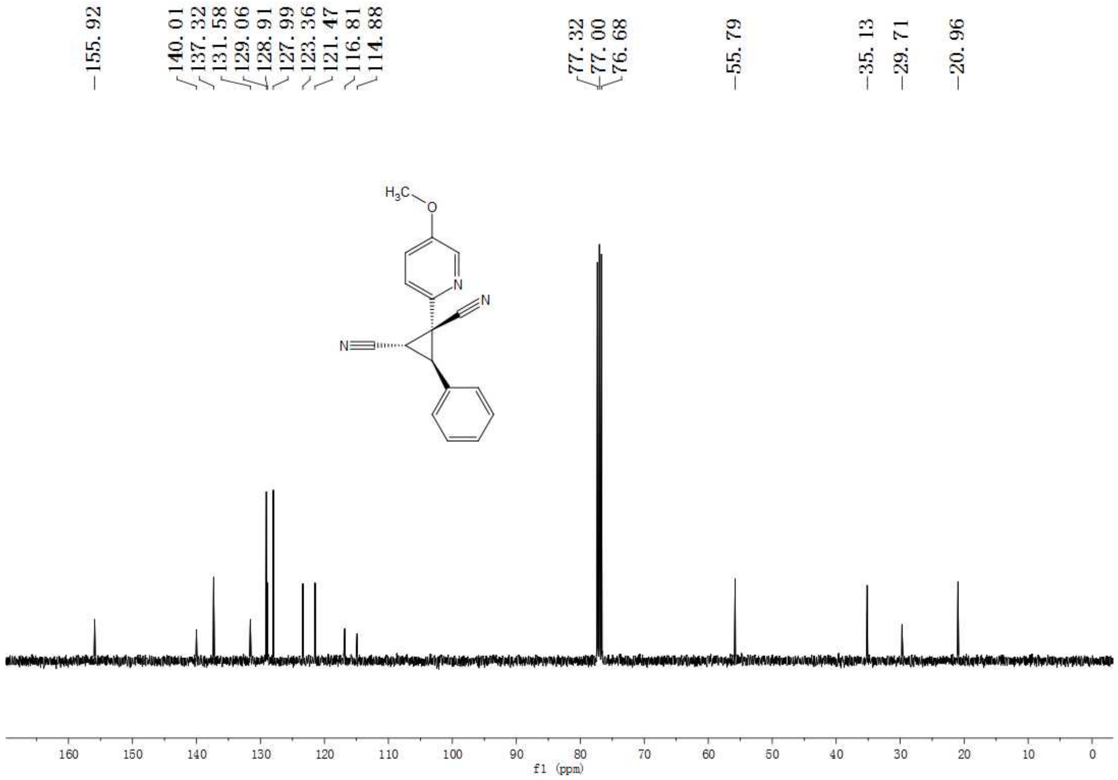

Figure S20. ^{13}C NMR Spectrum of *trans*-3e (100 MHz, CDCl$_3$)

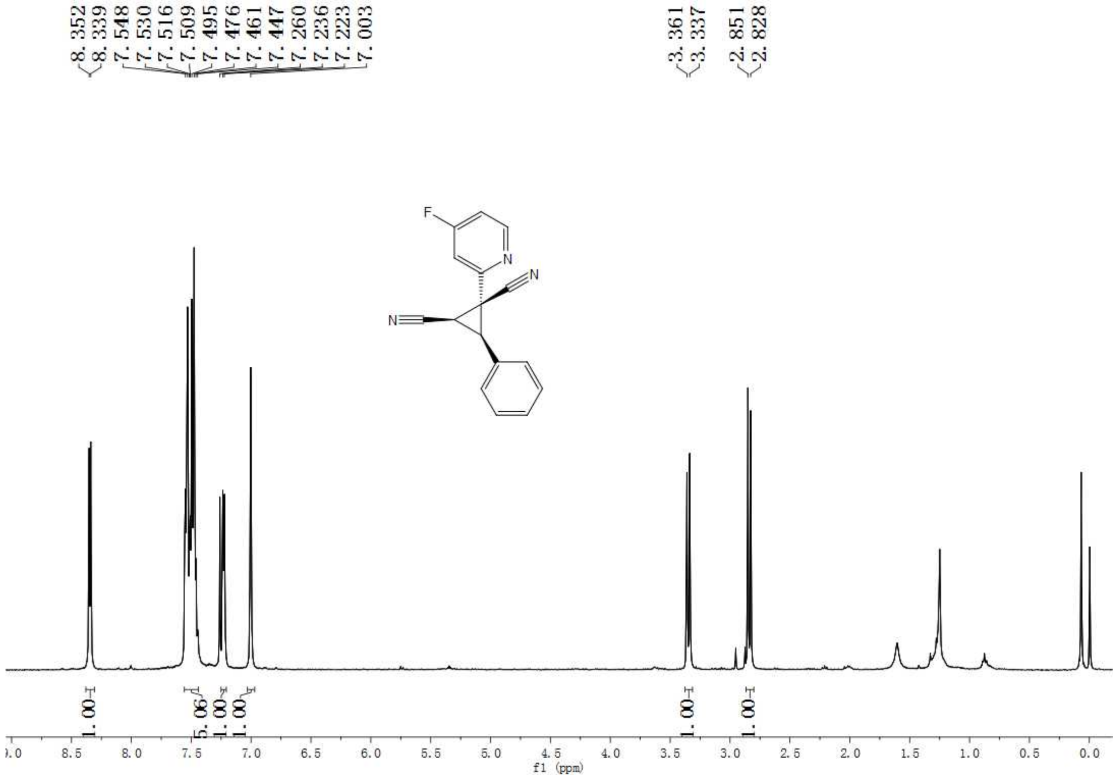

Figure S21. ^{1}H NMR Spectrum of *cis*-3f (400 MHz, $CDCl_3$)

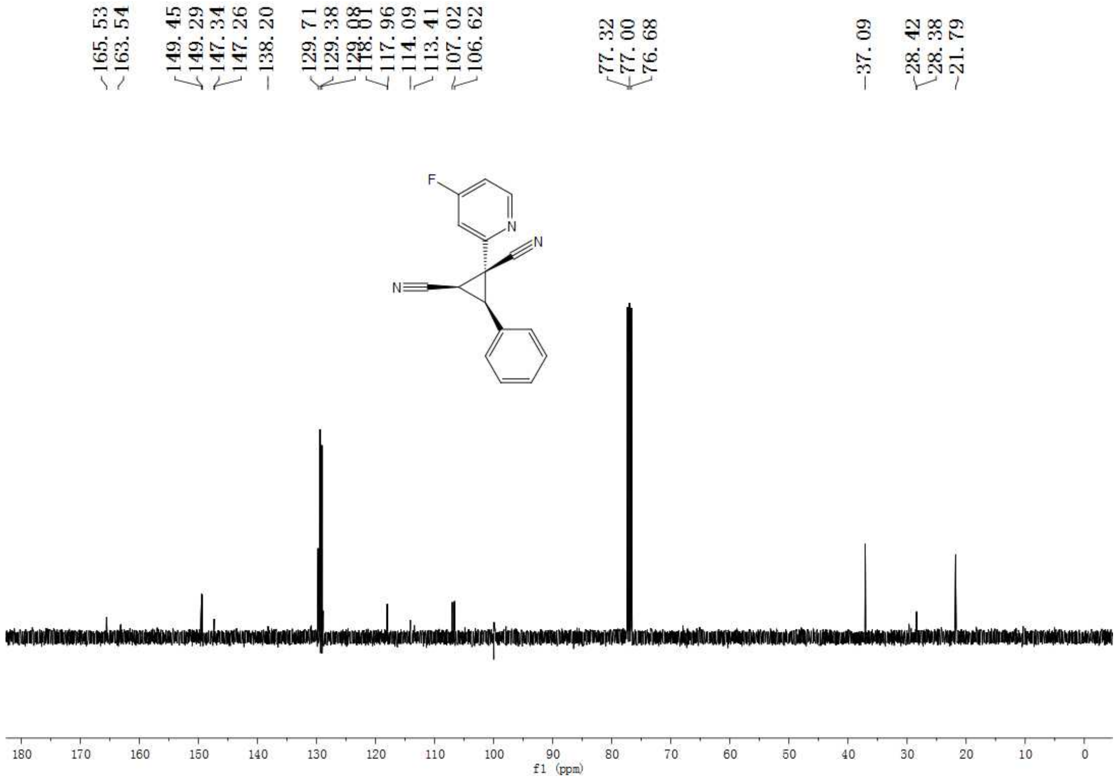

Figure S22. ^{13}C NMR Spectrum of *cis*-3f (100 MHz, $CDCl_3$)

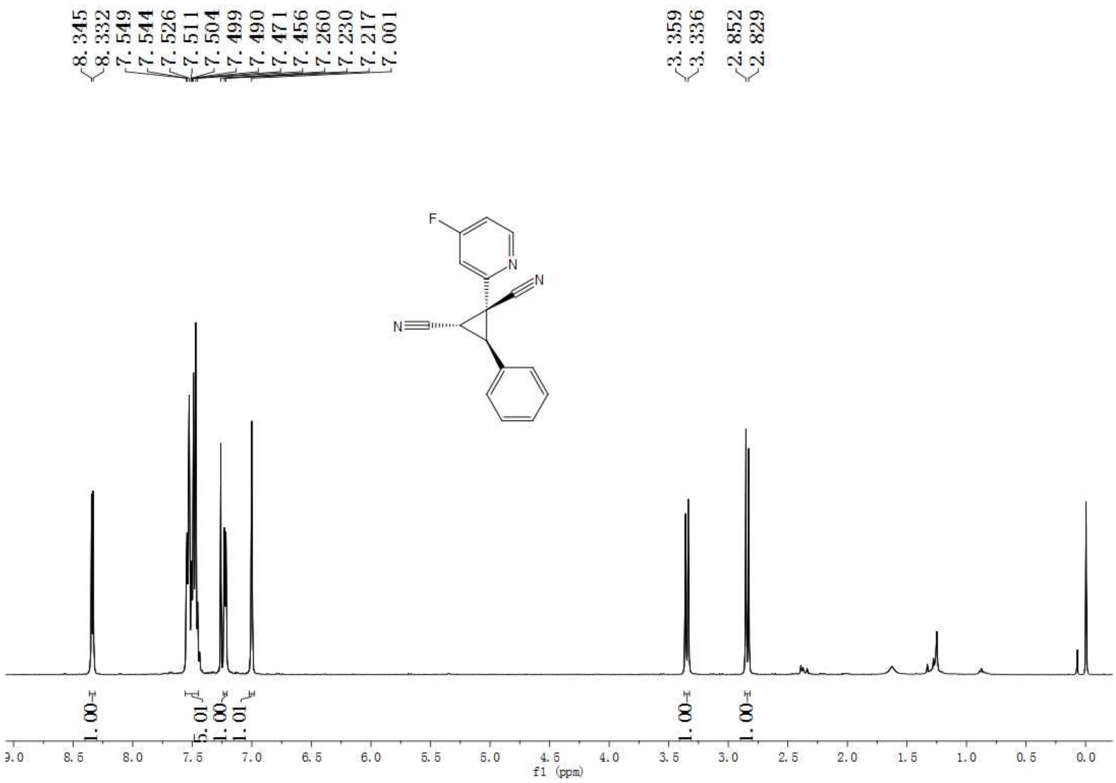

Figure S23. ^{1}H **NMR Spectrum of** ***trans*****-3f (400 MHz, $CDCl_3$)**

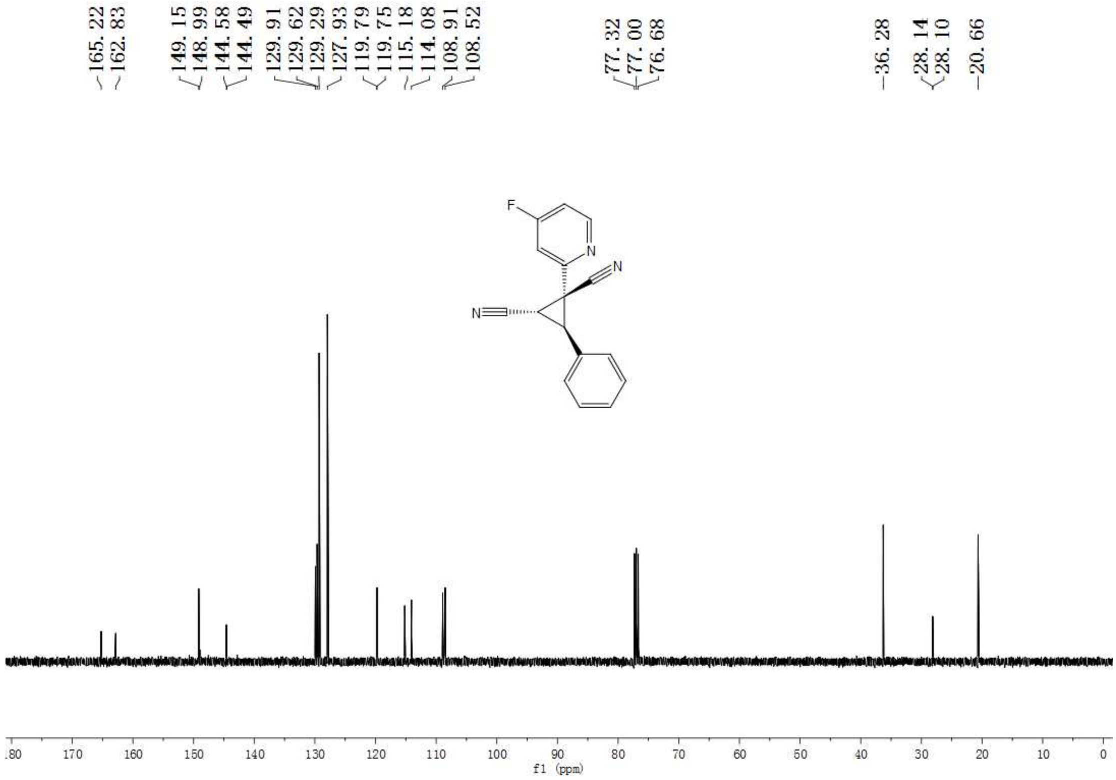

Figure S24. ^{13}C **NMR Spectrum of** ***trans*****-3f (100 MHz, $CDCl_3$)**

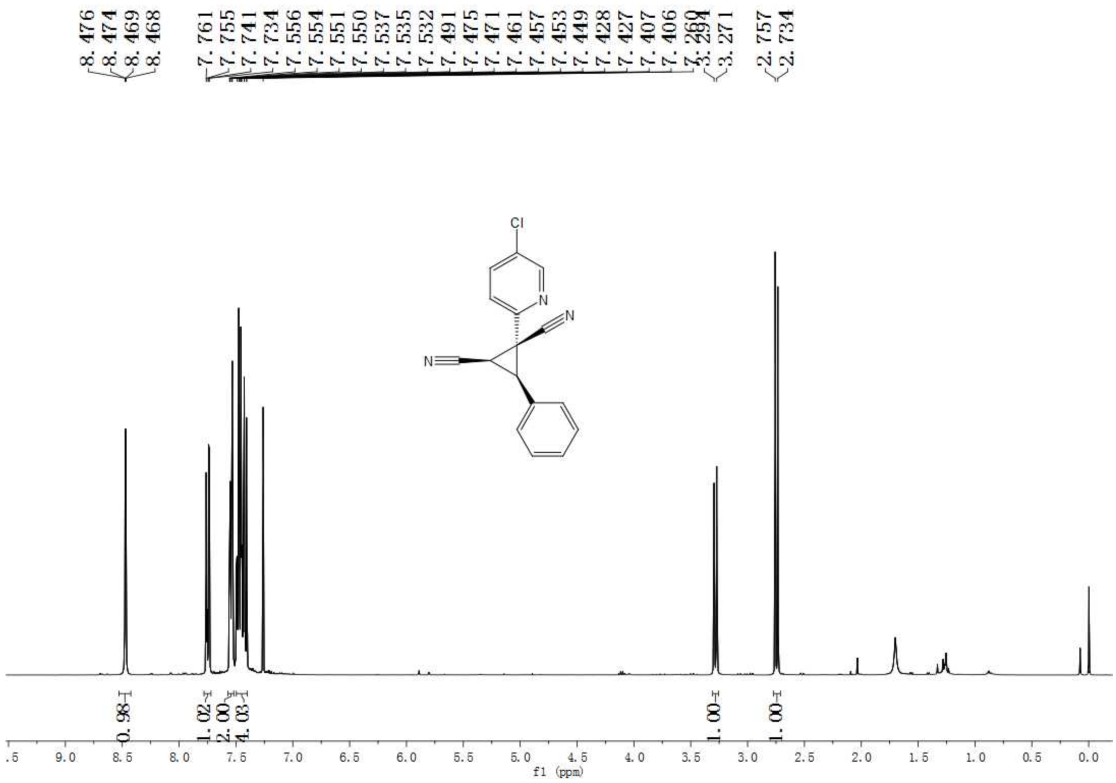

Figure S25. ^{1}H NMR Spectrum of *cis*-3g (400 MHz, $CDCl_3$)

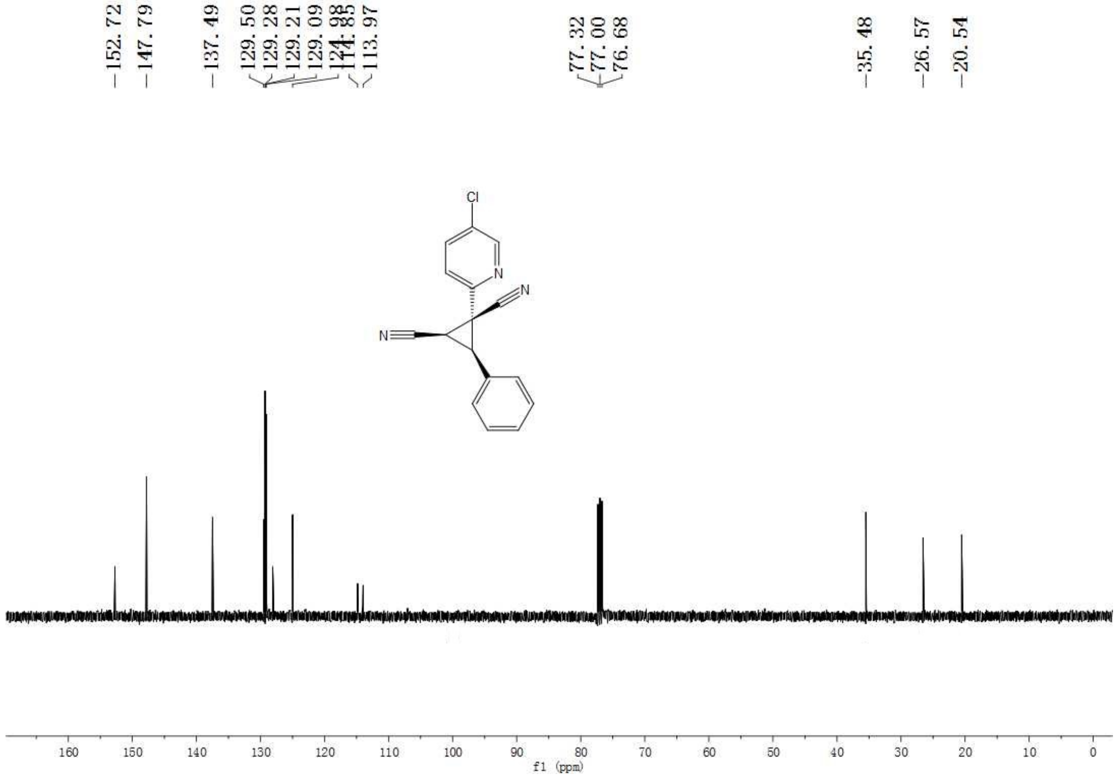

Figure S26. ^{13}C NMR Spectrum of *cis*-3g (100 MHz, $CDCl_3$)

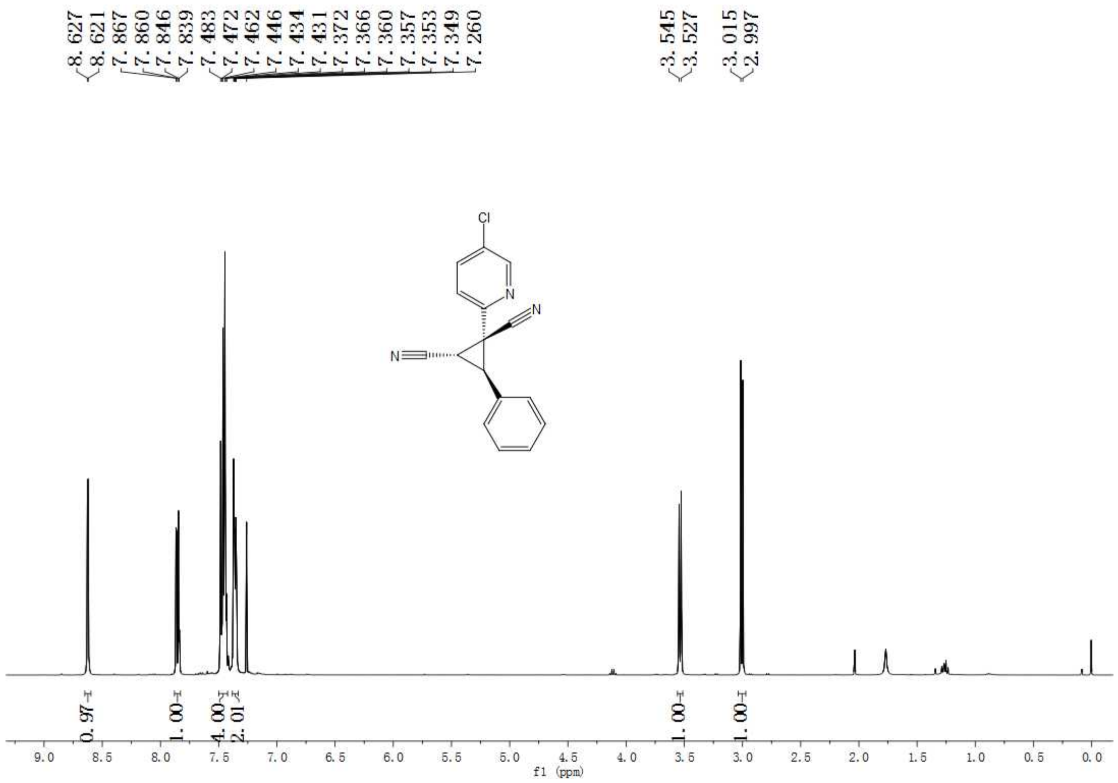

Figure S27. ^{1}H NMR Spectrum of *trans*-3g (400 MHz, $CDCl_3$)

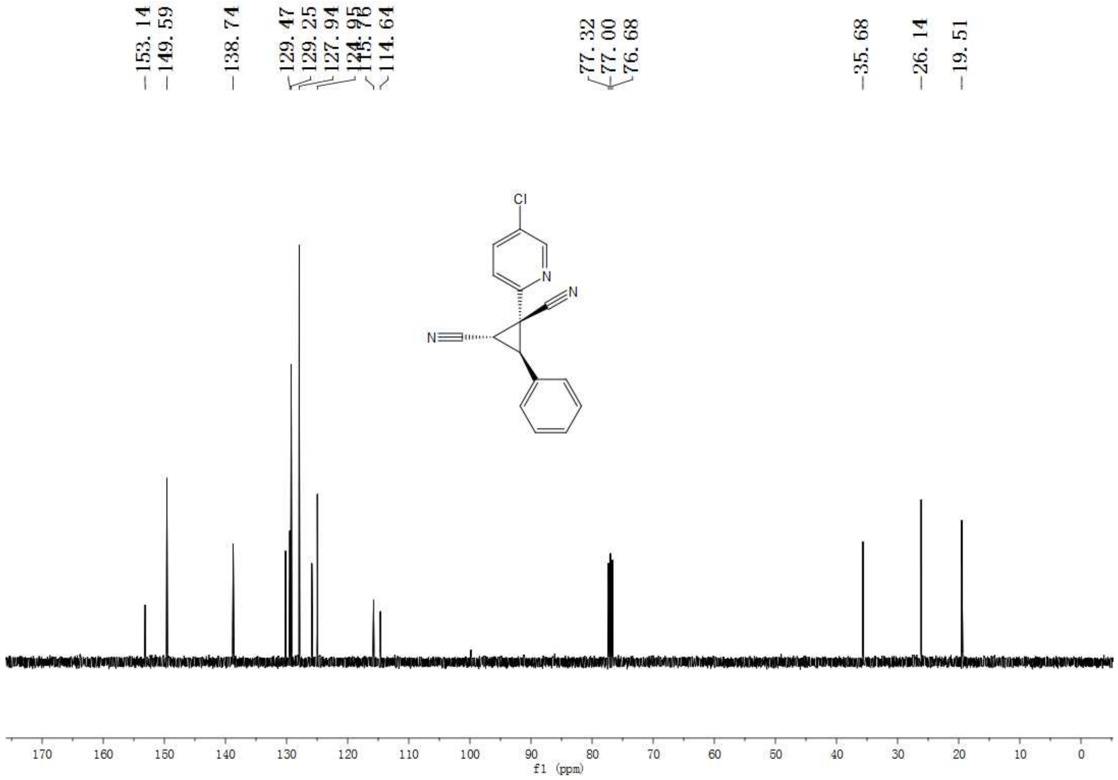

Figure S28. ^{13}C NMR Spectrum of *trans*-3g (100 MHz, $CDCl_3$)

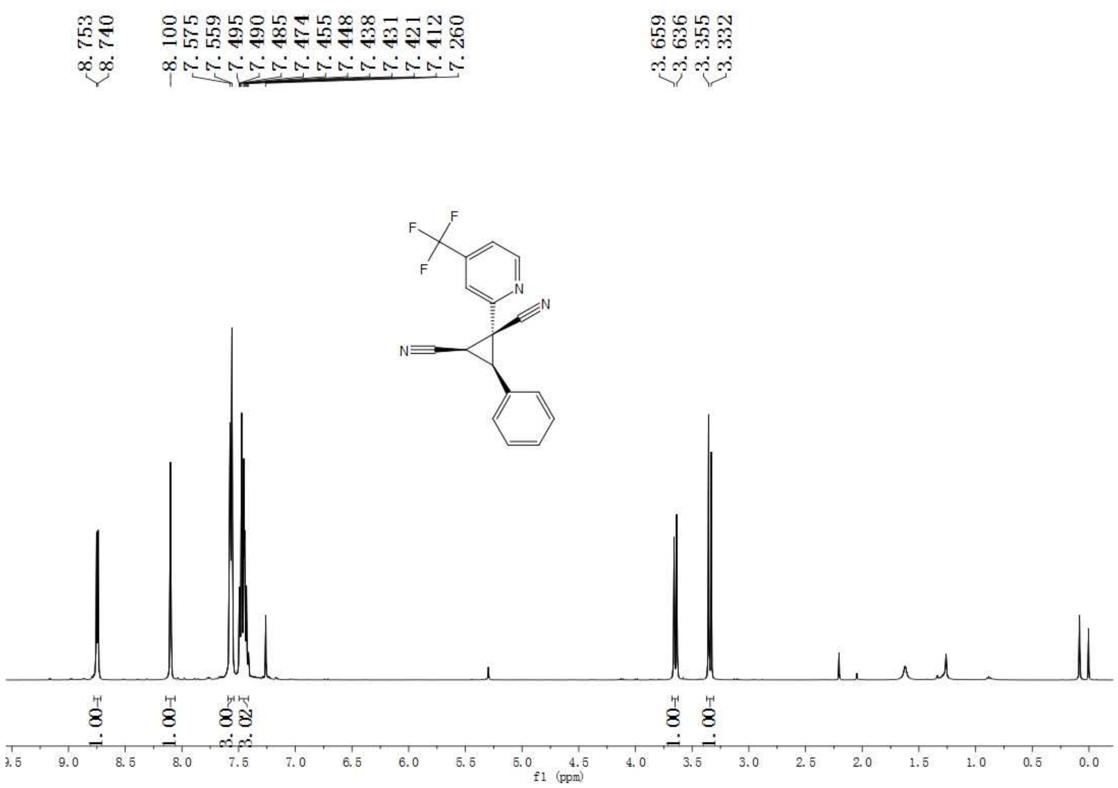

Figure S29. ^{1}**H NMR Spectrum of** ***cis*****-3h (400 MHz, $CDCl_3$)**

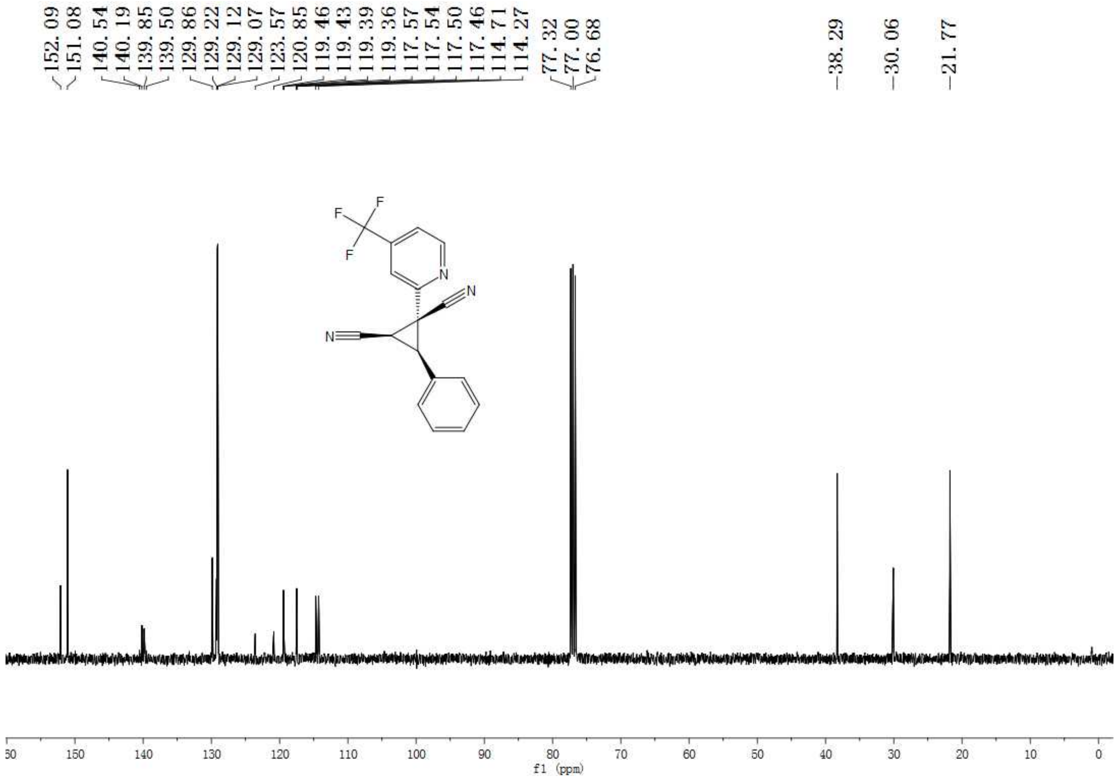

Figure S30. ^{13}**C NMR Spectrum of** ***cis*****-3h (100 MHz, $CDCl_3$)**

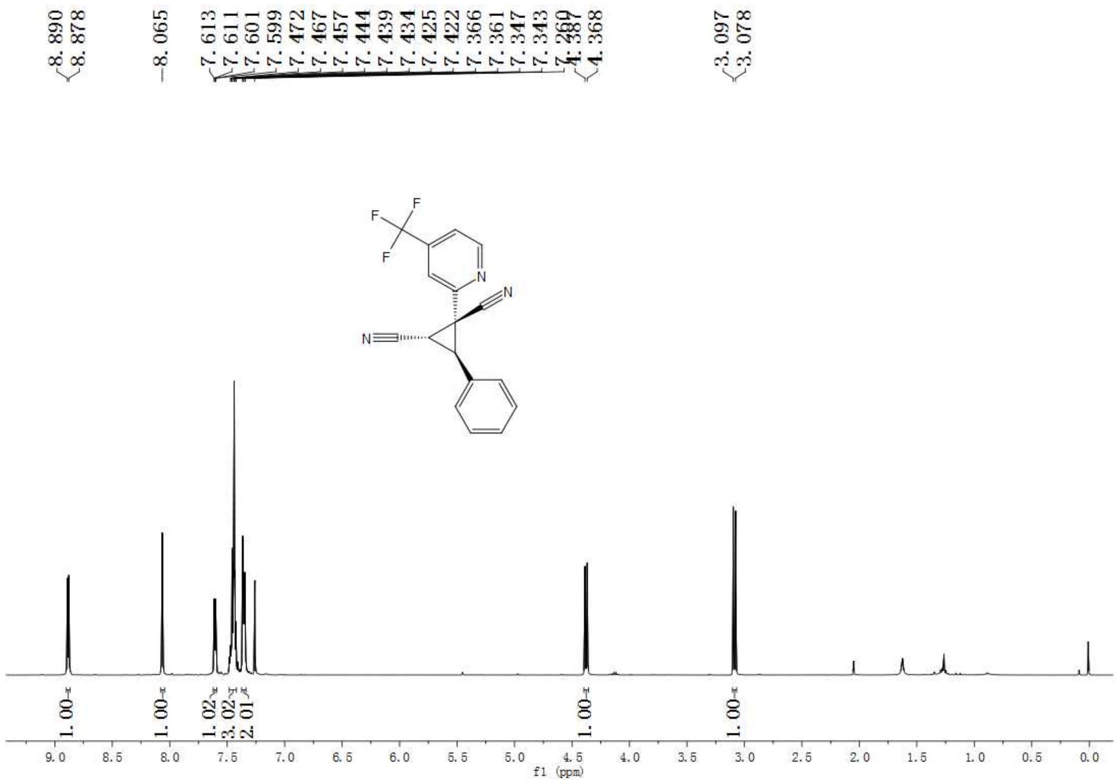

Figure S31. ^{1}H NMR Spectrum of *trans*-3h (400 MHz, $CDCl_3$)

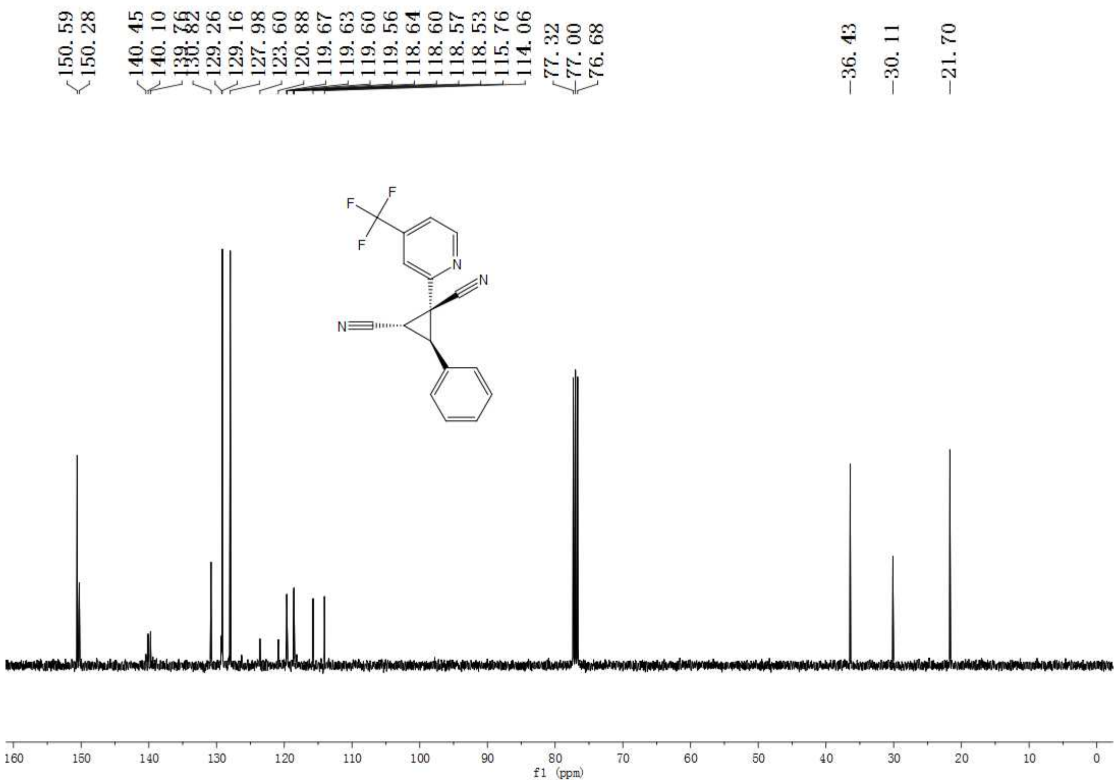

Figure S32. ^{13}C NMR Spectrum of *trans*-3h (100 MHz, $CDCl_3$)

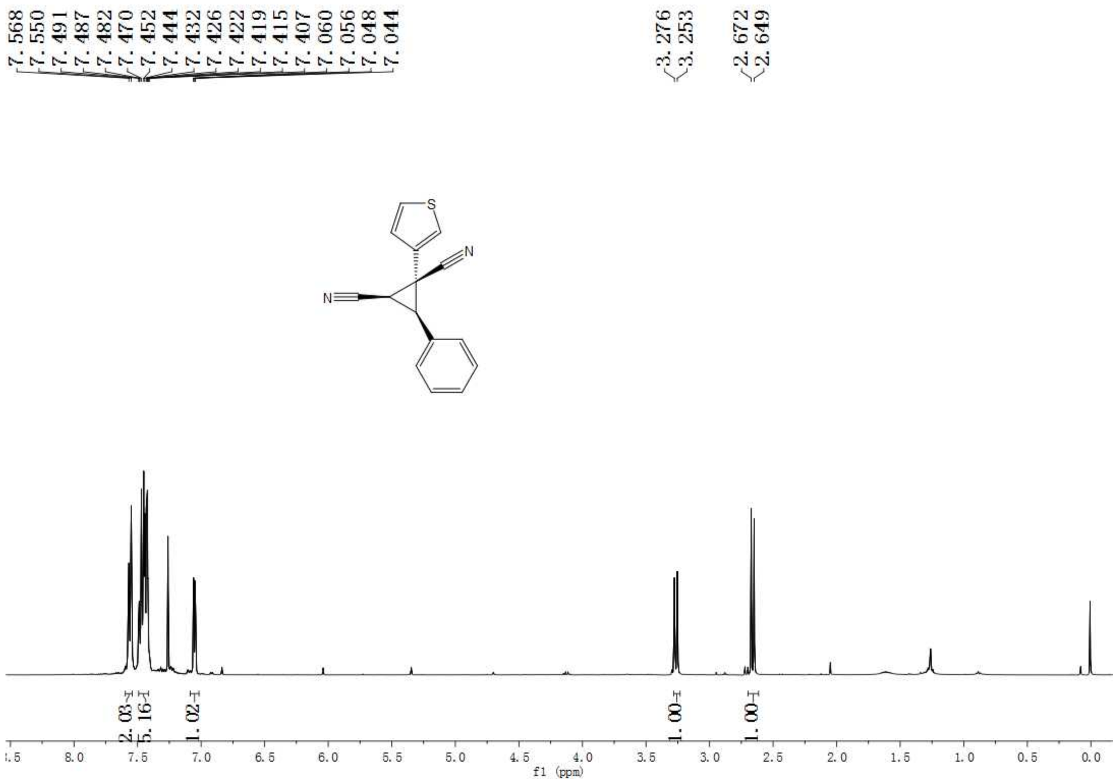

Figure S33. ^{1}H NMR Spectrum of *cis*-3i (400 MHz, $CDCl_3$)

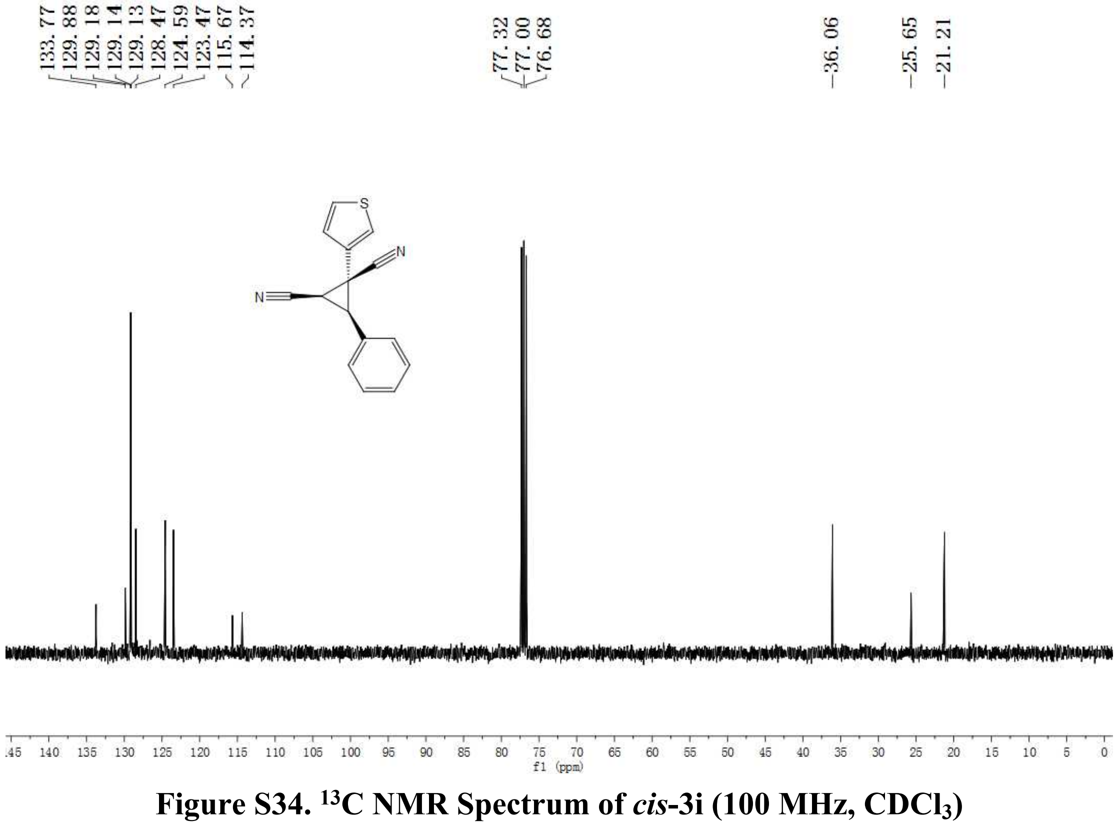

Figure S34. ^{13}C NMR Spectrum of *cis*-3i (100 MHz, $CDCl_3$)

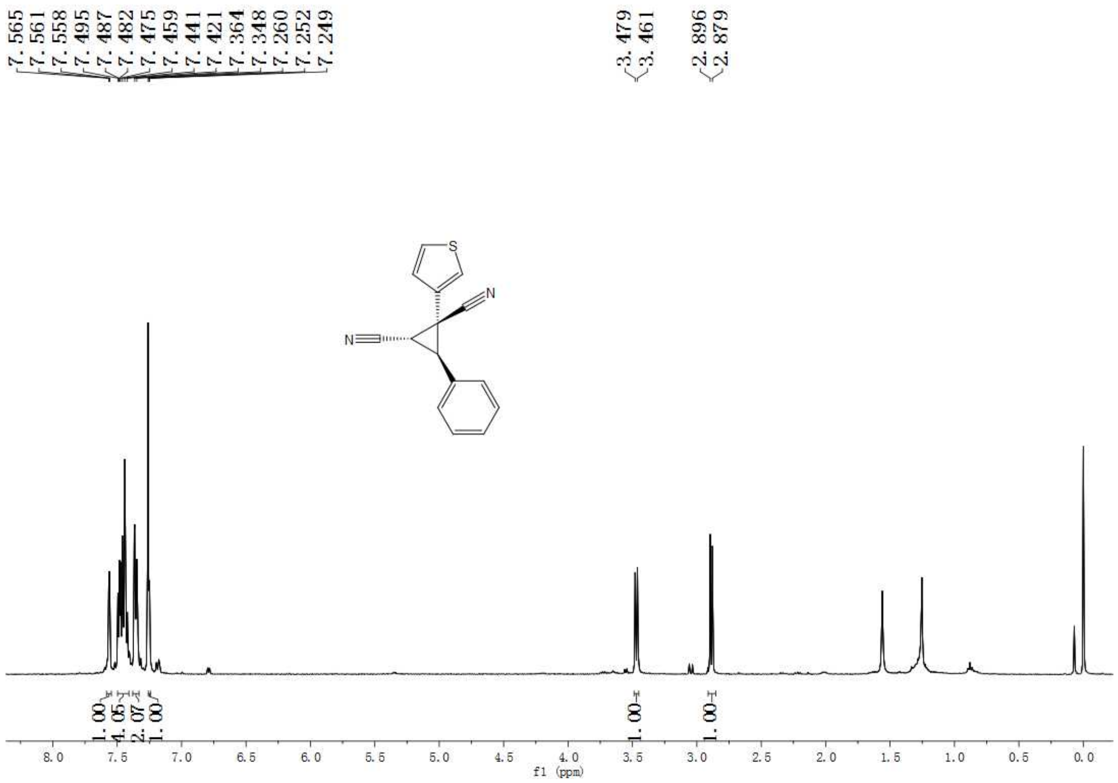

Figure S35. ^{1}H NMR Spectrum of *trans*-3i (400 MHz, $CDCl_3$)

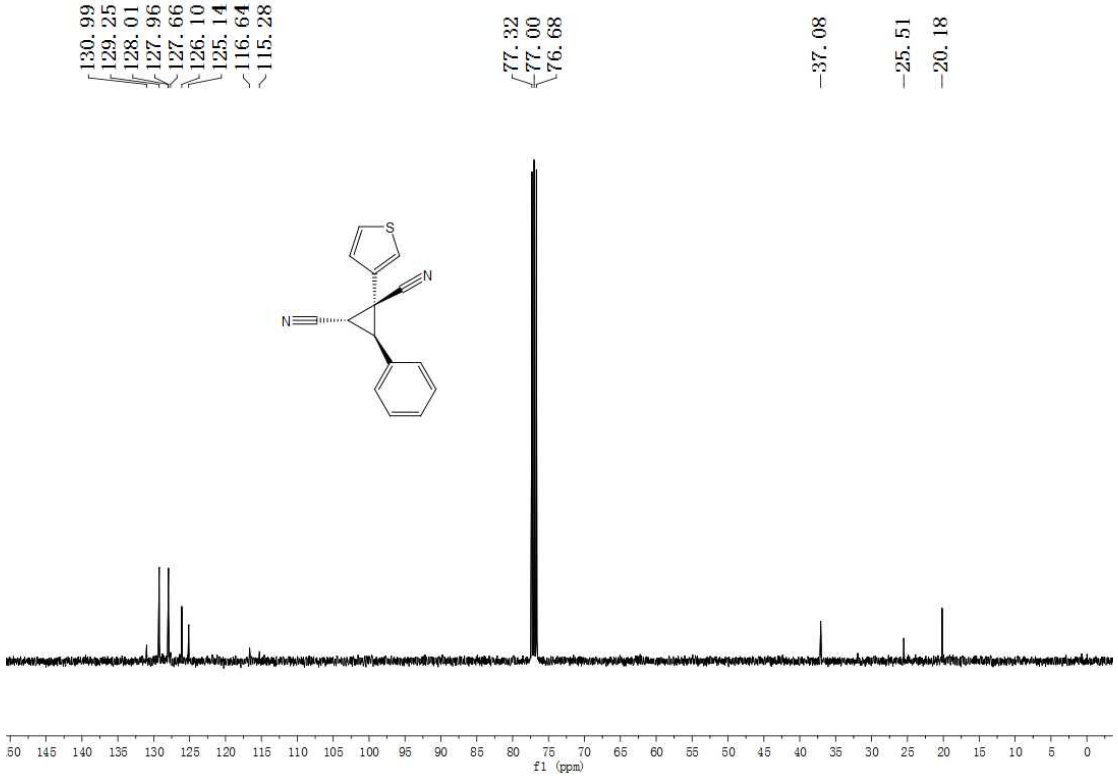

Figure S36. ^{13}C NMR Spectrum of *trans*-3i (100 MHz, $CDCl_3$)

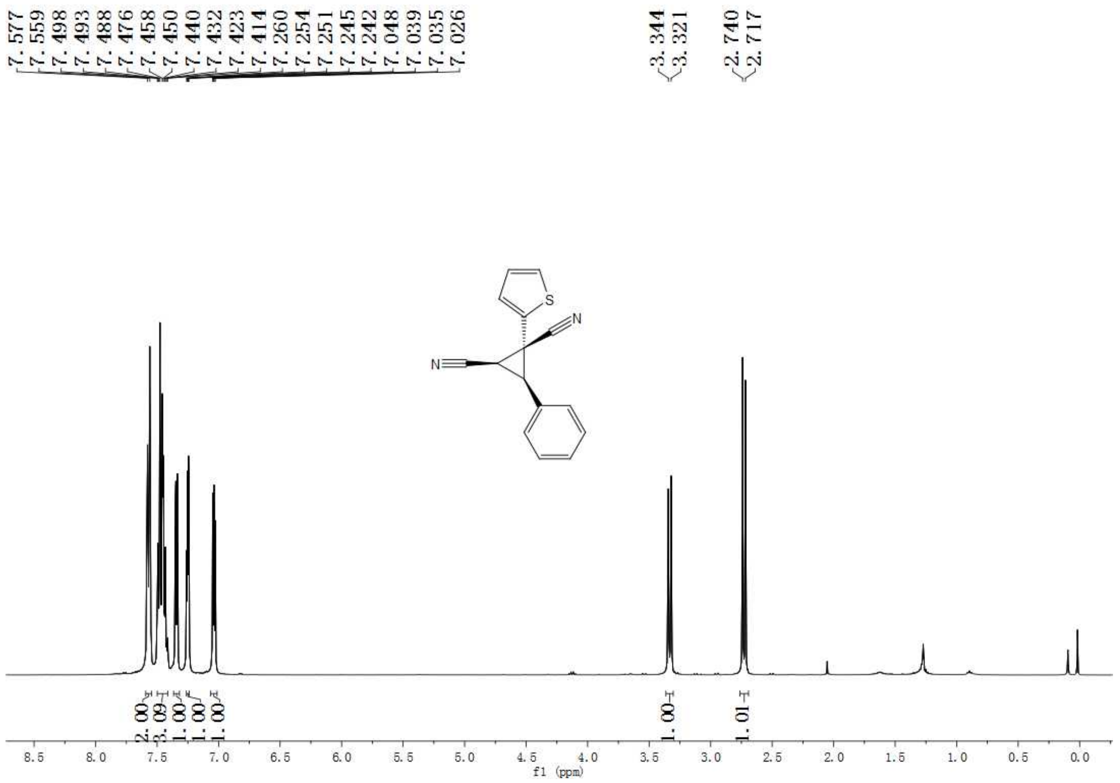

Figure S37. ^{1}H NMR Spectrum of *cis*-3j (400 MHz, $CDCl_3$)

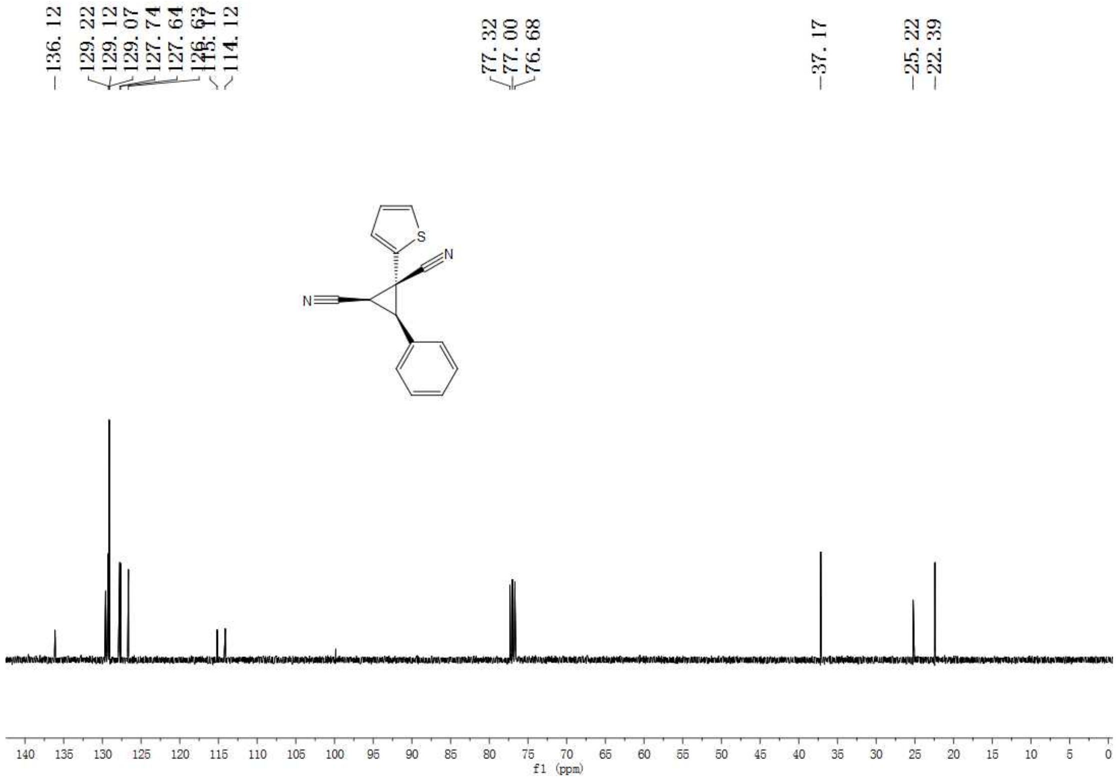

Figure S38. ^{13}C NMR Spectrum of *cis*-3j (100 MHz, $CDCl_3$)

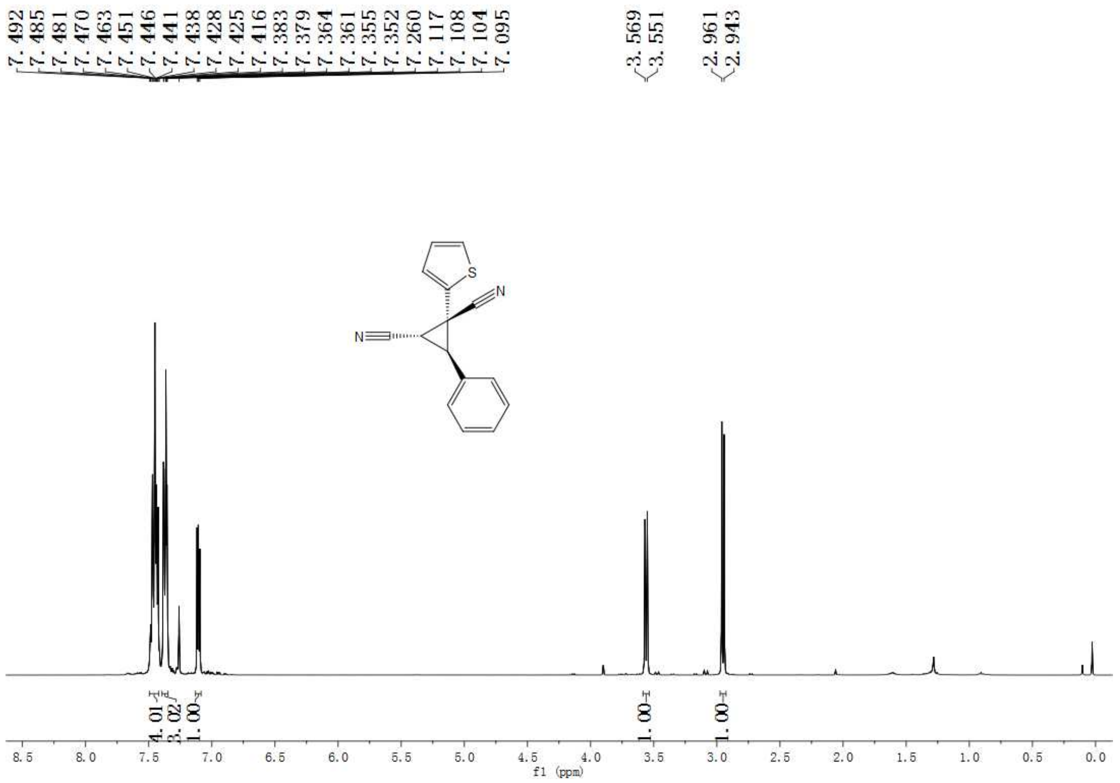

Figure S39. ^{1}H NMR Spectrum of *trans*-3j (400 MHz, $CDCl_3$)

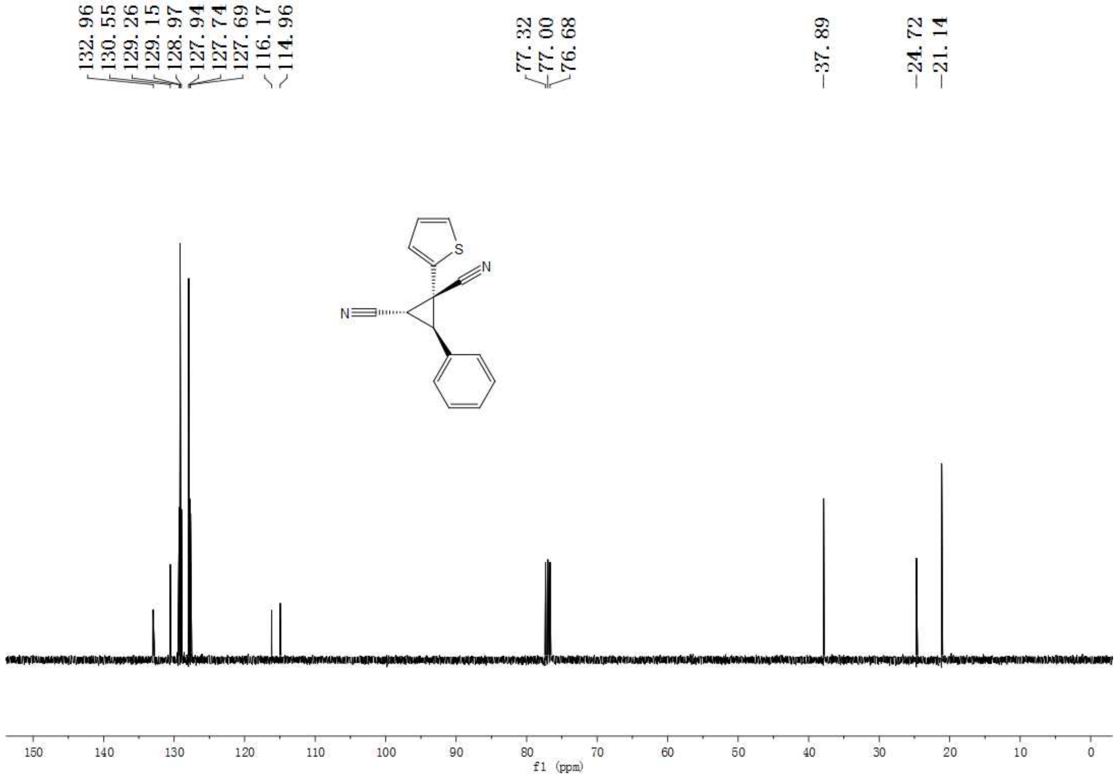

Figure S40. ^{13}C NMR Spectrum of *trans*-3j (100 MHz, $CDCl_3$)

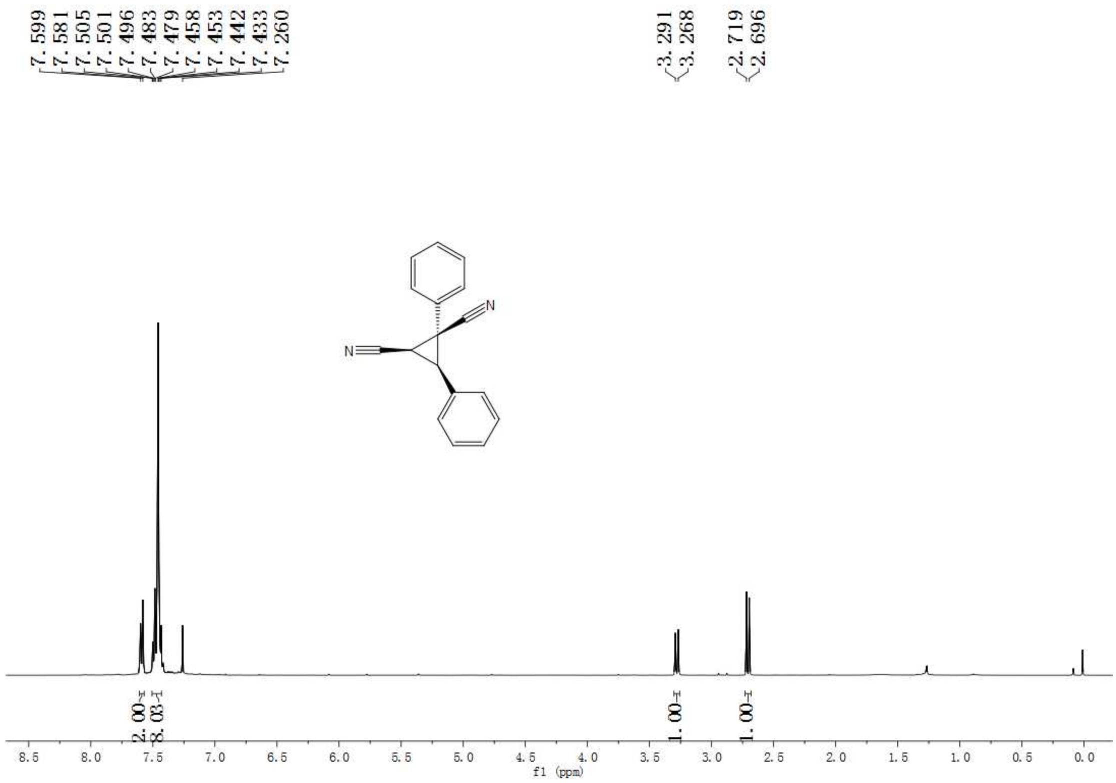

Figure S41. ^{1}H NMR Spectrum of *cis*-3k (400 MHz, $CDCl_3$)

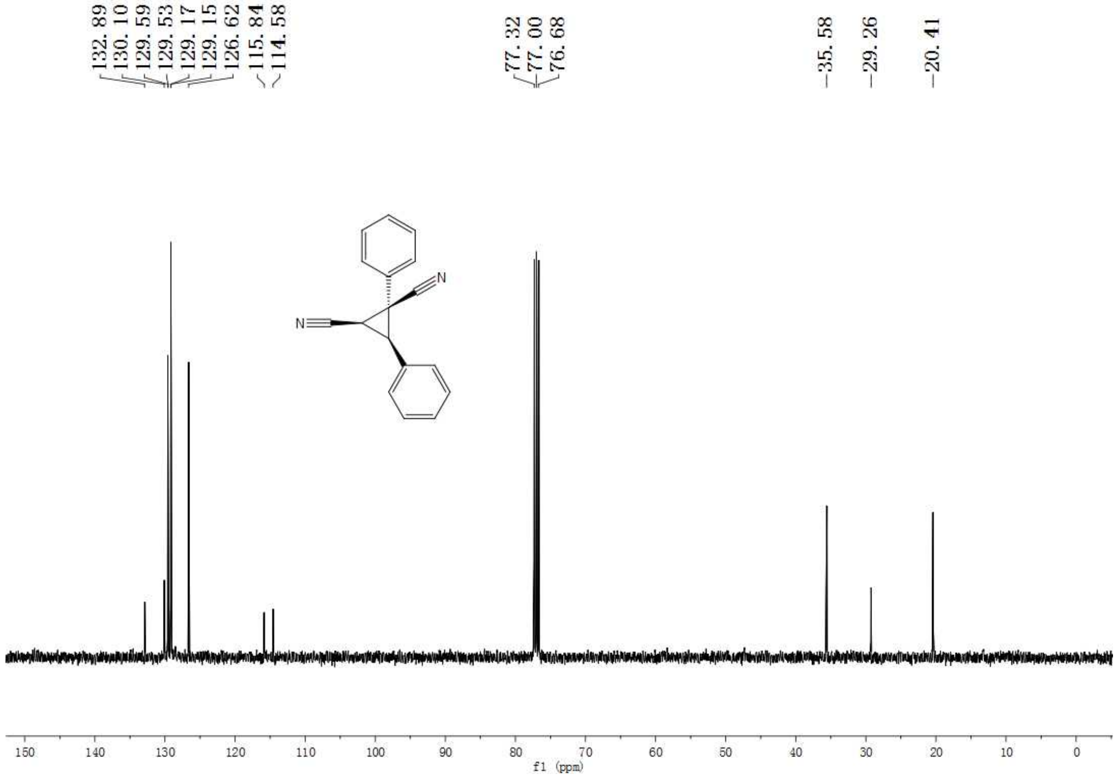

Figure S42. ^{13}C NMR Spectrum of *cis*-3k (100 MHz, $CDCl_3$)

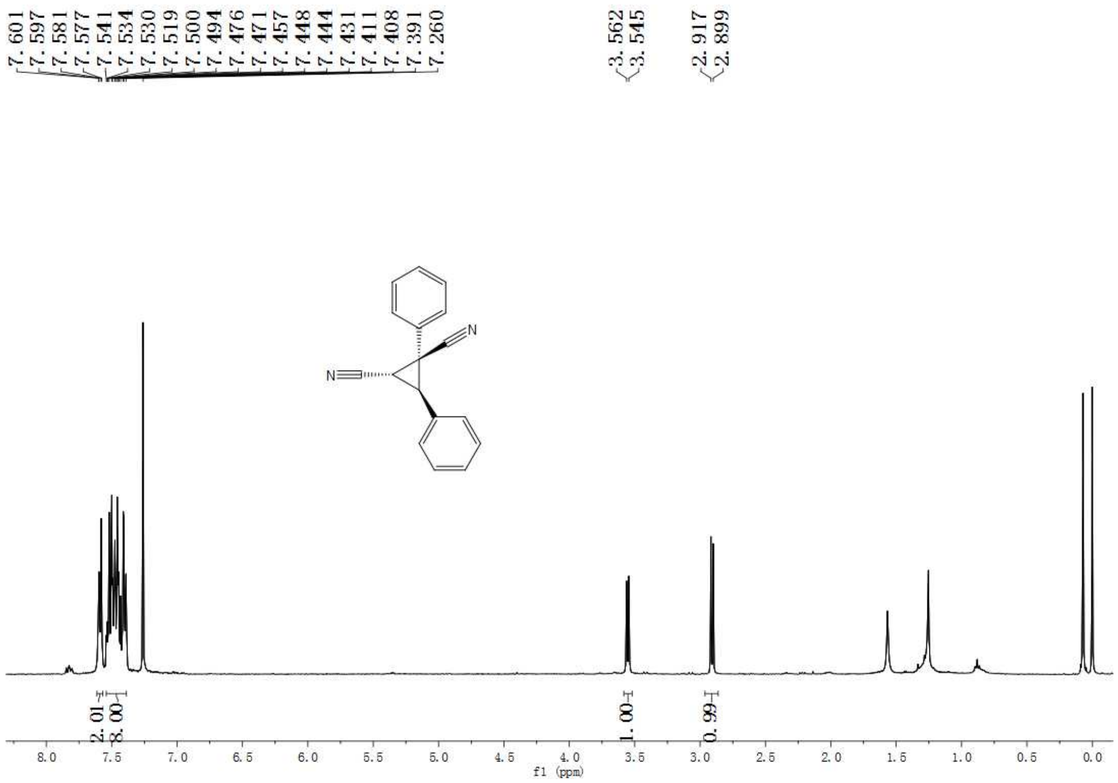

Figure S43. **^{1}H NMR Spectrum of *trans*-3k (400 MHz, $CDCl_3$)**

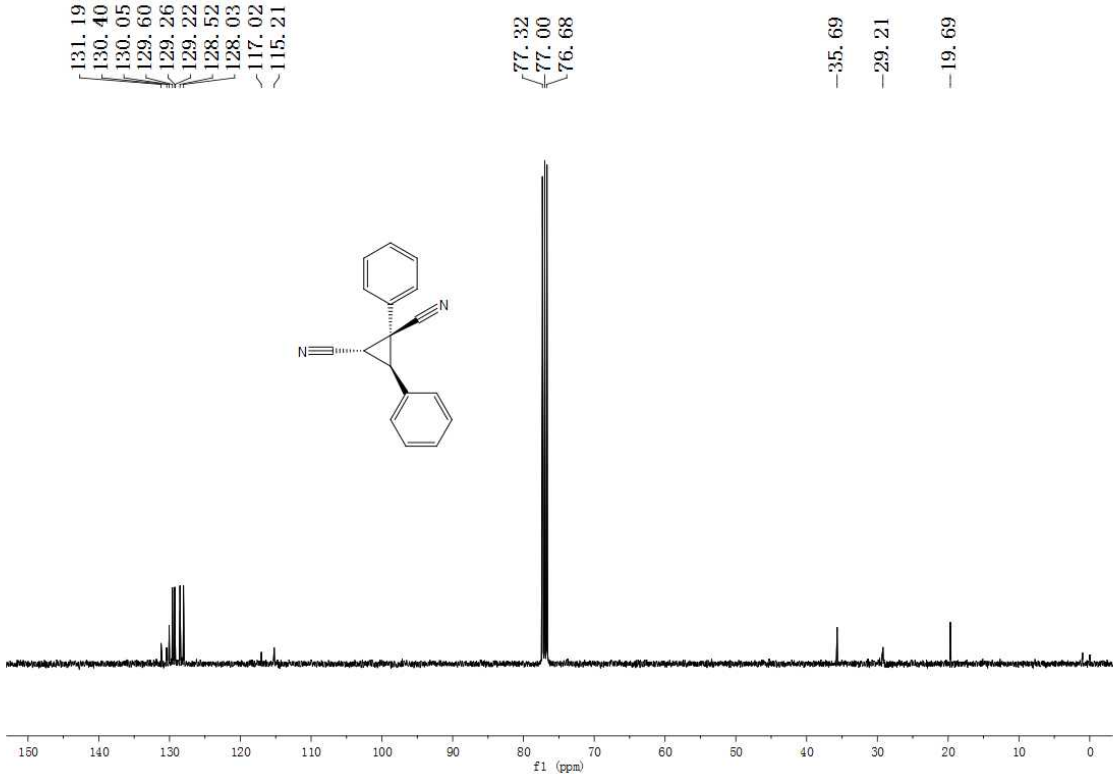

Figure S44. **^{13}C NMR Spectrum of *trans*-3k (100 MHz, $CDCl_3$)**

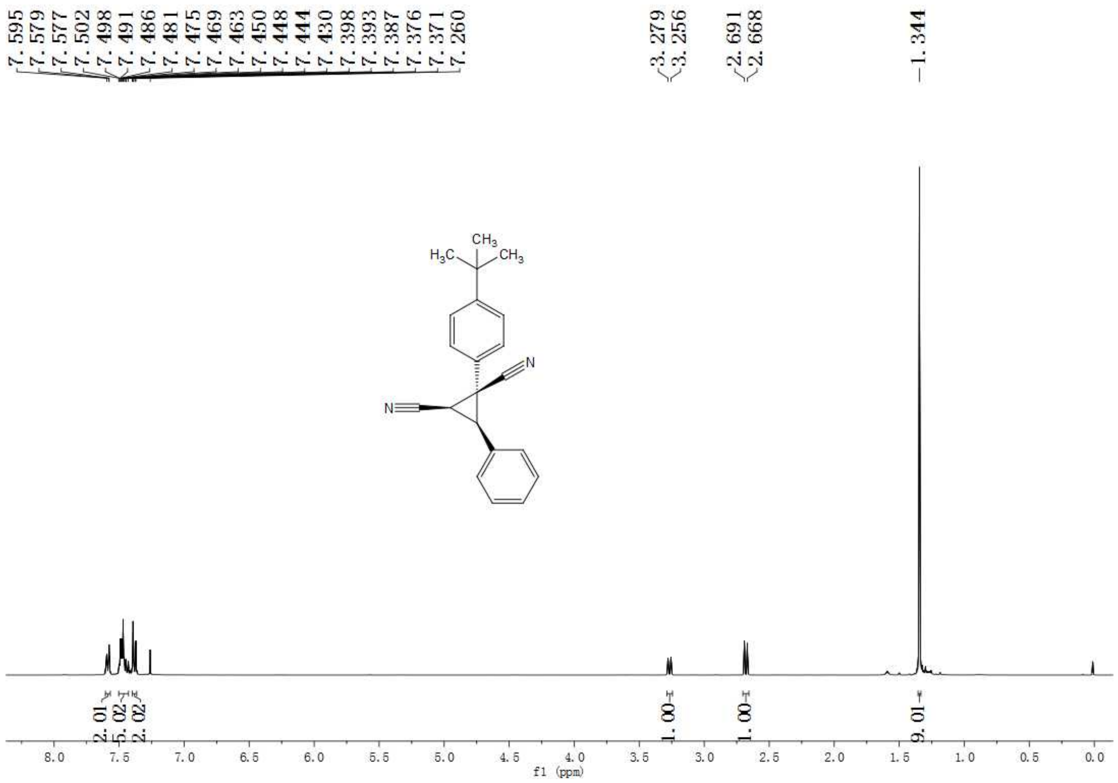

Figure S45. ^{1}H NMR Spectrum of *cis*-3l (400 MHz, $CDCl_3$)

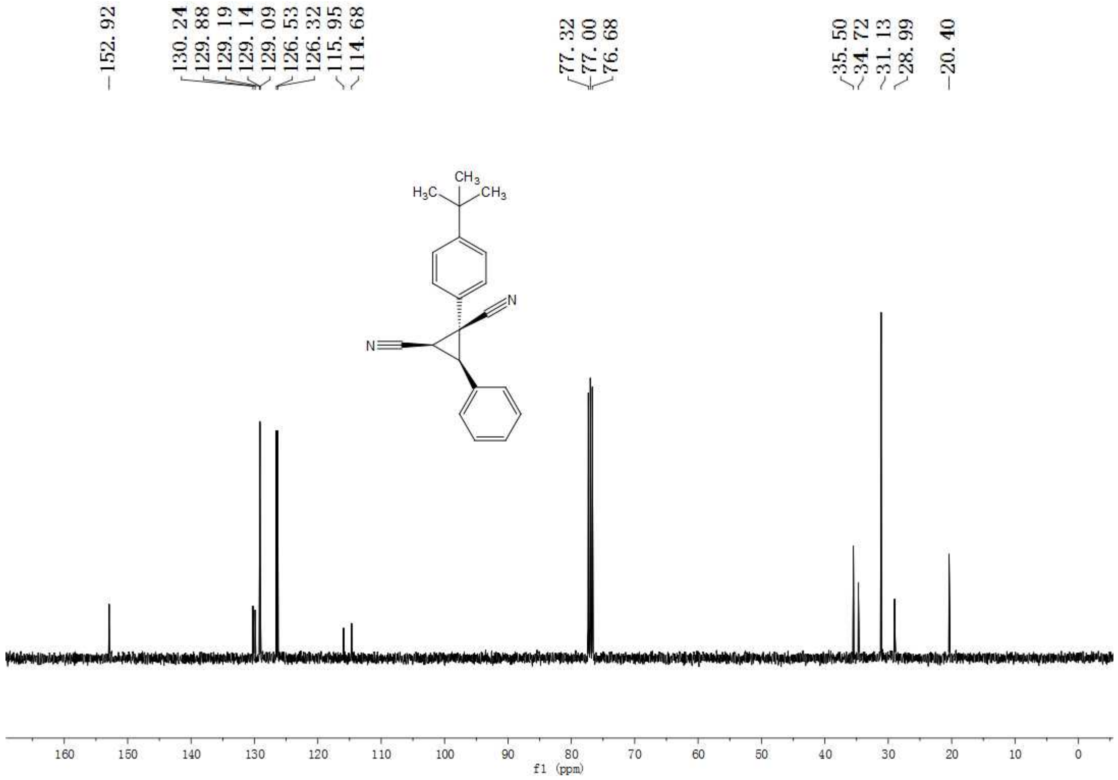

Figure S46. ^{13}C NMR Spectrum of *cis*-3l (100 MHz, $CDCl_3$)

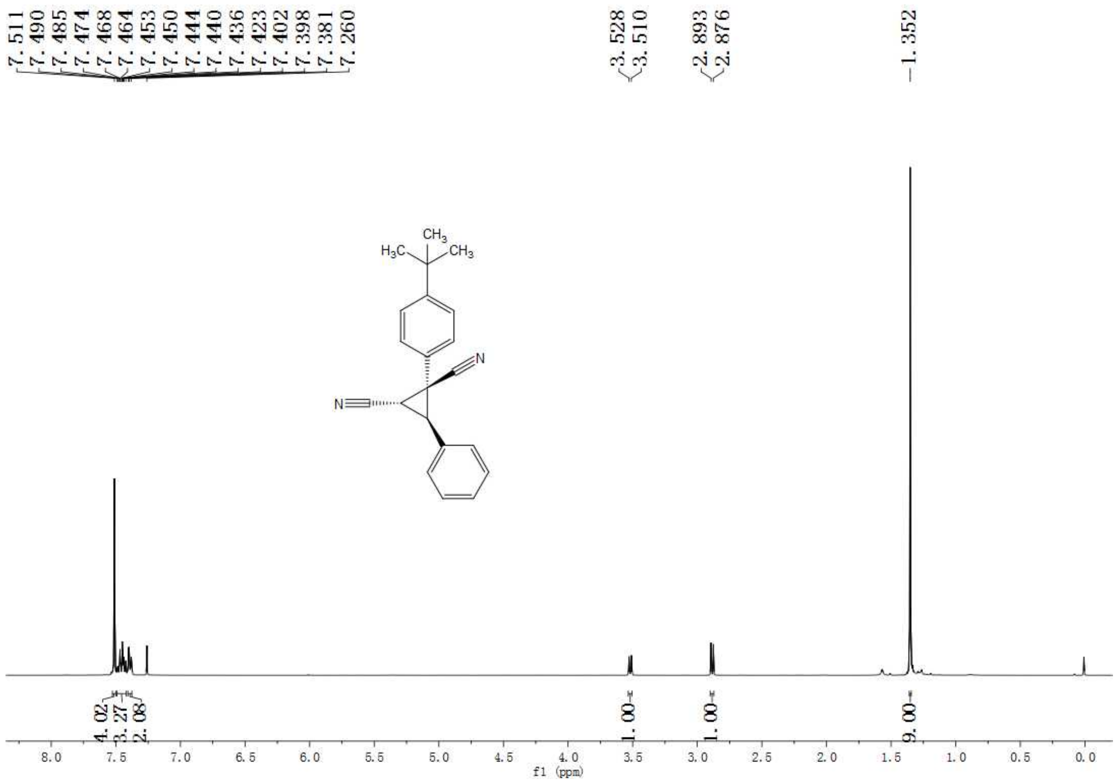

Figure S47. ^{1}H NMR Spectrum of *trans*-3l (400 MHz, $CDCl_3$)

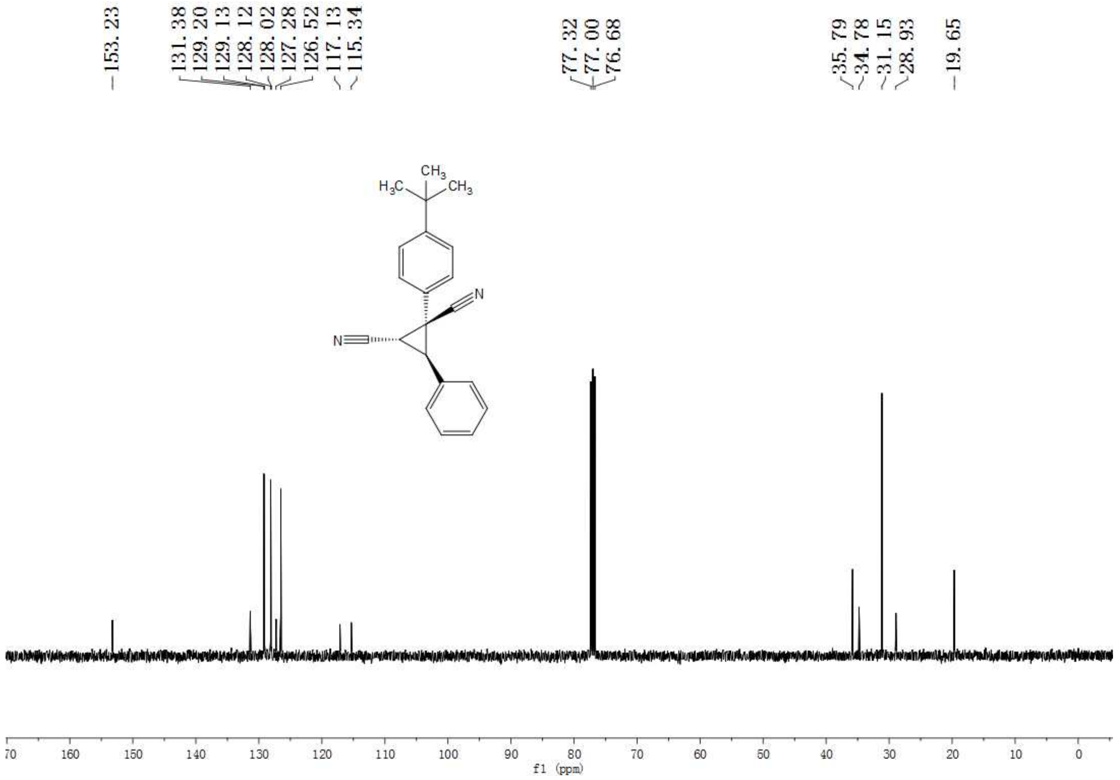

Figure S48. ^{13}C NMR Spectrum of *trans*-3l (100 MHz, $CDCl_3$)

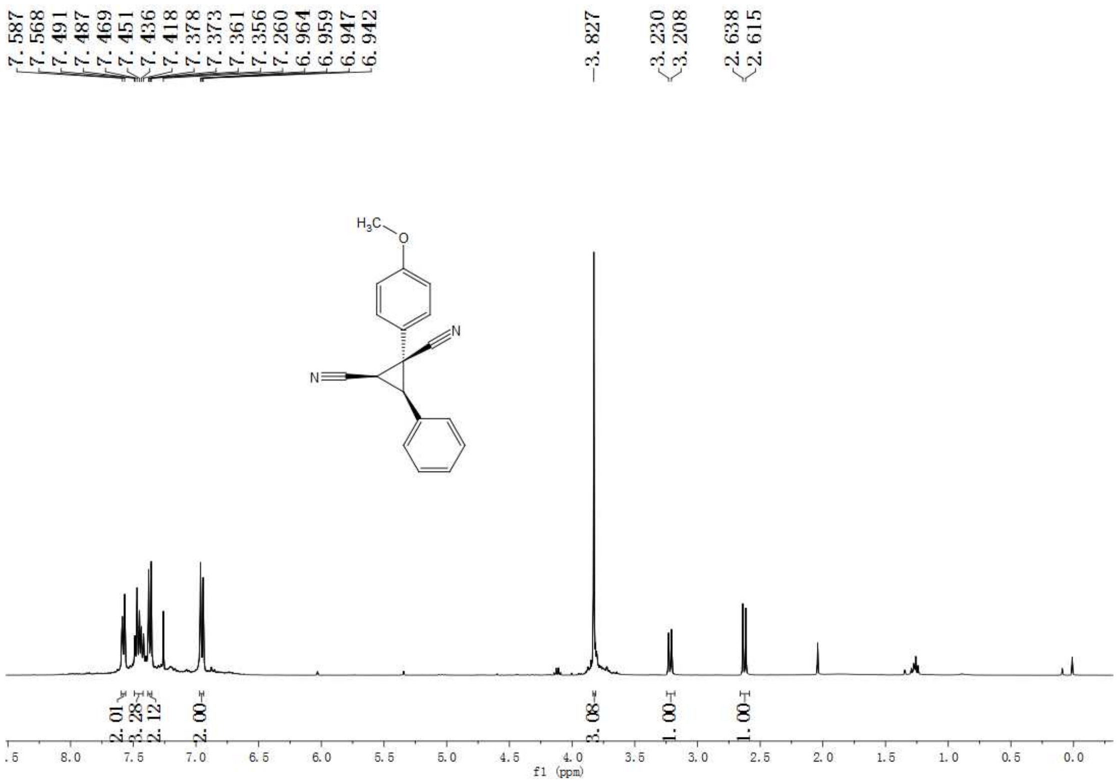

Figure S49. ^{1}H NMR Spectrum of *cis*-3m (400 MHz, $CDCl_3$)

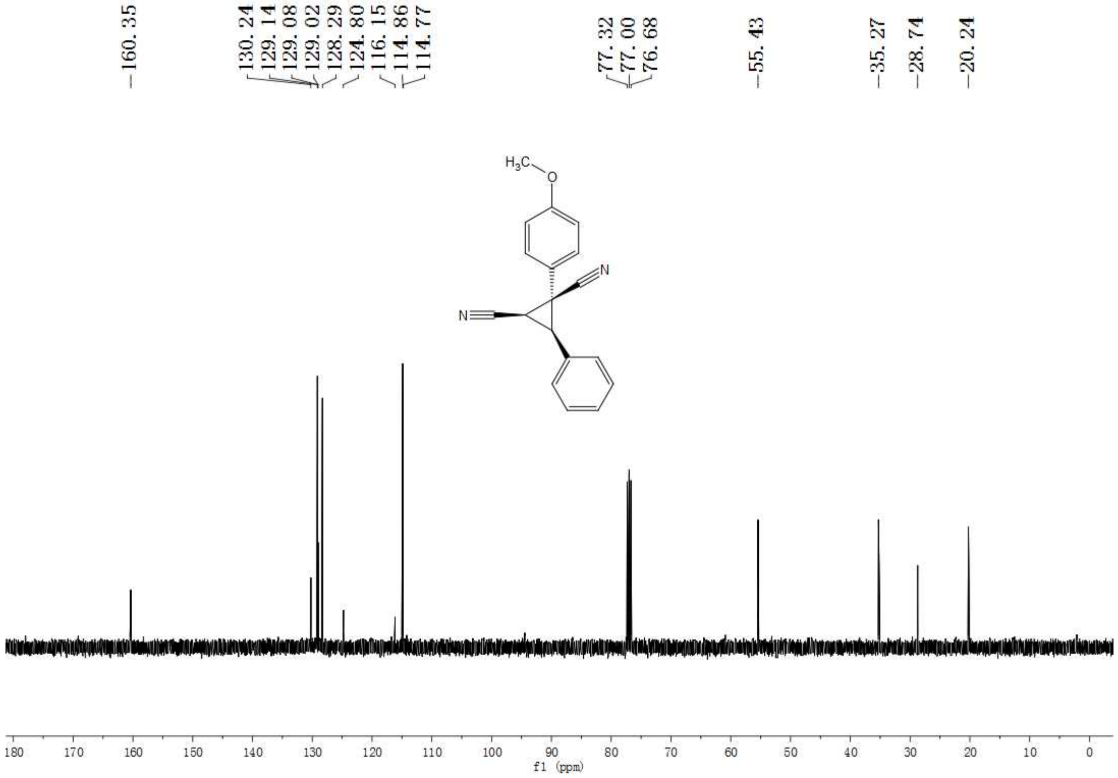

Figure S50. ^{13}C NMR Spectrum of *cis*-3m (100 MHz, $CDCl_3$)

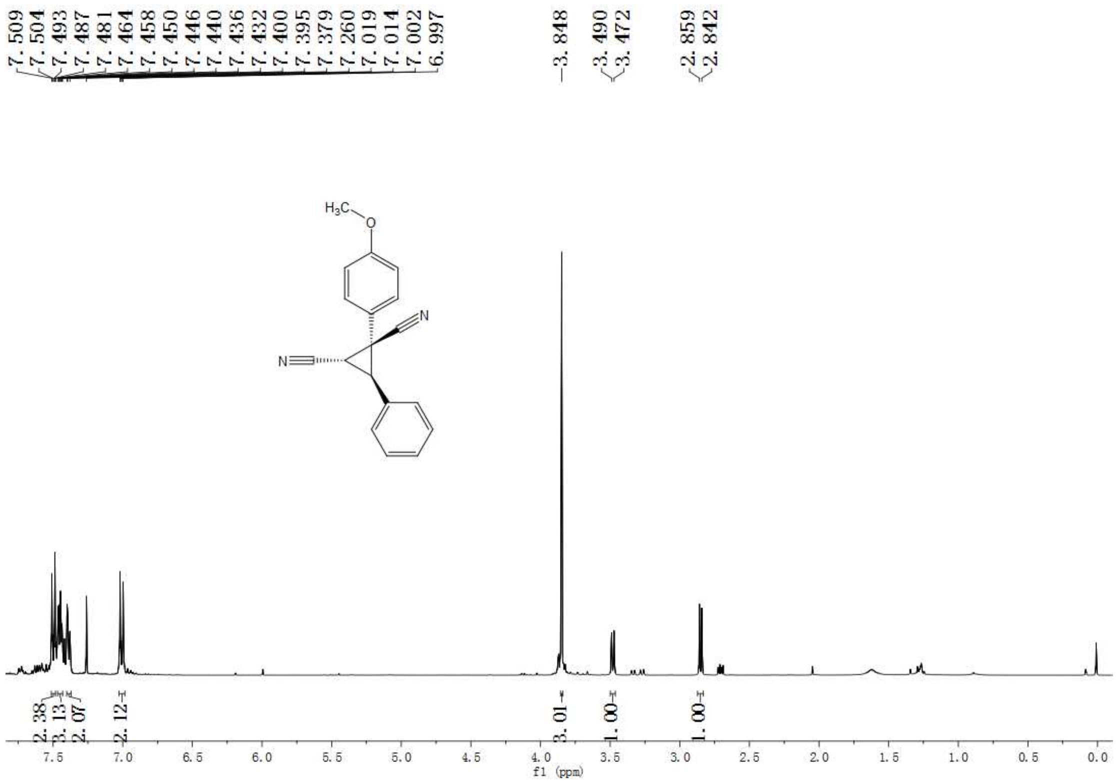

Figure S51. ^{1}**H NMR Spectrum of** ***trans*****-3m (400 MHz, CDCl**$_3$**)**

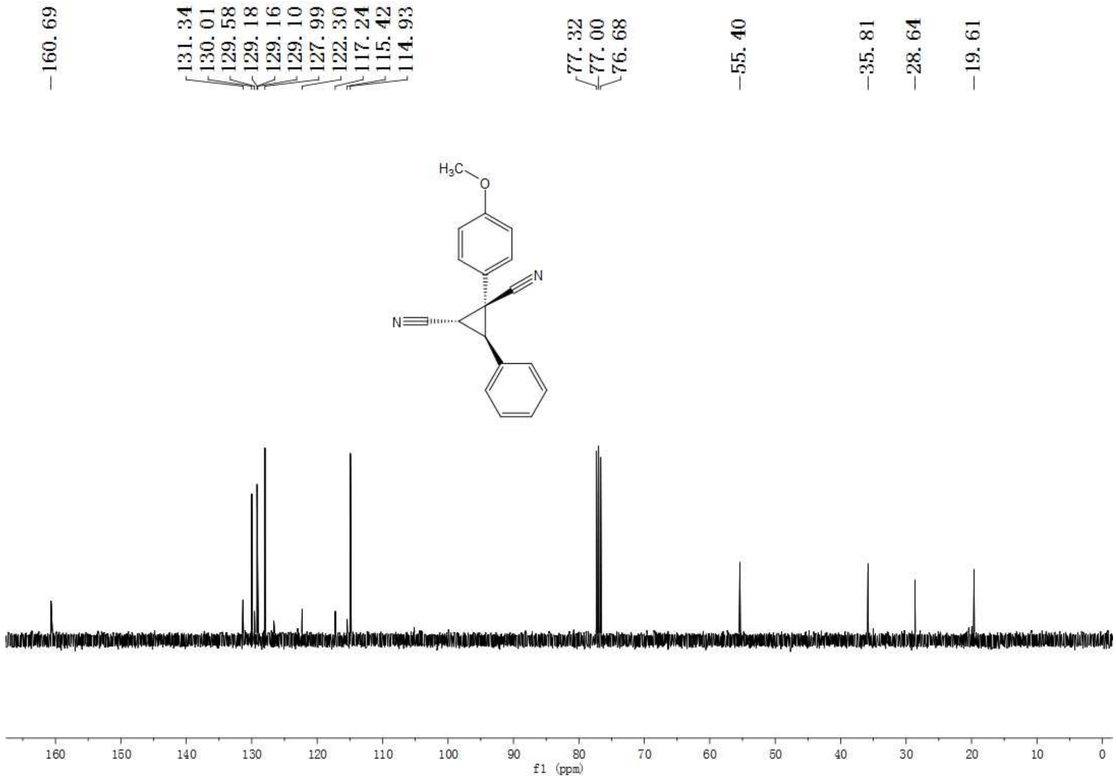

Figure S52. ^{13}**C NMR Spectrum of** ***trans*****-3m (100 MHz, CDCl**$_3$**)**

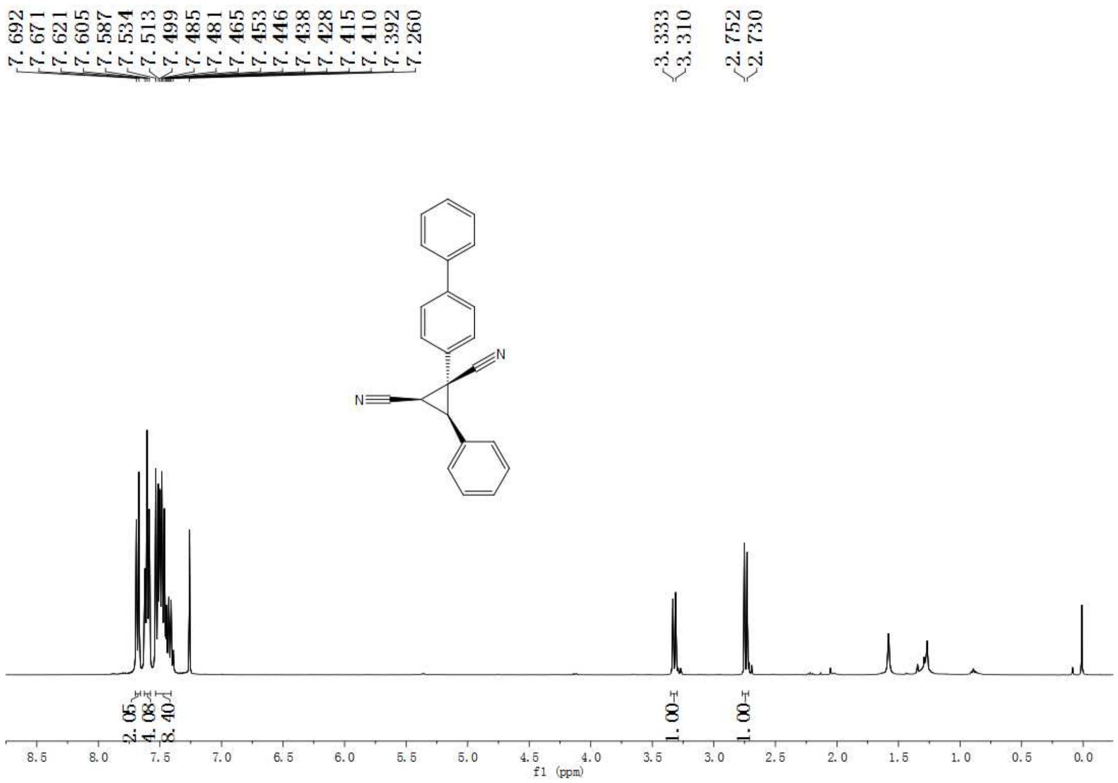

Figure S53. ^{1}H NMR Spectrum of *cis*-3n (400 MHz, $CDCl_3$)

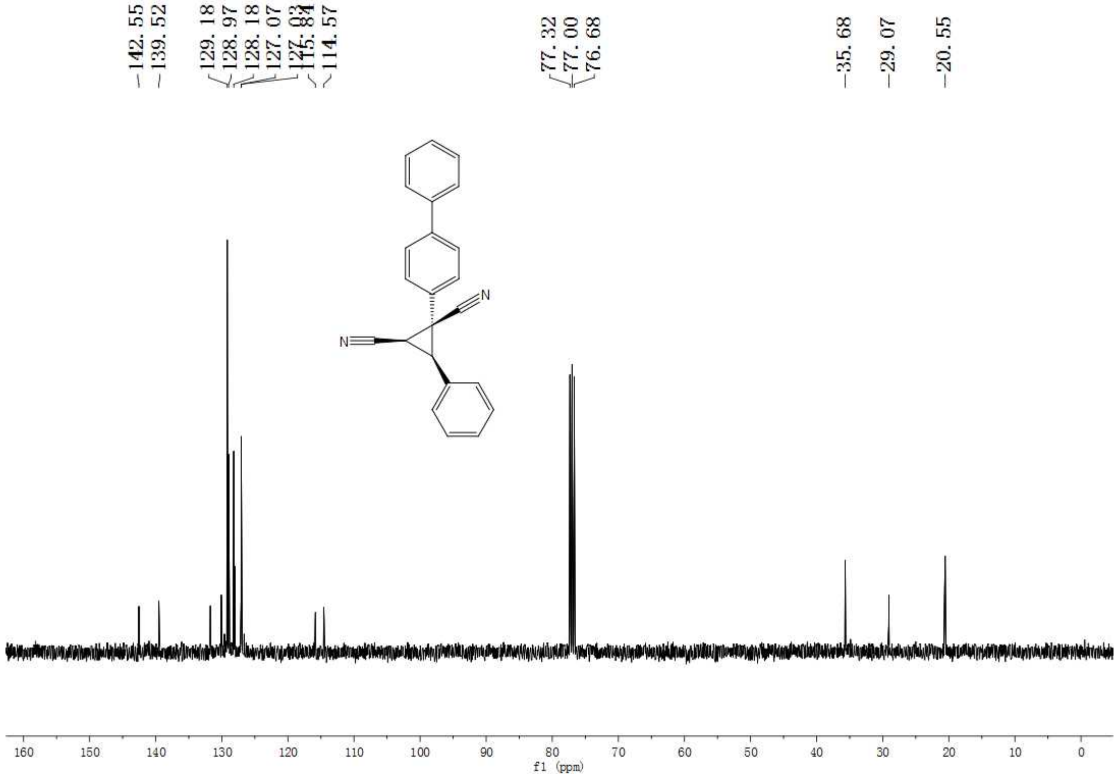

Figure S54. ^{13}C NMR Spectrum of *cis*-3n (100 MHz, $CDCl_3$)

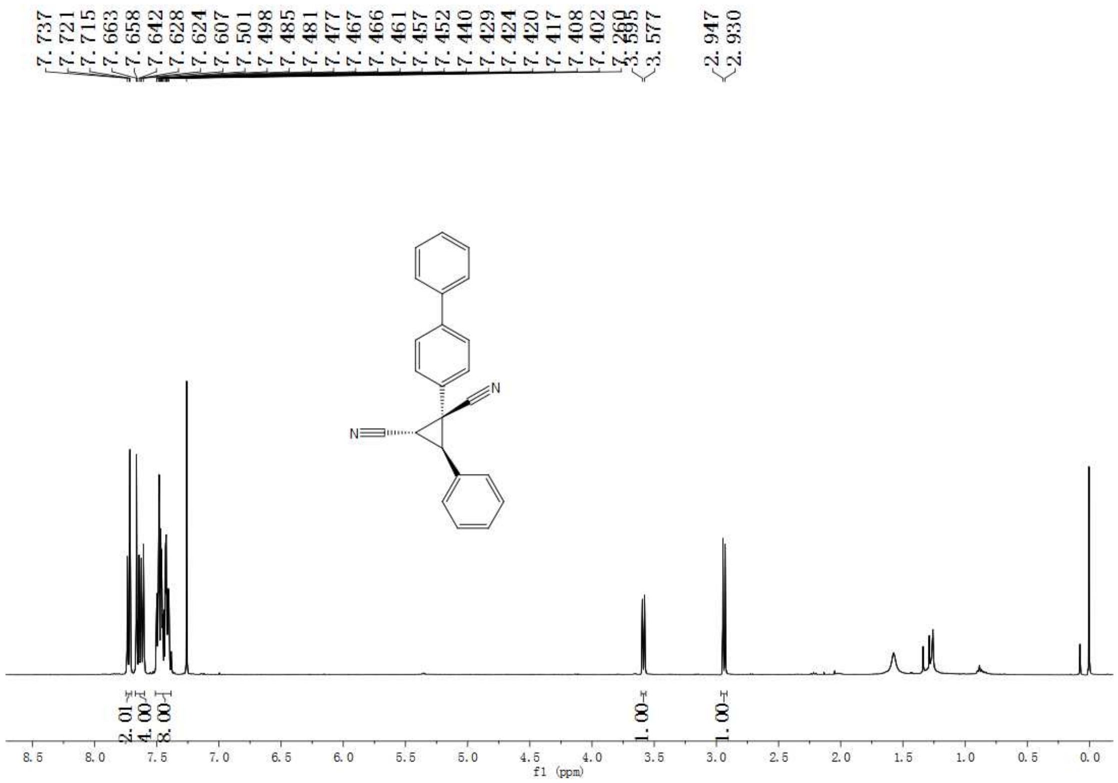

Figure S55. ^{1}H NMR Spectrum of *trans*-3n (400 MHz, $CDCl_3$)

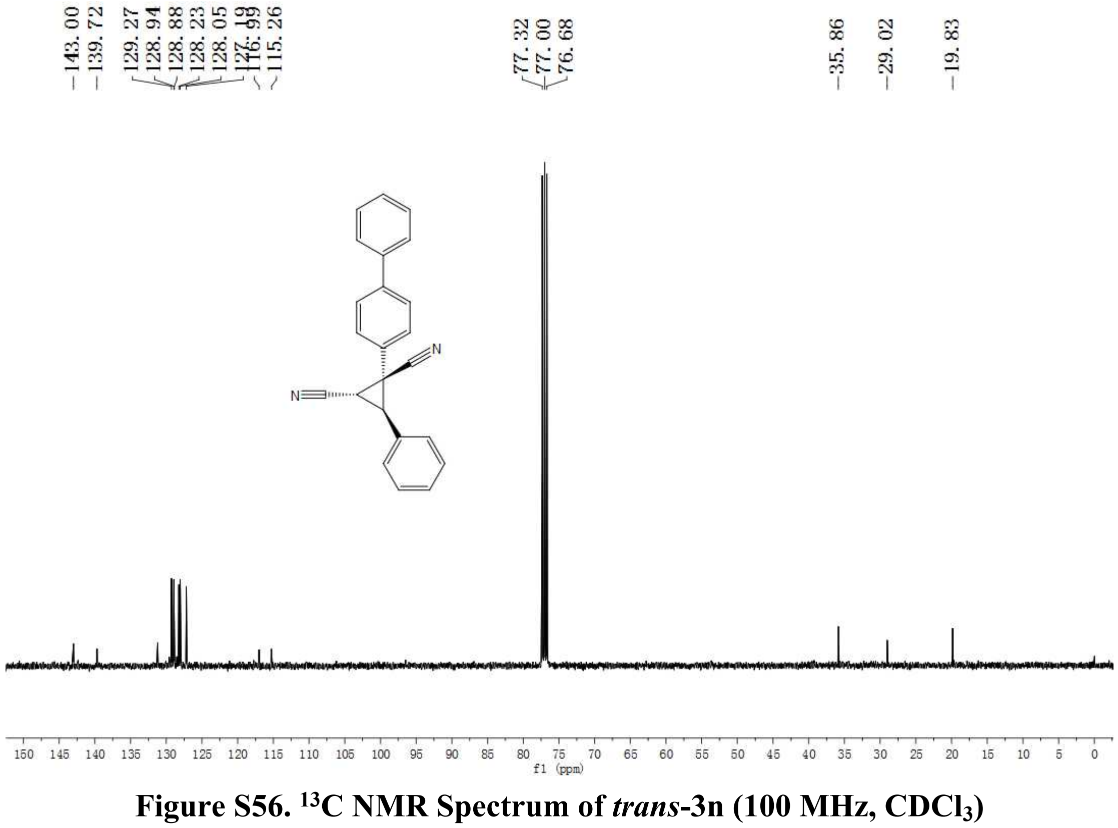

Figure S56. ^{13}C NMR Spectrum of *trans*-3n (100 MHz, $CDCl_3$)

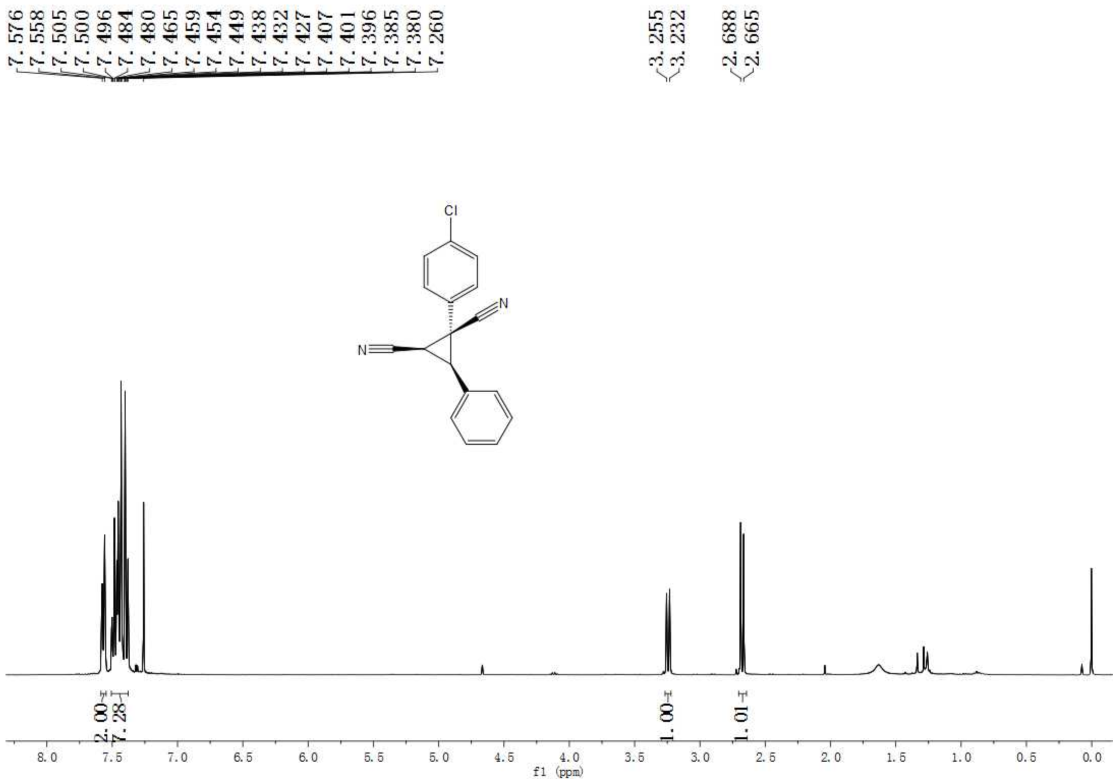

Figure S57. ^{1}H NMR Spectrum of *cis*-3o (400 MHz, $CDCl_3$)

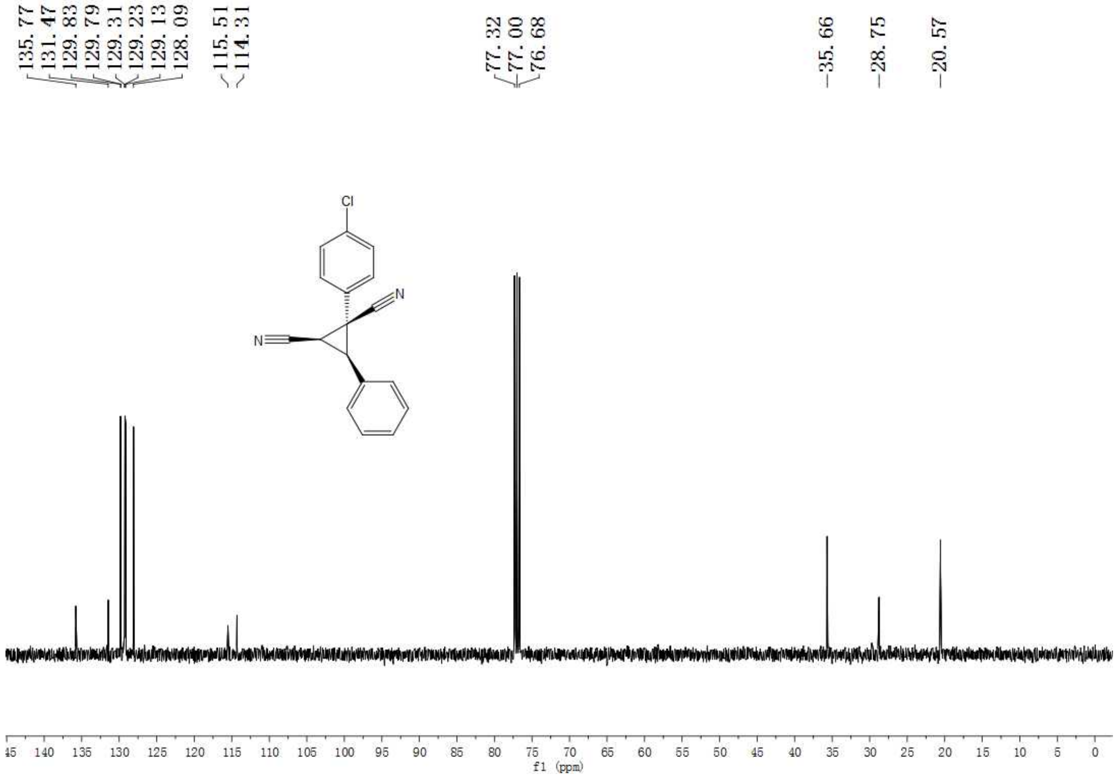

Figure S58. ^{13}C NMR Spectrum of *cis*-3o (100 MHz, $CDCl_3$)

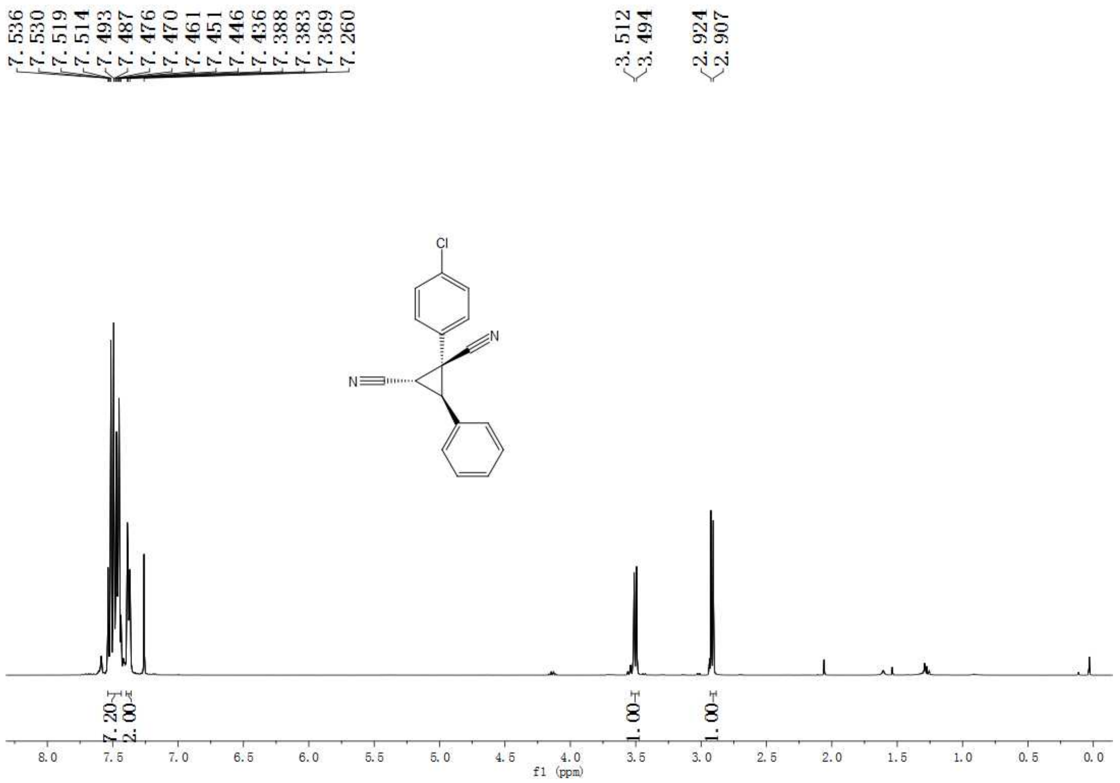

Figure S59. ^{1}H NMR Spectrum of *trans*-3o (400 MHz, $CDCl_3$)

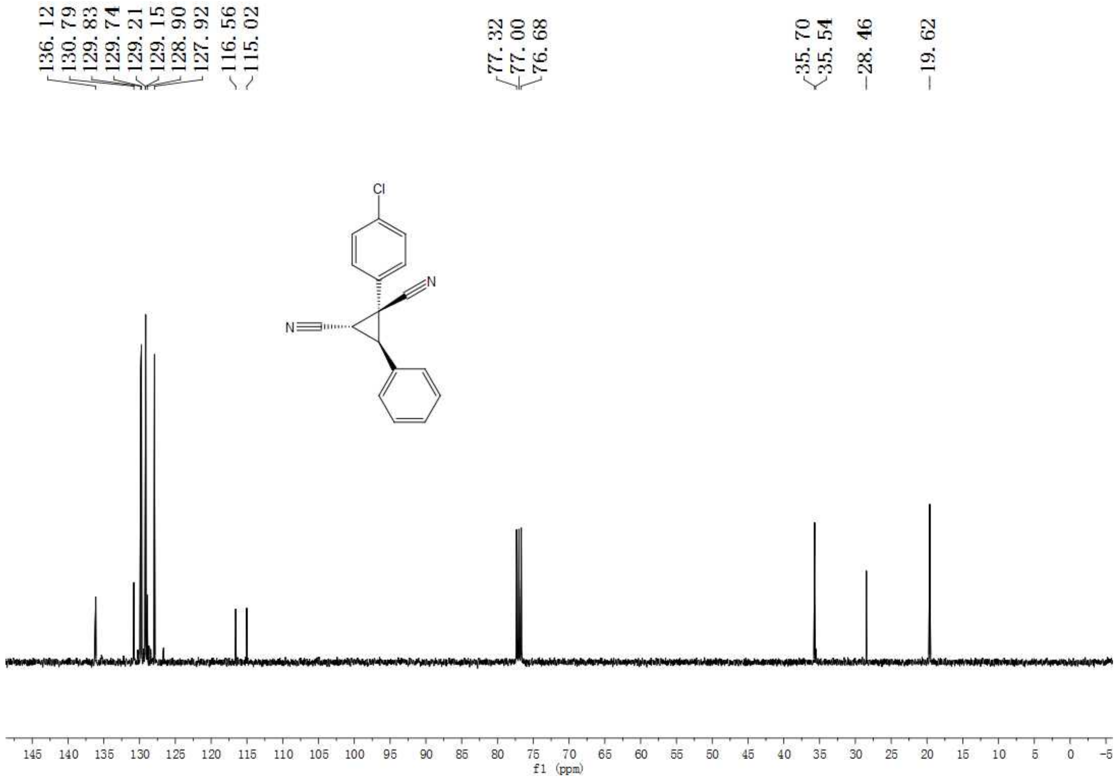

Figure S60. ^{13}C NMR Spectrum of *trans*-3o (100 MHz, $CDCl_3$)

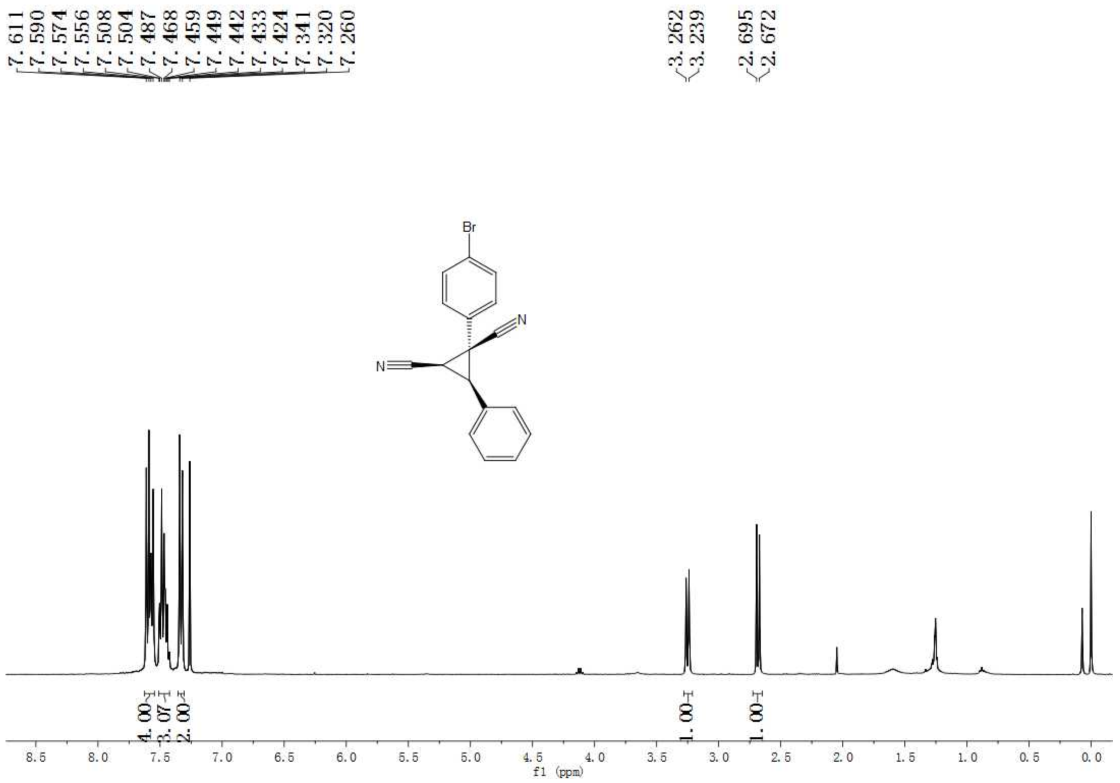

Figure S61. ^{1}H NMR Spectrum of *cis*-3p (400 MHz, $CDCl_3$)

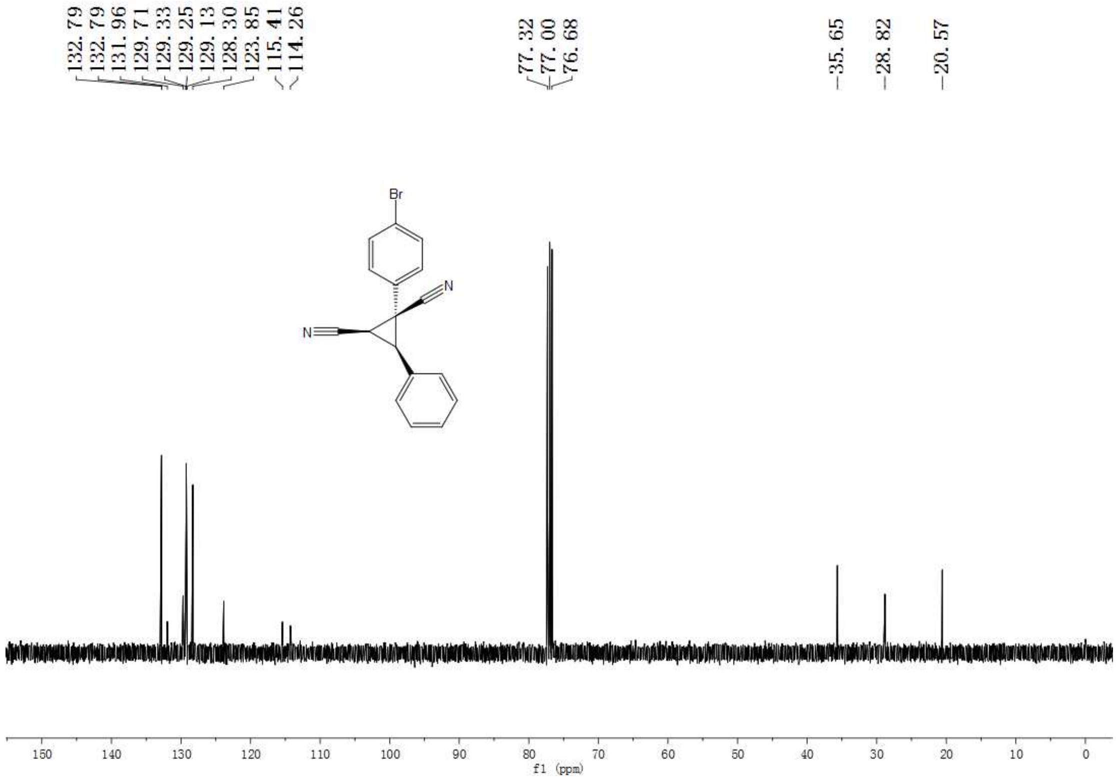

Figure S62. ^{13}C NMR Spectrum of *cis*-3p (100 MHz, $CDCl_3$)

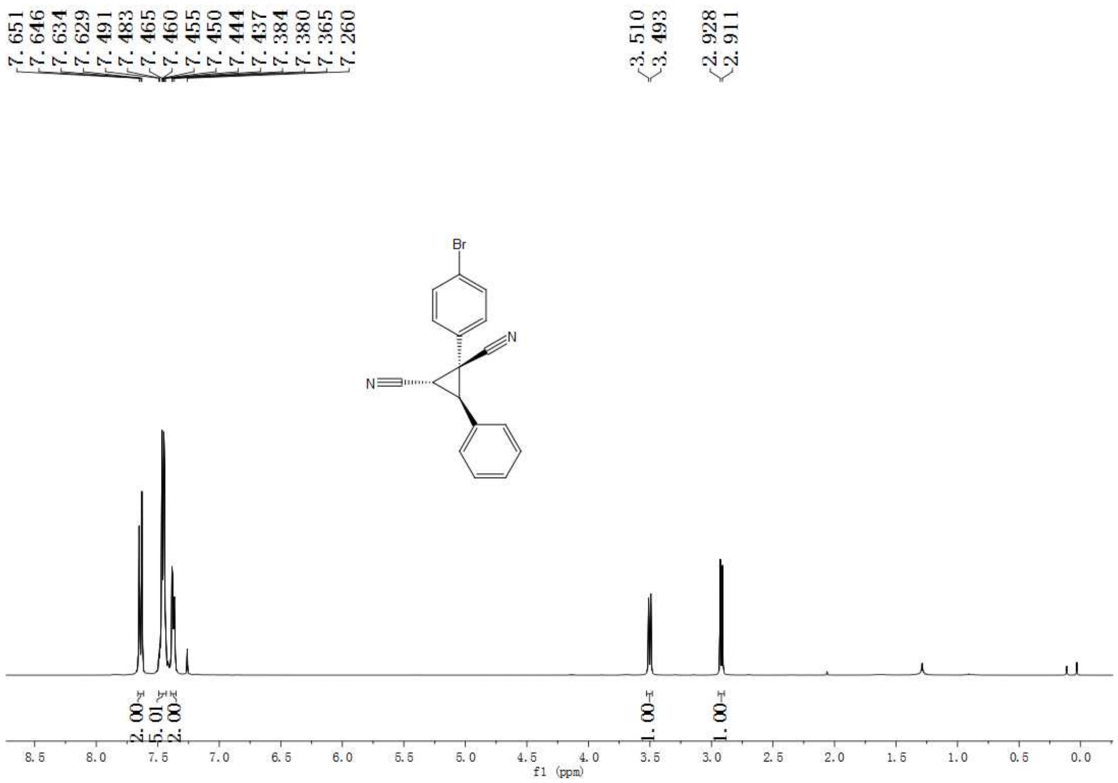

Figure S63. ^{1}H NMR Spectrum of *trans*-3p (400 MHz, $CDCl_3$)

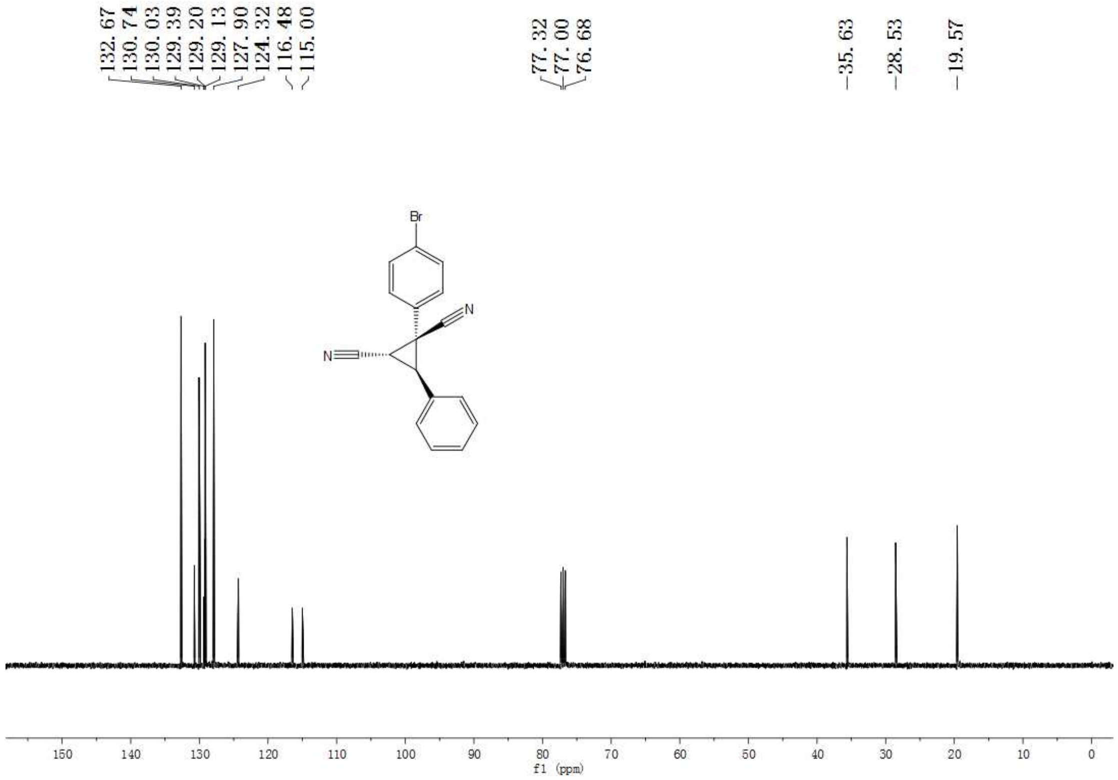

Figure S64. ^{13}C NMR Spectrum of *trans*-3p (100 MHz, $CDCl_3$)

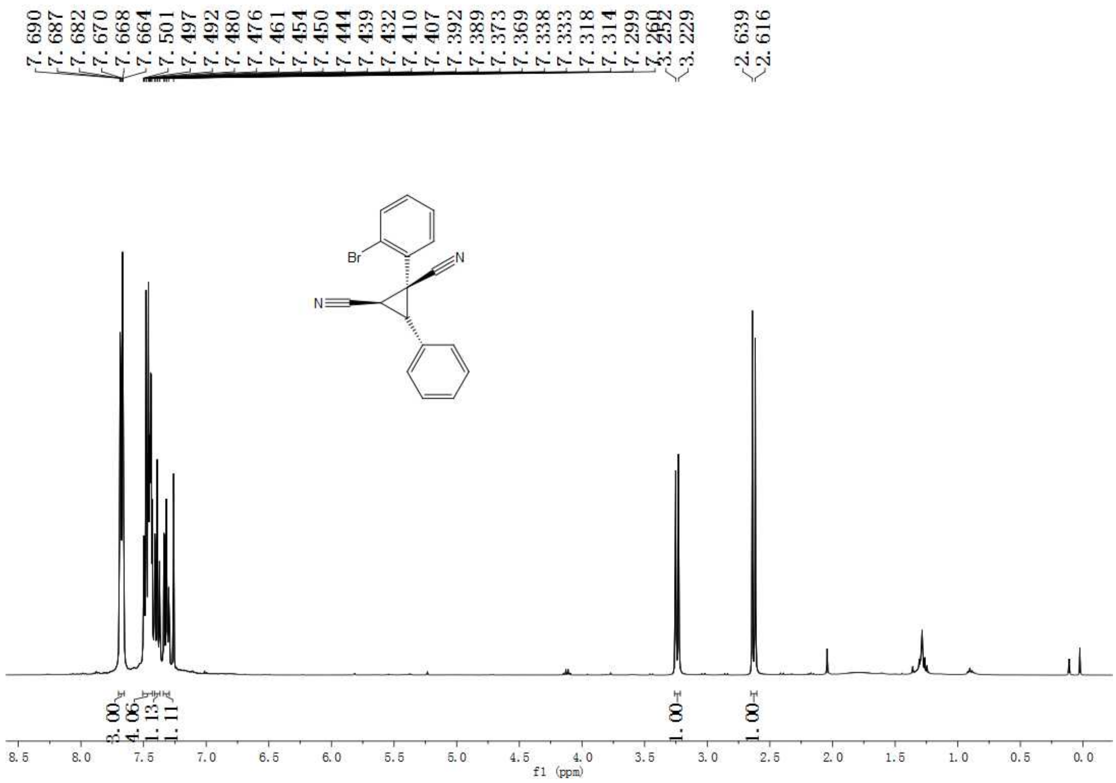

Figure S65. ^{1}H NMR Spectrum of *cis*-3q (400 MHz, $CDCl_3$)

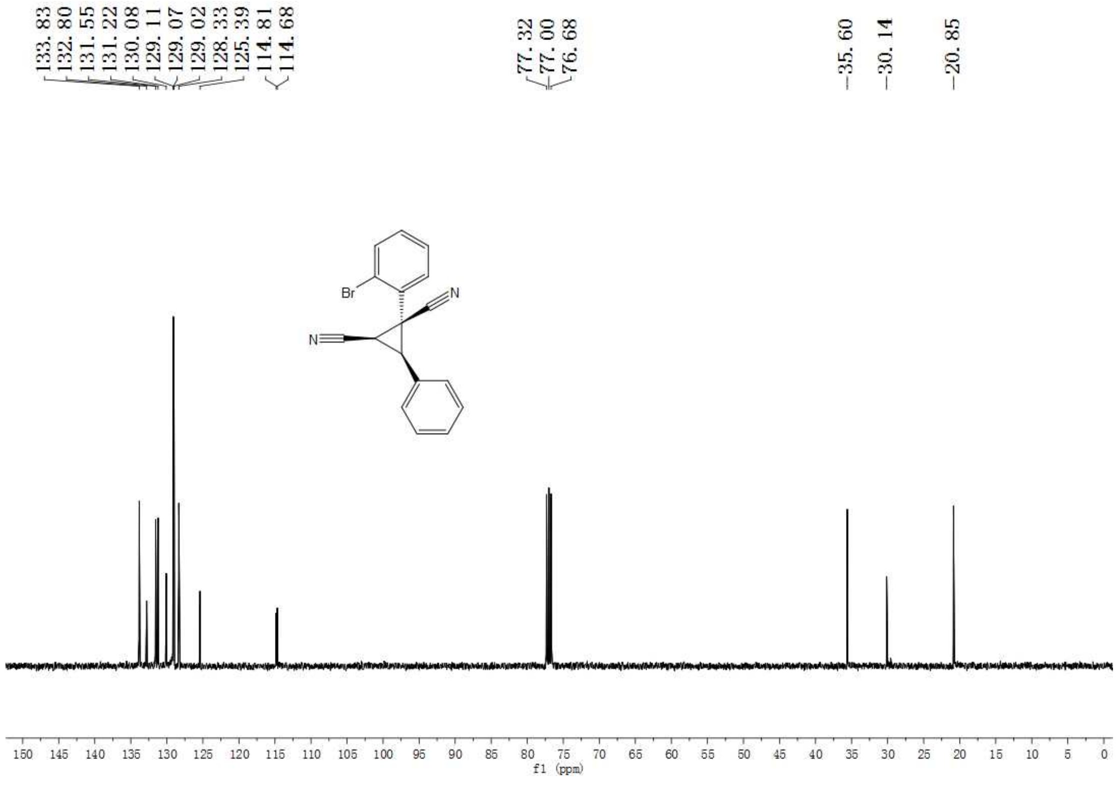

Figure S66. ^{13}C NMR Spectrum of *cis*-3q (100 MHz, $CDCl_3$)

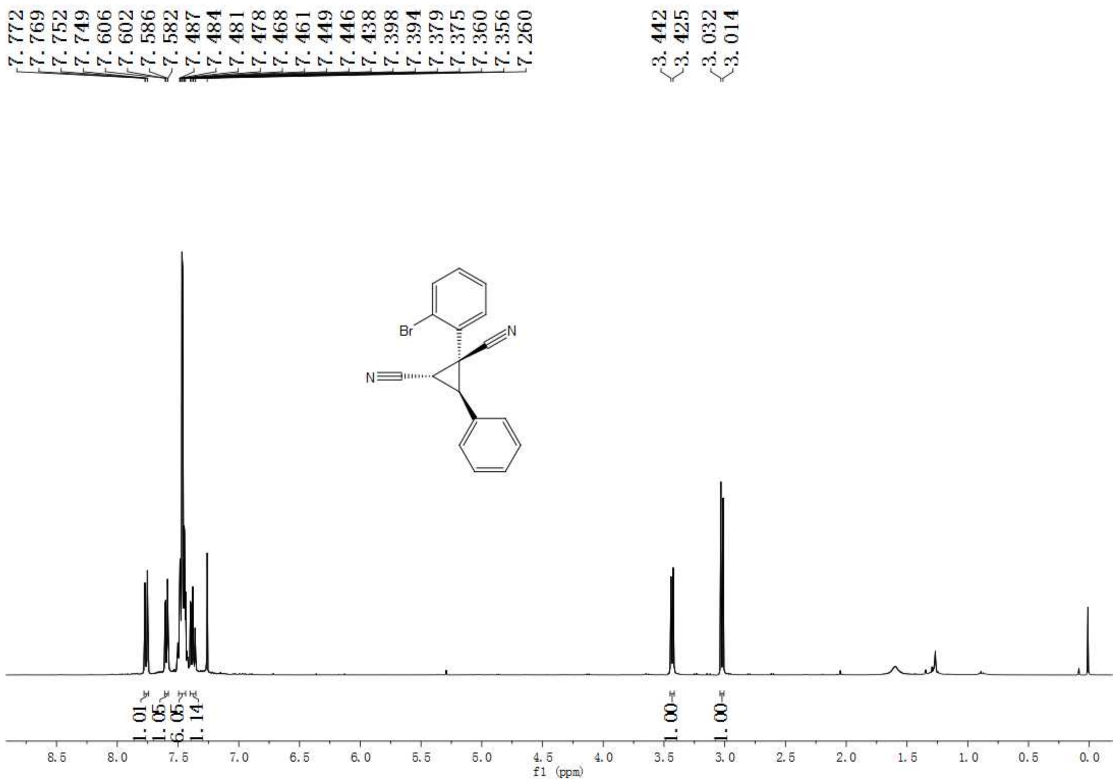

Figure S67. ^{1}H NMR Spectrum of *trans*-3q (400 MHz, $CDCl_3$)

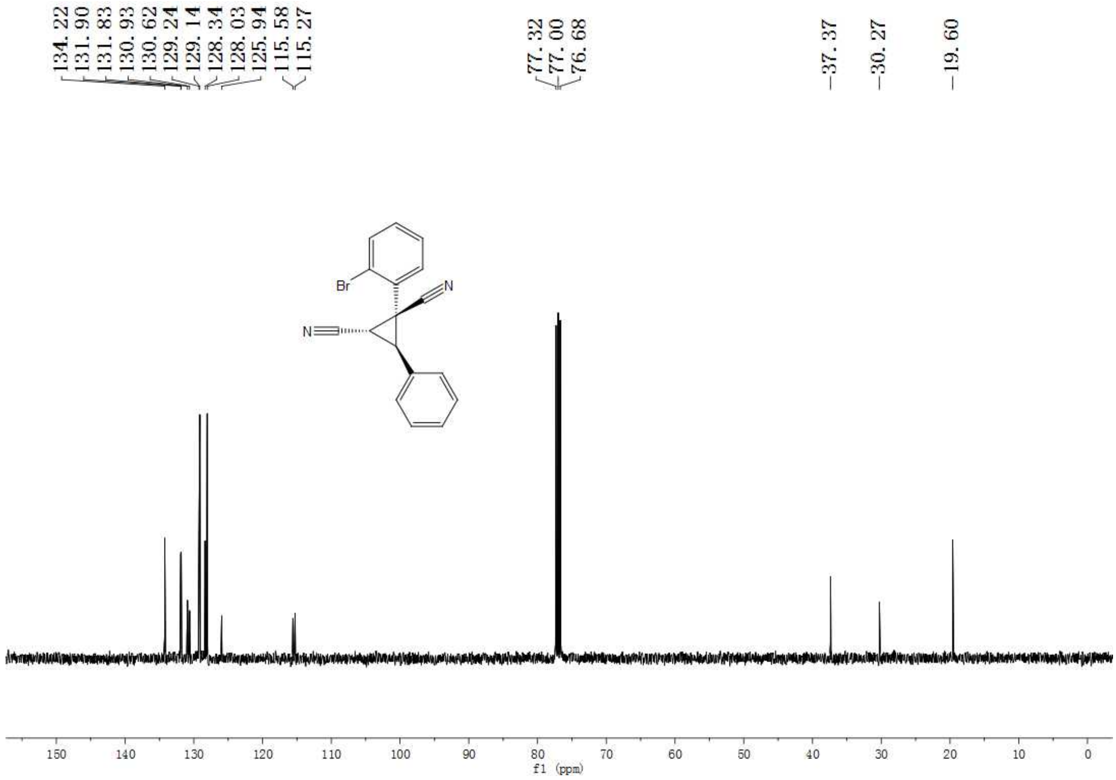

Figure S68. ^{13}C NMR Spectrum of *trans*-3q (100 MHz, $CDCl_3$)

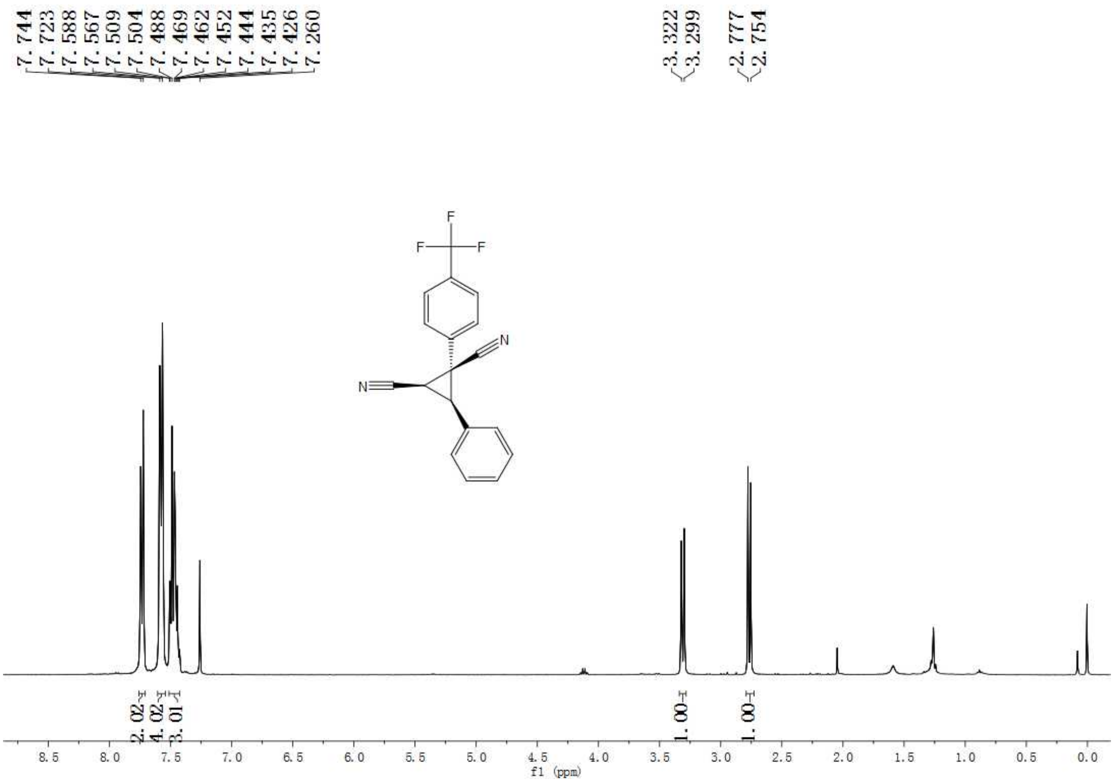

Figure S69. ^{1}H NMR Spectrum of *cis*-3r (400 MHz, $CDCl_3$)

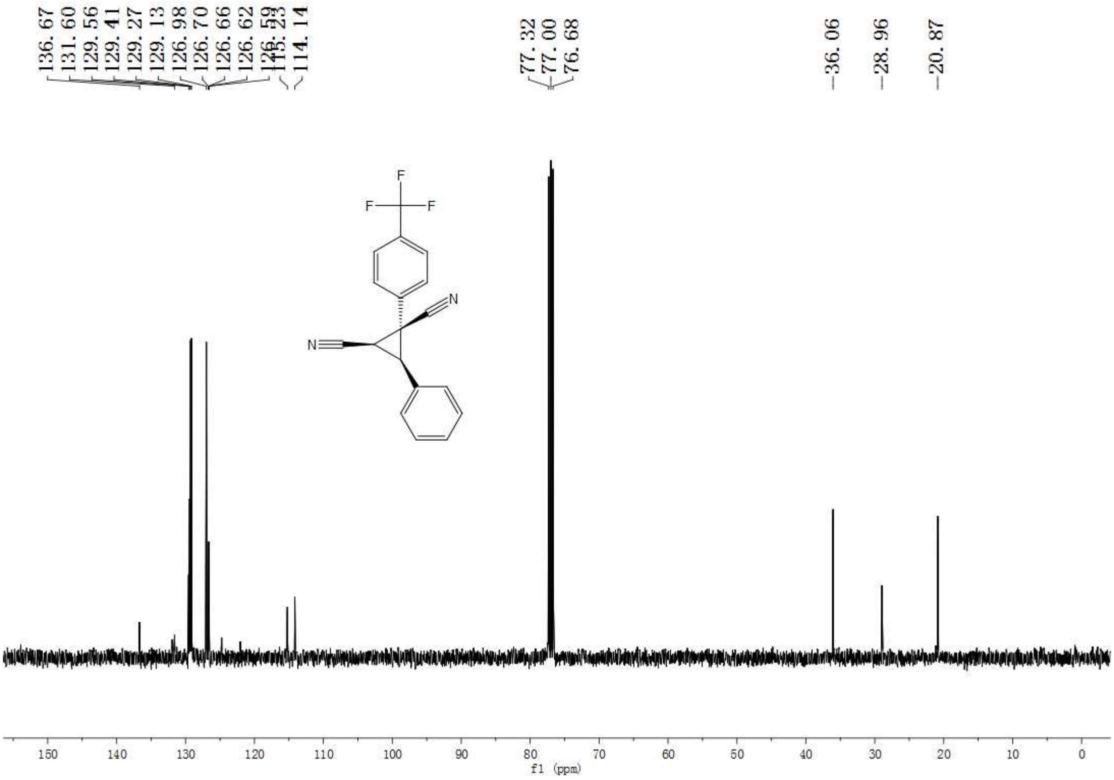

Figure S70. ^{13}C NMR Spectrum of *cis*-3r (100 MHz, $CDCl_3$)

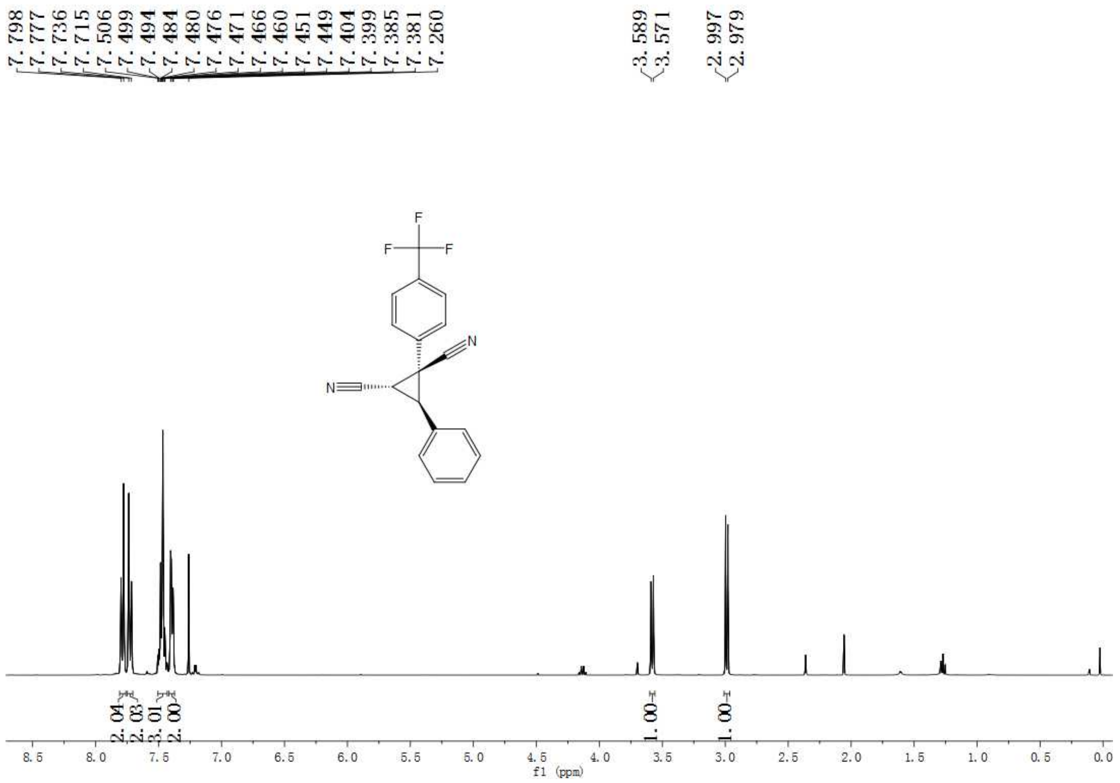

Figure S71. ^{1}H NMR Spectrum of *trans*-3r (400 MHz, $CDCl_3$)

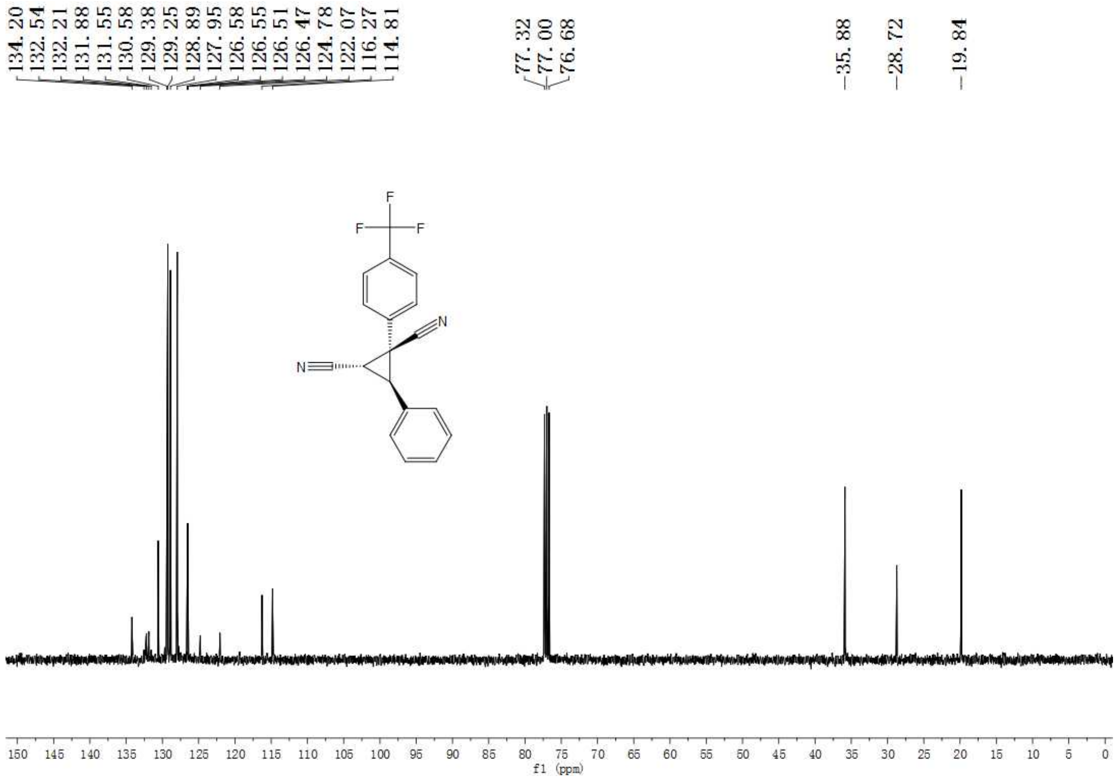

Figure S72. ^{13}C NMR Spectrum of *trans*-3r (100 MHz, $CDCl_3$)

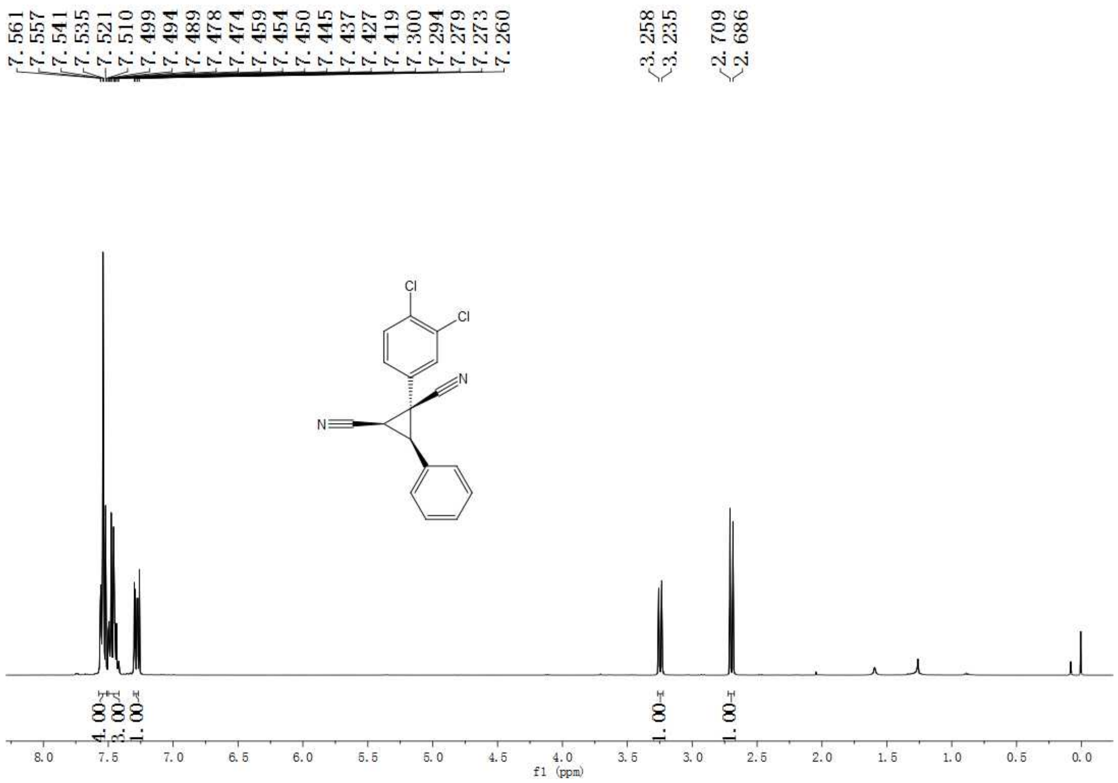

Figure S73. ^{1}H NMR Spectrum of *cis*-3s (400 MHz, $CDCl_3$)

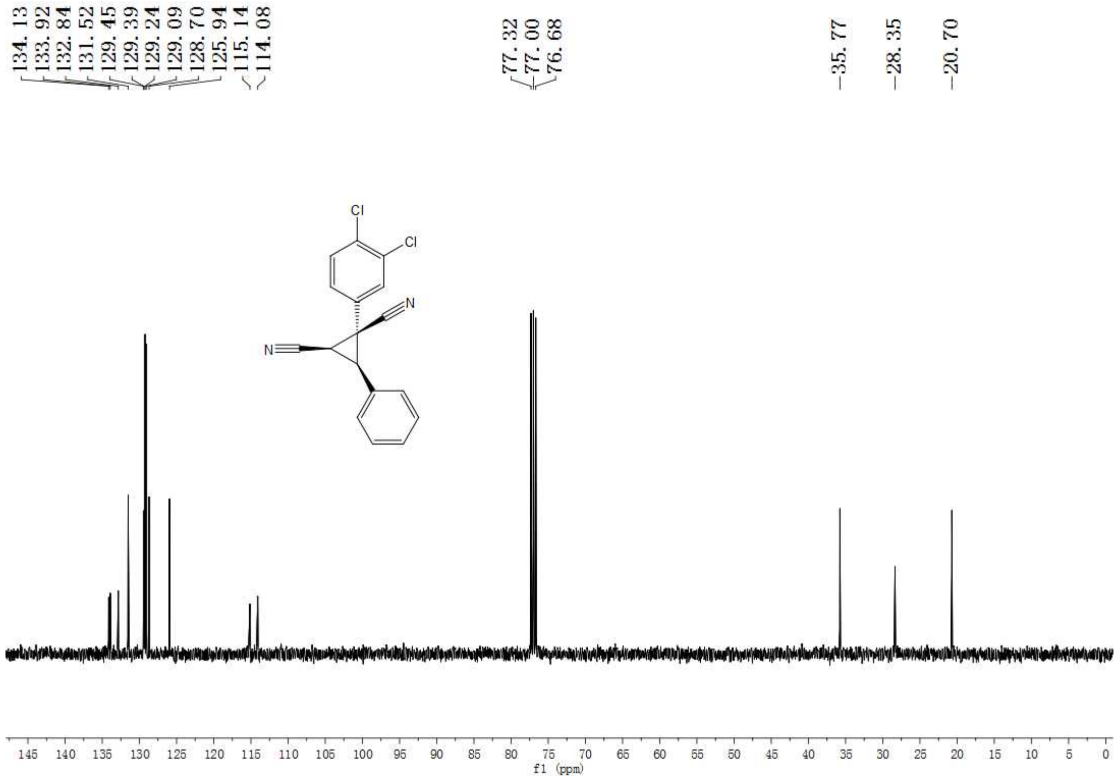

Figure S74. ^{13}C NMR Spectrum of *cis*-3s (100 MHz, $CDCl_3$)

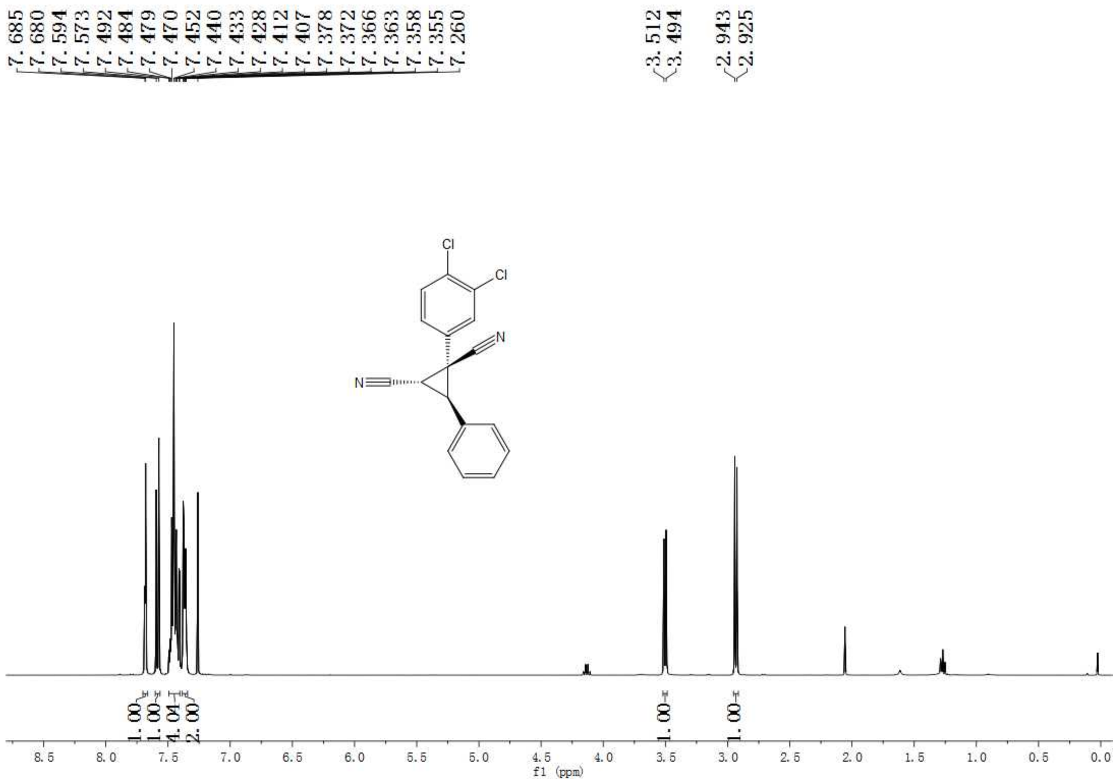

Figure S75. ^{1}H NMR Spectrum of *trans*-3s (400 MHz, $CDCl_3$)

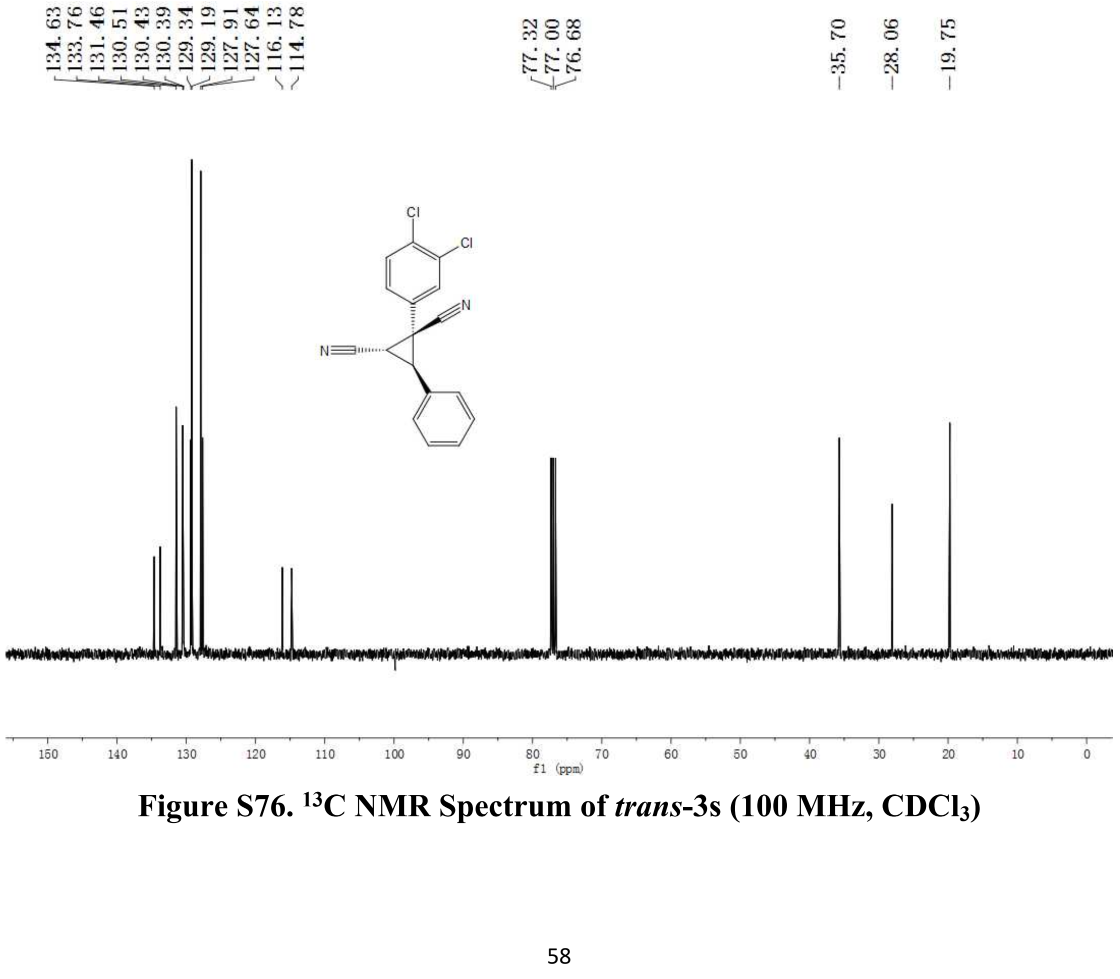

Figure S76. ^{13}C NMR Spectrum of *trans*-3s (100 MHz, $CDCl_3$)

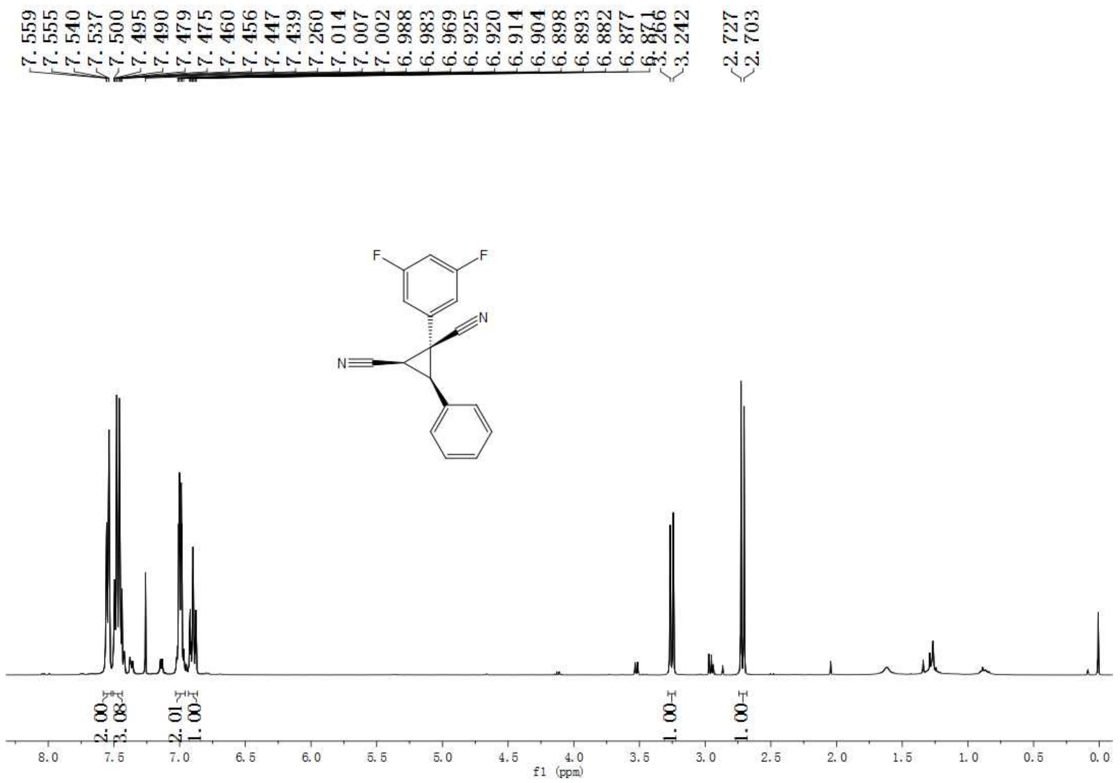

Figure S77. ^{1}H NMR Spectrum of *cis*-3t (400 MHz, $CDCl_3$)

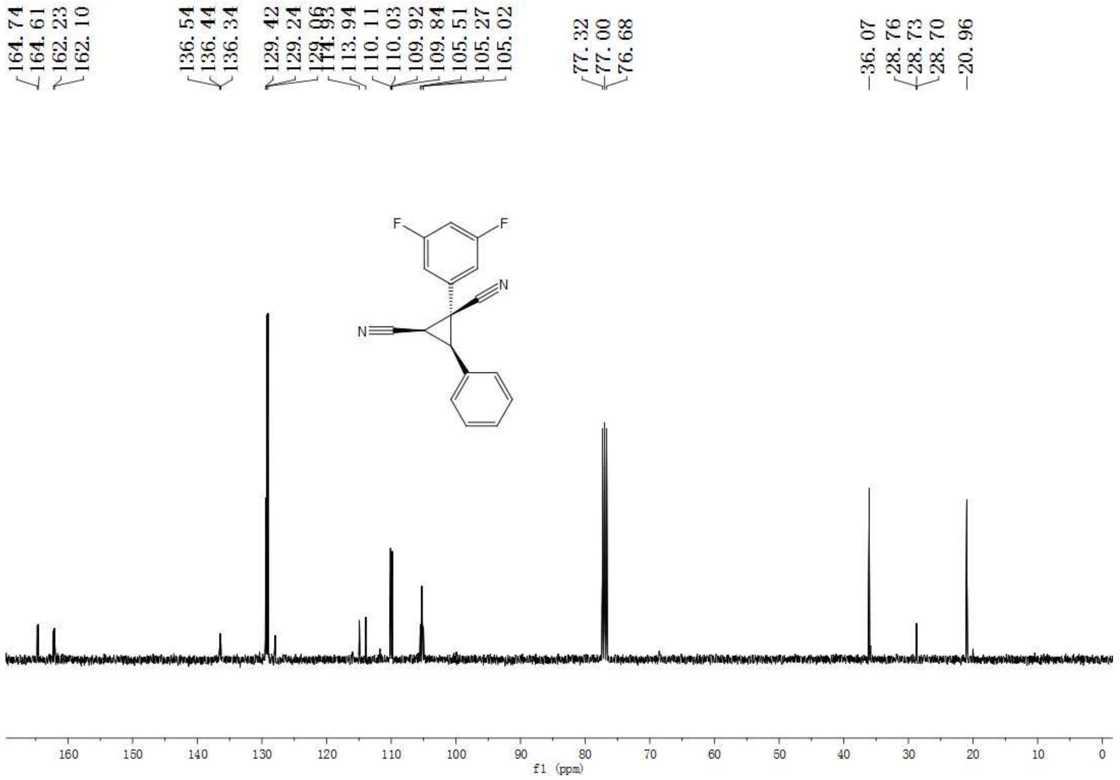

Figure S78. ^{13}C NMR Spectrum of *cis*-3t (100 MHz, $CDCl_3$)

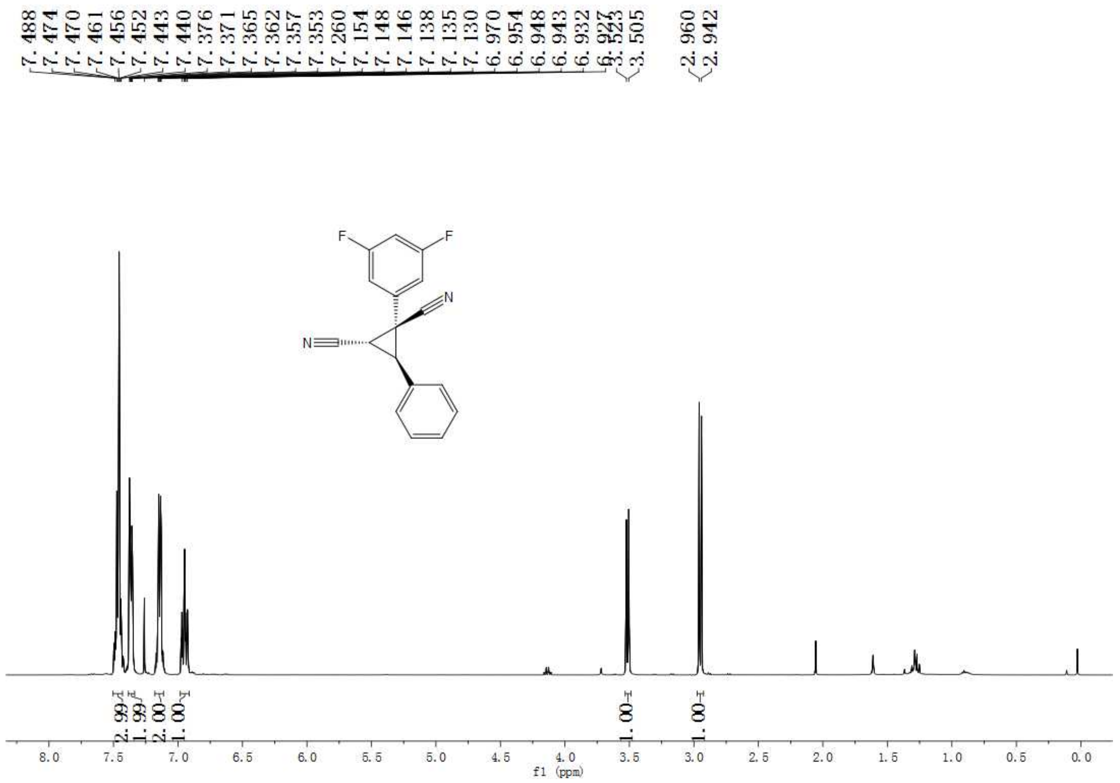

Figure S79. ^{1}H NMR Spectrum of *trans*-3t (400 MHz, $CDCl_3$)

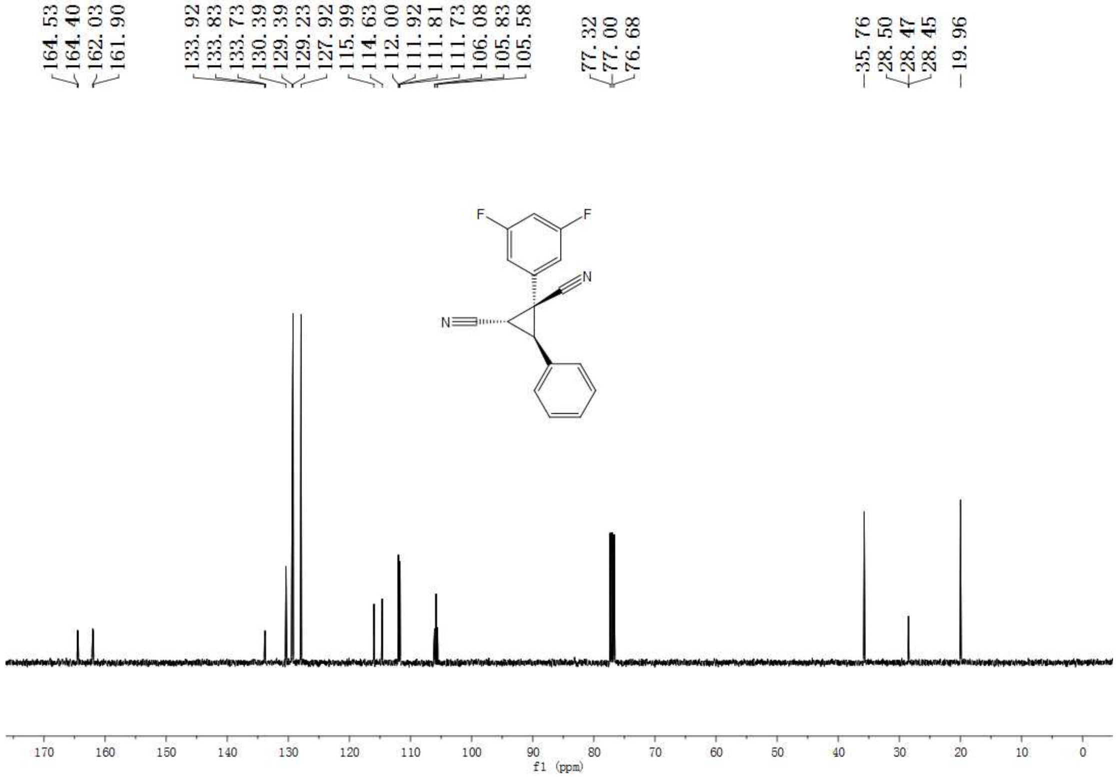

Figure S80. ^{13}C NMR Spectrum of *trans*-3t (100 MHz, $CDCl_3$)

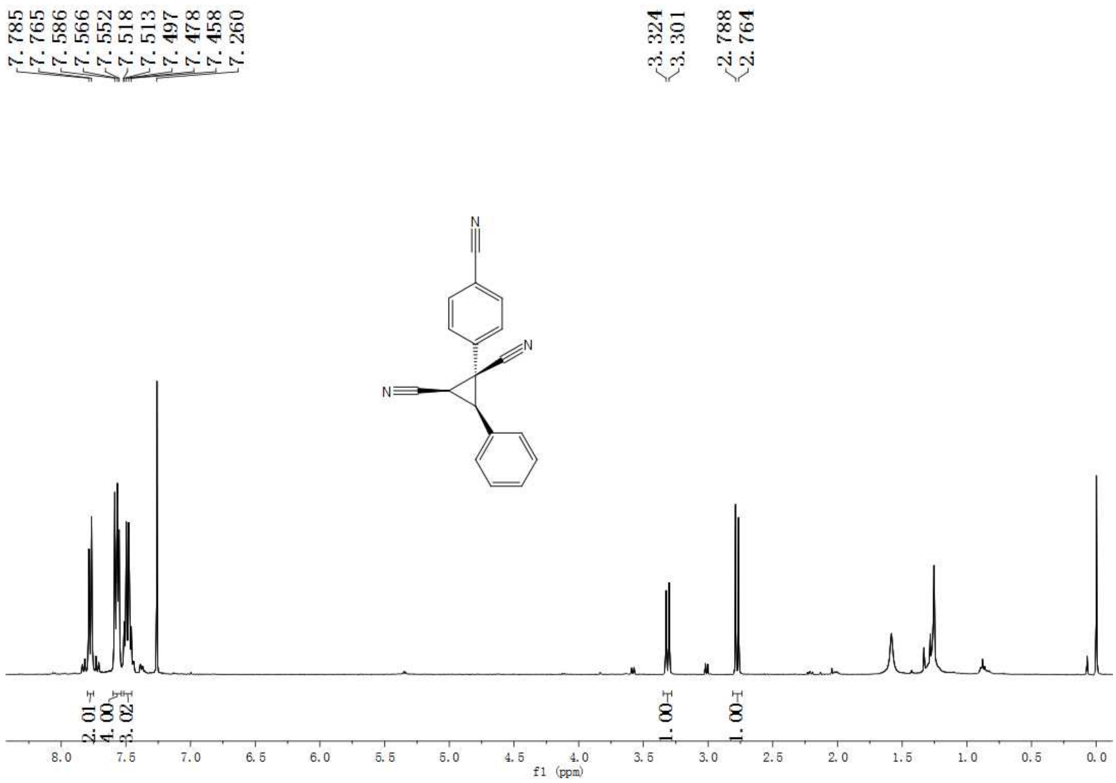

Figure S81. ¹H NMR Spectrum of *cis*-3u (400 MHz, CDCl$_3$)

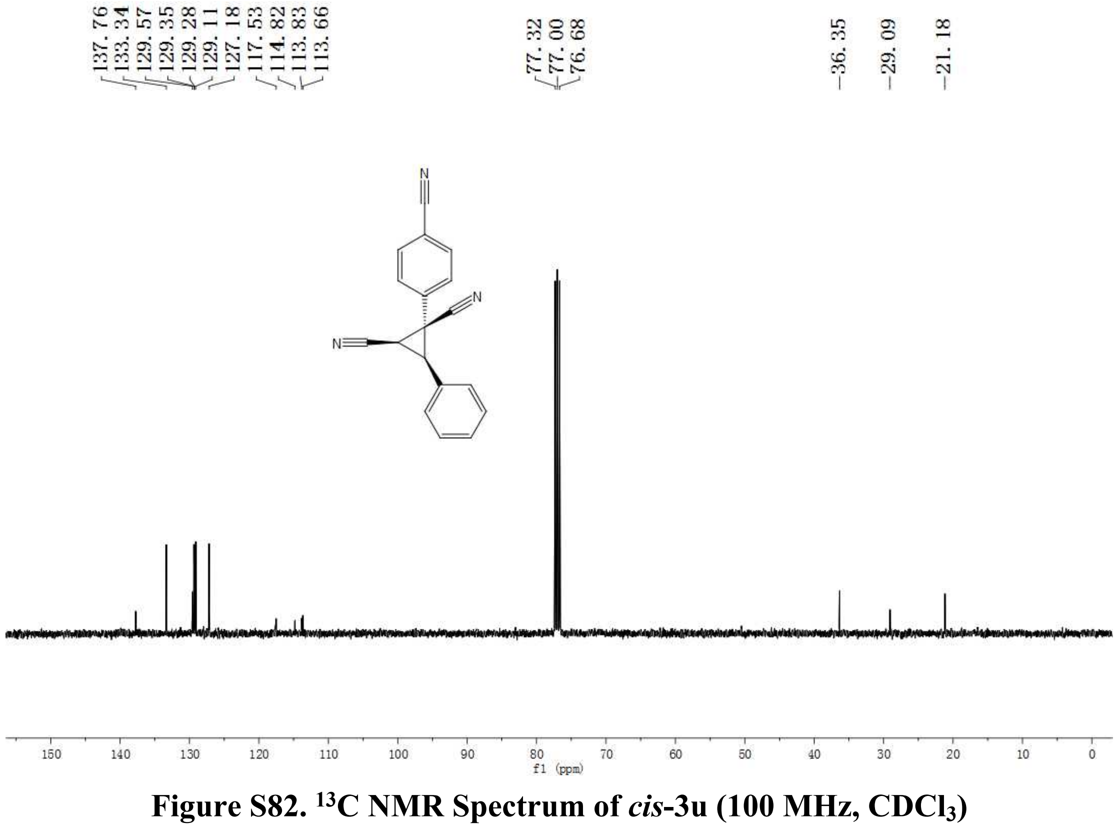

Figure S82. ¹³C NMR Spectrum of *cis*-3u (100 MHz, CDCl$_3$)

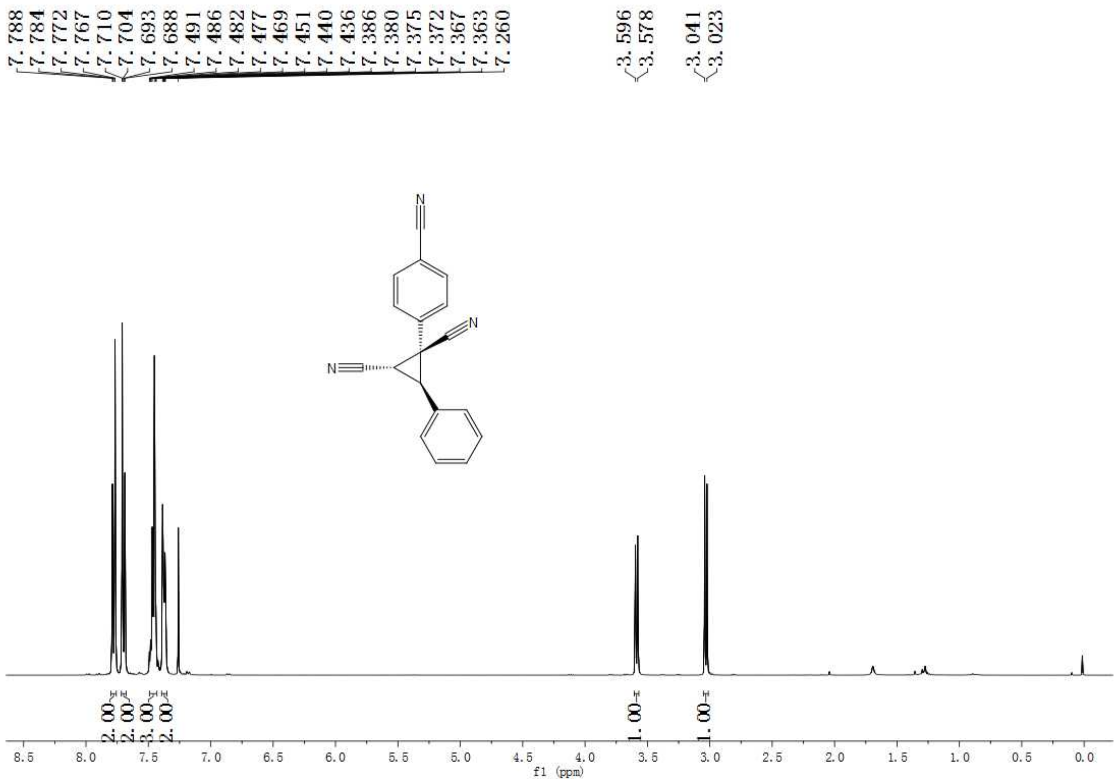

Figure S83. ^{1}H NMR Spectrum of *trans*-3u (400 MHz, $CDCl_3$)

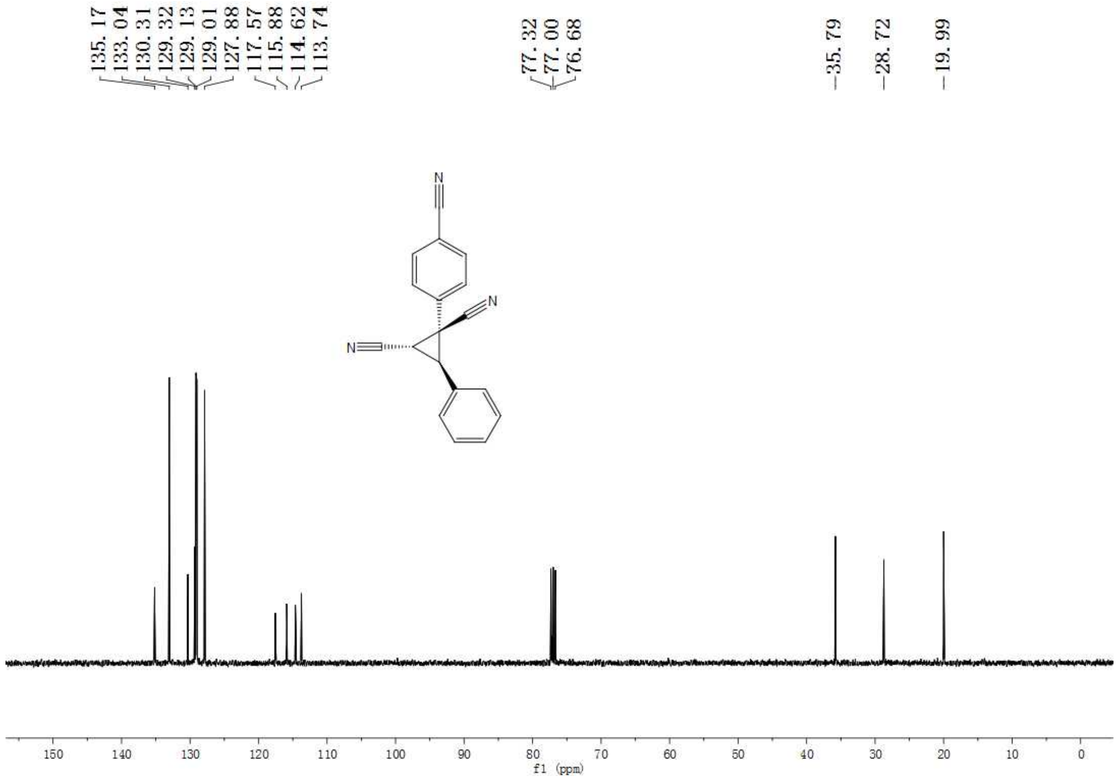

Figure S84. ^{13}C NMR Spectrum of *trans*-3u (100 MHz, $CDCl_3$)

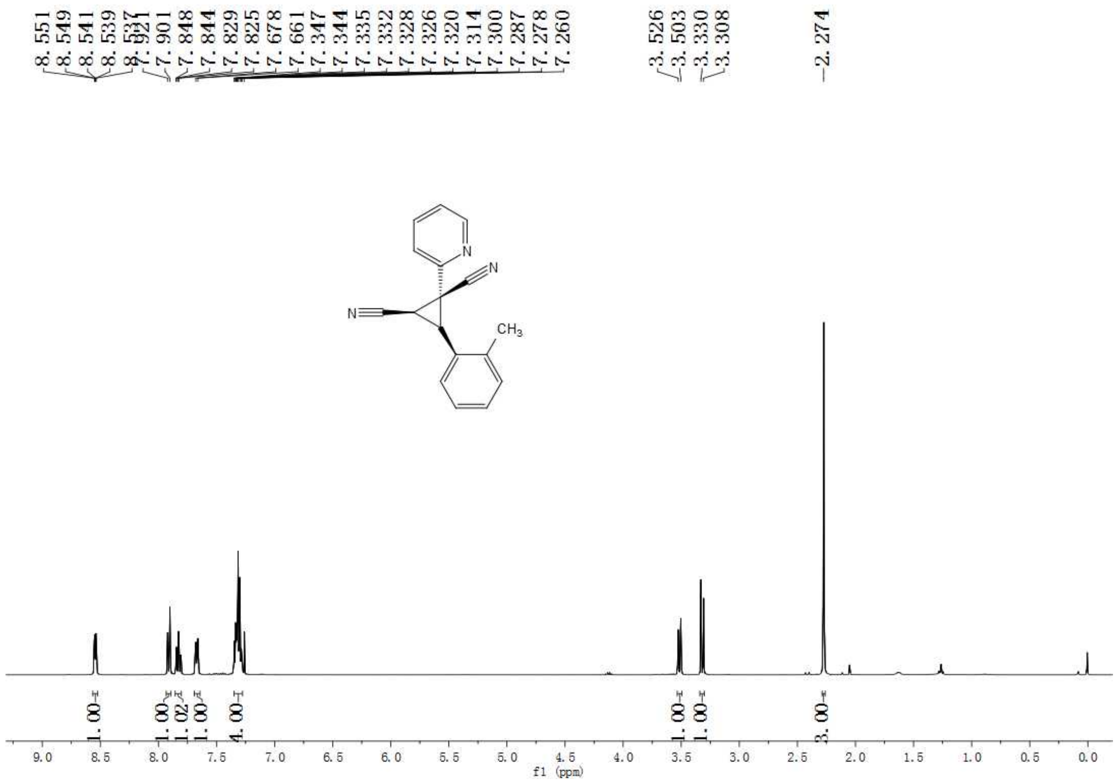

Figure S85. ^{1}H NMR Spectrum of *cis*-4a (400 MHz, $CDCl_3$)

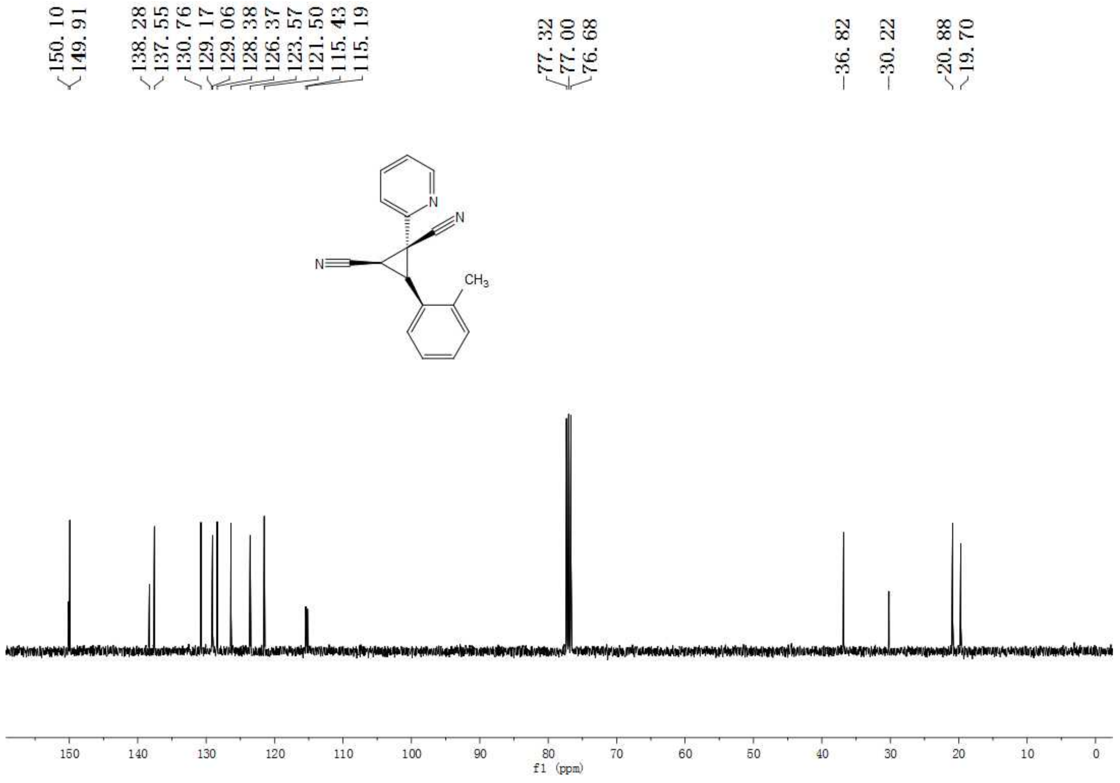

Figure S86. ^{13}C NMR Spectrum of *cis*-4a (100 MHz, $CDCl_3$)

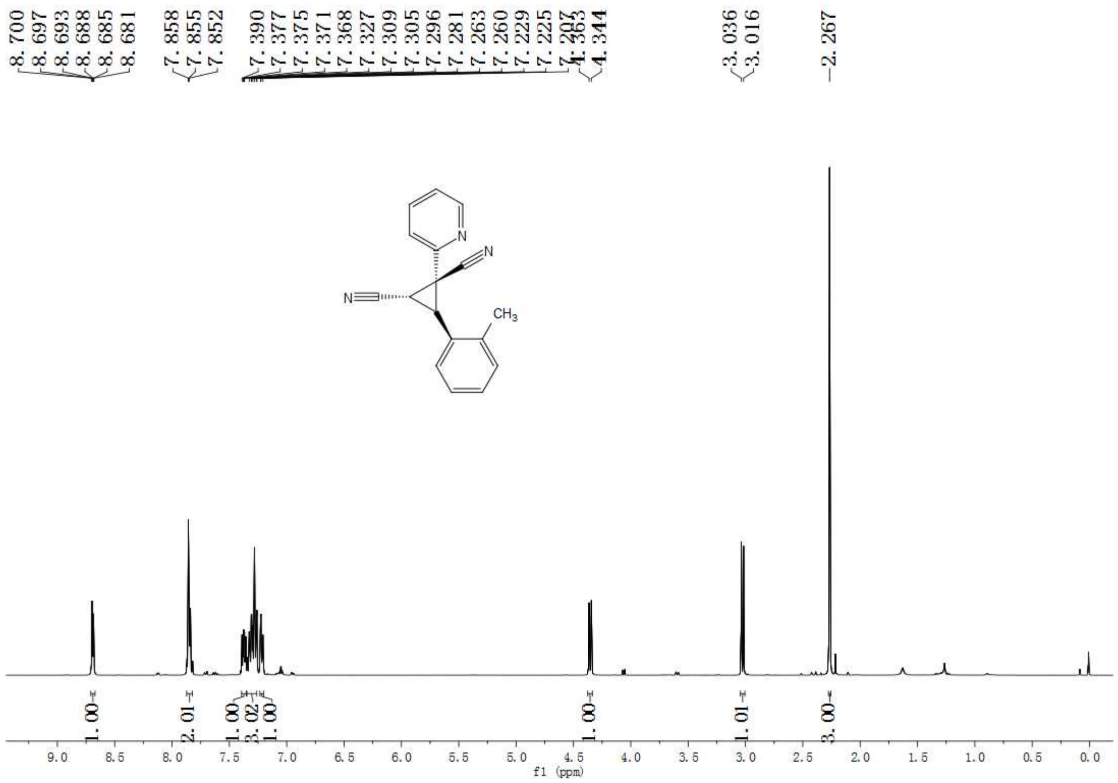

Figure S87. ^{1}H NMR Spectrum of *trans*-4a (400 MHz, $CDCl_3$)

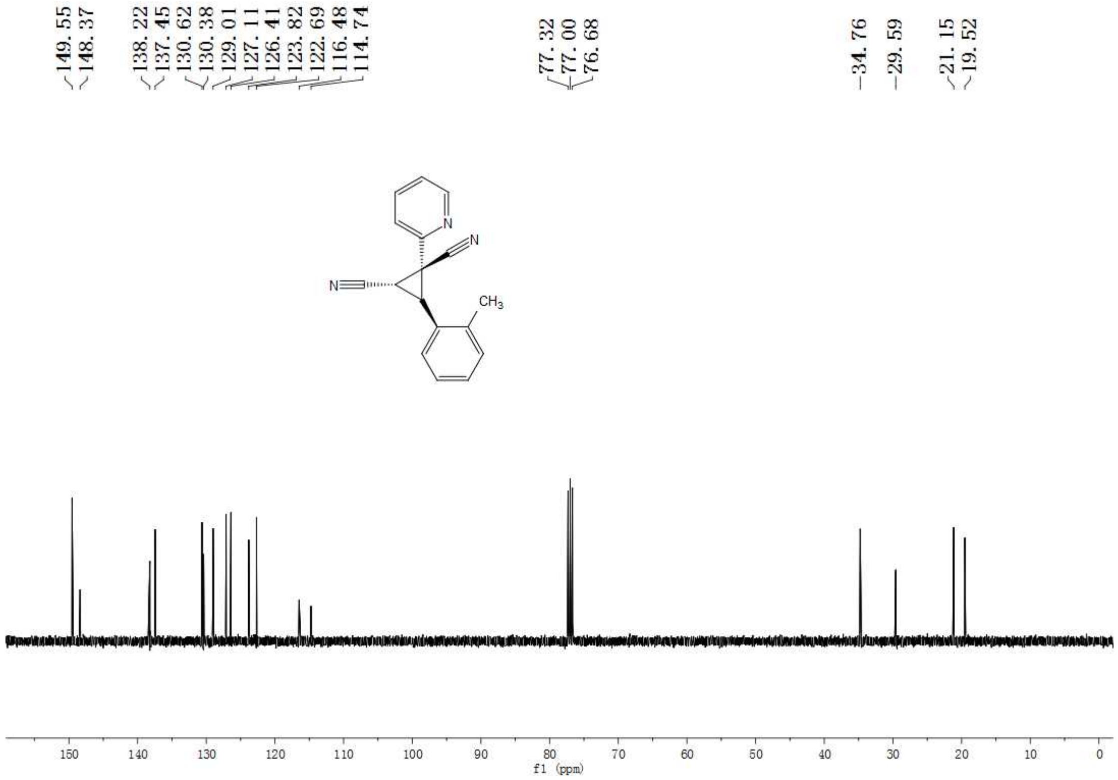

Figure S88. ^{13}C NMR Spectrum of *trans*-4a (100 MHz, $CDCl_3$)

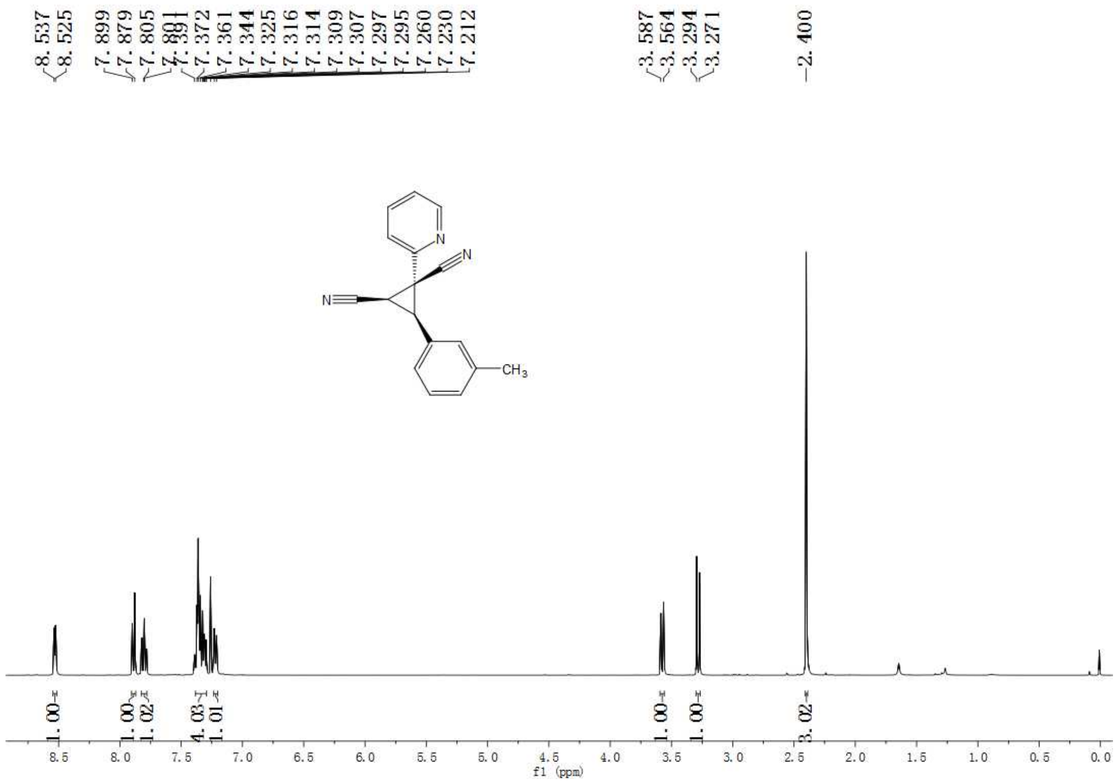

Figure S89. ^{1}H NMR Spectrum of *cis*-4b (400 MHz, $CDCl_3$)

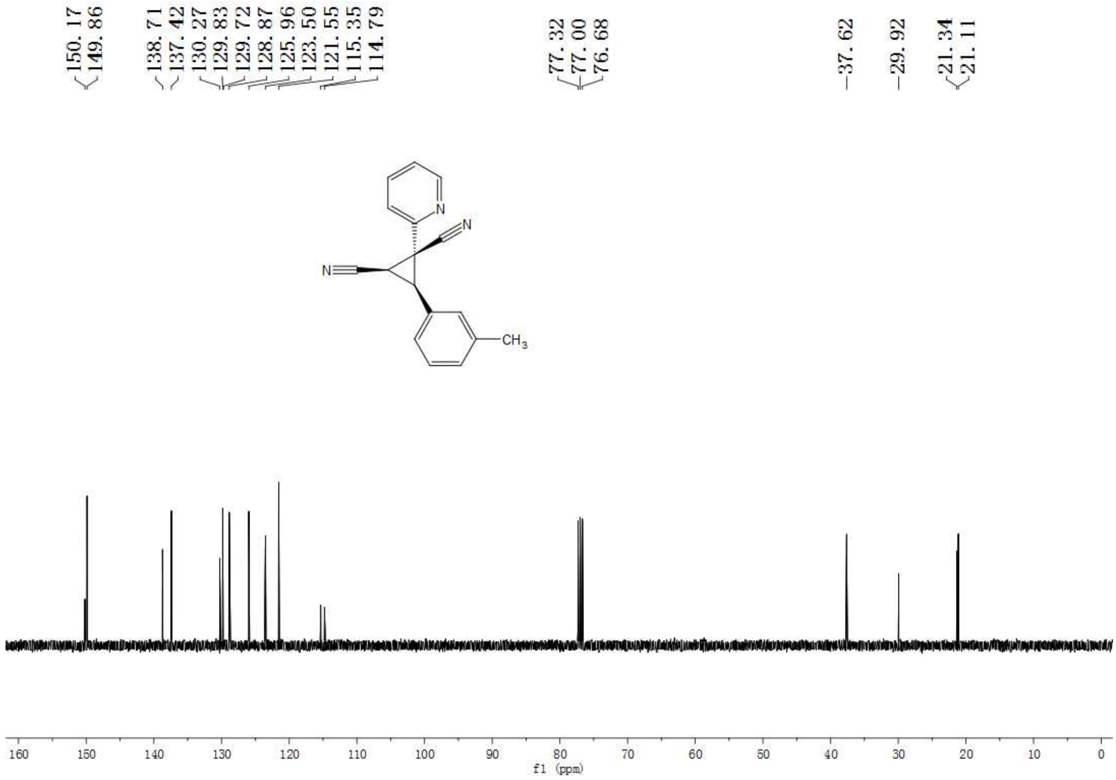

Figure S90. ^{13}C NMR Spectrum of *cis*-4b (100 MHz, $CDCl_3$)

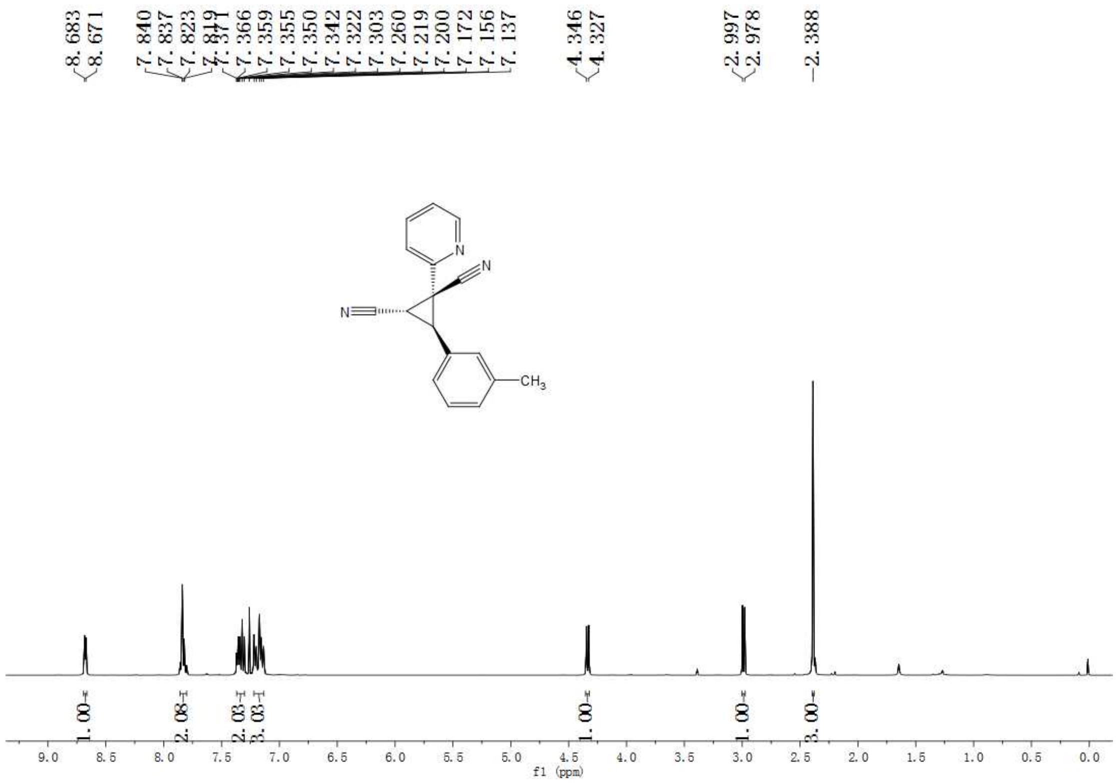

Figure S91. ^{1}H NMR Spectrum of *trans*-4b (400 MHz, $CDCl_3$)

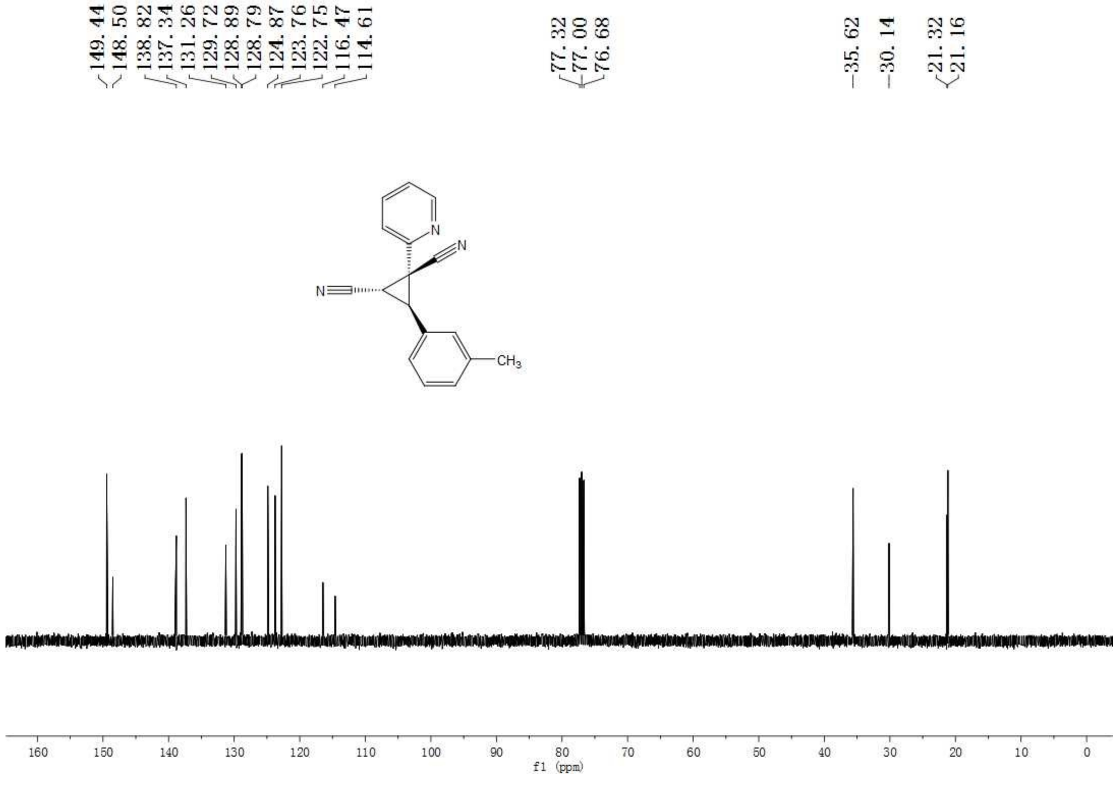

Figure S92. ^{13}C NMR Spectrum of *trans*-4b (100 MHz, $CDCl_3$)

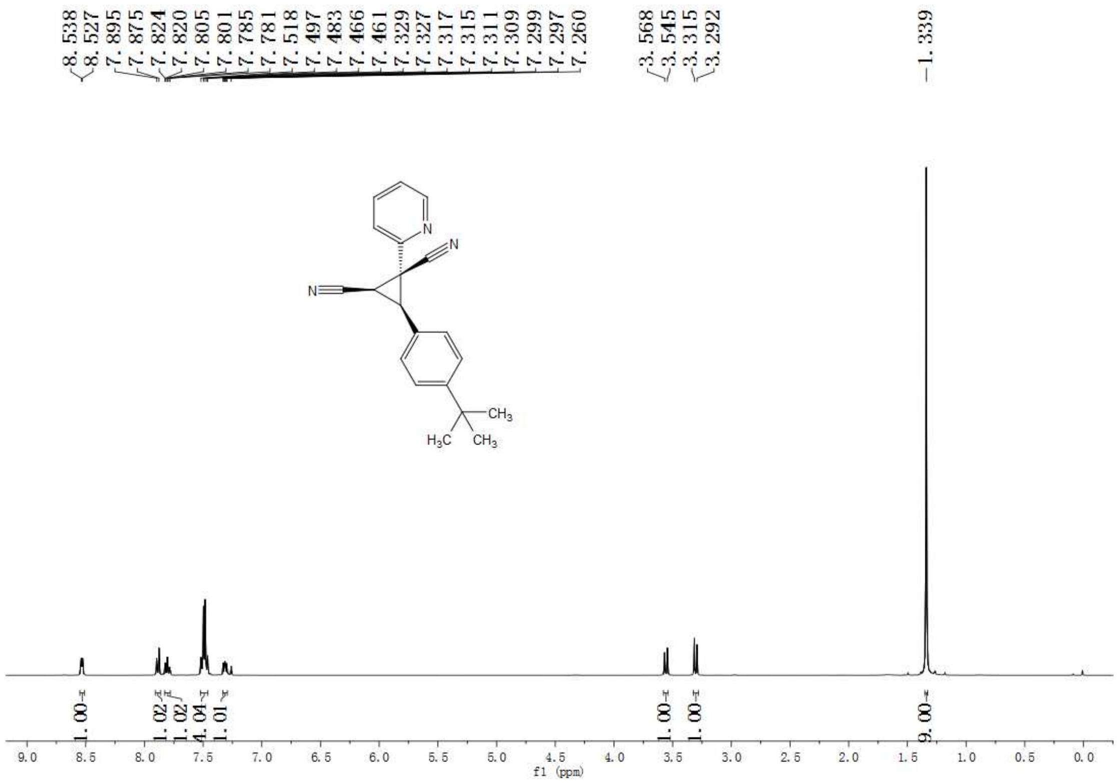

Figure S93. ^{1}H **NMR Spectrum of** ***cis*****-4c (400 MHz,** $CDCl_3$**)**

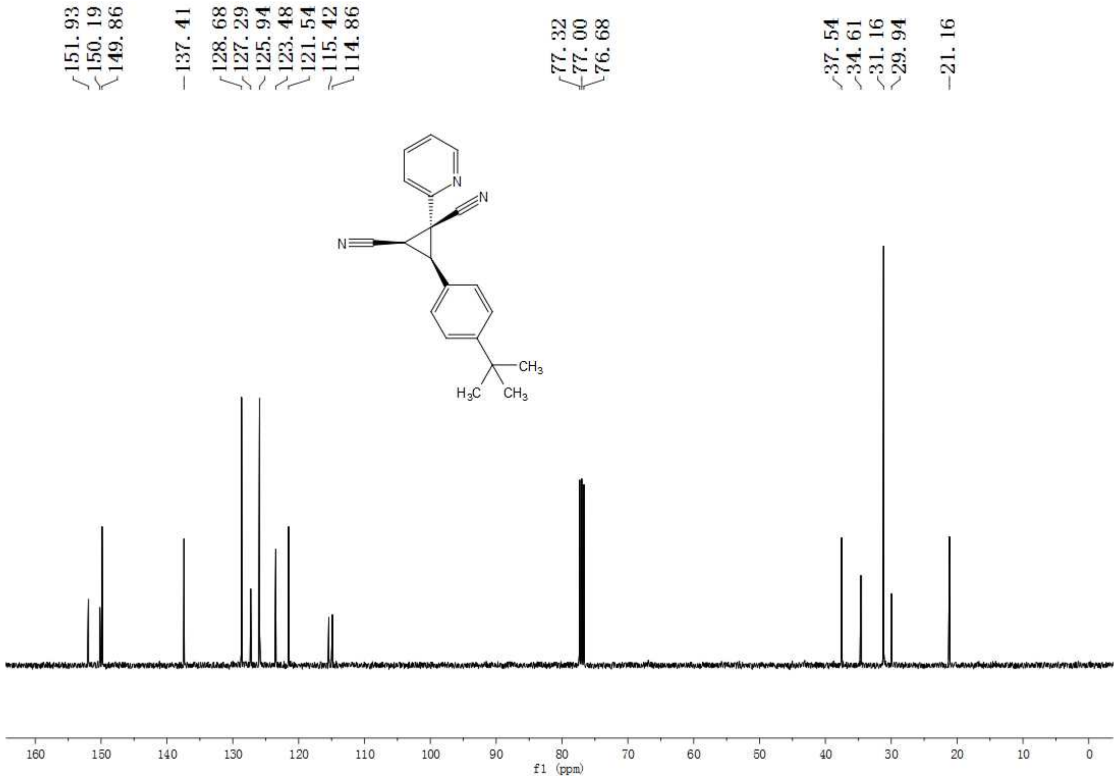

Figure S94. ^{13}C **NMR Spectrum of** ***cis*****-4c (100 MHz,** $CDCl_3$**)**

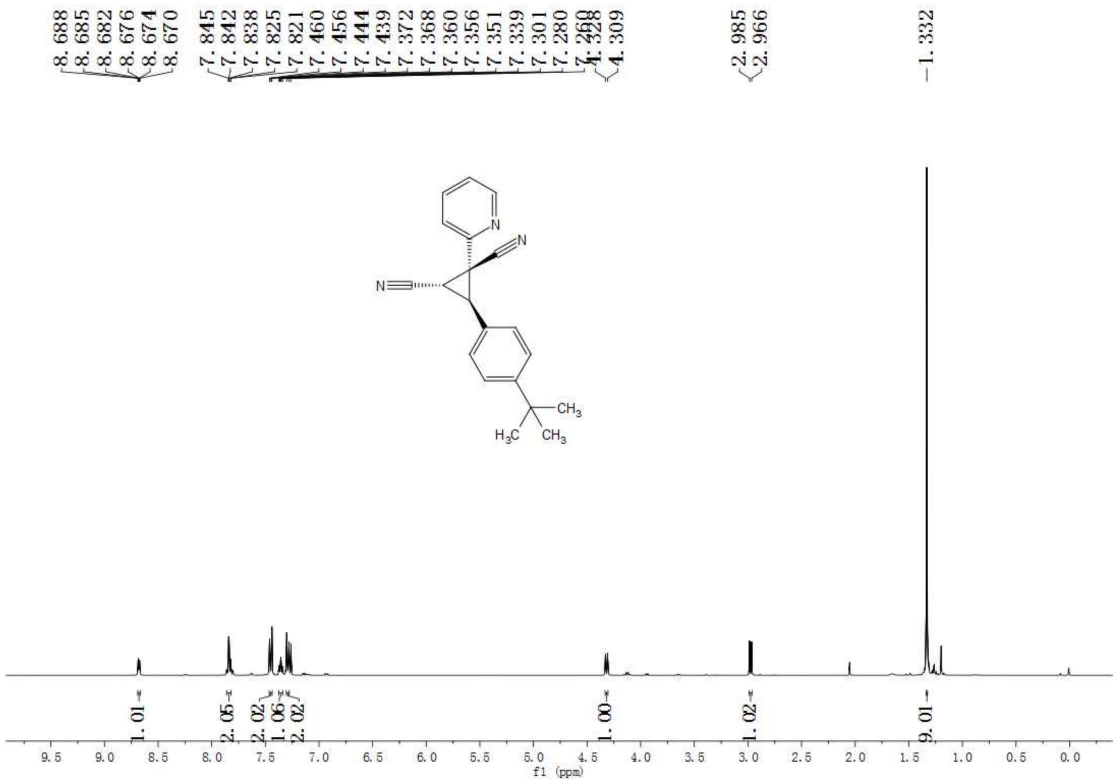

Figure S95. ^{1}H NMR Spectrum of *trans*-4c (400 MHz, $CDCl_3$)

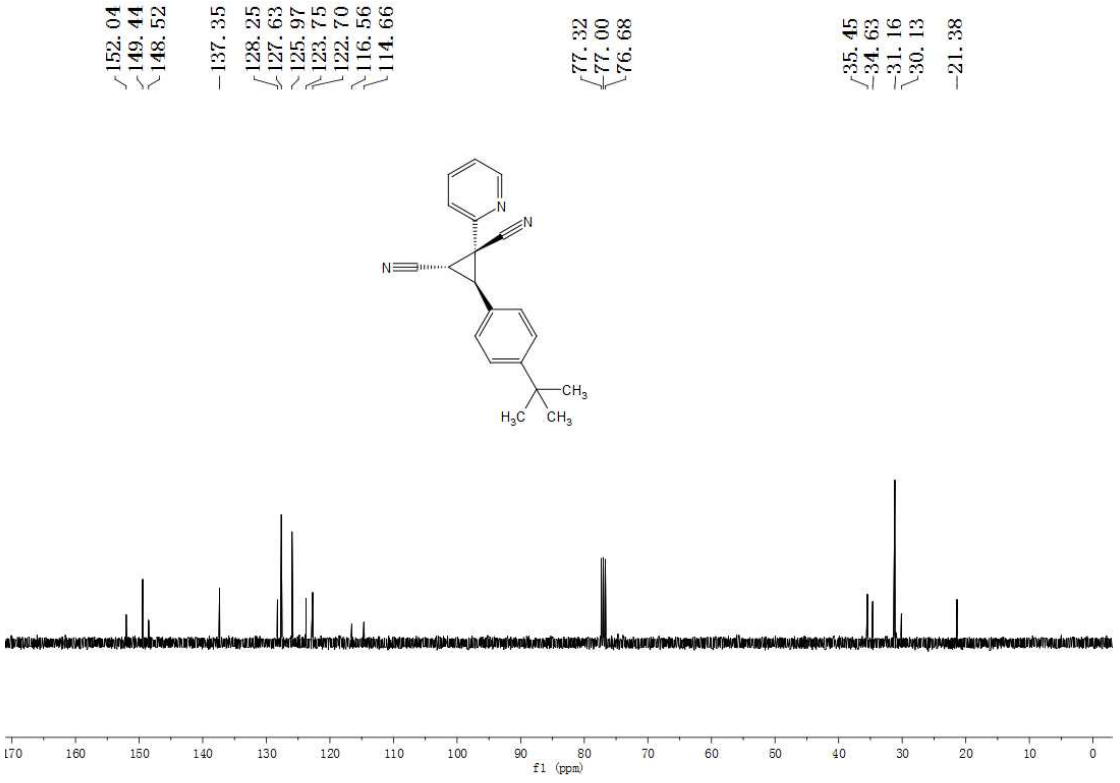

Figure S96. ^{13}C NMR Spectrum of *trans*-4c (100 MHz, $CDCl_3$)

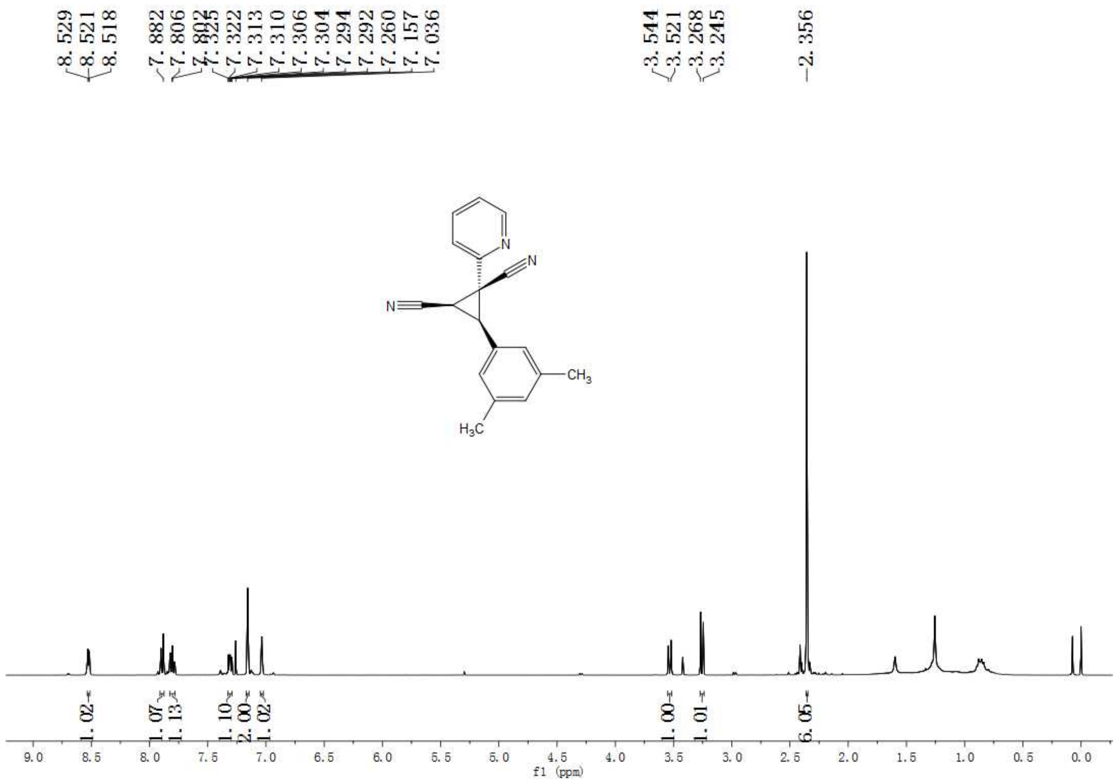

Figure S97. ^{1}H NMR Spectrum of *cis*-4d (400 MHz, $CDCl_3$)

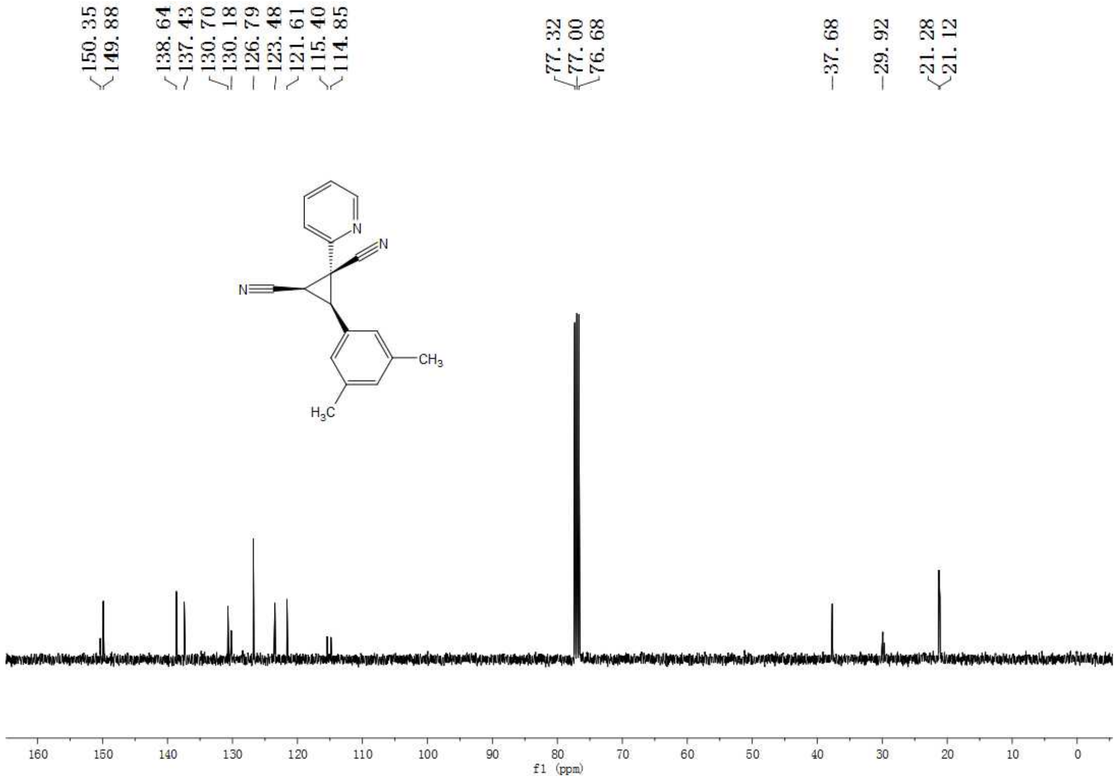

Figure S98. ^{13}C NMR Spectrum of *cis*-4d (100 MHz, $CDCl_3$)

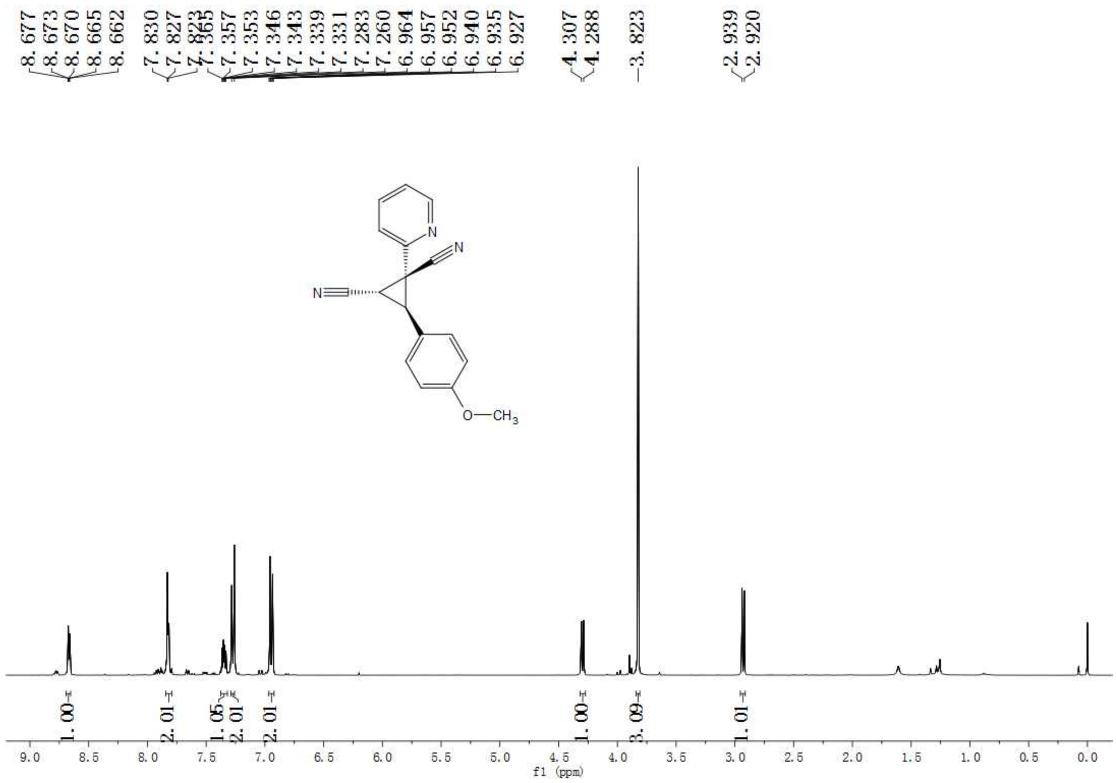

Figure S99. ^{1}H NMR Spectrum of *trans*-4e (400 MHz, $CDCl_3$)

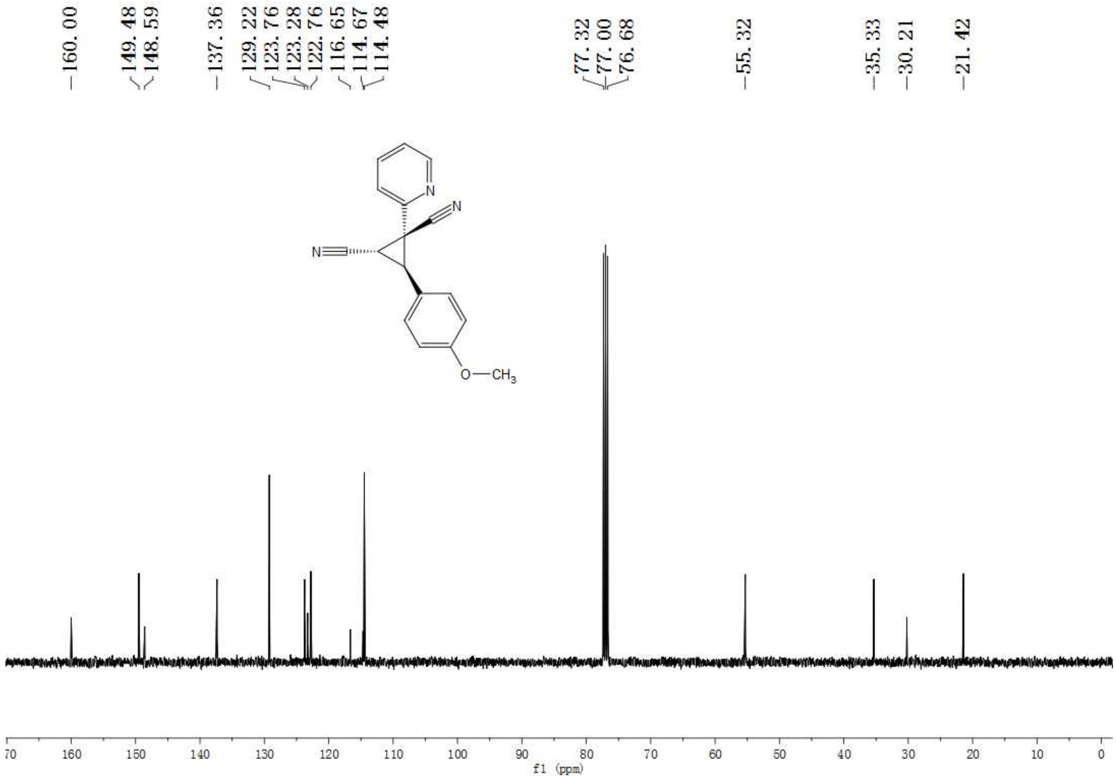

Figure S100. ^{13}C NMR Spectrum of *trans*-4e (100 MHz, $CDCl_3$)

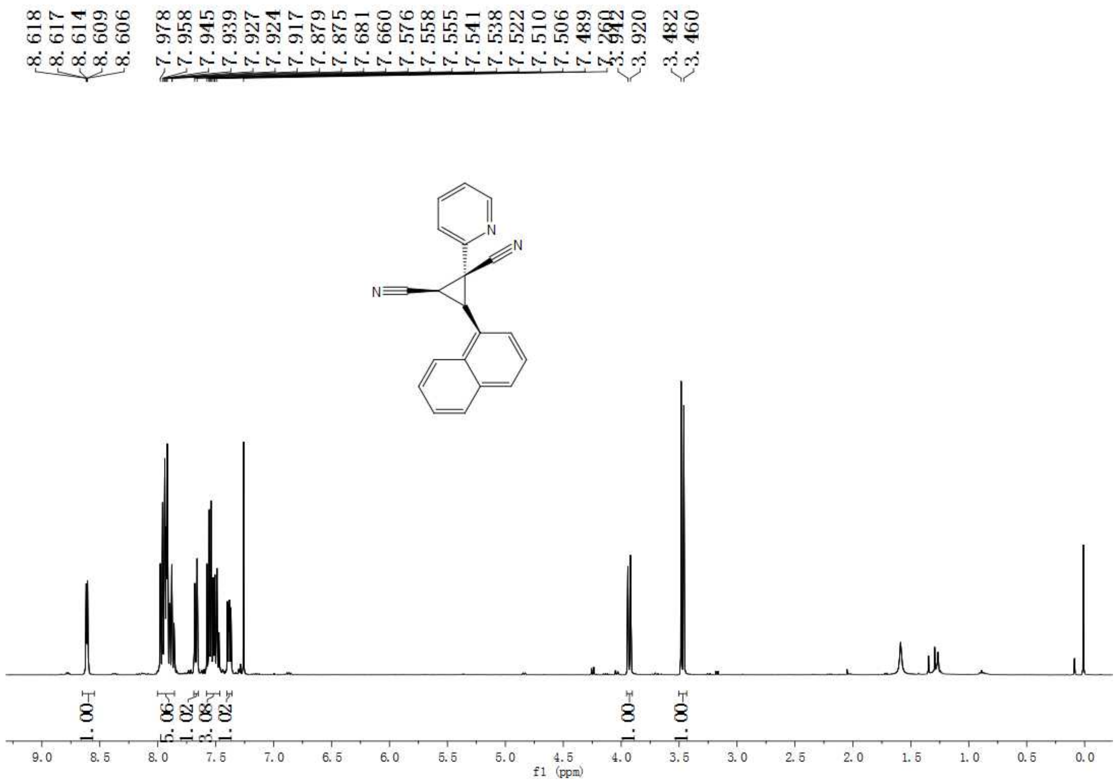

Figure S101. ^{1}H NMR Spectrum of *cis*-4f (400 MHz, $CDCl_3$)

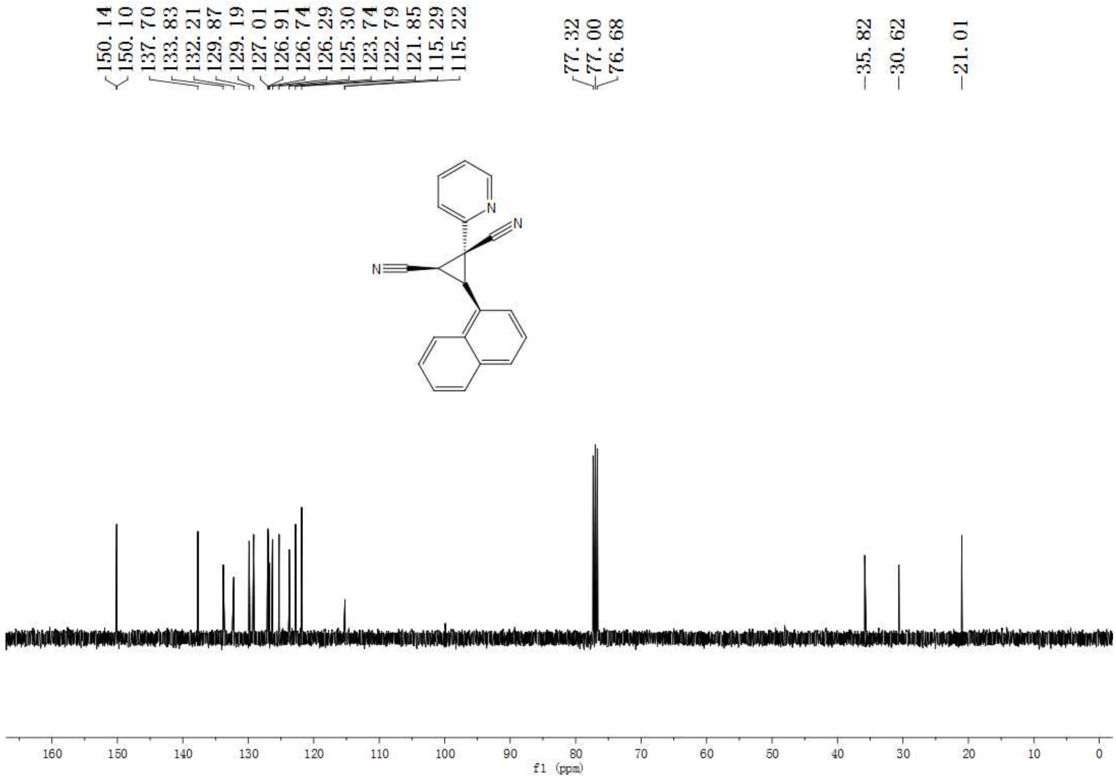

Figure S102. ^{13}C NMR Spectrum of *cis*-4f (100 MHz, $CDCl_3$)

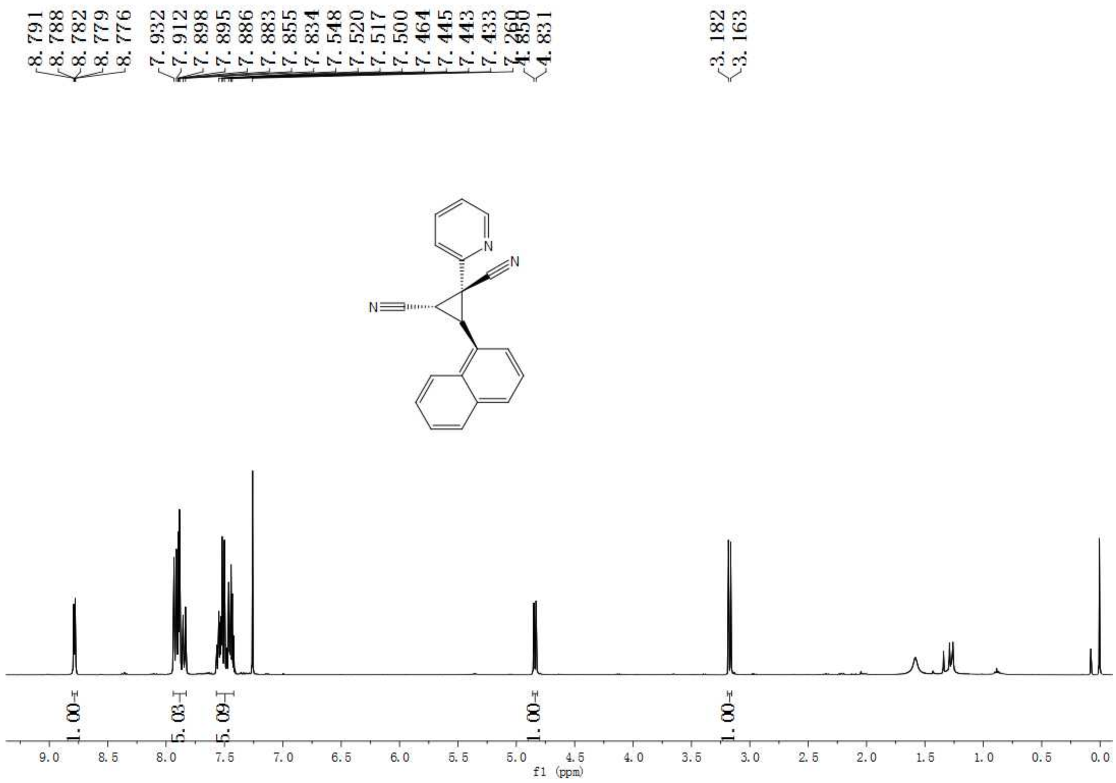

Figure S103. ^{1}H NMR Spectrum of *trans*-4f (400 MHz, $CDCl_3$)

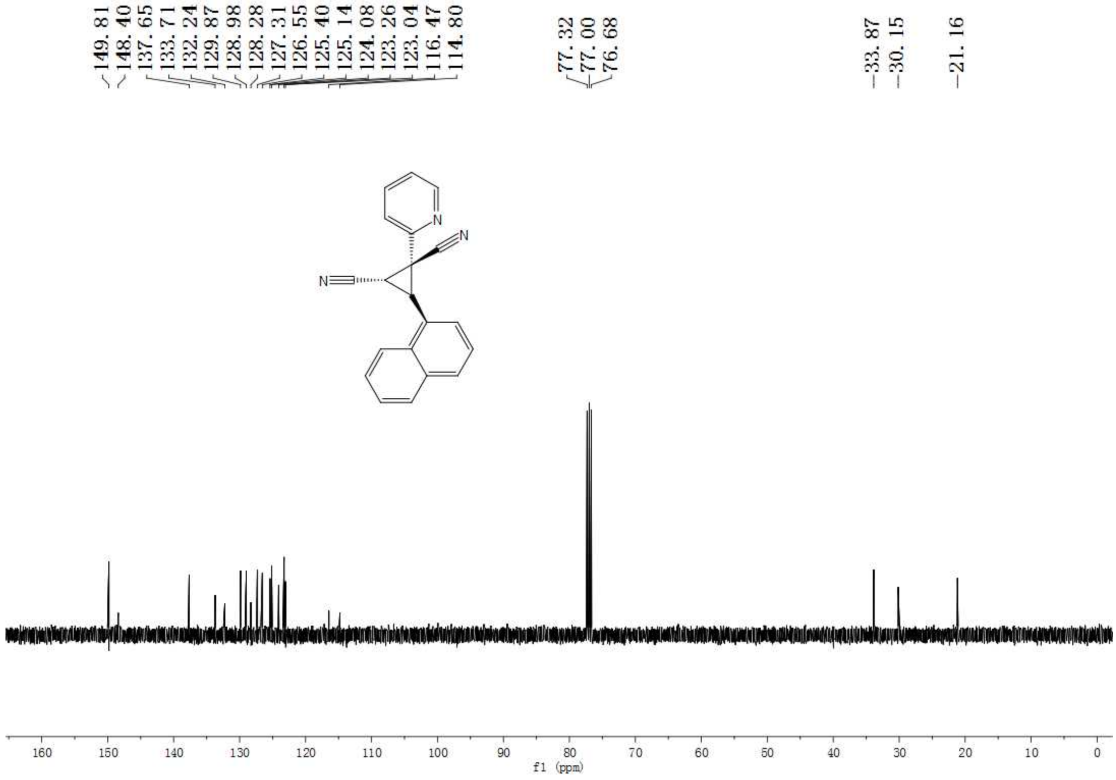

Figure S104. ^{13}C NMR Spectrum of *trans*-4f (100 MHz, $CDCl_3$)

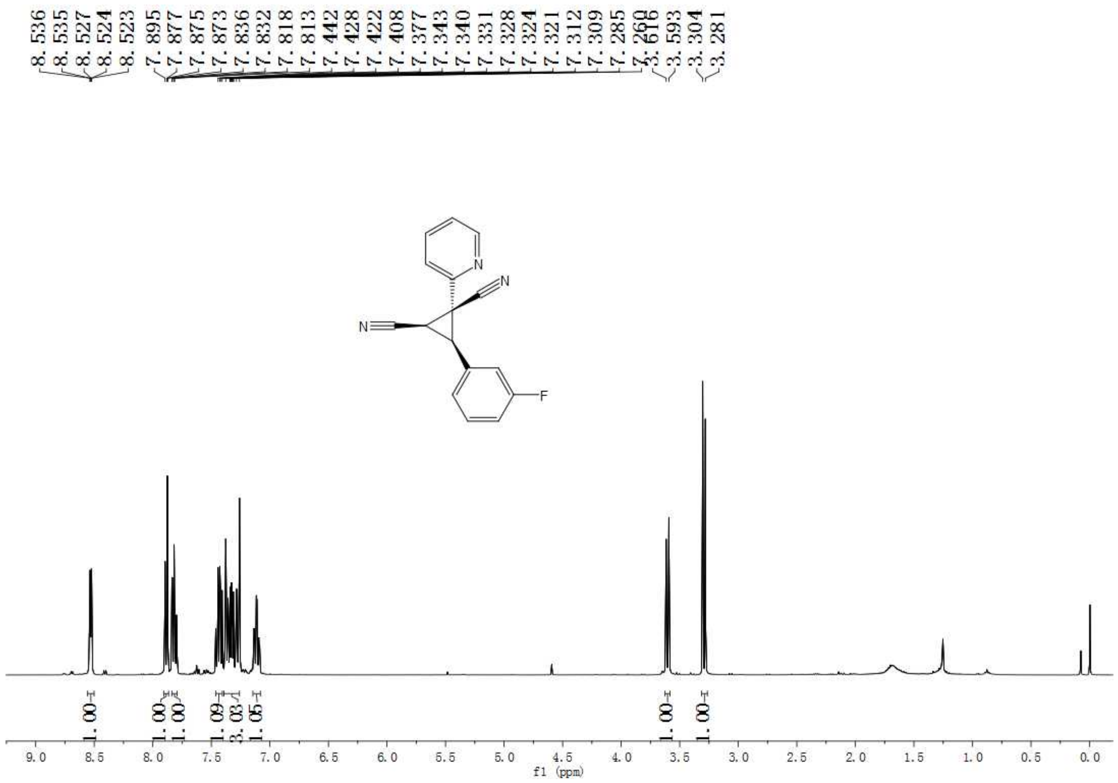

Figure S105. ^{1}H NMR Spectrum of *cis*-4g (400 MHz, $CDCl_3$)

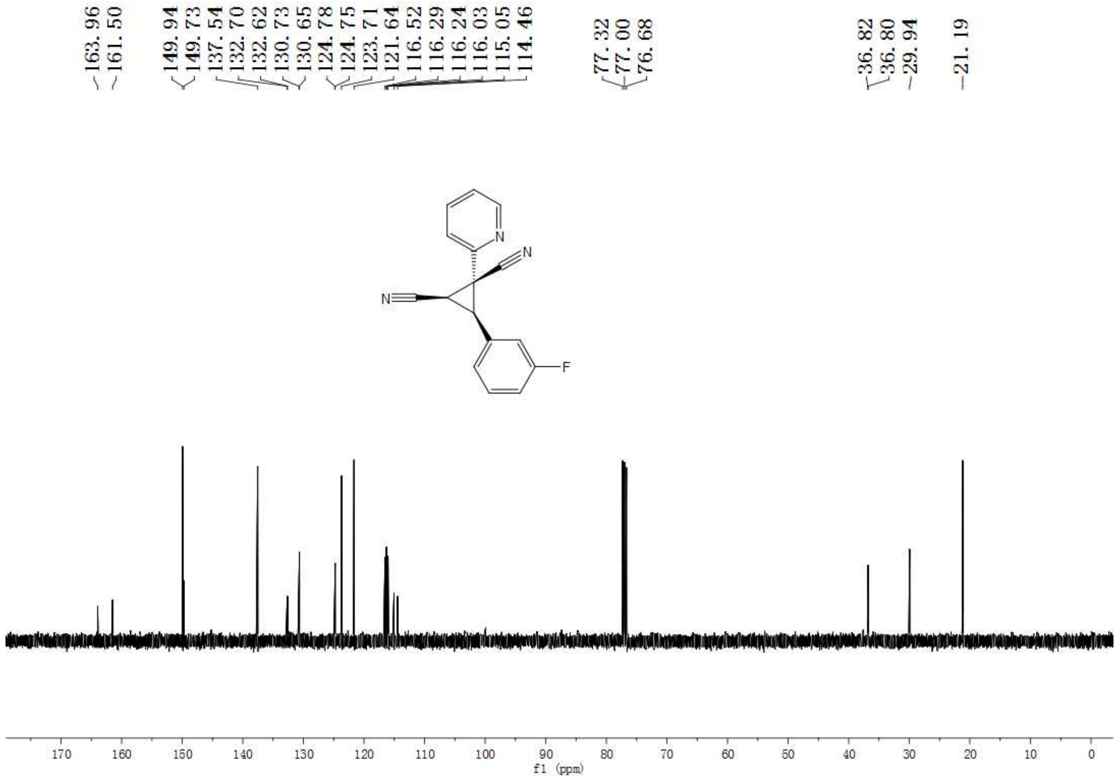

Figure S106. ^{13}C NMR Spectrum of *cis*-4g (100 MHz, $CDCl_3$)

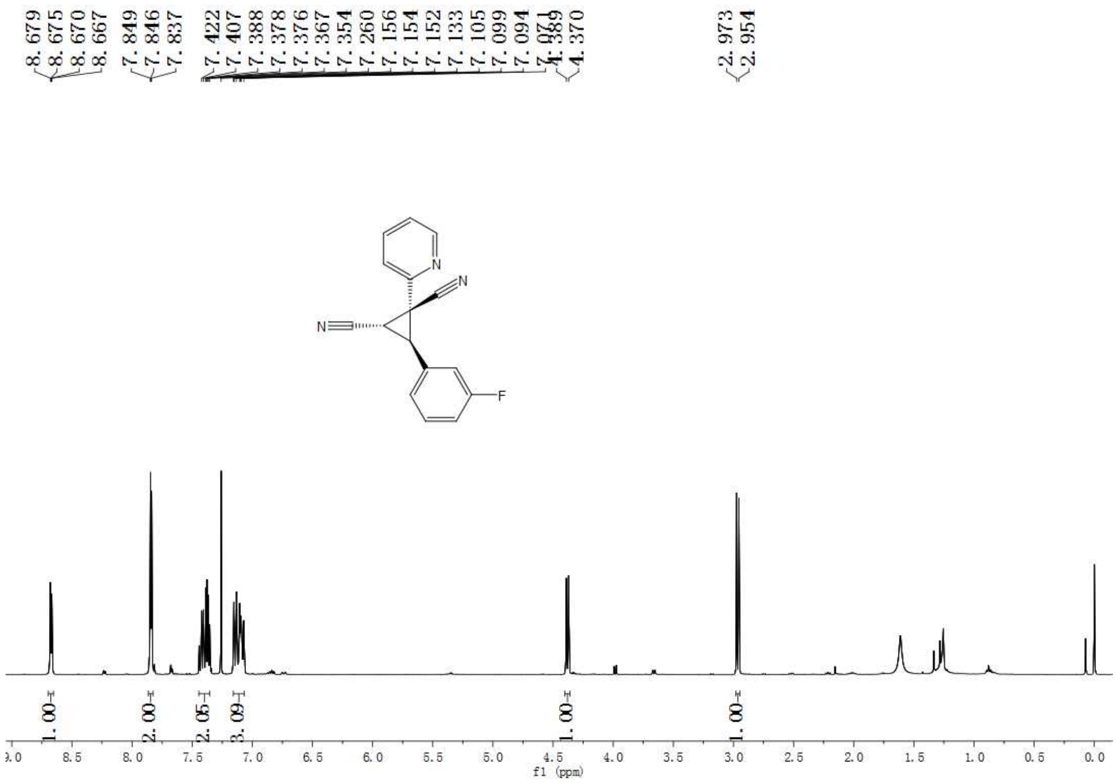

Figure S107. ¹H NMR Spectrum of *trans*-4g (400 MHz, $CDCl_3$)

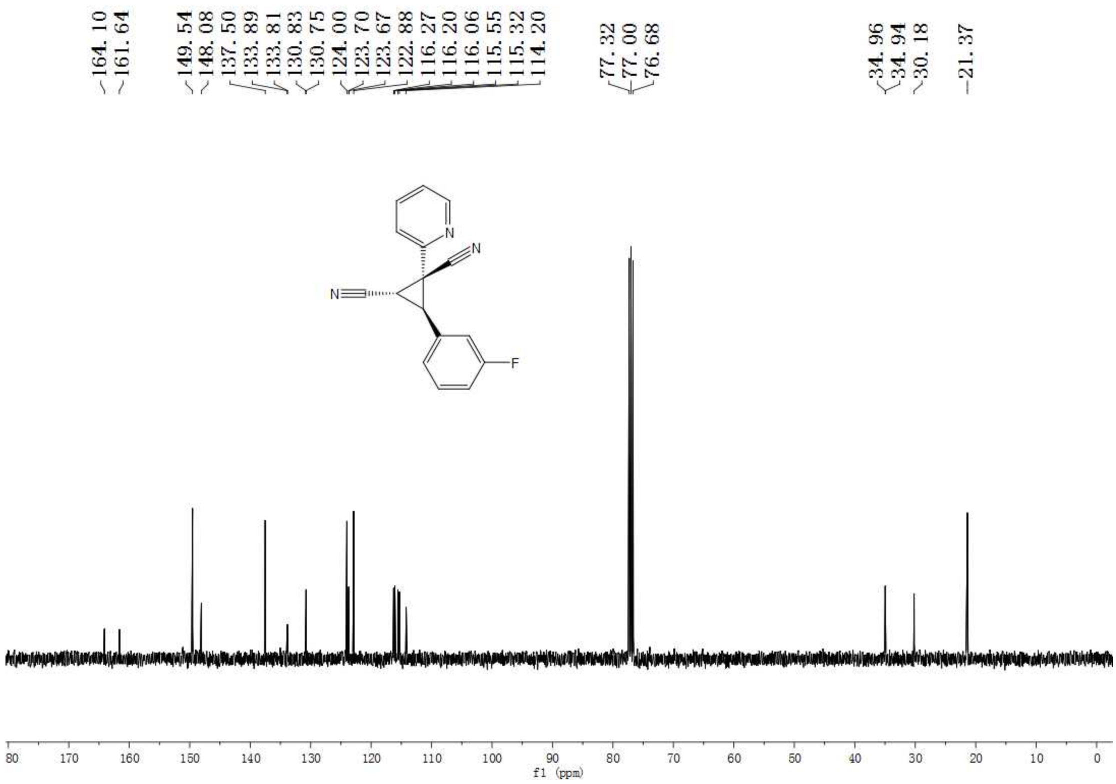

Figure S108. ¹³C NMR Spectrum of *trans*-4g (100 MHz, $CDCl_3$)

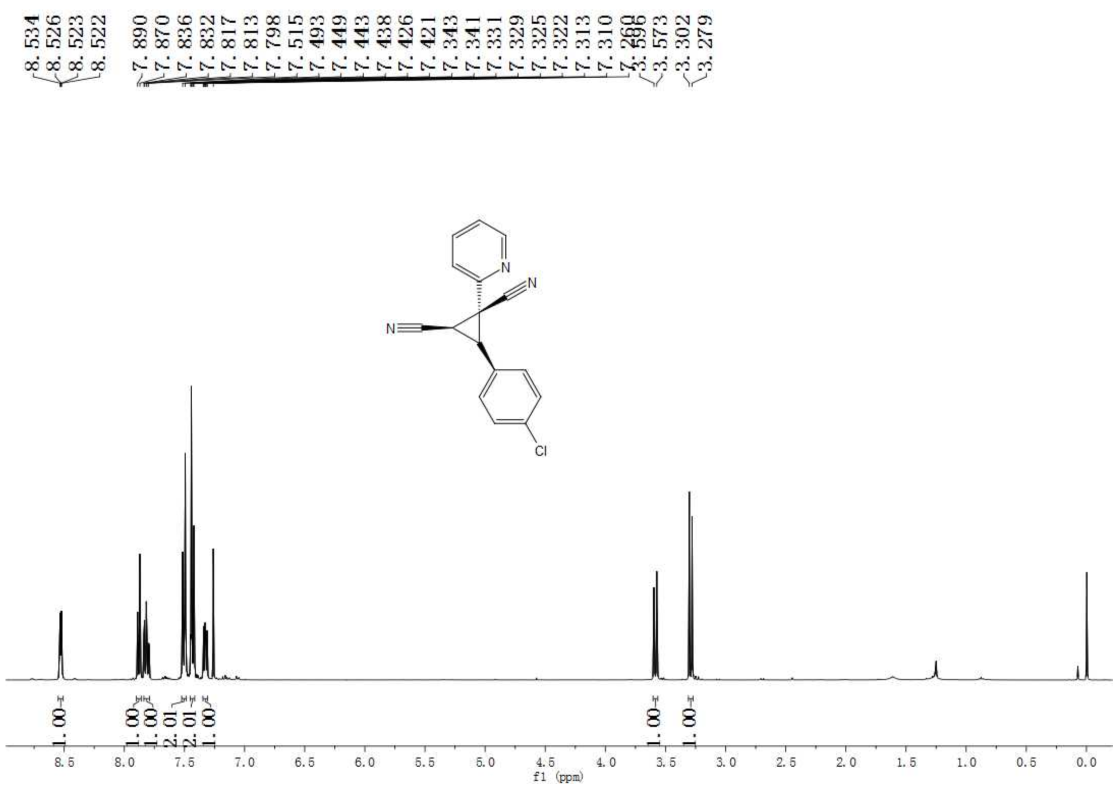

Figure S109. ^{1}H NMR Spectrum of *cis*-4h (400 MHz, $CDCl_3$)

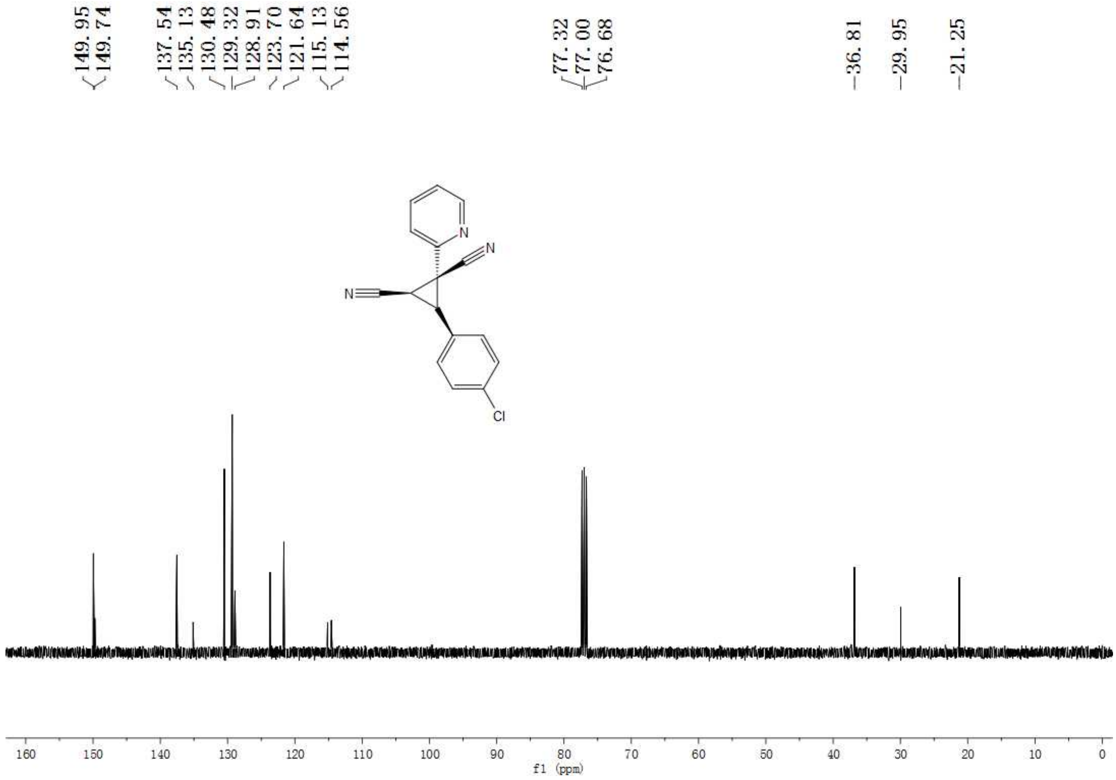

Figure S110. ^{13}C NMR Spectrum of *cis*-4h (100 MHz, $CDCl_3$)

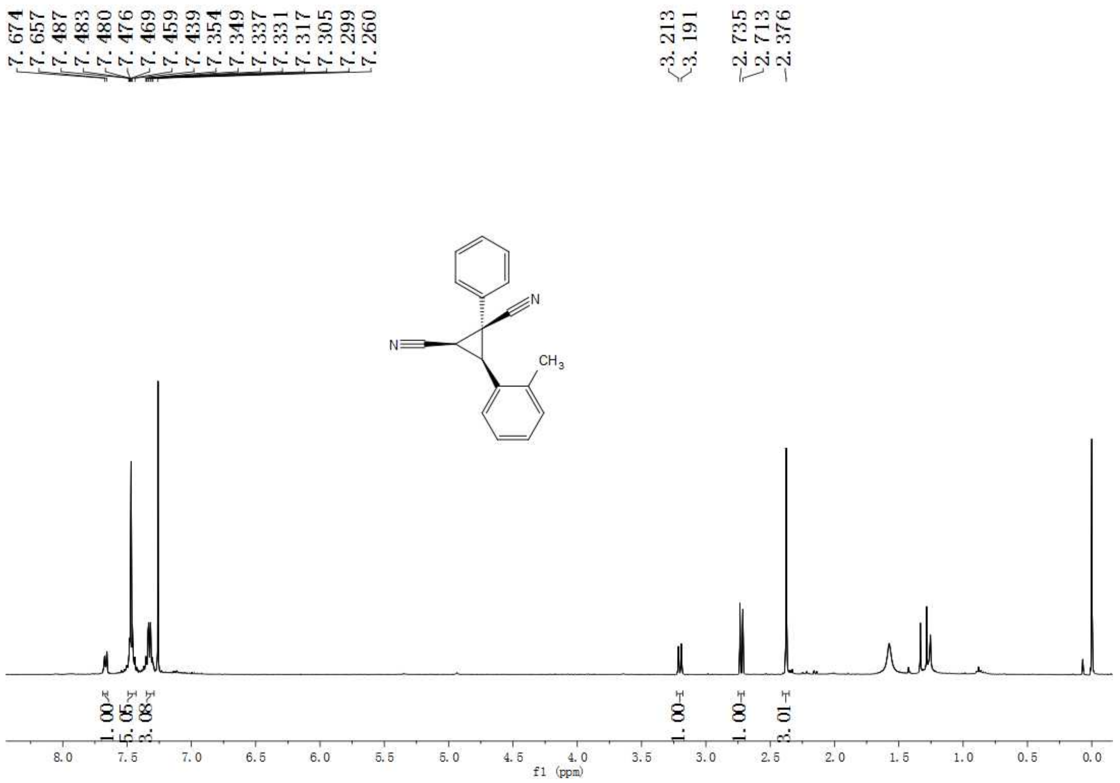

Figure S111. ^{1}H NMR Spectrum of *cis*-4i (400 MHz, $CDCl_3$)

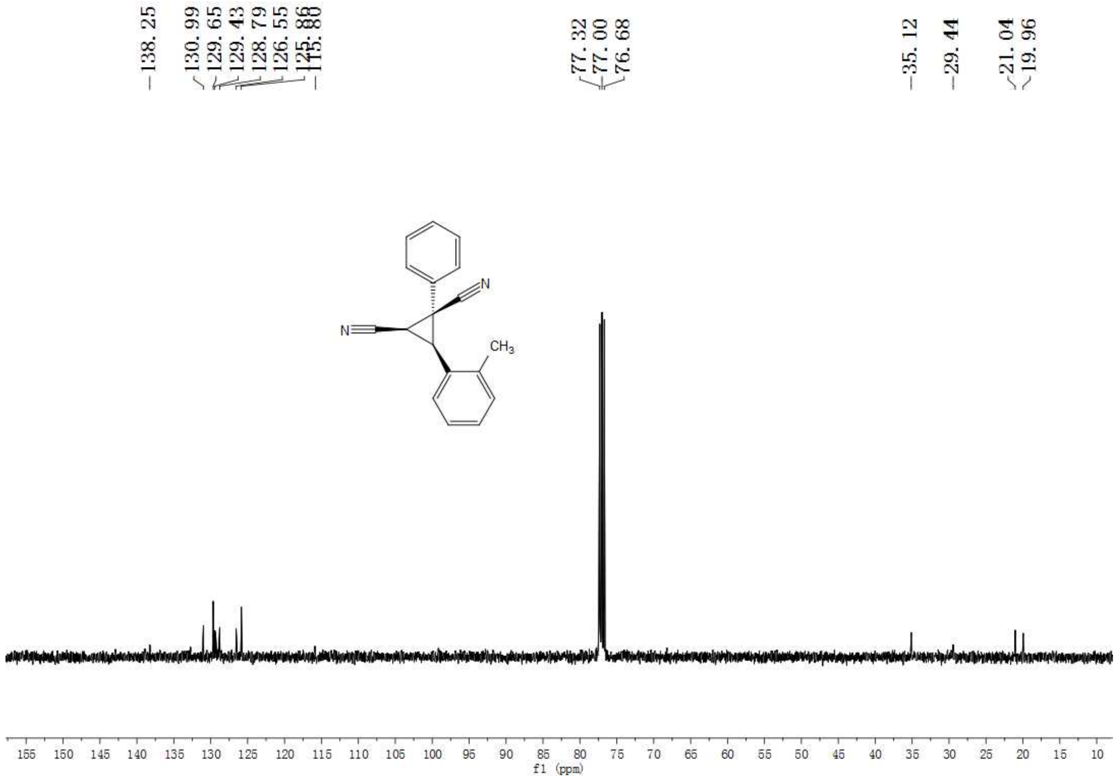

Figure S112. ^{13}C NMR Spectrum of *cis*-4i (100 MHz, $CDCl_3$)

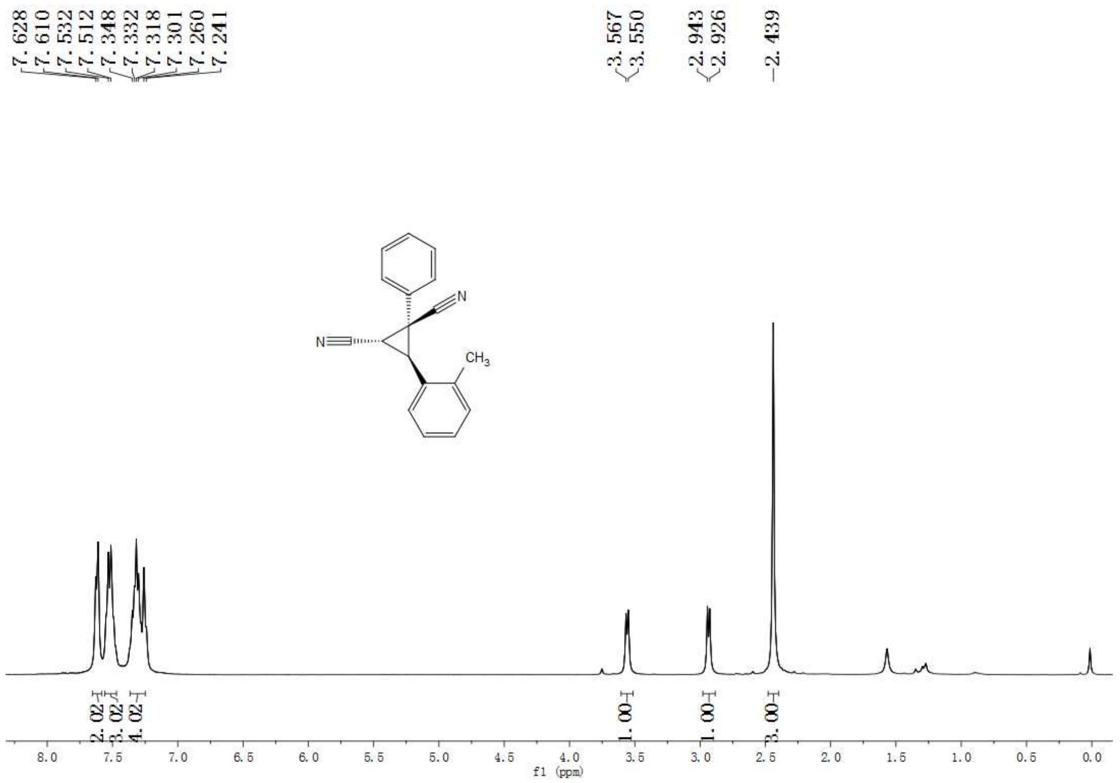

Figure S113. ^{1}H NMR Spectrum of *trans*-4i (400 MHz, $CDCl_3$)

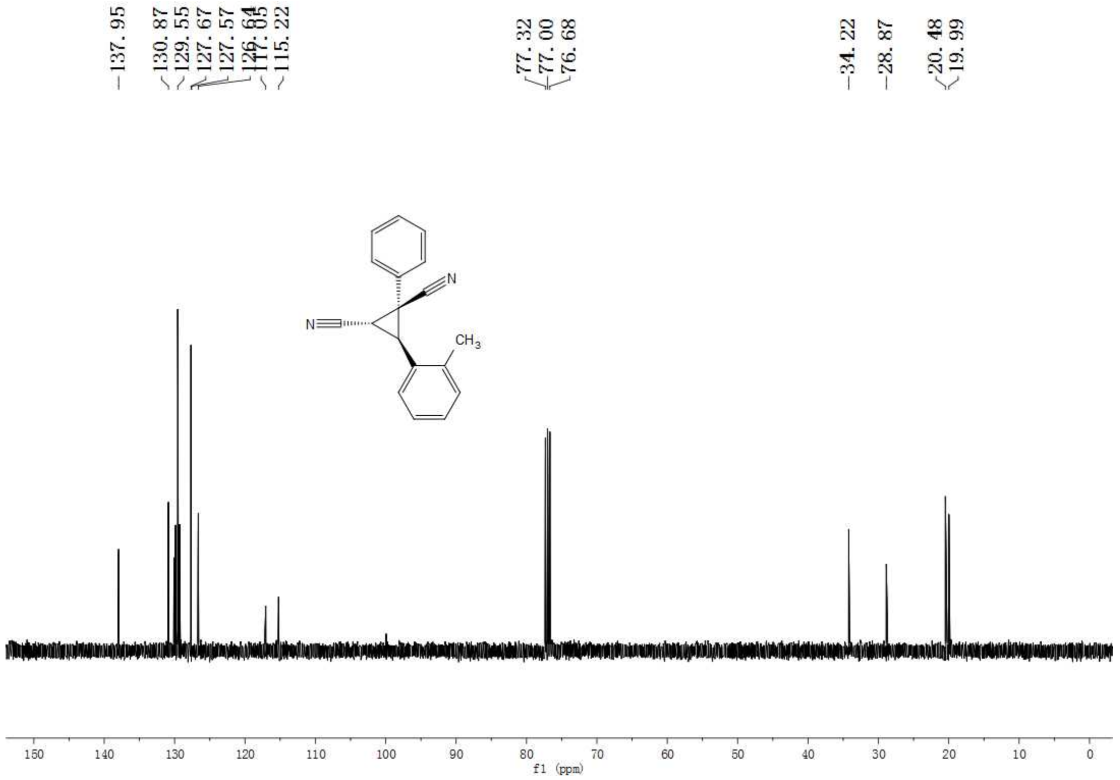

Figure S114. ^{13}C NMR Spectrum of *trans*-4i (100 MHz, $CDCl_3$)

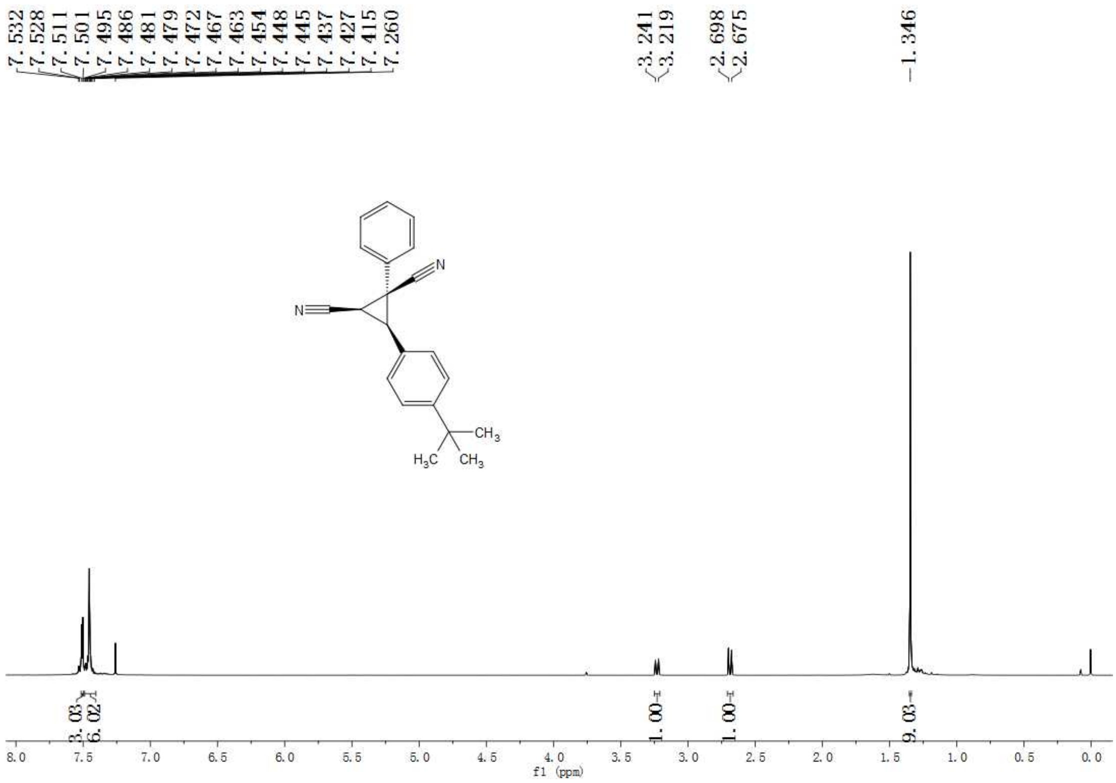

Figure S115. ^{1}H NMR Spectrum of *cis*-4j (400 MHz, $CDCl_3$)

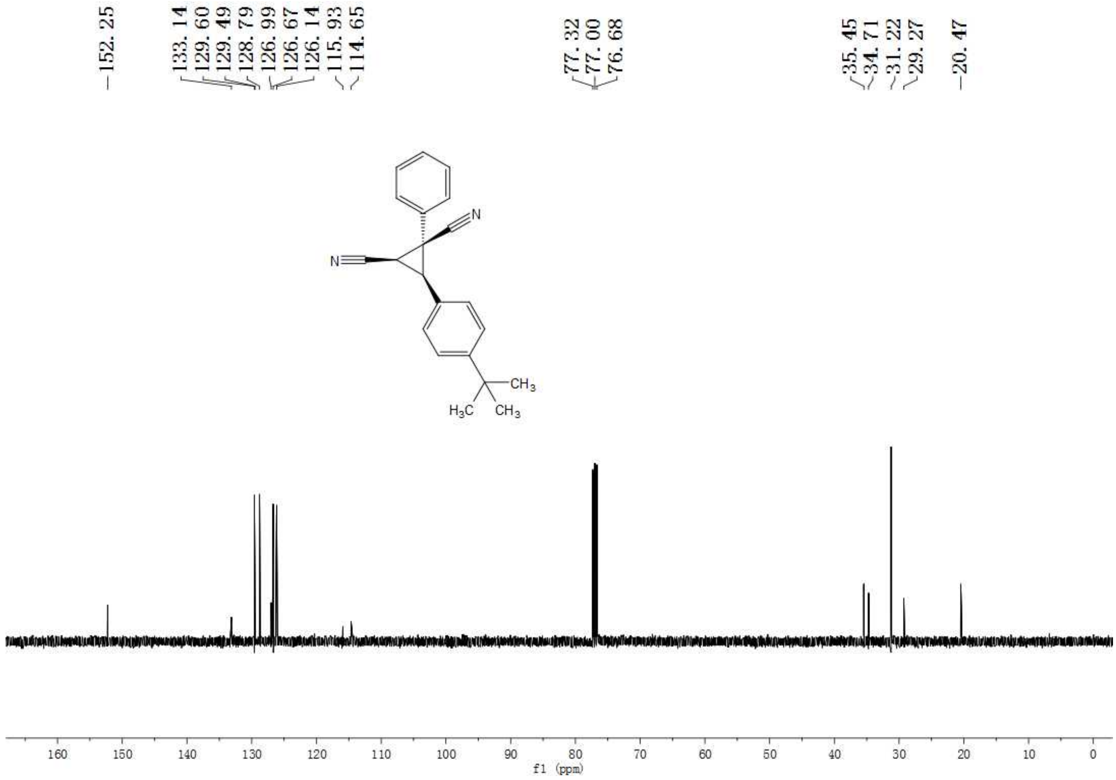

Figure S116. ^{13}C NMR Spectrum of *cis*-4j (100 MHz, $CDCl_3$)

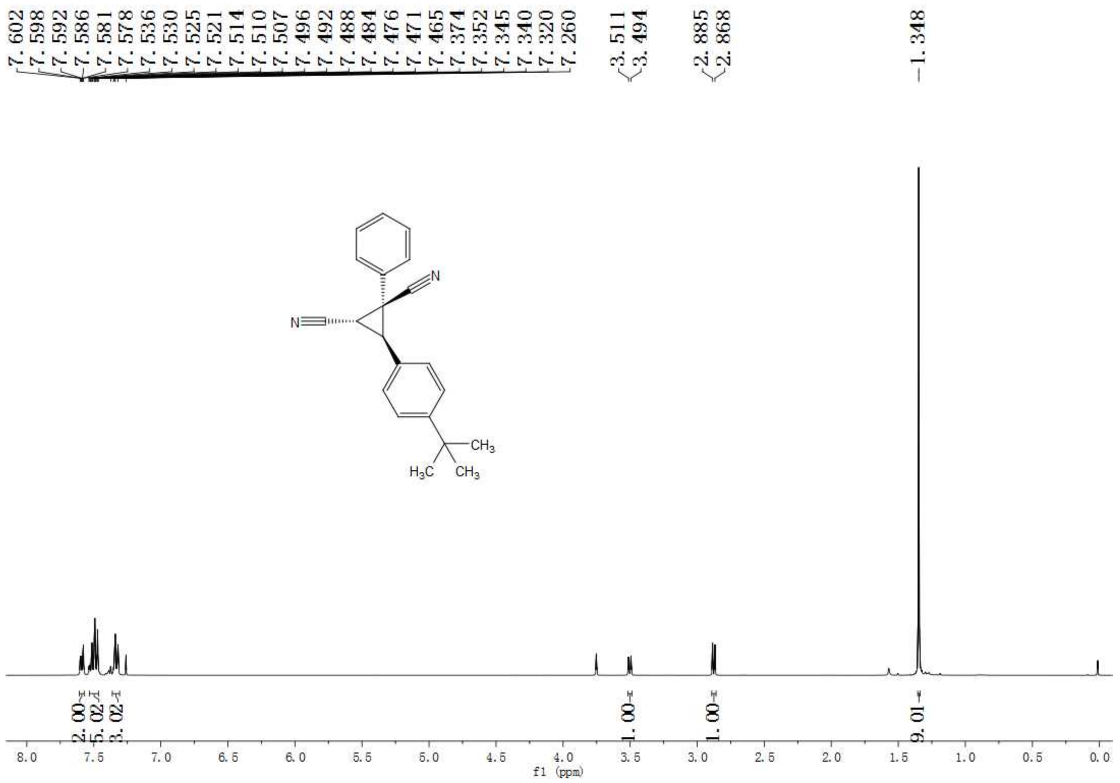

Figure S117. ^{1}H NMR Spectrum of *trans*-4j (400 MHz, $CDCl_3$)

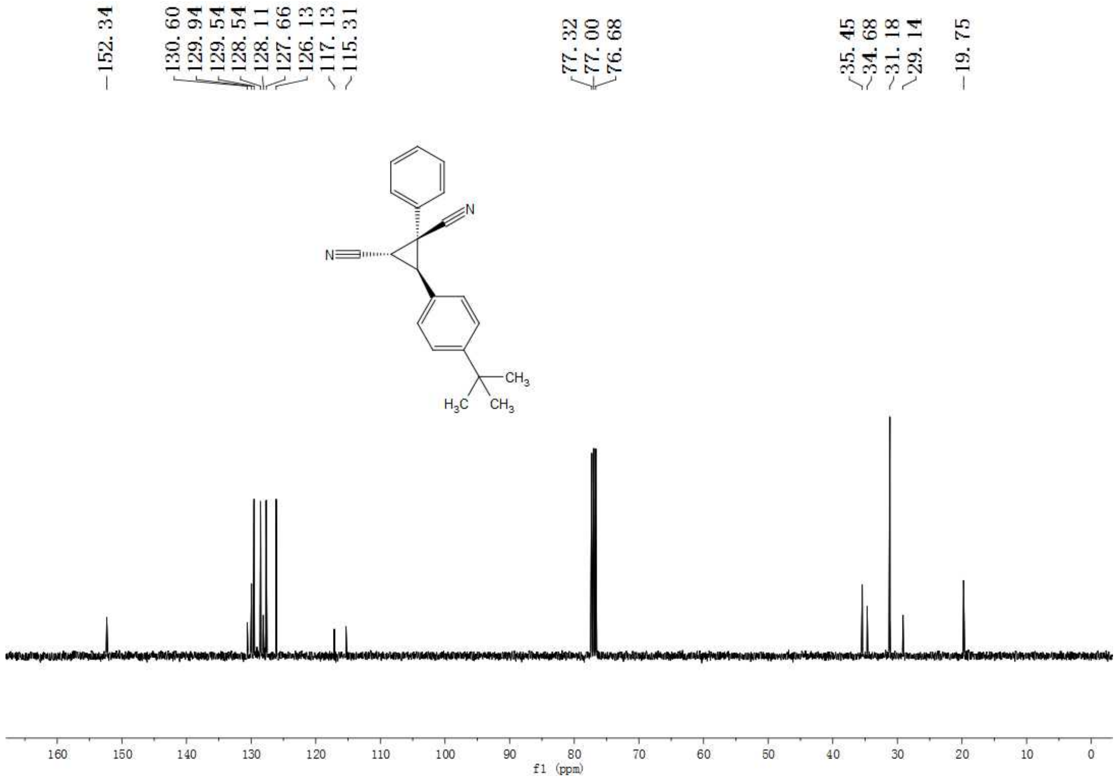

Figure S118. ^{13}C NMR Spectrum of *trans*-4j (100 MHz, $CDCl_3$)

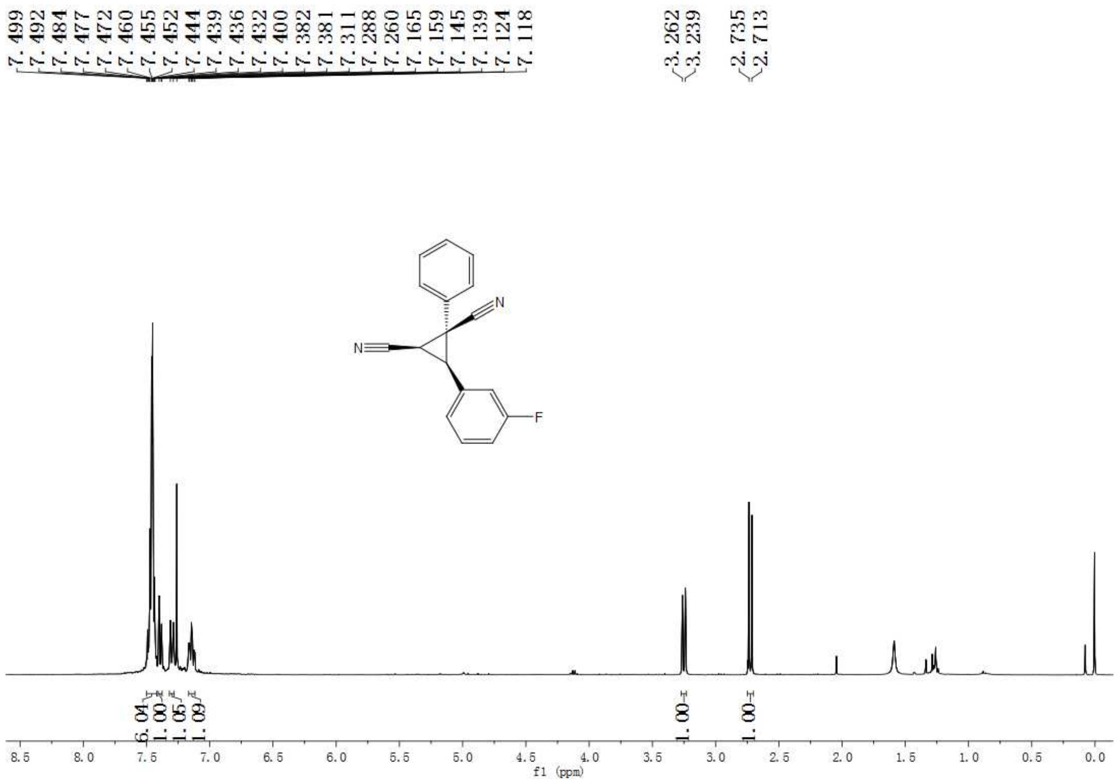

Figure S119. ^{1}H NMR Spectrum of *cis*-4k (400 MHz, $CDCl_3$)

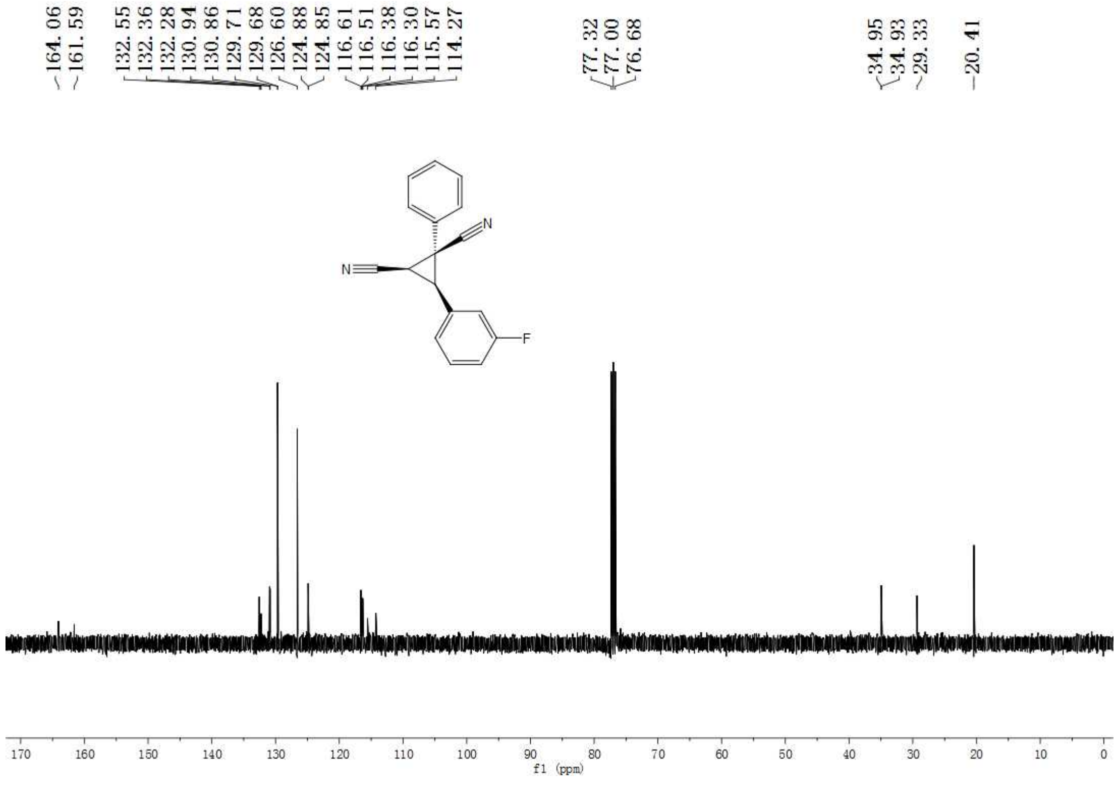

Figure S120. ^{13}C NMR Spectrum of *cis*-4k (100 MHz, $CDCl_3$)

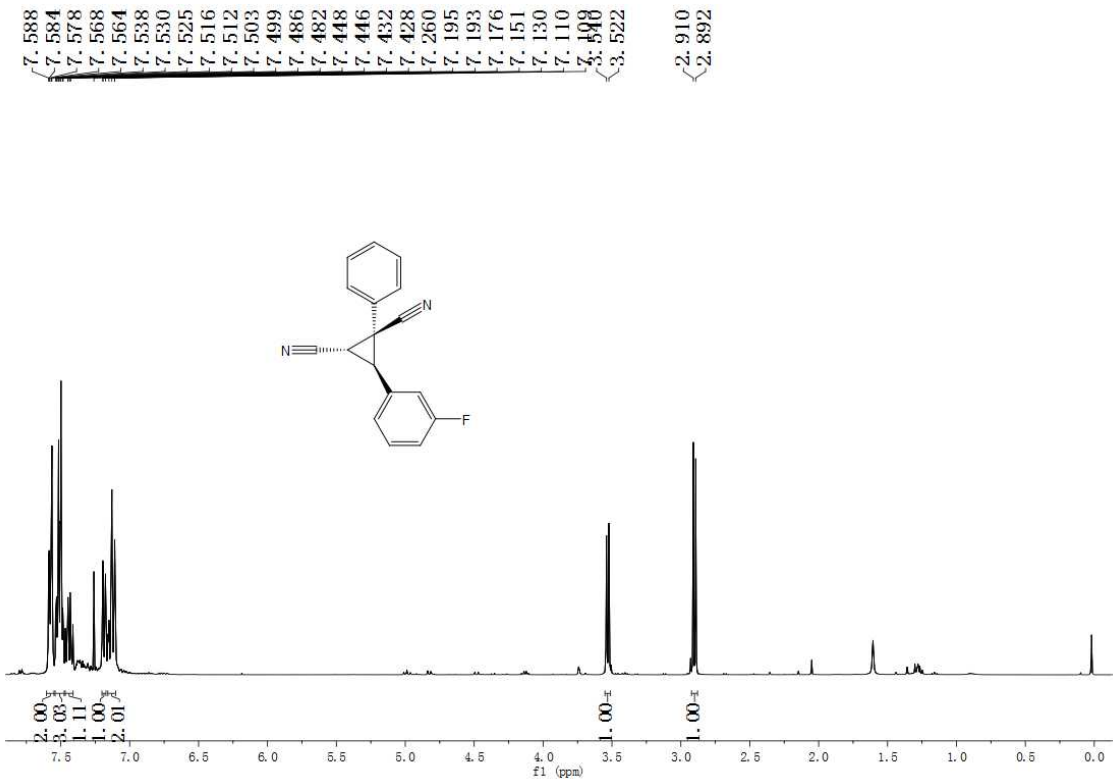

Figure S121. ^{1}H NMR Spectrum of *trans*-4k (400 MHz, $CDCl_3$)

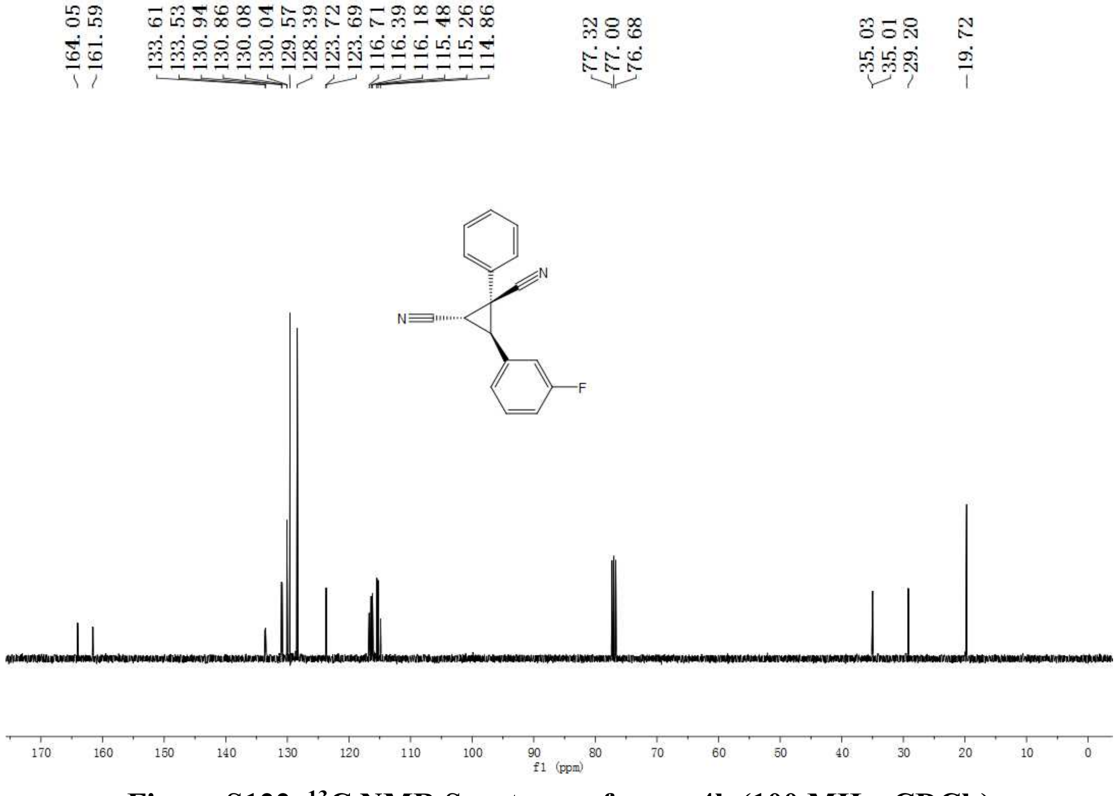

Figure S122. ^{13}C NMR Spectrum of *trans*-4k (100 MHz, $CDCl_3$)

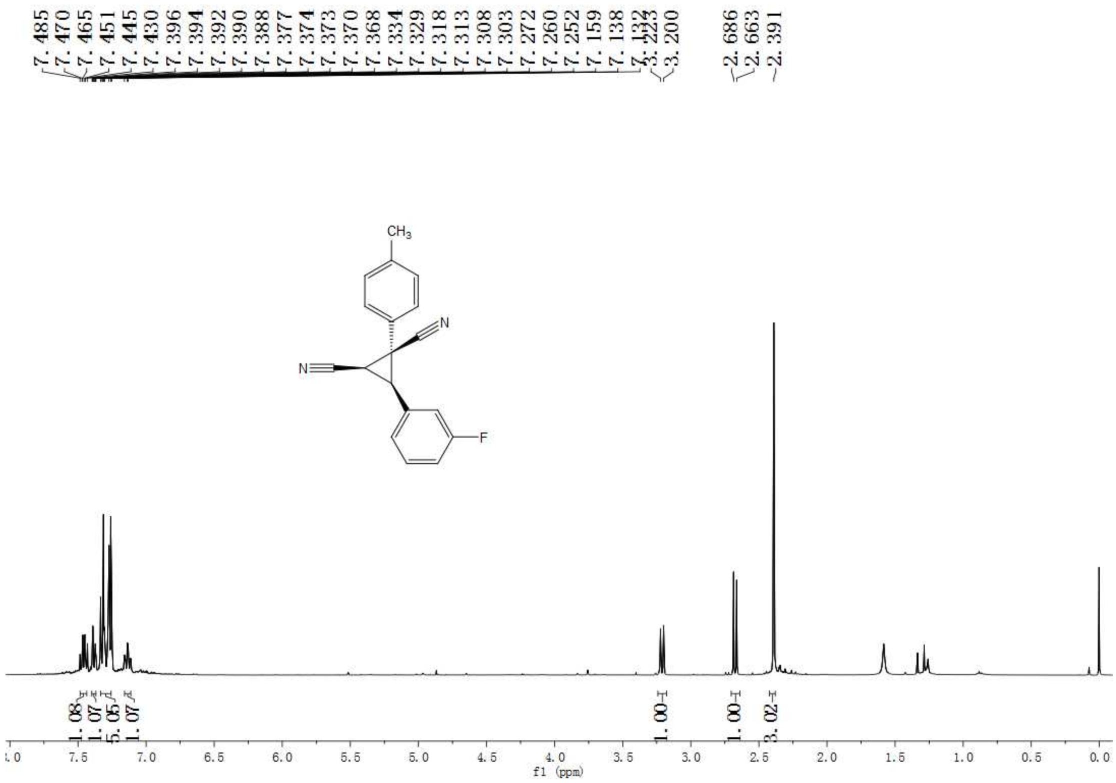

Figure S123. ^{1}H NMR Spectrum of *cis*-4l (400 MHz, $CDCl_3$)

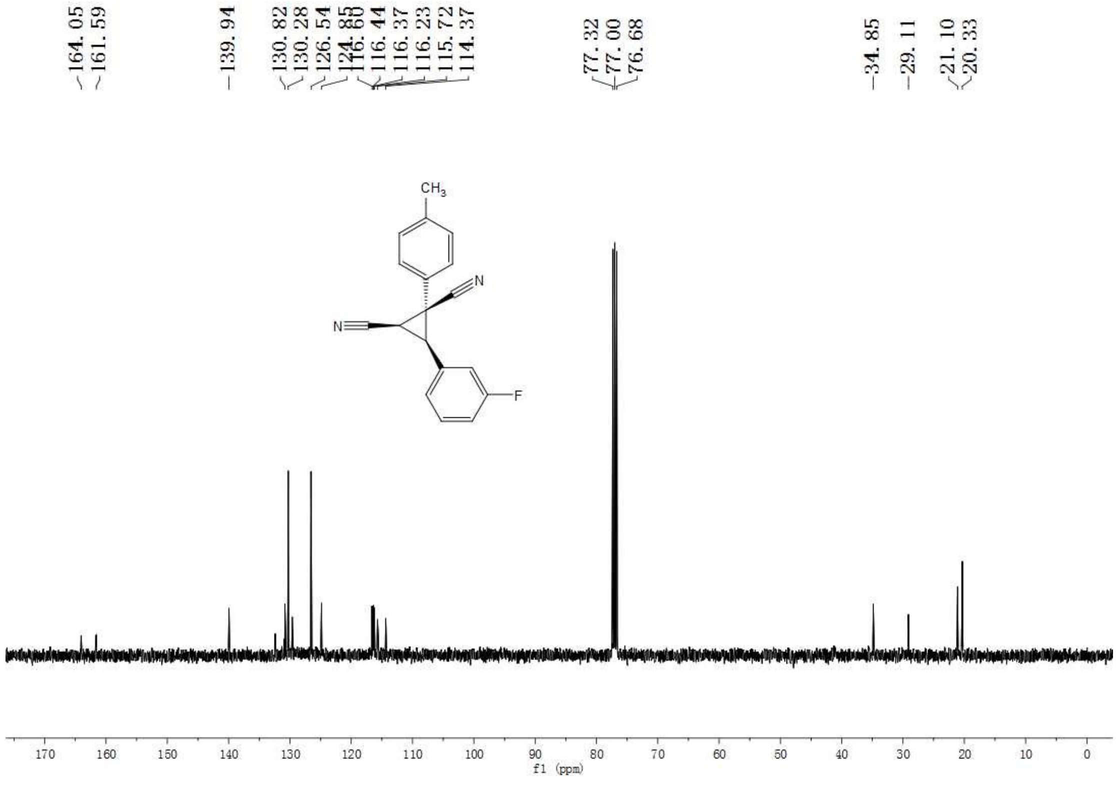

Figure S124. ^{13}C NMR Spectrum of *cis*-4l (100 MHz, $CDCl_3$)

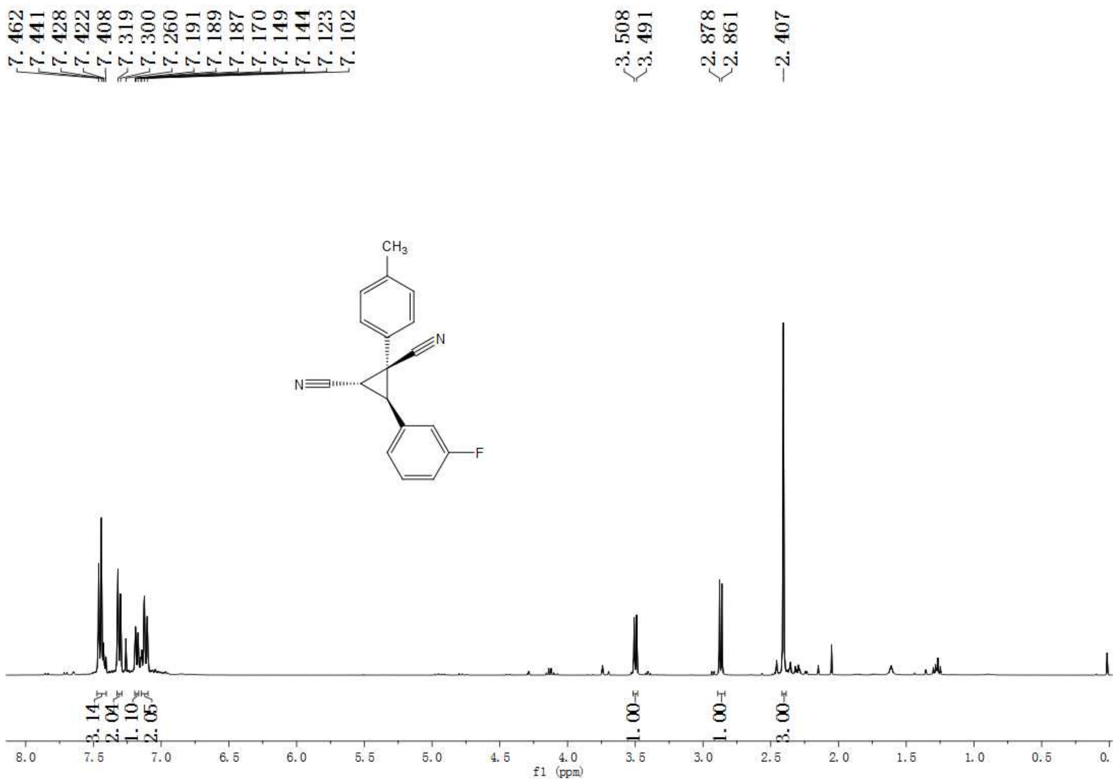

Figure S125. ^{1}H NMR Spectrum of *trans*-4l (400 MHz, $CDCl_3$)

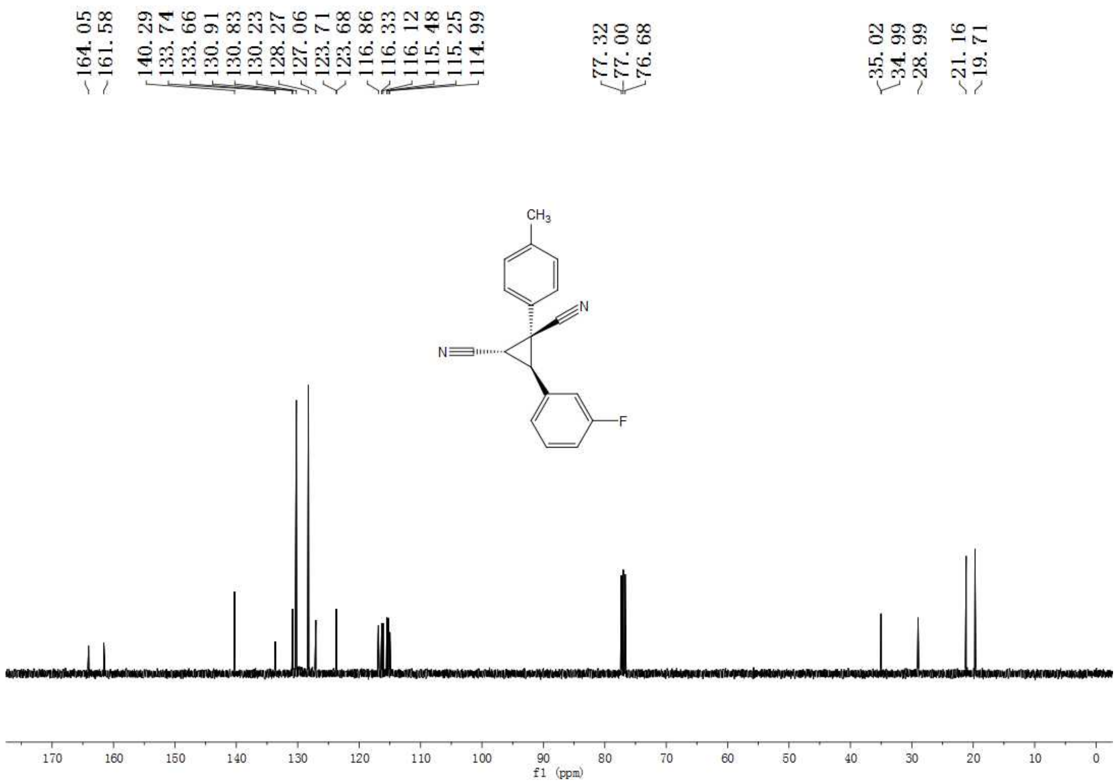

Figure S126. ^{13}C NMR Spectrum of *trans*-4l (100 MHz, $CDCl_3$)

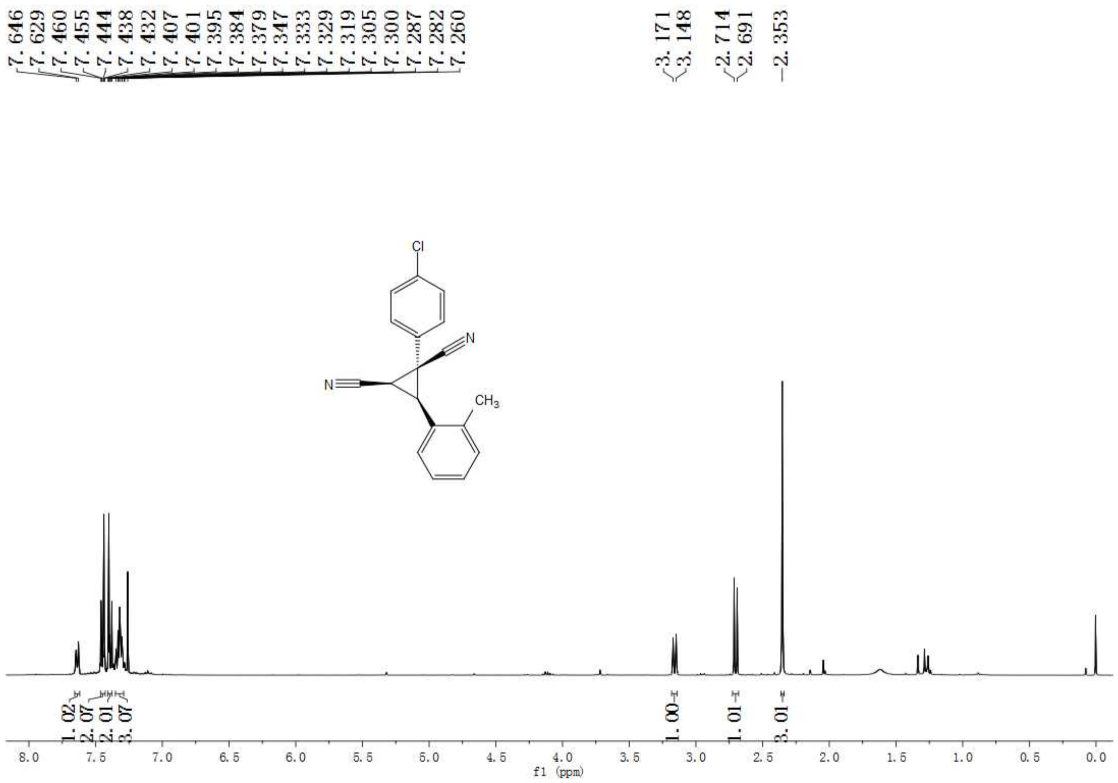

Figure S127. ^{1}H NMR Spectrum of *cis*-4m (400 MHz, $CDCl_3$)

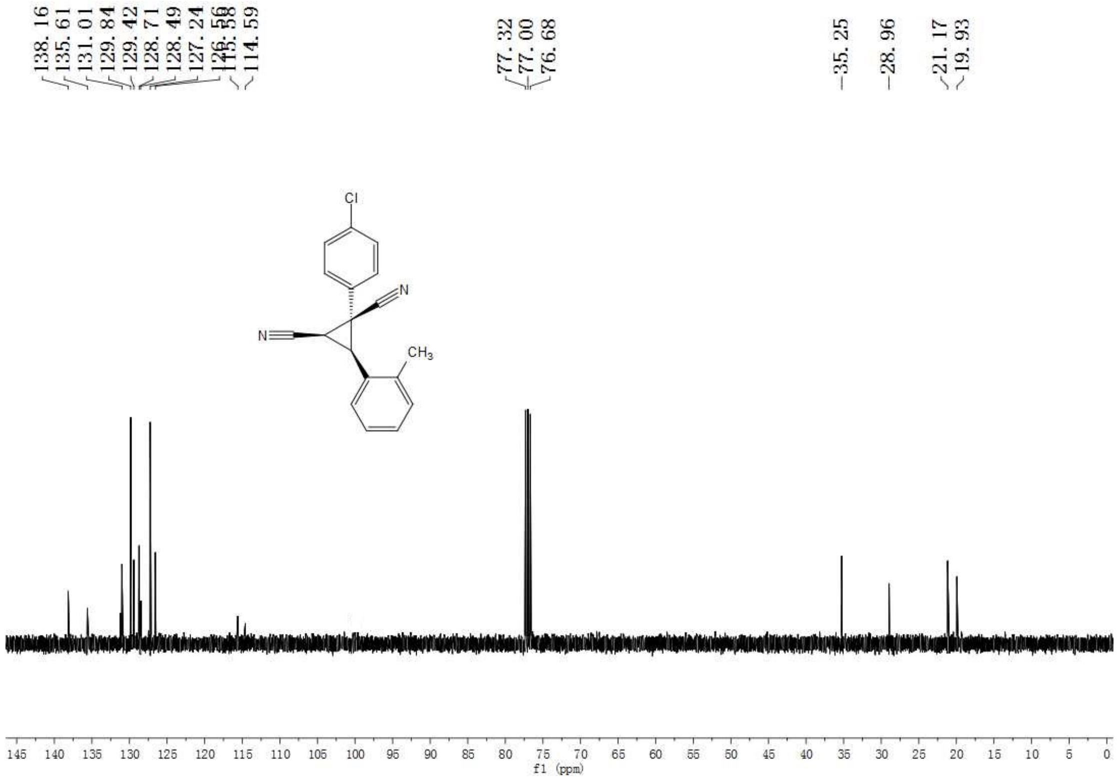

Figure S128. ^{13}C NMR Spectrum of *cis*-4m (100 MHz, $CDCl_3$)

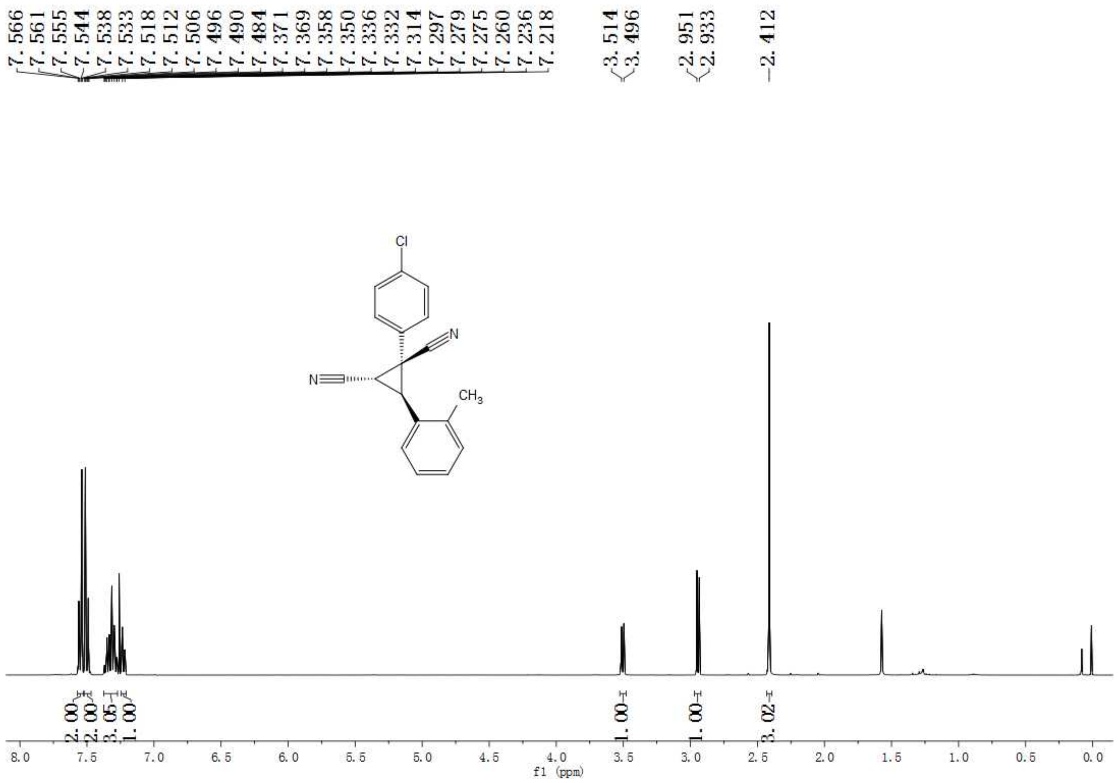

Figure S129. ^{1}H NMR Spectrum of *trans*-4m (400 MHz, $CDCl_3$)

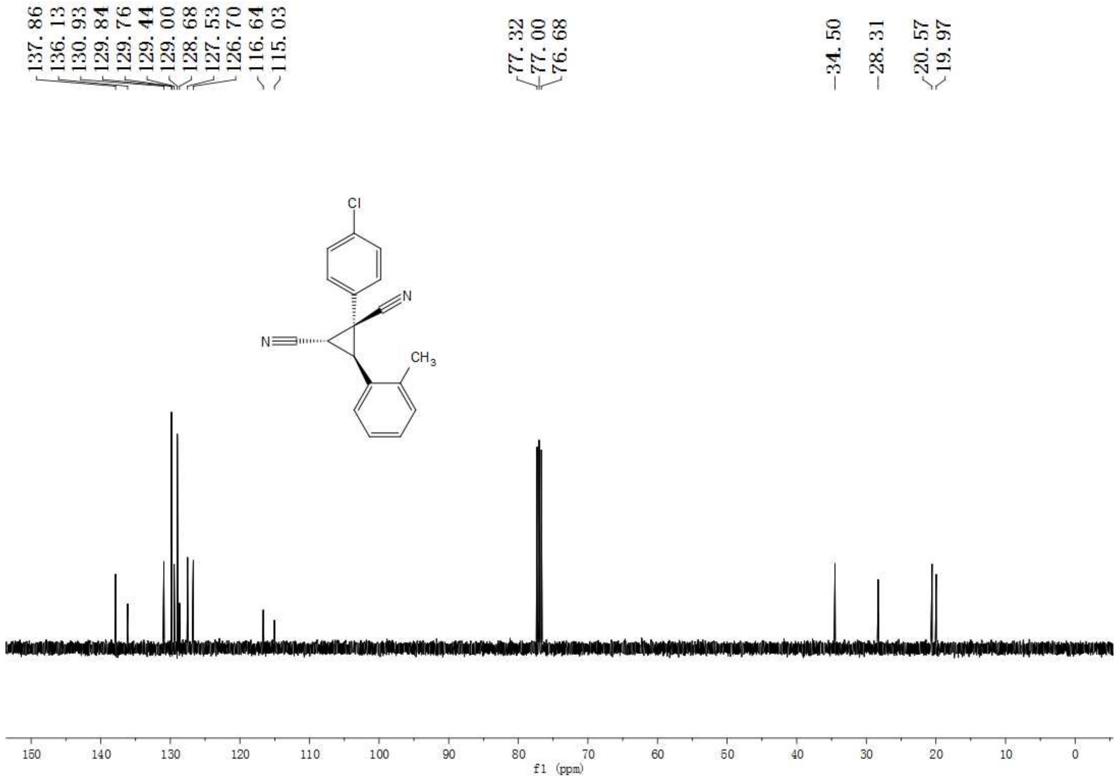

Figure S130. ^{13}C NMR Spectrum of *trans*-4m (100 MHz, $CDCl_3$)

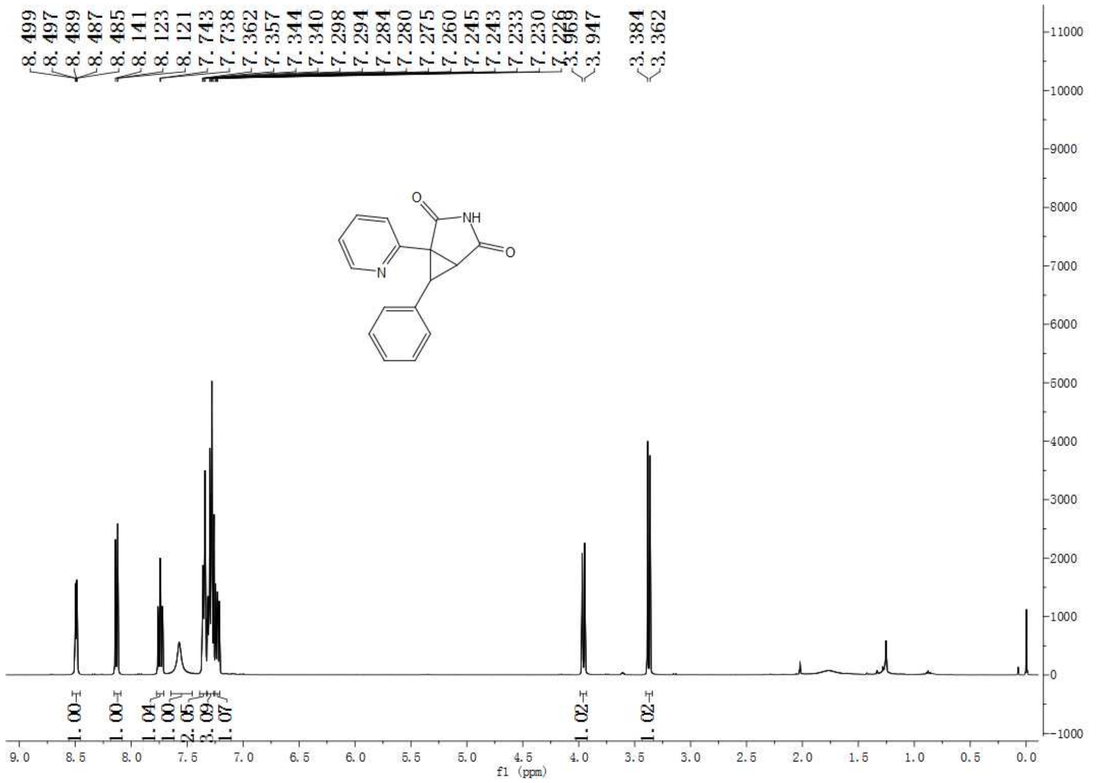

Figure S131. ^{1}H NMR Spectrum of 5a (400 MHz, $CDCl_3$)

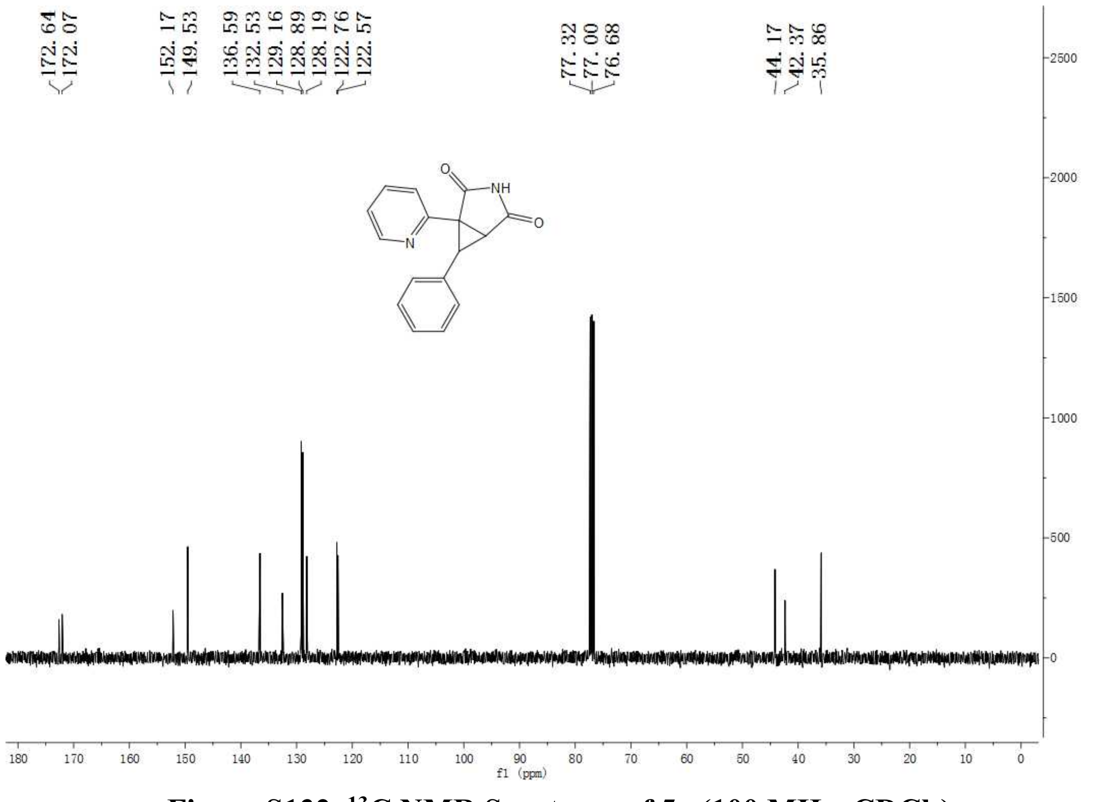

Figure S132. ^{13}C NMR Spectrum of 5a (100 MHz, $CDCl_3$)

G. X-ray crystallographic data

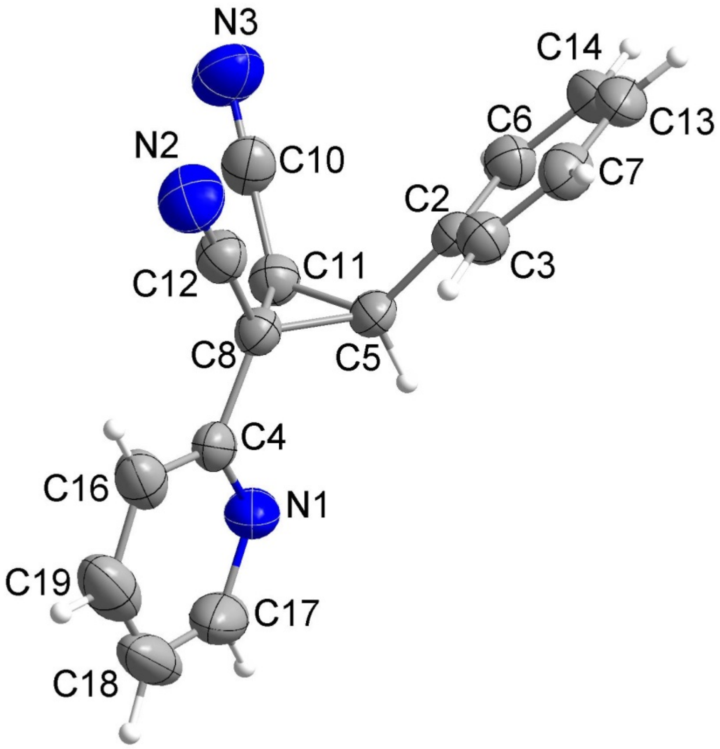

Figure S133. The Diamond diagram of *cis*-3a (thermal ellipsoids are shown at 50% probability)

Sample Preparation: A crystalline solid was obtained via slow evaporation of

compound **3a** in EA at room temperature.

Crystal data and structure refinement for compound ***cis*-3a** (CCDC: 2141258)

Table S1 Crystal data and structure refinement for *cis*-3a.

Identification code	***cis*-3a**
Empirical formula	$C_{16}H_{11}N_3$
Formula weight	245.28
Temperature/K	293(2)
Crystal system	monoclinic
Space group	$P2_1/n$
a/Å	9.3594(9)
b/Å	12.4742(12)
c/Å	11.0555(12)
α/°	90
β/°	95.258(4)
γ/°	90
Volume/Å^3	1285.3(2)
Z	4
ρ_{calc}g/cm^3	1.268
μ/mm^{-1}	0.078
F(000)	512.0
Crystal size/mm^3	0.2 × 0.2 × 0.2
Radiation	MoKα (λ = 0.71076)
2Θ range for data collection/°	6.364 to 55.156
Index ranges	$-12 \leq h \leq 12, -16 \leq k \leq 15, -14 \leq l \leq 14$
Reflections collected	16661
Independent reflections	2862 [R_{int} = 0.0779, R_{sigma} = 0.0562]
Data/restraints/parameters	2862/0/176
Goodness-of-fit on F^2	1.074
Final R indexes [I>=2σ (I)]	R_1 = 0.0629, wR_2 = 0.1279
Final R indexes [all data]	R_1 = 0.0961, wR_2 = 0.1408
Largest diff. peak/hole / e Å^{-3}	0.23/-0.16

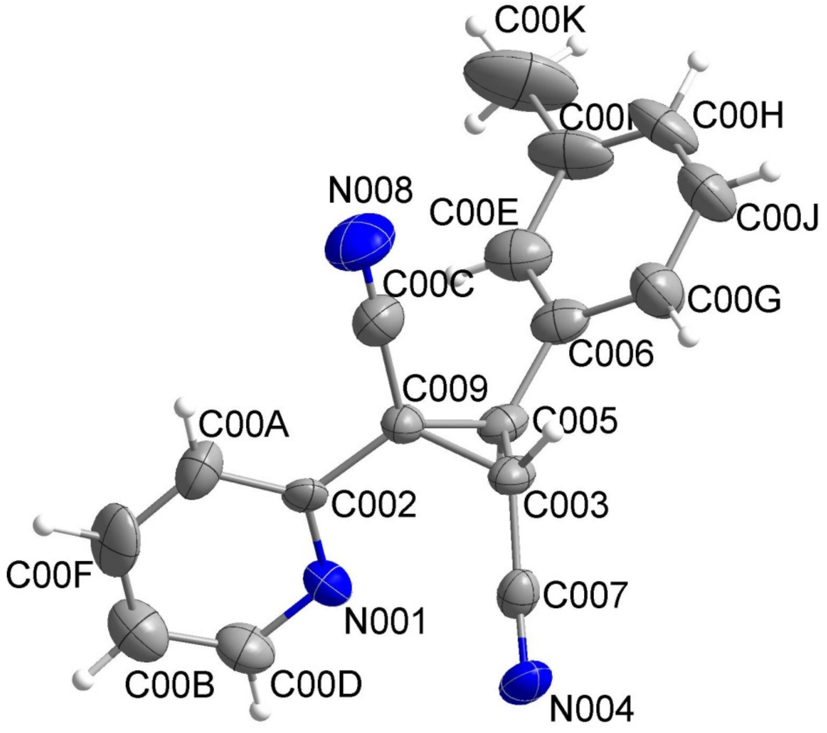

Figure S134. The Diamond diagram of *trans*-4b (thermal ellipsoids are shown at 50% probability)

Sample Preparation: A crystalline solid was obtained via slow evaporation of

compound ***trans*-4b** in EA at room temperature.

Crystal data and structure refinement for compound ***trans*-4b** (CCDC: 2142244)

Table S2 Crystal data and structure refinement for *trans*-4b.

Identification code	***trans*-4b**
Empirical formula	$C_{17}H_{13}N_3$
Formula weight	259.31
Temperature/K	273.15
Crystal system	orthorhombic
Space group	$Pna2_1$
a/Å	9.1611(17)
b/Å	28.828(6)
c/Å	5.3391(12)
α/°	90
β/°	90
γ/°	90
Volume/Å^3	1410.0(5)
Z	4
ρ_{calc}g/cm^3	1.2214
μ/mm^{-1}	0.074
F(000)	544.2
Crystal size/mm^3	0.2 × 0.2 × 0.2
Radiation	Mo Kα (λ = 0.71073)
2Θ range for data collection/°	5.66 to 50
Index ranges	$-11 \le h \le 11$, $-37 \le k \le 36$, $-6 \le l \le 6$
Reflections collected	17796
Independent reflections	2437 [R_{int} = 0.0486, R_{sigma} = 0.0477]
Data/restraints/parameters	2437/1/182
Goodness-of-fit on F^2	1.036
Final R indexes [I>=2σ (I)]	R_1 = 0.1207, wR_2 = 0.3122
Final R indexes [all data]	R_1 = 0.1316, wR_2 = 0.3193
Largest diff. peak/hole / e Å^{-3}	0.47/-0.46
